# Global burden of disease from cyclist road injuries in youth and young adults aged 15–39 years, 1990–2021

**DOI:** 10.3389/fpubh.2025.1581789

**Published:** 2025-05-12

**Authors:** Li Zhou, Ying Han

**Affiliations:** ^1^Department of Emergency, Ningbo Sixth Hospital, Ningbo, Zhejiang, China; ^2^Department of Anesthesiology Ward, Ningbo Sixth Hospital, Ningbo, Zhejiang, China

**Keywords:** cyclist road injuries (CRI), youth and young adult, age standardized incidence rate (ASIR), age standardized death (ASDR), age standardized disability adjusted life year (DALY)

## Abstract

**Background:**

Cyclist: individuals who ride bicycles as a mode of transportation or recreation. Cyclist Road Injuries (CRI): road traffic injuries sustained by cyclists, including collisions with motor vehicles, pedestrians, or other cyclists, as well as single-vehicle crash’s (e.g., falls or crashes without external collision). Detailed analyses of the global burden of CRI among youth and young adults aged 15–39 remain limited. This study evaluates the disease burden of CRI from 1990 to 2021 using Global Burden of Disease (GBD) 2021 data.

**Methods:**

Age-standardized incidence (ASIR), death (ASDR), and disability-adjusted life year (DALY) rates were calculated across 204 countries and 21 regions, stratified by socio-demographic index (SDI). Linear regression modeling estimated annual percentage changes (EAPC) to assess trends.

**Results:**

Globally, ASIR, ASDR, and DALY rates declined from 1990 to 2021 (EAPCs: −1.26, −0.38, −0.80). However, absolute numbers of death cases and DALYs increased by 26.63 and 11.19%, respectively. High-middle SDI regions had the highest ASIR (259.70) and DALY rate (53.77), while middle SDI regions showed the highest ASDR (0.68). East Asia exhibited the highest ASDR and DALY rates. Andean Latin America saw the largest increases (EAPCs: 0.85, 2.13, 1.43), whereas high-income Asia Pacific showed the most significant declines (EAPCs: −3.52, −6.37, −5.20).

**Conclusion:**

Despite declining rates, CRI burden remains significant, particularly in high-middle and middle SDI regions like East Asia and the Caribbean. Andean Latin America showed rising trends, while high-income Asia Pacific achieved substantial reductions.

## 1 Introduction

Over recent years, cycling has increasingly gained global recognition as a viable mode of transportation. Current data suggests that there are approximately 1 billion bicycles in use across the world, twice the number of automobiles (1). The widespread popularity of bicycles can be attributed to their environmentally friendly, health-enhancing, and convenient attributes, particularly their significant role in urban transportation. Cycling offers numerous health advantages to both individuals and society. It has been linked with a reduced risk of type 2 diabetes, cancer, and all-cause mortality (2, 3). Moreover, it is effective in decreasing cardiovascular disease (CVD) risk factors. Research indicates a dose–response relationship between cycling and all-cause mortality, suggesting that any level of cycling is beneficial (4, 5). In terms of risk versus reward, the health benefits of cycling outweigh the risk of injury by a factor of 21 and the risk of death by a factor of 238 (6). Concurrently, the economic benefits of developing cycling infrastructure are five times greater than its cost (7), indicating that the promotion of cycling not only has significant public health benefits but also substantial economic value.

Cyclist: individuals who ride bicycles as a mode of transportation or recreation. Cyclist Road Injuries (CRI): road traffic injuries sustained by cyclists, including collisions with motor vehicles, pedestrians, or other cyclists, as well as single-vehicle crash’s (e.g., falls or crashes without external collision) (7). As cycling prevalence increases, there is a corresponding rise in CRI. Research indicates that cyclists face twice the risk of fatal injuries per mile compared to automobile passengers (8). In fact, the likelihood of sustaining a fatal injury while cycling is 12 times greater than while driving a car (9). According to the World Health Organization (WHO) and other transportation research bodies, it was projected that approximately 41,000 individuals would die globally in 2021 due to bicycle-related crashes. This represents roughly 3% of the total road traffic fatalities worldwide (10). Concurrently, the number of cyclists suffering serious injuries from crashs is estimated to be in the millions. These severe injuries primarily include head trauma, spinal damage, and limb fractures, often necessitating long-term treatment or leading to permanent disability. Consequently, safety concerns are becoming a significant factor in the decision against cycling (11). Beyond physical harm and disability, cycling risk events also pose substantial economic challenges due to medical costs and property damage. The resultant economic and social losses are significant (12).

Cyclists aged 15 to 39 are particularly susceptible to road traffic crashs. This susceptibility can be attributed to their physiological attributes, behavioral traits and psychosocial influence (8, 10, 13). Physiologically, these individuals possess strong physical capabilities and frequently engage in cycling, especially during nighttime hours when low visibility and fatigue heighten the likelihood of crashs. Their inclination toward risk-taking, susceptibility to distraction, and inexperience render them a high-risk group in traffic scenarios. They often exhibit behaviors such as speeding, disregarding traffic signals, and are more likely to encounter crashs in intricate traffic situations. As primary users of e-bikes, the high speeds and inherent instability of their vehicles amplify the risks. Psychosocial factors, including peer pressure and an underestimation of risks, further exacerbate their hazardous behaviors. The confluence of these elements elevates the danger this age group faces in traffic mishaps. Consequently, it is imperative to prioritize addressing the disease burden of CRI among the 15-39-year-old demographic.

Previous Global Burden of Disease (GBD) studies have predominantly focused on the comprehensive burden of road traffic injuries (14, 15), with a relative lack of detailed analysis of specific injury types. To address this, the present study aims to analyze the age standardized incidence rate (ASIR), age standardized death (ASDR), and age standardized disability adjusted life year (DALY) indicators. This endeavor seeks to systematically evaluate the global burden of disease associated with CRI, providing a scientific foundation for the development of more precise and cost-effective prevention and intervention strategies.

## 2 Methods

### 2.1 Data sources

The GBD 2021 is a comprehensive health assessment program that offers estimates of the global, regional, and national burden of diseases, injuries, and risk factors. This study presents incidence, death, and DALYs estimates for 371 diseases and injuries spanning the years 1990 to 2021. Furthermore, GBD 2021 delivers updated demographic data for 204 countries and territories, along with an additional 811 subnational locations, for the period from 1950 to 2021. A particular focus has been placed on variations in death rates and life expectancy in 2021. The study employs a standardized methodology to estimate the rate of incidence, death, and DALYs indicators for CRIs. This estimation integrates various sources such as epidemiological data, hospital records, police reports, and death certificates from around the world, providing a comprehensive assessment of their disease burden. For the use of identified data in GBD study, a waiver of informed consent has been approved by the University of Washington Institutional Review Board. This study did not involve individual participants. The ethics approval can be found at https://www.healthdata.org/.

### 2.2 Definition of cases

In the GBD 2021, CRI is encompassed within the broader category of “transportation injuries. These injuries include both fatal and non-fatal outcomes (e.g., fractures, traumatic brain injuries, soft-tissue injuries). Data sources include police reports, hospital records, and mortality registries, aligned with GBD injury surveillance standards. The International Classification of Diseases (ICD)-10 codes V10-V19.9define CRI, are utilized to classify the external causes of injuries or deaths resulting from pedal cycle (bicycle) accidents. These codes encompass both collision and non-collision incidents, including specific categories such as collisions with pedestrians, animals, motor vehicles, or stationary objects, as well as non-collision accidents like falls (16). By providing a comprehensive framework for categorizing bicycle-related events, these codes support epidemiological research, burden of disease assessment (e.g., calculating DALYs), and the development of targeted public health and road safety policies.

The Socio-Demographic Index (SDI) serves as a composite indicator for evaluating societal development levels. Derived from data sources such as average per capita income, the mean educational attainment of those aged 15 and above, and the total fertility rate, the SDI typically ranges from 0 to 1. This range facilitates an assessment of a region’s socio-demographic progress. Depending on the SDI value, countries and regions can be classified into distinct categories: low, low-middle, middle, high-middle, and high (17).

### 2.3 Statistical methods

ASIR/ASDR calculation: Rates were computed using the direct standardization method, with the WHO 2000–2025 global standard population as the denominator. Numerators included injury cases (for ASIR) and fatalities (for ASDR) stratified by age groups. Incidence determination: Incident cases were defined as medically attended injuries confirmed through hospital records and trauma registries, excluding non-clinical/self-reported events. Data scope: Analyses focused on non-motorized bicycle injuries (excluding e-bikes), with police-reported collision data used only for severity validation. DALYs are computed by summing years of healthy life lost due to illness, injury, or disability (YLDs) and years of life lost due to premature death (YLLs). Healthy life expectancy (HALE) estimates are calculated using per capita YLDs and age-specific death rates, taking into account location, age, sex, year, and cause. To ensure statistical reliability, 95% uncertainty intervals (UIs) for all final estimates were established, based on the 2.5th and 97.5th percentile values from a total of 500 samples.

The estimated the annual percentage change (EAPC) serves as a statistical tool employed to quantify the trend of a disease indicator, such as incident, death, or disability-adjusted life years, over a distinct time frame (18). It was calculated by fitting a regression line to the natural logarithm of the rates (y = *α* + *β*x + *ε*), this measure illustrates the rate of escalation or de-escalation of the indicator, utilizing the subsequent formula:


EAPC=(eβ−1)×100

In the regression model, β represents the coefficient associated with the time variable, while e corresponds to the base of the natural logarithm, approximately 2.718. Using a linear regression model, one can fit the natural logarithmic value of the disease indicator to time, subsequently determining the EAPC by calculating *β*. For the computation of the EAPC and the corresponding 95% confidence intervals (CIs), the R (4.21) programming language is employed. Data Quality Control: GBD 2021 data were validated through multi-source integration (hospital records, police reports, death registries) and corrected for missing values or misreporting using Bayesian meta-regression models (DisMod-MR; see GBD methodology). Sensitivity Analysis: Recalculating EAPC after excluding outliers (e.g., countries with >20% missing data) yielded robust results (<5% variation).

## 3 Results

### 3.1 Global burden of CRI among youth and young adults aged 15–39 years

**Figure 1:**
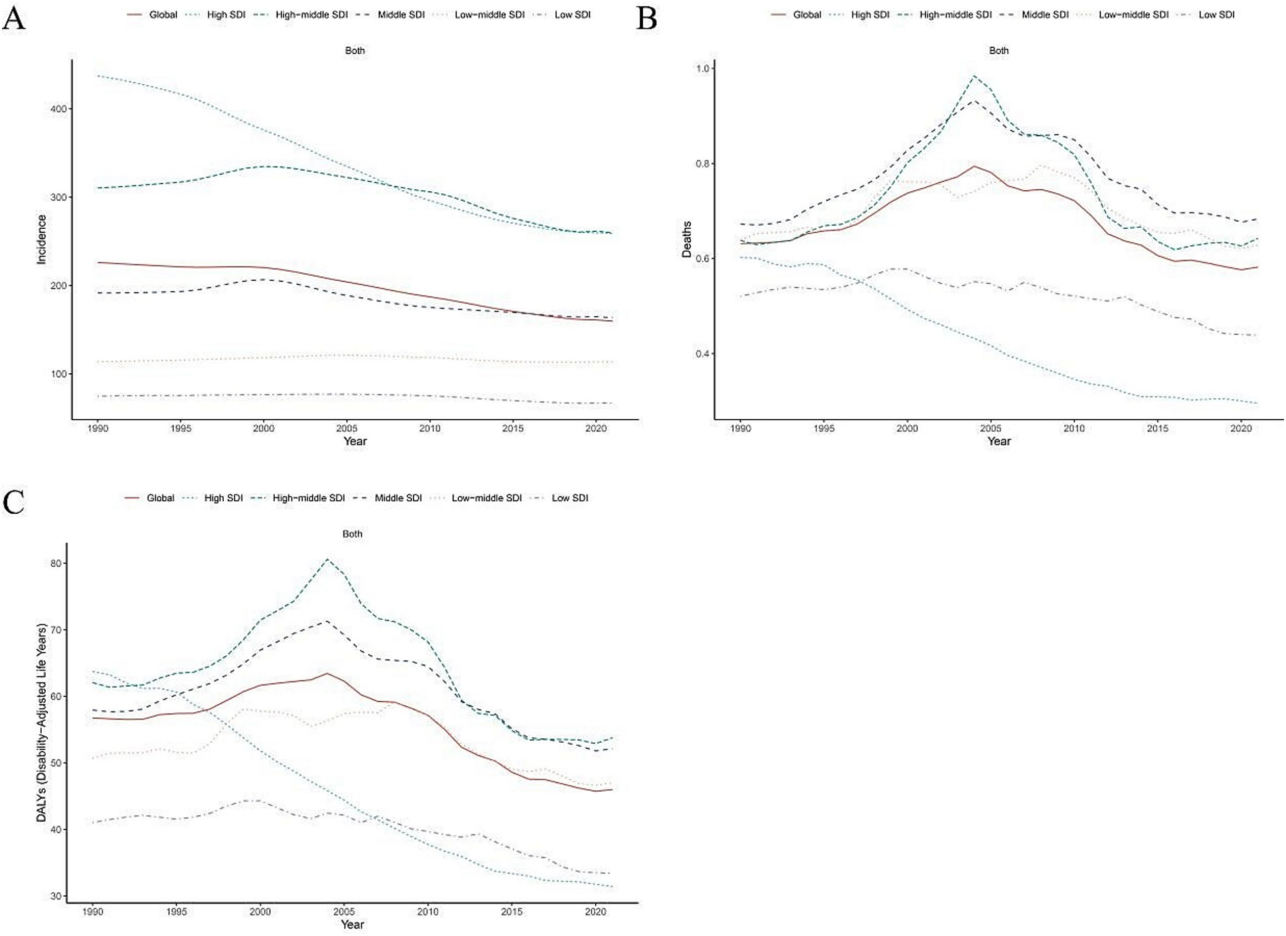
Age-standardized rate trends in incidence, disability-adjusted life-years and deaths of cyclist road injuries from 1990 to 2021 by global and SDI regions. ASIR, age standardized incidence rate. ASDR, age standardized death rate. DALY, disability adjusted life-year. **(A)** ASIR, **(B)** ASDR, and **(C)** age standardized DALY rate.

In 2021, global estimates for young adults aged 15–39 with CRI showed 47,482,686.8 incident cases, 17,431,881.34 deaths, and 13,756,333.34 absolute DALYs, compared to 1990 figures of 4,995,943.51, 13,765,474.73, and 12,372,277.37, respectively. While the incidence rate decreased by −4.96%, the death rate increased by 26.63%, and absolute DALYs rose by 11.19% between 1990 and 2021. ASIR, ASDR, and age-standardized DALY rates in 2021 were 159.69 (95% UI: 159.55–159.84), 0.58 (0.57–0.59), and 46.00 (45.92–46.07), respectively, reflecting declines of −29.38, −7.94%, and −18.94% from 1990 levels (ASIR: 226.12; ASDR: 0.63; DALY: 56.75; Table 1; Figure 1; Supplementary Table S1; Supplementary Figure S1).

**Table 1:** Age-standardized rate and its trends of incidence, deaths and DALYs of cyclist road injuries aged 15–39 in global and regions, 1990 to 2021.

	Incidence	Incidence	Incidence	Deaths	Deaths	Deaths	DALYs (Disability-adjusted life years)	DALYs (Disability-adjusted life years)	DALYs (Disability-adjusted life years)
	1990	2021		1990	2021		1990	2021	
	ASIR			ASDR			Age-standardized DALY rate (per 100,000) No.95%UI		
Location	1990 No.(95%UI)	2021 No.(95%UI)	1990–2021 EAPC No.(95%CI)	1990 No.(95%UI)	2021 No.(95%UI)	1990–2021 EAPC No.(95%CI)	1990 No.(95%UI)	2021 No.(95%UI)	1990–2021 EAPC No.(95%CI)
Global									
Both	226.12(225.92,226.32)	159.69(159.55,159.84)	−1.26(−1.38,−1.14)	0.63(0.62,0.64)	0.58(0.57,0.59)	−0.38(−0.76,0.00)	56.75(56.65,56.85)	46.00(45.92,46.07)	−0.80(−1.10,−0.50)
Female	150.47(150.24,150.70)	96.06(95.90,96.21)	−1.66(−1.83,−1.49)	0.27(0.26,0.28)	0.18(0.18,0.19)	−1.57(−2.11,−1.02)	29.55(29.45,29.66)	18.15(18.08,18.22)	−1.89(−2.27,−1.51)
Male	300.04(299.72,300.36)	221.42(221.18,221.65)	−1.08(−1.18,−0.98)	0.98(0.97,1.00)	0.97(0.95,0.98)	−0.11(−0.46,0.24)	83.31(83.14,83.49)	73.04(72.91,73.18)	−0.48(−0.77,−0.20)
SDI									
High-middle SDI	310.45(309.93,310.96)	259.70(259.21,260.18)	−0.70(−0.91,−0.50)	0.64(0.61,0.66)	0.64(0.62,0.67)	−0.15(−0.76,0.46)	62.07(61.84,62.30)	53.77(53.55,53.99)	−0.64(−1.10,−0.19)
High SDI	437.20(436.49,437.91)	258.84(258.29,259.38)	−1.95(−2.03,−1.86)	0.60(0.58,0.63)	0.30(0.28,0.31)	−2.71(−2.89,−2.54)	63.74(63.47,64.01)	31.42(31.23,31.60)	−2.61(−2.74,−2.49)
Low-middle SDI	113.73(113.42,114.04)	113.60(113.37,113.83)	−0.05(−0.15,0.04)	0.64(0.62,0.66)	0.63(0.61,0.65)	−0.10(−0.43,0.22)	50.69(50.48,50.90)	47.03(46.88,47.18)	−0.30(−0.58,−0.02)
Low SDI	74.84(74.45,75.24)	67.13(66.89,67.37)	−0.40(−0.53,−0.27)	0.52(0.49,0.56)	0.44(0.42,0.46)	−0.62(−0.83,−0.42)	41.02(40.72,41.31)	33.41(33.24,33.59)	−0.73(−0.92,−0.54)
Middle SDI	191.60(191.29,191.92)	163.58(163.31,163.84)	−0.70(−0.86,−0.54)	0.67(0.65,0.69)	0.68(0.67,0.70)	−0.02(−0.47,0.42)	57.94(57.77,58.12)	52.15(52.00,52.30)	−0.44(−0.79,−0.08)
Regions									
Andean Latin America	107.79(106.15,109.45)	124.89(123.57,126.23)	0.85(0.70,1.00)	0.35(0.26,0.47)	0.49(0.41,0.58)	2.13(1.50,2.76)	32.09(31.18,33.02)	38.11(37.38,38.85)	1.43(0.95,1.91)
Australasia	325.34(321.39,329.33)	183.31(180.65,186.01)	−2.18(−2.35,−2.02)	0.49(0.35,0.67)	0.15(0.09,0.25)	−3.61(−3.92,−3.29)	49.68(48.15,51.24)	18.21(17.40,19.06)	−3.30(−3.54,−3.06)
Caribbean	332.68(329.77,335.62)	188.87(186.88,190.89)	−2.09(−2.26,−1.92)	2.83(2.57,3.12)	0.84(0.71,0.99)	−4.63(−5.25,−4.02)	205.90(203.60,208.23)	67.55(66.36,68.76)	−4.23(−4.79,−3.67)
Central Asia	99.18(98.03,100.35)	90.82(89.84,91.81)	−0.11(−0.18,−0.03)	0.24(0.19,0.31)	0.23(0.18,0.28)	0.48(−0.53,1.49)	23.75(23.18,24.34)	21.17(20.71,21.64)	0.04(−0.51,0.60)
Central Europe	417.53(415.65,419.40)	287.88(286.02,289.74)	−1.48(−1.67,−1.28)	0.82(0.74,0.91)	0.50(0.42,0.58)	−2.45(−2.94,−1.95)	83.59(82.76,84.42)	48.66(47.92,49.40)	−2.35(−2.71,−1.99)
Central Latin America	196.36(195.31,197.41)	161.35(160.57,162.13)	−0.33(−0.52,−0.14)	0.61(0.55,0.67)	0.46(0.42,0.50)	−1.16(−1.46,−0.85)	56.12(55.55,56.69)	39.97(39.58,40.36)	−1.12(−1.42,−0.82)
Central Sub-Saharan Africa	56.64(55.62,57.68)	49.98(49.39,50.58)	−0.52(−0.58,−0.46)	0.24(0.18,0.33)	0.25(0.21,0.30)	0.37(0.19,0.56)	22.78(22.12,23.45)	21.30(20.90,21.70)	−0.08(−0.19,0.03)
East Asia	283.69(283.25,284.14)	300.40(299.89,300.90)	−0.06(−0.29,0.16)	0.77(0.75,0.80)	1.08(1.05,1.11)	0.89(0.22,1.57)	70.26(70.03,70.48)	82.13(81.87,82.39)	0.28(−0.24,0.81)
Eastern Europe	279.58(278.44,280.73)	207.03(205.86,208.20)	−0.94(−1.10,−0.78)	0.45(0.41,0.50)	0.22(0.18,0.26)	−1.95(−2.77,−1.13)	49.23(48.76,49.70)	26.72(26.32,27.12)	−1.79(−2.34,−1.23)
Eastern Sub-Saharan Africa	65.24(64.64,65.85)	56.67(56.31,57.02)	−0.59(−0.68,−0.50)	0.61(0.55,0.67)	0.54(0.51,0.58)	−0.60(−0.69,−0.51)	46.33(45.82,46.84)	39.51(39.21,39.81)	−0.71(−0.79,−0.63)
High-income Asia Pacific	574.12(572.32,575.93)	227.24(225.88,228.61)	−3.52(−3.72,−3.32)	0.84(0.77,0.91)	0.16(0.13,0.21)	−6.37(−6.75,−5.98)	83.70(83.01,84.39)	21.71(21.30,22.13)	−5.20(−5.49,−4.91)
High-income North America	408.72(407.50,409.94)	279.63(278.68,280.57)	−1.40(−1.62,−1.18)	0.47(0.43,0.52)	0.25(0.22,0.28)	−2.02(−2.48,−1.56)	55.04(54.61,55.49)	28.76(28.46,29.06)	−2.15(−2.47,−1.83)
North Africa and Middle East	116.52(115.94,117.10)	107.64(107.24,108.05)	−0.18(−0.21,−0.14)	0.10(0.09,0.12)	0.12(0.11,0.13)	1.34(0.59,2.09)	18.54(18.30,18.78)	15.40(15.25,15.56)	−0.22(−0.54,0.09)
Oceania	72.37(69.16,75.70)	60.72(58.70,62.79)	−0.78(−0.86,−0.70)	0.09(0.01,0.33)	0.08(0.02,0.20)	−0.23(−0.33,−0.14)	11.96(10.65,13.41)	10.17(9.35,11.05)	−0.59(−0.65,−0.54)
South Asia	112.03(111.71,112.35)	119.04(118.80,119.28)	0.13(−0.02,0.28)	0.82(0.80,0.85)	0.77(0.75,0.79)	−0.27(−0.61,0.06)	61.34(61.11,61.58)	55.35(55.18,55.51)	−0.39(−0.70,−0.08)
Southeast Asia	140.77(140.24,141.29)	109.35(108.96,109.75)	−0.92(−1.03,−0.81)	0.52(0.49,0.55)	0.56(0.54,0.59)	−0.08(−0.30,0.14)	45.29(44.99,45.60)	42.67(42.43,42.91)	−0.46(−0.62,−0.30)
Southern Latin America	316.48(313.97,319.01)	326.75(324.52,328.98)	0.05(−0.07,0.17)	0.39(0.30,0.49)	0.28(0.22,0.35)	−1.25(−1.89,−0.60)	44.70(43.75,45.66)	34.39(33.68,35.12)	−1.03(−1.45,−0.60)
Southern Sub-Saharan Africa	90.58(89.30,91.89)	74.37(73.46,75.30)	−0.71(−0.85,−0.56)	0.44(0.35,0.54)	0.42(0.35,0.49)	−0.30(−1.01,0.42)	33.55(32.74,34.38)	30.88(30.30,31.47)	−0.43(−0.99,0.14)
Tropical Latin America	199.04(197.95,200.13)	196.51(195.57,197.45)	−0.08(−0.32,0.16)	0.49(0.43,0.54)	0.57(0.52,0.62)	0.75(−0.16,1.67)	46.51(45.98,47.05)	47.89(47.43,48.34)	0.27(−0.48,1.02)
Western Europe	459.25(458.12,460.37)	263.06(262.15,263.97)	−1.98(−2.11,−1.85)	0.53(0.49,0.57)	0.18(0.16,0.21)	−3.85(−4.07,−3.64)	57.80(57.41,58.20)	24.89(24.62,25.17)	−3.00(−3.13,−2.86)
Western Sub-Saharan Africa	45.84(45.34,46.34)	44.89(44.59,45.20)	−0.07(−0.16,0.02)	0.28(0.24,0.33)	0.29(0.27,0.32)	0.24(0.12,0.36)	23.38(23.02,23.75)	22.52(22.30,22.74)	−0.02(−0.13,0.08)

On a global scale, the proportion of cases number for incidence, deaths and DALYs of youth and young adults aged 15–39 with CRI was 0.95, 1.27 and 1.11 from 1990 to 2021. The ASIR, ASDR, and age-standardized DALY rates for youth and young adults aged 15–39 with CRI have demonstrated a decreasing trend from 1990 to 2021. The corresponding EAPCs were −1.26 (95% CI: −1.38, −1.14), −0.38 (95% CI: −0.76, 0.00), and −0.80 (95% CI: −1.10, −0.50; Table 1; Figure 1; Supplementary Table S1).

### 3.2 Global burden of CRI among youth and young adults aged 15–39 years in different SDI regions

Globally, the ASIR for youth and young adults aged 15–39 years in 2021 was highest in the high-middle SDI bracket at 259.70 (95% UI: 259.21, 260.18) and lowest in the low SDI bracket at 67.13 (95% UI: 66.89, 67.37). The ASDR was highest in the middle SDI at 0.68 (95% UI: 0.67, 0.70) and lowest in the high SDI at 0.30 (95% UI: 0.28, 0.31). The age standardized DALY rate was highest at the high-middle SDI at 53.77 (95% UI: 53.55, 53.99) and lowest at the high SDI at 31.42 (95% UI: 31.23, 31.60; Table 1; Figure 1).

Globally, the ASIR, ASDR and age-standardized DALY rate for youth and young adults aged 15–39 with CRI showed a decline across all five SDI regions from 1990 to 2021. The most significant decrease was observed in the high SDI region, with an EAPC for ASIR of −1.95 (95% CI: −2.03, −1.86), with an EAPC for ASDR of −2.71 (95% CI: −2.89, −2.54) and an EAPC for age-standardized DALY rate of −2.61 (95% CI: −2.74, −2.49). Conversely, the least pronounced decline for the ASIR and age-standardized DALY rate was noted in the Low-middle SDI region, where the EAPC for ASIR was −0.05 (95% CI: −0.15, 0.04) and for the age-standardized DALY rate was −0.30 (95% CI: −0.58, −0.02). Meanwhile, the least pronounced decline for ASDR in middle SDI region, with an EAPC of −0.02 (95% CI: −0.47, 0.42; Table 1; Figure 1).

### 3.3 Burden of youth and young adult CRI by gender and age group

Globally, in 2021, the number of incidence, death and absolute DALYs among youth and young adults aged 15–39 with CRI was notably higher for males compared to females. Specifically, the absolute figures for males stood at 3340112.93, 1107391.73, and 14705.11, respectively. In contrast, the corresponding figures for females were 1408155.75, 268241.62, and 2726.78. The growth in absolute numbers was considerably more pronounced for males (0.99, 1.20, and 1.35) than for females (0.86, 0.85, and 0.94; Table 1).

In 2021, globally, the ASIR, ASDR, and age-standardized DALY rates for youth and young adults aged 15–39 with CRI were notably higher among males compared to females. Specifically, the values for these three metrics were 221.42 (221.18, 221.65), 0.97 (0.95, 0.98), and 73.04 for males, in contrast to 96.06 (95.90, 96.21), 0.18 (0.18, 0.19), and 18.15 (18.08, 18.22) for females, respectively. Over the period from 1990 to 2021, there was a global decreasing trend in the ASIR and age-standardized DALY rate of CRIs for both youth and young adult men and women. This decline was more pronounced among females, with EAPC values of −1.66, −1.57, and −1.89. For males, the decrease was relatively slower, with EAPC values of −1.08, −0.11, and −0.48 (Table 1).

**Figure 2:**
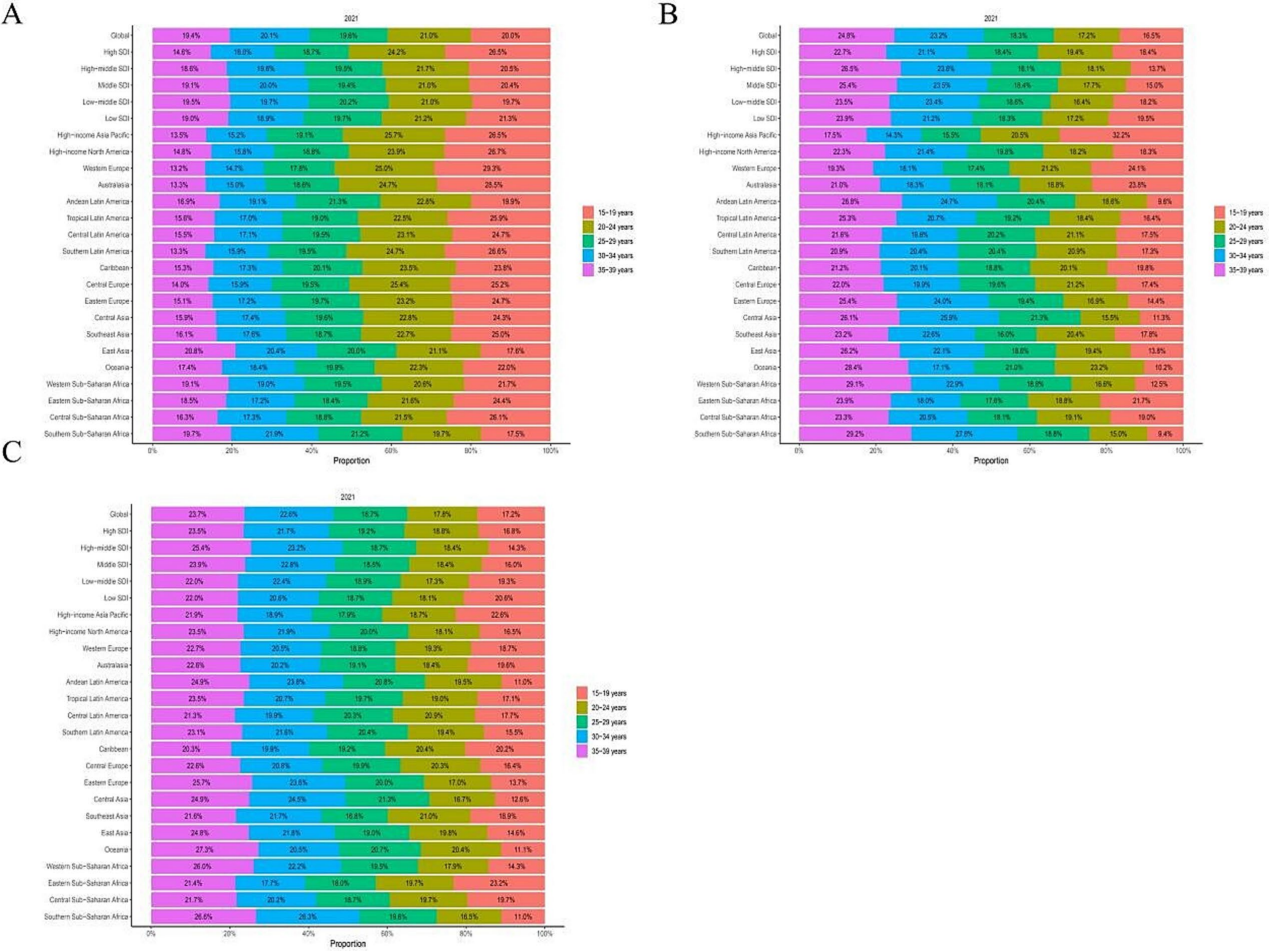
Global and regional age-specific incidence, deaths and DALYs rate of cyclist road injuries in 2021. DALY, disability adjusted life-year. **(A)** Incidence rate. **(B)** Deaths rate. **(C)** DALYs rate.

In 2021, globally, the incidence rate of CRI among youth and young adults aged 15–39 years peaked in the 20–24 age group (21.0%). Concurrently, death rate was highest in the 35–39 age bracket (24.8%), and this same group recorded the highest DALY rate (23.7%). As age increased, both death rate and DALY rates also rose. Moreover, when compared to other SDI regions, the low and medium-low SDI areas exhibited relatively elevated death rate and DALY rates for ages 15–19 (Supplementary Tables S2–S4; Figure 2).

### 3.4 Trends in CRI burden among youth and young adults aged 15–39 in 21 geographic areas

**Figure 3:**
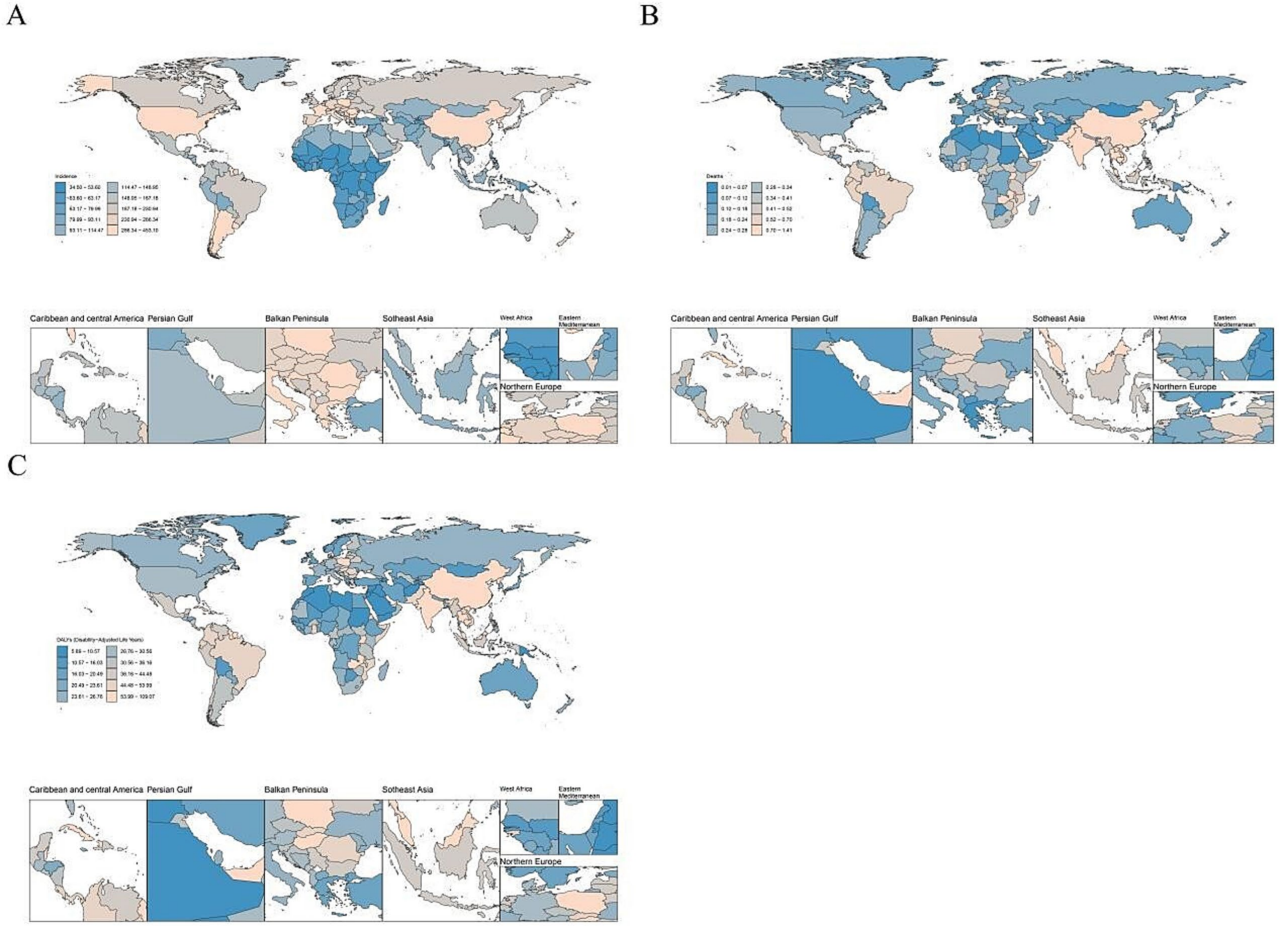
The global disease burden of cyclist road injuries in 204 countries and territories in 2021. ASIR, age standardized incidence rate. ASDR, age standardized death rate. DALY, disability adjusted life-year. **(A)** ASIR, **(B)** ASDR, and **(C)** age standardized DALY rate.

**Figure 4:**
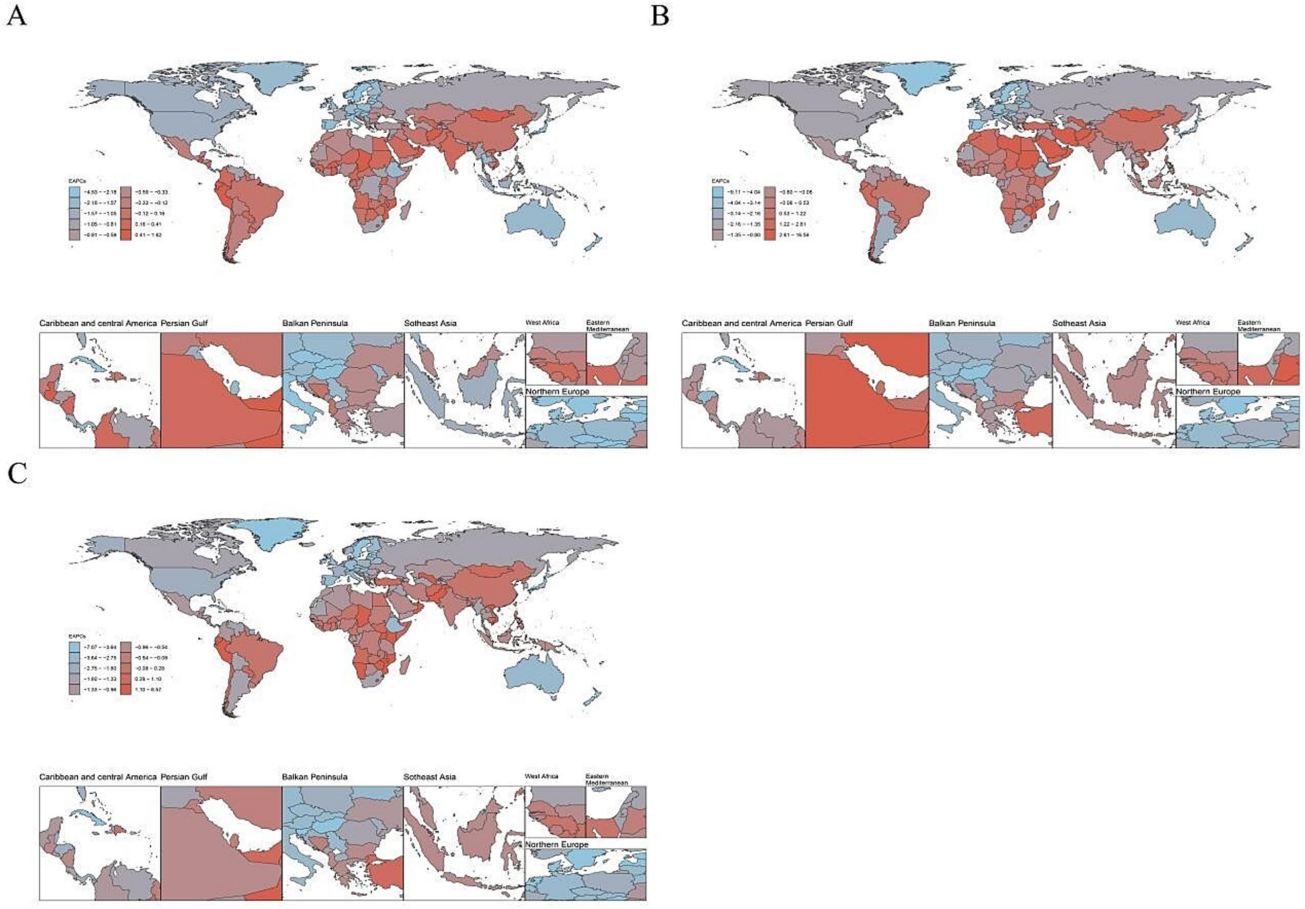
The estimated annual percentage changes (EAPC) of age standardized rate of cyclist road injuries in 204 countries and territories in 2021. ASIR, age standardized incidence rate. ASDR, age standardized death rate. DALY, disability adjusted life-year. **(A)** EAPC of ASIR, **(B)** EAPC of ASDR, and **(C)** EAPC of age standardized DALY rate.

Among the 21 regions, the ASIR for youth and young adult CRI aged 15–39 years was highest in Southern Latin America (326.75), East Asia (300.40), and Central Europe (287.88). Conversely, the regions with the lowest burden were Western sub-Saharan Africa (44.89), Central sub-Saharan Africa (49.98), and Eastern Sub-Saharan Africa (56.67). Globally, from 1990 to 2021, the ASIR for youth and young adult CKD saw the most significant increases in Andean Latin America (EAPC = 0.85), South Asia (EAPC = 0.13), and Southern Latin America (EAPC = 0.05). On the other hand, decreases were observed in high-income Asia Pacific (EAPC = −3.52), Australasia (EAPC = −2.18), and the Caribbean (EAPC = −2.09; Table 1; Figures 3A, 4A).

Among the 21 regions examined, the ASDR for youth and young adult CRI cases aged 15–39 was highest in East Asia (1.08), followed by the Caribbean (0.84) and South Asia (0.77). The lowest rates were observed in Oceania (0.08), North Africa and the Middle East (0.12), and Australasia (0.15). Globally, from 1990 to 2021, the ASDR for youth and young adult CRIs aged 15–39 was highest in Andean Latin America (EAPC = 2.13), North Africa and the Middle East (EAPC = 1.34), and East Asia (EAPC = 0.89). In contrast, the high-income Asia Pacific (EAPC = −6.37), the Caribbean (EAPC = −4.63), and Western Europe (EAPC = −3.85) experienced the most significant decreases (Table 1; Figures 3B, 4B).

Among the 21 regions examined, the age-standardized DALY rate for youth and young adults aged 15–39 years, globally in 2021, was highest in East Asia (82.13), the Caribbean (67.55), and South Asia (55.35). The lowest rates were observed in Oceania (10.17), North Africa and the Middle East (15.40), and Australasia (18.21). From 1990 to 2021, the age-standardized DALY rate for this age group worldwide displayed the highest increases in Andean Latin America (EAPC = 1.43), East Asia (EAPC = 0.28), and Tropical Latin America (EAPC = 0.27). In contrast, the largest decreases were noted in high-income Asia Pacific (EAPC = −5.20), the Caribbean (EAPC = −4.23), and Australasia (EAPC = −3.30; Table 1; Figures 3C, 4C).

### 3.5 Trends in CRI burden among youth and young adults aged 15–39 in different country regions

In 2021, among 204 countries, the ASIR of CRI for youth and young adults aged 15–39 years was highest in Brunei Darussalam (448.61), Andorra (379.30), and Cuba (356.56). Conversely, the lowest ASIRs were observed in Mali (34.85), Gambia (36.83), and Ethiopia (36.97). Globally, between 1990 and 2021, the most significant increases in the ASIR of youth and young adult CRI aged 15–39 years were noted in Ecuador (EAPC = 1.61), Armenia (EAPC = 1.25), and Oman (EAPC = 1.15). In contrast, the largest decreases were in Croatia (EAPC = −4.49), the Republic of Korea (EAPC = −4.23), and New Zealand (EAPC = −3.99; Supplementary Table S5; Figures 3A, 4A).

In 2021, among the 204 examined countries, the ASDR for youth and young adults aged 15–39 with CRI was found to be highest in Belize (1.40), Guyana (1.39), and Zambia (1.23). Conversely, the lowest ASDR for the same age group with CRI was observed in Palau (0.01), Morocco (0.02), and Palestine (0.02). Globally, from 1990 to 2021, the ASDR for CRI among youth and young adults aged 15–39 years was highest in Taiwan (Province of China) and Palestine, both recording an ASDR of 0.02. During the same period, the regions with the most significant increases in ASDR were Taiwan (Province of China; EAPC = 16.39), Mauritius (EAPC = 15.04), and Zimbabwe (EAPC = 9.16). Conversely, the largest decreases were observed in the Republic of Korea (EAPC = −9.02), Bermuda (EAPC = −8.16), and Croatia (EAPC = −7.18). These findings are detailed in Supplementary Table S6 and Figures 3B, 4B.

In 2021, among the 204 countries analyzed, the age-standardized DALY rate for CRI among youth and young adults aged 15–39 years was highest in Belize (107.99), Guyana (106.18), and Haiti (96.50), while the lowest rates were observed in Palestine (5.95), Palau (7.04), and Tonga (7.07). Globally, from 1990 to 2021, the age-standardized DALY rate trends for youth and young adult CRI showed the most significant increases in Zimbabwe (EAPC = 6.50), Mauritius (EAPC = 6.01), and Taiwan (Province of China; EAPC = 5.11). In contrast, the largest decreases were recorded in the Republic of Korea (EAPC = −7.00), Croatia (EAPC = −6.99), and Bermuda (EAPC = −6.19). These trends are further detailed in Supplementary Table S7 and Figures 3C, 4C.

## 4 Discussion

Globally, the ASIR, ASDR, and age-standardized DALY rates for youth and young adults aged 15–39 with CRI showed a decline from 1990 to 2021. There was an increase in both the number of deaths and the absolute number of DALYs. In all five SDI regions, ASIR, ASDR and the age-standardized DALY rate exhibited a decreasing trend, with the highest rates in the high-middle SDI and the lowest in the low-middle SDI, while ASDR with the highest rates in the middle SDI. Among the 21 regions studied, the ASDR for those aged 15–39 was the highest, while the ASDR for youth and young adult CRI was the lowest. Notably, both ASDR and the age-standardized DALY rate for youth and young adult CRI were highest in East Asia. Furthermore, between 1990 and 2021, the global burden of youth and young adult CRI for ages 15–39 increased most significantly in Andean Latin America and decreased most notably in high-income Asia Pacific.

The observed decline in CRI among youth and young adults aged 15–39 appears paradoxical given the simultaneous increase in cycling fatalities. While improved roadway infrastructure, dedicated bicycle facilities, and enhanced traffic management systems (19–22) have likely contributed to reduced crash rates, the rising fatality trend may reflect underaddressed risk factors. This discrepancy could stem from: (1) increased severity of remaining crashs due to higher vehicle speeds associated with modernized road networks (23), (2) potential time-lags in emergency response system adaptations to new infrastructure configurations, and (3) shifting demographics of cyclists exposed to risk.

Notably, this study’s injury/fatality rates are calculated without exposure metrics (e.g., kilometers cycled or modal shift patterns), limiting direct comparisons across populations. For instance, increased cycling uptake in urban areas - while beneficial for sustainability - might paradoxically elevate absolute fatality numbers if not matched with proportional safety improvements (24). Future research incorporating exposure data and modal shift analysis would better elucidate these trends. The growth in DALYs remains primarily attributable to population aging and expanded demographic bases (22).

The high burden of CRI in high-middle and middle SDI regions is likely driven by systemic challenges, including inadequate transportation infrastructure, rapid motorization, and weak policy enforcement (23, 24). These regions often prioritize motor vehicle-centric road design during urbanization, leaving cyclists to share mixed-traffic environments without dedicated lanes or segregated infrastructure (25). Emerging mobility trends, such as the proliferation of high-speed bikes (which require distinct regulatory frameworks), further compound safety risks in these settings. Structural deficiencies—including poorly designed intersections, limited traffic calming measures, and high-density traffic flows—amplify collision risks. While population-level trends suggest associations with risky behaviors (e.g., non-compliance with traffic rules), this study’s ecological design precludes direct attribution to individual-level factors. Instead, the findings emphasize systemic contributors, such as insufficient traffic management systems and gaps in cyclist safety education. Addressing these challenges will require multifaceted interventions, including infrastructure upgrades, adaptive regulations for emerging vehicle types, and data-driven traffic management to reduce conflicts between cyclists and motorized traffic.

Among the 21 regions analyzed, East Asia demonstrated the highest ASDR and DALY rate for CRI among youth and young adults aged 15–39. This regional burden aligns with rapid urbanization patterns, growing adoption of micromobility devices (e.g., shared bicycles), and persistent gaps in infrastructure development across East Asian countries. While motor vehicle population growth and delayed bicycle infrastructure modernization contribute to collision risks, regional variations in regulatory frameworks and road safety culture likely mediate these associations (26, 27). These findings underscore the need for region-specific interventions that account for local transportation modalities and urbanization trajectories.

High-income Asia Pacific regions, such as Japan, South Korea, and Singapore, have achieved significant reductions in cycling injury burdens through multifaceted strategies. These include investments in physically segregated bicycle lanes, dedicated cycling signals, and intersection designs that minimize conflicts with motor vehicles (28). Strict enforcement of helmet mandates, red-light compliance, and penalties for risky cycling behaviors have further reduced injury risks (29). Additionally, public education campaigns and smart traffic management systems (e.g., real-time cyclist detection) have enhanced safety outcomes (30). In contrast, low- and middle-income regions, including parts of Latin America, North Africa, and South Asia, face persistent challenges. Studies highlight gaps in infrastructure quality, inconsistent enforcement of traffic laws, and limited safety education in these areas (31). For instance, shared road spaces without cyclist prioritization and inadequate lighting increase collision risks (29). To address these disparities, context-specific adaptations of proven interventions—such as phased infrastructure upgrades, community-led education programs, and data-driven enforcement—are critical to reducing cycling injury burdens in resource-limited settings.

Consistent with prior studies, death rates from CRI rise with age, likely reflecting higher cumulative exposure to cycling among older age groups (e.g., individuals in their 30s) rather than declines in physical fitness (32). Notably, a sharp increase in mortality is observed among adolescents aged 15–19, a group for which road injuries rank as the second-leading cause of death in China, surpassed only by cancer (33). Adolescents face heightened vulnerability due to developmental factors—including impulsivity, reduced risk perception, and diminishing parental supervision—coupled with risky behaviors such as helmet non-use and smartphone distractions while cycling (34). Gender disparities further amplify risks: males exhibit higher cycling participation rates and engagement in high-risk behaviors (e.g., helmet avoidance, reckless riding), which align with their elevated injury and fatality rates (35). These findings underscore the need for targeted interventions, such as stricter helmet mandates, safer infrastructure design (36), and youth-focused education programs addressing peer-influenced risk-taking (37).

While this study provides critical insights, several limitations must be acknowledged. First, the analysis is susceptible to ecological fallacy due to its reliance on aggregated population-level data, which may obscure individual-level risk factors (e.g., actual cycling exposure, behavioral patterns) and lead to misinterpretation of associations. Second, the use of population-based denominators (e.g., regional population size) rather than exposure-based metrics (e.g., cycling frequency or kilometers traveled) limits accurate risk estimation, potentially masking disparities in injury rates among active cyclists versus non-cyclists. Additionally, inconsistent data quality across regions—particularly between urban and rural areas—and insufficient consideration of cultural, socioeconomic, and emerging trends (e.g., bike-sharing adoption, shifting youth behaviors) further constrain the granularity of findings. The study also lacks robust assessment of confounders and intervention-specific impacts. Future research should prioritize exposure-adjusted denominators, integrate dynamic behavioral data, and employ mixed-methods approaches to mitigate these limitations and strengthen policy-relevant evidence.

## 5 Conclusion

This study reveals a nuanced global trend in CRI burden among youth and young adults (15–39 years), showing an overall decline from 1990 to 2021 despite rising absolute deaths and DALYs in specific regions. While high-middle and middle SDI regions (e.g., East Asia, the Caribbean) bear disproportionate burdens, the Andean Latin America region exhibits the sharpest increase, contrasting with significant declines in high-income Asia Pacific. These findings highlight critical geographic and developmental disparities, underscoring the need for tailored interventions. Unlike prior reviews, this analysis emphasizes the compounding effects of population growth and evolving transportation systems, advocating for region-specific strategies: stricter enforcement of road safety laws in high-burden areas, infrastructure upgrades in rapidly urbanizing regions, and targeted education campaigns to mitigate emerging risks in transitioning economies.

## Data Availability

The original contributions presented in the study are included in the article/Supplementary material, further inquiries can be directed to the corresponding author.
