# Supplementary material for: Global burden of disease from cyclist road injuries in youth and young adults aged 15–39 years, 1990–2021
Source: Front Public Health. 2025 May 12;13:1581789. doi: 10.3389/fpubh.2025.1581789 (PMC12104176; doi:10.3389/fpubh.2025.1581789)
Supplement: Supplementary file 1 [file Data_Sheet_1.docx]

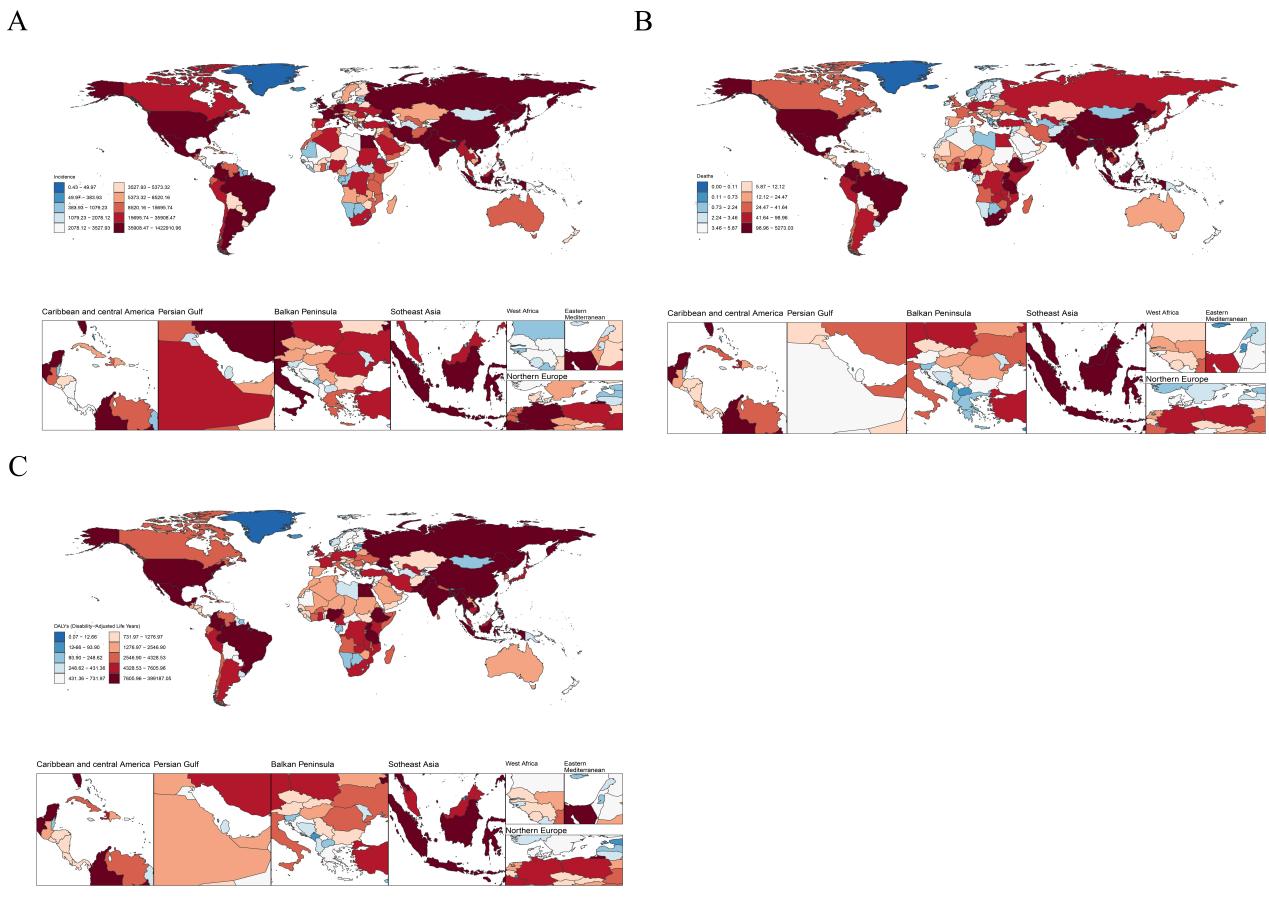


Figure S1 The global disease burden of cyclist road injuries in 204 countries and territories in 2021.

DALY = disability adjusted life-year.

(A) incidence number (B) deaths number (C) DALYs number
