# Supplementary material for: Global burden of disease from cyclist road injuries in youth and young adults aged 15–39 years, 1990–2021
Source: Front Public Health. 2025 May 12;13:1581789. doi: 10.3389/fpubh.2025.1581789 (PMC12104176; doi:10.3389/fpubh.2025.1581789)
Supplement: Supplementary file 2 [file Data_Sheet_2.docx]

Table S1 Cases number of incidence, deaths and DALYs of cyclist road injuries aged 15-39 in 204 countries, 1990 to 2021

|  |  | incidence | incidence | DALYs (Disability-Adjusted Life Years) | DALYs (Disability-Adjusted Life Years) | deaths | deaths |  |  |  |
| --- | --- | --- | --- | --- | --- | --- | --- | --- | --- | --- |
|  |  | 1990 | 2021 | 1990 | 2021 | 1990 | 2021 |  |  |  |
| Country | sex | nIncidence_90 | nIncidence_21 | nDALY_90 | nDALY_21 | nDeaths_90 | nDeaths_21 | time_Incidence | time_daly | time_deaths |
| global | both | 4995943.51 | 4748268.68 | 1237227.37 | 1375633.34 | 13765.47 | 17431.88 | 0.95 | 1.11 | 1.27 |
| High-middle SDI | both | 1405217.02 | 1133407.46 | 280357.79 | 246533.60 | 2880.04 | 2963.81 | 0.81 | 0.88 | 1.03 |
| High SDI | both | 1470921.29 | 880480.26 | 218994.85 | 113072.81 | 2048.81 | 1056.71 | 0.60 | 0.52 | 0.52 |
| Low-middle SDI | both | 521846.90 | 912395.80 | 228960.32 | 375799.88 | 2871.63 | 5007.32 | 1.75 | 1.64 | 1.74 |
| Low SDI | both | 139993.35 | 304157.07 | 76528.55 | 149297.74 | 964.98 | 1944.26 | 2.17 | 1.95 | 2.01 |
| Middle SDI | both | 1451975.93 | 1514055.34 | 430451.85 | 489941.99 | 4976.87 | 6448.33 | 1.04 | 1.14 | 1.30 |
| Andean Latin America | both | 16906.88 | 33912.35 | 4825.23 | 10367.98 | 52.99 | 133.24 | 2.01 | 2.15 | 2.51 |
| Australasia | both | 25931.67 | 18410.19 | 4008.87 | 1919.68 | 39.63 | 15.73 | 0.71 | 0.48 | 0.40 |
| Caribbean | both | 50668.65 | 34252.15 | 30979.69 | 12293.34 | 423.70 | 153.21 | 0.68 | 0.40 | 0.36 |
| Central Asia | both | 28465.76 | 33376.96 | 6648.70 | 8130.86 | 67.57 | 88.19 | 1.17 | 1.22 | 1.31 |
| Central Europe | both | 192175.88 | 95976.80 | 39372.14 | 17355.30 | 385.25 | 175.34 | 0.50 | 0.44 | 0.46 |
| Central Latin America | both | 137599.14 | 163219.34 | 37759.38 | 40456.74 | 410.53 | 466.87 | 1.19 | 1.07 | 1.14 |
| Central sub-Saharan Africa | both | 12152.73 | 27854.08 | 4772.19 | 11468.49 | 51.35 | 136.02 | 2.29 | 2.40 | 2.65 |
| East Asia | both | 1602453.00 | 1450016.13 | 393201.15 | 407085.14 | 4331.48 | 5375.69 | 0.90 | 1.04 | 1.24 |
| Eastern Europe | both | 234600.84 | 130024.93 | 42714.49 | 18778.96 | 387.45 | 154.21 | 0.55 | 0.44 | 0.40 |
| Eastern Sub-Saharan Africa | both | 47566.36 | 101683.64 | 34003.04 | 69989.37 | 442.55 | 946.92 | 2.14 | 2.06 | 2.14 |
| High-income Asia Pacific | both | 388030.87 | 109887.16 | 56713.94 | 10951.78 | 570.78 | 79.20 | 0.28 | 0.19 | 0.14 |
| High-income North America | both | 439793.38 | 337941.91 | 61515.44 | 35798.35 | 521.53 | 311.41 | 0.77 | 0.58 | 0.60 |
| North Africa and Middle East | both | 160199.94 | 272098.58 | 24080.18 | 39517.59 | 134.38 | 301.96 | 1.70 | 1.64 | 2.25 |
| Oceania | both | 1950.66 | 3442.47 | 306.43 | 565.91 | 2.33 | 4.58 | 1.76 | 1.85 | 1.97 |
| South Asia | both | 485793.95 | 941360.37 | 264149.41 | 436161.85 | 3526.23 | 6036.57 | 1.94 | 1.65 | 1.71 |
| Southeast Asia | both | 283084.82 | 301134.13 | 88557.88 | 118477.61 | 1012.35 | 1566.47 | 1.06 | 1.34 | 1.55 |
| Southern Latin America | both | 60927.79 | 83065.18 | 8486.89 | 8946.32 | 73.65 | 72.90 | 1.36 | 1.05 | 0.99 |
| Southern sub-Saharan Africa | both | 19419.39 | 25365.57 | 6613.76 | 10617.78 | 83.74 | 144.21 | 1.31 | 1.61 | 1.72 |
| Tropical Latin America | both | 129591.68 | 170451.97 | 29543.50 | 42723.18 | 308.68 | 506.66 | 1.32 | 1.45 | 1.64 |
| Western Europe | both | 645361.04 | 328085.51 | 82581.32 | 32590.66 | 744.24 | 233.46 | 0.51 | 0.39 | 0.31 |
| Western sub-Saharan Africa | both | 33269.10 | 86709.26 | 16393.74 | 41436.44 | 195.05 | 529.05 | 2.61 | 2.53 | 2.71 |
| global | female | 1637730.71 | 1408155.75 | 316898.00 | 268241.62 | 2903.17 | 2726.78 | 0.86 | 0.85 | 0.94 |
| High-middle SDI | female | 498376.49 | 374852.60 | 89227.34 | 66223.51 | 809.64 | 696.90 | 0.75 | 0.74 | 0.86 |
| High SDI | female | 414218.77 | 261188.65 | 56380.02 | 29753.67 | 448.81 | 228.74 | 0.63 | 0.53 | 0.51 |
| Low-middle SDI | female | 163795.06 | 226863.88 | 42344.12 | 50540.90 | 395.22 | 493.06 | 1.39 | 1.19 | 1.25 |
| Low SDI | female | 46313.28 | 88194.58 | 12636.48 | 23571.11 | 111.03 | 224.58 | 1.90 | 1.87 | 2.02 |
| Middle SDI | female | 513181.50 | 455906.88 | 115933.83 | 97934.62 | 1135.18 | 1081.56 | 0.89 | 0.84 | 0.95 |
| Andean Latin America | female | 4763.29 | 8326.31 | 998.49 | 1692.69 | 7.16 | 15.95 | 1.75 | 1.70 | 2.23 |
| Australasia | female | 6295.95 | 4924.82 | 802.71 | 423.54 | 6.64 | 2.32 | 0.78 | 0.53 | 0.35 |
| Caribbean | female | 13298.49 | 9628.55 | 4720.38 | 2557.78 | 55.26 | 25.78 | 0.72 | 0.54 | 0.47 |
| Central Asia | female | 10093.23 | 11722.93 | 1641.12 | 1590.60 | 10.17 | 8.33 | 1.16 | 0.97 | 0.82 |
| Central Europe | female | 61725.23 | 28736.34 | 9025.39 | 4197.46 | 49.95 | 30.28 | 0.47 | 0.47 | 0.61 |
| Central Latin America | female | 23670.04 | 31680.86 | 5177.72 | 6009.52 | 34.89 | 48.13 | 1.34 | 1.16 | 1.38 |
| Central sub-Saharan Africa | female | 4679.12 | 9737.63 | 719.19 | 1497.13 | 2.03 | 6.40 | 2.08 | 2.08 | 3.15 |
| East Asia | female | 647672.96 | 509703.73 | 146116.02 | 115897.38 | 1535.77 | 1416.06 | 0.79 | 0.79 | 0.92 |
| Eastern Europe | female | 74091.66 | 46185.63 | 8926.32 | 4953.30 | 32.81 | 20.05 | 0.62 | 0.55 | 0.61 |
| Eastern Sub-Saharan Africa | female | 16501.36 | 29984.27 | 6879.39 | 13302.69 | 76.32 | 156.68 | 1.82 | 1.93 | 2.05 |
| High-income Asia Pacific | female | 106498.40 | 32382.02 | 15567.98 | 3666.52 | 142.85 | 26.06 | 0.30 | 0.24 | 0.18 |
| High-income North America | female | 113597.82 | 101438.43 | 12190.42 | 8313.18 | 70.21 | 49.45 | 0.89 | 0.68 | 0.70 |
| North Africa and Middle East | female | 54045.68 | 87168.07 | 6284.82 | 8093.73 | 11.31 | 17.68 | 1.61 | 1.29 | 1.56 |
| Oceania | female | 908.46 | 1564.49 | 121.00 | 215.21 | 0.77 | 1.43 | 1.72 | 1.78 | 1.86 |
| South Asia | female | 135520.26 | 201490.83 | 35240.51 | 42908.98 | 332.72 | 408.67 | 1.49 | 1.22 | 1.23 |
| Southeast Asia | female | 108551.10 | 105757.43 | 24956.56 | 23863.08 | 234.00 | 257.34 | 0.97 | 0.96 | 1.10 |
| Southern Latin America | female | 19348.14 | 25999.07 | 2787.80 | 2657.94 | 21.76 | 16.57 | 1.34 | 0.95 | 0.76 |
| Southern sub-Saharan Africa | female | 7124.45 | 8069.97 | 1169.62 | 1532.73 | 7.48 | 12.95 | 1.13 | 1.31 | 1.73 |
| Tropical Latin America | female | 36038.52 | 36325.12 | 6338.32 | 8398.92 | 47.09 | 87.67 | 1.01 | 1.33 | 1.86 |
| Western Europe | female | 179949.44 | 86907.48 | 23517.27 | 8763.20 | 193.58 | 51.53 | 0.48 | 0.37 | 0.27 |
| Western sub-Saharan Africa | female | 13357.10 | 30421.77 | 3716.97 | 7706.04 | 30.42 | 67.44 | 2.28 | 2.07 | 2.22 |
| global | male | 3358212.80 | 3340112.93 | 920329.36 | 1107391.73 | 10862.30 | 14705.11 | 0.99 | 1.20 | 1.35 |
| High-middle SDI | male | 906840.53 | 758554.86 | 191130.45 | 180310.09 | 2070.40 | 2266.91 | 0.84 | 0.94 | 1.09 |
| High SDI | male | 1056702.53 | 619291.60 | 162614.82 | 83319.15 | 1599.99 | 827.97 | 0.59 | 0.51 | 0.52 |
| Low-middle SDI | male | 358051.84 | 685531.92 | 186616.20 | 325258.98 | 2476.40 | 4514.26 | 1.91 | 1.74 | 1.82 |
| Low SDI | male | 93680.07 | 215962.49 | 63892.08 | 125726.63 | 853.96 | 1719.68 | 2.31 | 1.97 | 2.01 |
| Middle SDI | male | 938794.43 | 1058148.46 | 314518.02 | 392007.37 | 3841.69 | 5366.77 | 1.13 | 1.25 | 1.40 |
| Andean Latin America | male | 12143.58 | 25586.04 | 3826.75 | 8675.28 | 45.84 | 117.29 | 2.11 | 2.27 | 2.56 |
| Australasia | male | 19635.72 | 13485.37 | 3206.16 | 1496.14 | 32.99 | 13.41 | 0.69 | 0.47 | 0.41 |
| Caribbean | male | 37370.16 | 24623.60 | 26259.30 | 9735.56 | 368.44 | 127.43 | 0.66 | 0.37 | 0.35 |
| Central Asia | male | 18372.52 | 21654.03 | 5007.58 | 6540.26 | 57.40 | 79.86 | 1.18 | 1.31 | 1.39 |
| Central Europe | male | 130450.65 | 67240.47 | 30346.75 | 13157.84 | 335.30 | 145.06 | 0.52 | 0.43 | 0.43 |
| Central Latin America | male | 113929.10 | 131538.48 | 32581.66 | 34447.22 | 375.65 | 418.74 | 1.15 | 1.06 | 1.11 |
| Central sub-Saharan Africa | male | 7473.61 | 18116.45 | 4052.99 | 9971.36 | 49.32 | 129.62 | 2.42 | 2.46 | 2.63 |
| East Asia | male | 954780.04 | 940312.40 | 247085.13 | 291187.75 | 2795.71 | 3959.62 | 0.98 | 1.18 | 1.42 |
| Eastern Europe | male | 160509.19 | 83839.30 | 33788.16 | 13825.66 | 354.65 | 134.16 | 0.52 | 0.41 | 0.38 |
| Eastern Sub-Saharan Africa | male | 31065.00 | 71699.37 | 27123.65 | 56686.68 | 366.23 | 790.23 | 2.31 | 2.09 | 2.16 |
| High-income Asia Pacific | male | 281532.47 | 77505.14 | 41145.96 | 7285.26 | 427.93 | 53.14 | 0.28 | 0.18 | 0.12 |
| High-income North America | male | 326195.56 | 236503.48 | 49325.02 | 27485.17 | 451.33 | 261.96 | 0.73 | 0.56 | 0.58 |
| North Africa and Middle East | male | 106154.26 | 184930.51 | 17795.36 | 31423.87 | 123.07 | 284.28 | 1.74 | 1.77 | 2.31 |
| Oceania | male | 1042.19 | 1877.98 | 185.43 | 350.70 | 1.56 | 3.16 | 1.80 | 1.89 | 2.02 |
| South Asia | male | 350273.69 | 739869.54 | 228908.91 | 393252.87 | 3193.52 | 5627.90 | 2.11 | 1.72 | 1.76 |
| Southeast Asia | male | 174533.72 | 195376.70 | 63601.32 | 94614.53 | 778.35 | 1309.13 | 1.12 | 1.49 | 1.68 |
| Southern Latin America | male | 41579.66 | 57066.11 | 5699.10 | 6288.38 | 51.89 | 56.33 | 1.37 | 1.10 | 1.09 |
| Southern sub-Saharan Africa | male | 12294.94 | 17295.60 | 5444.13 | 9085.05 | 76.25 | 131.26 | 1.41 | 1.67 | 1.72 |
| Tropical Latin America | male | 93553.16 | 134126.85 | 23205.18 | 34324.26 | 261.60 | 418.99 | 1.43 | 1.48 | 1.60 |
| Western Europe | male | 465411.60 | 241178.03 | 59064.05 | 23827.46 | 550.66 | 181.93 | 0.52 | 0.40 | 0.33 |
| Western sub-Saharan Africa | male | 19911.99 | 56287.49 | 12676.78 | 33730.40 | 164.63 | 461.61 | 2.83 | 2.66 | 2.80 |

Table S2 Age specific incidence rate of cyclist road injuries aged 15-39 in 204 countries, 2021

| incidence | 15-19 years | 20-24 years | 25-29 years | 30-34 years | 35-39 years |
| --- | --- | --- | --- | --- | --- |
| Afghanistan | 95.31 | 87.65 | 80.44 | 74.95 | 67.55 |
| Albania | 306.17 | 316.46 | 258.01 | 207.21 | 169.07 |
| Algeria | 136.77 | 119.28 | 105.51 | 96.44 | 86.05 |
| American Samoa | 72.44 | 83.72 | 73.49 | 65.63 | 58.26 |
| Andorra | 498.14 | 459.99 | 353.24 | 296.90 | 258.76 |
| Angola | 74.01 | 60.97 | 52.88 | 48.33 | 45.59 |
| Antigua and Barbuda | 135.05 | 148.10 | 114.82 | 87.53 | 74.60 |
| Argentina | 437.62 | 383.41 | 290.92 | 240.29 | 206.18 |
| Armenia | 112.23 | 118.55 | 95.09 | 78.94 | 74.83 |
| Australia | 239.38 | 207.39 | 154.98 | 124.79 | 111.83 |
| Austria | 405.42 | 318.09 | 204.13 | 175.30 | 168.08 |
| Azerbaijan | 93.23 | 85.78 | 68.03 | 58.60 | 53.91 |
| Bahrain | 202.31 | 217.94 | 181.32 | 151.21 | 123.18 |
| Bangladesh | 131.82 | 126.42 | 106.16 | 88.90 | 77.37 |
| Barbados | 74.09 | 66.54 | 59.10 | 49.20 | 44.74 |
| Belarus | 165.59 | 161.58 | 135.70 | 117.61 | 93.36 |
| Belgium | 228.11 | 229.13 | 183.50 | 158.18 | 153.97 |
| Belize | 455.65 | 376.06 | 264.07 | 230.26 | 209.25 |
| Benin | 340.75 | 406.32 | 356.03 | 293.30 | 223.76 |
| Bermuda | 43.31 | 39.77 | 37.83 | 36.15 | 36.32 |
| Bhutan | 169.16 | 178.30 | 143.37 | 118.27 | 105.90 |
| Bolivia (Plurinational State of) | 84.66 | 86.47 | 72.75 | 65.24 | 61.75 |
| Bosnia and Herzegovina | 94.47 | 93.86 | 85.83 | 77.98 | 71.66 |
| Botswana | 272.67 | 282.02 | 209.36 | 161.40 | 136.21 |
| Brazil | 77.10 | 67.47 | 59.26 | 55.24 | 52.15 |
| Brunei Darussalam | 253.83 | 219.86 | 186.31 | 166.62 | 152.47 |
| Bulgaria | 537.44 | 562.03 | 449.89 | 354.06 | 309.34 |
| Burkina Faso | 407.11 | 377.67 | 281.59 | 228.07 | 188.66 |
| Burundi | 62.91 | 56.29 | 53.20 | 52.66 | 53.81 |
| Cambodia | 81.93 | 69.74 | 58.62 | 54.38 | 60.68 |
| Cameroon | 42.71 | 43.27 | 39.80 | 40.88 | 40.34 |
| Canada | 149.60 | 135.66 | 105.99 | 97.57 | 88.61 |
| Cabo Verde | 60.85 | 57.47 | 57.45 | 57.49 | 57.54 |
| Central African Republic | 303.62 | 255.76 | 184.43 | 148.47 | 132.12 |
| Chad | 66.29 | 50.08 | 42.33 | 40.52 | 41.17 |
| Chile | 47.57 | 42.83 | 39.58 | 36.62 | 36.27 |
| China | 398.24 | 431.71 | 370.18 | 289.04 | 228.33 |
| Colombia | 265.74 | 320.32 | 304.64 | 310.20 | 317.77 |
| Comoros | 233.78 | 221.19 | 177.61 | 146.31 | 130.44 |
| Congo | 69.69 | 59.87 | 50.80 | 46.17 | 48.04 |
| Costa Rica | 57.86 | 48.81 | 42.72 | 38.95 | 36.88 |
| Côte d'Ivoire | 94.08 | 126.75 | 126.94 | 121.73 | 105.84 |
| Croatia | 191.53 | 173.51 | 149.45 | 140.80 | 127.39 |
| Cuba | 397.19 | 359.80 | 268.55 | 217.85 | 183.02 |
| Cyprus | 179.74 | 209.96 | 188.16 | 164.15 | 150.24 |
| Czechia | 531.55 | 439.29 | 313.07 | 247.92 | 212.79 |
| Democratic Republic of the Congo | 348.88 | 347.71 | 251.07 | 193.01 | 169.03 |
| Denmark | 52.23 | 48.09 | 43.89 | 41.38 | 42.15 |
| Djibouti | 282.26 | 314.26 | 276.07 | 269.71 | 278.57 |
| Dominica | 61.21 | 50.75 | 44.48 | 41.07 | 38.74 |
| Dominican Republic | 394.37 | 320.26 | 212.78 | 178.92 | 156.36 |
| Ecuador | 66.96 | 56.53 | 48.96 | 45.22 | 48.10 |
| Egypt | 240.14 | 228.31 | 189.39 | 159.31 | 136.67 |
| El Salvador | 220.42 | 219.55 | 186.05 | 160.29 | 139.37 |
| Equatorial Guinea | 176.23 | 205.39 | 193.03 | 170.22 | 142.25 |
| Eritrea | 131.72 | 112.56 | 101.03 | 96.43 | 88.37 |
| Estonia | 147.71 | 128.61 | 108.84 | 100.52 | 90.94 |
| Ethiopia | 71.57 | 60.22 | 51.28 | 46.24 | 43.05 |
| Micronesia (Federated States of) | 74.50 | 68.73 | 61.14 | 58.68 | 66.60 |
| Fiji | 179.21 | 173.21 | 141.17 | 122.40 | 104.71 |
| Finland | 77.17 | 73.34 | 71.01 | 71.79 | 71.30 |
| France | 42.35 | 39.48 | 35.04 | 33.18 | 33.75 |
| Gabon | 70.36 | 76.28 | 64.58 | 55.72 | 49.39 |
| Georgia | 359.08 | 281.45 | 188.87 | 158.34 | 139.68 |
| Germany | 435.12 | 386.97 | 283.34 | 232.86 | 201.62 |
| Ghana | 71.01 | 59.35 | 50.11 | 47.22 | 45.95 |
| Greece | 42.00 | 39.17 | 35.12 | 32.94 | 33.92 |
| Greenland | 169.56 | 121.69 | 85.31 | 71.62 | 62.30 |
| Grenada | 393.23 | 330.45 | 224.10 | 180.15 | 159.25 |
| Guam | 81.12 | 73.71 | 64.49 | 61.55 | 65.37 |
| Guatemala | 503.64 | 403.81 | 292.28 | 247.09 | 223.20 |
| Guinea | 198.89 | 170.21 | 124.57 | 99.37 | 86.29 |
| Guinea-Bissau | 161.19 | 170.31 | 138.15 | 126.96 | 107.33 |
| Guyana | 88.16 | 92.91 | 78.12 | 74.47 | 68.16 |
| Haiti | 165.37 | 150.23 | 130.94 | 121.39 | 110.14 |
| Honduras | 54.02 | 48.71 | 45.05 | 41.76 | 40.70 |
| Hungary | 73.33 | 70.40 | 65.59 | 61.96 | 62.18 |
| Iceland | 291.38 | 340.46 | 292.15 | 243.75 | 202.52 |
| India | 262.67 | 231.88 | 201.00 | 175.67 | 159.53 |
| Indonesia | 143.52 | 124.45 | 103.75 | 101.35 | 102.01 |
| Iran (Islamic Republic of) | 281.02 | 291.08 | 233.22 | 194.86 | 175.51 |
| Iraq | 282.66 | 250.68 | 174.89 | 141.24 | 129.33 |
| Ireland | 112.57 | 135.79 | 137.12 | 136.63 | 135.28 |
| Israel | 137.02 | 119.26 | 89.45 | 76.25 | 69.77 |
| Italy | 212.95 | 196.18 | 164.37 | 139.91 | 118.00 |
| Jamaica | 101.06 | 94.15 | 83.27 | 77.07 | 70.15 |
| Japan | 313.84 | 270.89 | 195.73 | 153.23 | 131.58 |
| Jordan | 345.36 | 291.80 | 217.11 | 182.85 | 160.86 |
| Kazakhstan | 441.15 | 339.90 | 250.73 | 220.61 | 205.18 |
| Kenya | 107.88 | 122.46 | 103.41 | 87.03 | 76.55 |
| Kiribati | 280.32 | 271.21 | 193.61 | 150.43 | 132.45 |
| Kuwait | 94.02 | 88.84 | 79.09 | 72.20 | 64.43 |
| Kyrgyzstan | 134.06 | 119.82 | 98.07 | 86.82 | 77.34 |
| Lao People's Democratic Republic | 52.96 | 50.83 | 47.07 | 48.15 | 53.00 |
| Latvia | 74.06 | 91.06 | 86.38 | 79.11 | 66.51 |
| Lebanon | 127.01 | 108.38 | 89.77 | 79.33 | 71.59 |
| Lesotho | 101.97 | 97.41 | 82.88 | 75.54 | 69.72 |
| Liberia | 139.81 | 128.75 | 108.03 | 99.68 | 90.17 |
| Libya | 245.45 | 239.15 | 194.63 | 178.41 | 172.01 |
| Lithuania | 113.18 | 107.57 | 90.01 | 78.52 | 67.35 |
| Luxembourg | 64.19 | 60.99 | 59.79 | 63.52 | 64.69 |
| North Macedonia | 41.18 | 39.45 | 36.34 | 34.23 | 33.86 |
| Madagascar | 119.51 | 106.74 | 97.94 | 96.82 | 88.67 |
| Malawi | 244.05 | 229.90 | 196.73 | 179.89 | 164.99 |
| Malaysia | 418.15 | 353.42 | 247.71 | 207.08 | 176.35 |
| Maldives | 63.91 | 58.72 | 50.80 | 47.15 | 49.91 |
| Mali | 67.80 | 58.10 | 48.70 | 45.23 | 47.81 |
| Malta | 174.49 | 159.08 | 127.86 | 120.50 | 104.68 |
| Marshall Islands | 74.30 | 77.48 | 65.16 | 53.14 | 42.86 |
| Mauritania | 39.05 | 37.21 | 34.13 | 31.73 | 31.13 |
| Mauritius | 353.04 | 305.95 | 215.27 | 169.57 | 147.18 |
| Mexico | 60.64 | 68.19 | 63.37 | 58.41 | 53.17 |
| Republic of Moldova | 63.41 | 58.11 | 55.39 | 53.64 | 51.80 |
| Mongolia | 97.26 | 94.08 | 83.16 | 79.02 | 67.44 |
| Montenegro | 195.51 | 189.51 | 164.61 | 145.49 | 130.15 |
| Morocco | 81.94 | 96.93 | 92.99 | 81.65 | 69.67 |
| Mozambique | 412.37 | 364.53 | 261.69 | 215.01 | 182.99 |
| Myanmar | 117.17 | 109.85 | 91.30 | 79.51 | 70.22 |
| Namibia | 333.80 | 356.40 | 273.41 | 213.25 | 186.34 |
| Nepal | 120.61 | 104.84 | 89.35 | 81.86 | 75.53 |
| Netherlands | 91.45 | 78.65 | 66.14 | 62.19 | 67.33 |
| New Zealand | 122.23 | 111.85 | 90.49 | 80.70 | 72.38 |
| Nicaragua | 70.12 | 64.95 | 60.86 | 60.26 | 58.33 |
| Niger | 113.95 | 141.43 | 137.14 | 130.76 | 115.24 |
| Nigeria | 72.70 | 74.70 | 64.38 | 58.70 | 53.37 |
| Democratic People's Republic of Korea | 387.40 | 302.90 | 197.11 | 161.36 | 139.84 |
| Northern Mariana Islands | 333.39 | 289.17 | 222.79 | 181.83 | 166.06 |
| Norway | 157.96 | 132.47 | 97.56 | 81.94 | 75.77 |
| Oman | 48.19 | 43.83 | 40.54 | 37.73 | 37.20 |
| Pakistan | 41.75 | 40.66 | 38.95 | 38.16 | 38.03 |
| Palestine | 83.57 | 87.95 | 76.27 | 67.88 | 61.02 |
| Panama | 274.71 | 271.27 | 207.08 | 166.49 | 147.15 |
| Papua New Guinea | 84.31 | 88.56 | 80.25 | 75.20 | 69.68 |
| Paraguay | 253.26 | 247.41 | 187.39 | 161.04 | 141.74 |
| Peru | 297.20 | 228.20 | 154.64 | 133.87 | 119.59 |
| Philippines | 96.38 | 102.02 | 95.61 | 87.44 | 82.11 |
| Poland | 91.94 | 116.20 | 116.09 | 109.52 | 93.98 |
| Portugal | 78.30 | 74.07 | 64.58 | 57.57 | 49.10 |
| Puerto Rico | 203.32 | 188.54 | 155.61 | 138.91 | 122.15 |
| Qatar | 63.55 | 62.17 | 55.45 | 51.04 | 49.04 |
| Romania | 196.80 | 168.87 | 145.02 | 124.31 | 107.70 |
| Russian Federation | 107.34 | 124.89 | 118.43 | 107.81 | 97.55 |
| Rwanda | 75.36 | 79.17 | 76.40 | 74.20 | 67.83 |
| Saint Lucia | 375.85 | 383.30 | 290.40 | 238.63 | 218.83 |
| Saint Vincent and the Grenadines | 365.04 | 351.59 | 275.46 | 221.95 | 201.74 |
| Samoa | 210.37 | 227.46 | 177.37 | 144.83 | 124.51 |
| Sao Tome and Principe | 169.20 | 161.41 | 126.49 | 117.70 | 109.12 |
| Saudi Arabia | 337.43 | 319.86 | 249.48 | 211.28 | 191.70 |
| Senegal | 206.47 | 202.57 | 165.28 | 138.80 | 123.88 |
| Serbia | 402.12 | 408.24 | 308.16 | 240.78 | 212.14 |
| Seychelles | 249.39 | 239.01 | 206.43 | 178.88 | 153.22 |
| Sierra Leone | 121.54 | 103.90 | 83.04 | 71.92 | 76.70 |
| Singapore | 191.33 | 191.70 | 145.35 | 116.89 | 94.05 |
| Slovakia | 200.83 | 222.51 | 196.50 | 162.60 | 128.50 |
| Slovenia | 152.12 | 163.04 | 136.76 | 117.43 | 100.29 |
| Solomon Islands | 85.30 | 95.52 | 87.71 | 82.30 | 74.64 |
| Somalia | 488.33 | 419.38 | 301.74 | 250.14 | 220.80 |
| South Africa | 43.64 | 42.13 | 36.86 | 35.23 | 35.15 |
| Republic of Korea | 142.58 | 134.08 | 123.00 | 115.71 | 104.90 |
| South Sudan | 44.57 | 41.36 | 37.08 | 33.73 | 33.53 |
| Spain | 304.89 | 329.26 | 297.80 | 266.53 | 212.45 |
| Sri Lanka | 131.06 | 146.87 | 135.62 | 126.84 | 104.49 |
| Sudan | 48.55 | 45.40 | 43.70 | 41.65 | 40.98 |
| Suriname | 260.23 | 243.53 | 168.76 | 130.18 | 114.74 |
| Eswatini | 357.66 | 347.82 | 266.54 | 217.26 | 184.93 |
| Sweden | 371.47 | 335.10 | 230.81 | 180.09 | 149.31 |
| Switzerland | 76.92 | 92.91 | 89.36 | 86.30 | 83.26 |
| Syrian Arab Republic | 71.83 | 58.86 | 50.04 | 48.06 | 55.47 |
| Taiwan (Province of China) | 65.12 | 79.73 | 87.80 | 89.53 | 77.93 |
| Tajikistan | 53.38 | 46.88 | 41.07 | 37.99 | 40.59 |
| United Republic of Tanzania | 332.27 | 296.73 | 230.33 | 192.67 | 172.71 |
| Thailand | 104.76 | 116.98 | 100.79 | 90.65 | 81.16 |
| Bahamas | 112.49 | 97.28 | 88.27 | 81.47 | 72.28 |
| Gambia | 297.65 | 294.67 | 259.24 | 225.47 | 191.43 |
| Timor-Leste | 270.10 | 257.94 | 183.92 | 145.24 | 131.41 |
| Togo | 339.04 | 292.96 | 201.44 | 159.47 | 137.15 |
| Tonga | 91.14 | 78.19 | 67.36 | 60.81 | 56.08 |
| Trinidad and Tobago | 223.47 | 211.79 | 155.96 | 143.29 | 130.59 |
| Tunisia | 71.11 | 71.01 | 61.01 | 52.69 | 46.87 |
| Turkey | 181.74 | 155.19 | 144.93 | 140.55 | 115.23 |
| Turkmenistan | 108.16 | 97.66 | 77.98 | 70.70 | 65.00 |
| Uganda | 64.40 | 59.66 | 55.14 | 51.16 | 49.97 |
| Ukraine | 63.51 | 76.14 | 69.76 | 62.54 | 57.56 |
| United Arab Emirates | 58.20 | 62.79 | 53.97 | 48.33 | 43.23 |
| United Kingdom | 201.26 | 205.87 | 160.35 | 129.55 | 107.55 |
| United States of America | 144.65 | 131.07 | 120.75 | 119.27 | 110.82 |
| Uruguay | 100.50 | 94.75 | 81.39 | 74.28 | 68.08 |
| Uzbekistan | 92.07 | 93.95 | 79.93 | 66.47 | 55.56 |
| Vanuatu | 76.07 | 91.60 | 87.92 | 84.42 | 75.12 |
| Venezuela (Bolivarian Republic of) | 114.55 | 96.27 | 77.34 | 68.83 | 74.06 |
| Viet nam | 278.64 | 239.99 | 197.75 | 177.08 | 160.47 |
| United States Virgin Islands | 163.56 | 159.15 | 148.97 | 168.02 | 148.33 |
| Yemen | 288.97 | 265.59 | 187.39 | 151.38 | 136.78 |
| Zambia | 62.77 | 55.58 | 48.14 | 45.04 | 47.19 |
| Zimbabwe | 374.71 | 337.34 | 266.72 | 224.84 | 212.15 |
| Monaco | 164.27 | 173.89 | 145.97 | 130.97 | 121.11 |
| San Marino | 439.67 | 399.02 | 295.51 | 249.46 | 230.70 |
| Saint Kitts and Nevis | 113.29 | 108.73 | 98.76 | 90.52 | 84.02 |
| Cook Islands | 84.50 | 98.10 | 94.91 | 91.35 | 81.53 |
| Nauru | 214.03 | 165.62 | 132.11 | 119.65 | 110.76 |
| Niue | 197.11 | 173.64 | 137.69 | 141.89 | 136.95 |
| Palau | 99.69 | 88.27 | 83.04 | 81.30 | 76.09 |
| Tokelau | 103.50 | 94.94 | 74.87 | 68.69 | 80.57 |
| Tuvalu | 63.38 | 57.54 | 52.13 | 54.45 | 60.19 |

Table S3 Age specific deaths rate of cyclist road injuries aged 15-39 in 204 countries, 2021

| deaths | 15-19 years | 20-24 years | 25-29 years | 30-34 years | 35-39 years |
| --- | --- | --- | --- | --- | --- |
| Afghanistan | 0.02 | 0.02 | 0.03 | 0.03 | 0.04 |
| Albania | 0.09 | 0.15 | 0.14 | 0.17 | 0.24 |
| Algeria | 0.02 | 0.02 | 0.02 | 0.02 | 0.02 |
| American Samoa | 0.02 | 0.09 | 0.11 | 0.07 | 0.10 |
| Andorra | 0.49 | 0.51 | 0.42 | 0.38 | 0.37 |
| Angola | 0.26 | 0.26 | 0.24 | 0.28 | 0.31 |
| Antigua and Barbuda | 0.30 | 0.24 | 0.17 | 0.14 | 0.13 |
| Argentina | 0.24 | 0.28 | 0.26 | 0.24 | 0.25 |
| Armenia | 0.16 | 0.28 | 0.27 | 0.28 | 0.31 |
| Australia | 0.17 | 0.14 | 0.13 | 0.12 | 0.15 |
| Austria | 0.33 | 0.23 | 0.16 | 0.16 | 0.19 |
| Azerbaijan | 0.02 | 0.02 | 0.03 | 0.04 | 0.04 |
| Bahrain | 0.36 | 0.51 | 0.47 | 0.55 | 0.58 |
| Bangladesh | 0.04 | 0.06 | 0.06 | 0.07 | 0.06 |
| Barbados | 0.42 | 0.23 | 0.29 | 0.29 | 0.27 |
| Belarus | 0.37 | 0.29 | 0.31 | 0.35 | 0.27 |
| Belgium | 0.41 | 0.43 | 0.45 | 0.59 | 0.68 |
| Belize | 0.33 | 0.24 | 0.24 | 0.24 | 0.31 |
| Benin | 1.02 | 1.50 | 1.83 | 1.26 | 1.42 |
| Bermuda | 0.18 | 0.23 | 0.26 | 0.32 | 0.38 |
| Bhutan | 0.15 | 0.14 | 0.10 | 0.11 | 0.13 |
| Bolivia (Plurinational State of) | 0.19 | 0.11 | 0.14 | 0.15 | 0.13 |
| Bosnia and Herzegovina | 0.07 | 0.08 | 0.08 | 0.10 | 0.13 |
| Botswana | 0.17 | 0.33 | 0.31 | 0.19 | 0.21 |
| Brazil | 0.12 | 0.12 | 0.09 | 0.10 | 0.13 |
| Brunei Darussalam | 0.48 | 0.54 | 0.56 | 0.60 | 0.74 |
| Bulgaria | 0.28 | 0.28 | 0.42 | 0.41 | 0.52 |
| Burkina Faso | 0.23 | 0.34 | 0.29 | 0.26 | 0.31 |
| Burundi | 0.26 | 0.33 | 0.37 | 0.47 | 0.64 |
| Cambodia | 0.72 | 0.61 | 0.59 | 0.60 | 0.82 |
| Cameroon | 0.21 | 0.28 | 0.24 | 0.32 | 0.40 |
| Canada | 0.77 | 0.93 | 0.71 | 0.96 | 0.99 |
| Cabo Verde | 0.29 | 0.40 | 0.47 | 0.58 | 0.72 |
| Central African Republic | 0.25 | 0.24 | 0.24 | 0.24 | 0.22 |
| Chad | 0.42 | 0.35 | 0.31 | 0.36 | 0.47 |
| Chile | 0.23 | 0.27 | 0.27 | 0.30 | 0.38 |
| China | 0.26 | 0.34 | 0.35 | 0.39 | 0.40 |
| Colombia | 0.75 | 1.06 | 1.02 | 1.21 | 1.44 |
| Comoros | 0.78 | 0.67 | 0.54 | 0.50 | 0.50 |
| Congo | 0.47 | 0.42 | 0.41 | 0.38 | 0.48 |
| Costa Rica | 0.26 | 0.26 | 0.26 | 0.29 | 0.33 |
| Côte d'Ivoire | 0.08 | 0.41 | 0.44 | 0.31 | 0.50 |
| Croatia | 0.51 | 0.70 | 0.65 | 0.70 | 0.84 |
| Cuba | 0.29 | 0.30 | 0.26 | 0.27 | 0.32 |
| Cyprus | 0.97 | 1.00 | 0.97 | 1.12 | 1.25 |
| Czechia | 0.05 | 0.21 | 0.12 | 0.09 | 0.11 |
| Democratic Republic of the Congo | 0.25 | 0.32 | 0.30 | 0.32 | 0.34 |
| Denmark | 0.22 | 0.29 | 0.29 | 0.34 | 0.45 |
| Djibouti | 0.86 | 1.18 | 1.19 | 1.23 | 1.46 |
| Dominica | 0.22 | 0.23 | 0.22 | 0.25 | 0.28 |
| Dominican Republic | 0.34 | 0.31 | 0.21 | 0.18 | 0.22 |
| Ecuador | 0.38 | 0.34 | 0.34 | 0.35 | 0.46 |
| Egypt | 0.68 | 0.58 | 0.46 | 0.48 | 0.55 |
| El Salvador | 0.37 | 0.44 | 0.43 | 0.41 | 0.41 |
| Equatorial Guinea | 0.32 | 0.66 | 0.71 | 0.85 | 0.81 |
| Eritrea | 0.20 | 0.21 | 0.20 | 0.20 | 0.15 |
| Estonia | 0.30 | 0.35 | 0.35 | 0.40 | 0.51 |
| Ethiopia | 0.27 | 0.26 | 0.25 | 0.29 | 0.31 |
| Micronesia (Federated States of) | 0.68 | 0.66 | 0.62 | 0.70 | 0.95 |
| Fiji | 0.21 | 0.19 | 0.18 | 0.20 | 0.27 |
| Finland | 0.30 | 0.41 | 0.43 | 0.64 | 0.96 |
| France | 0.26 | 0.23 | 0.23 | 0.25 | 0.33 |
| Gabon | 0.05 | 0.18 | 0.15 | 0.10 | 0.16 |
| Georgia | 0.56 | 0.36 | 0.23 | 0.24 | 0.26 |
| Germany | 0.13 | 0.17 | 0.13 | 0.15 | 0.15 |
| Ghana | 0.34 | 0.33 | 0.32 | 0.38 | 0.45 |
| Greece | 0.22 | 0.28 | 0.27 | 0.30 | 0.38 |
| Greenland | 0.19 | 0.24 | 0.26 | 0.29 | 0.31 |
| Grenada | 0.33 | 0.21 | 0.19 | 0.19 | 0.20 |
| Guam | 0.60 | 0.65 | 0.60 | 0.67 | 0.89 |
| Guatemala | 0.06 | 0.05 | 0.04 | 0.05 | 0.05 |
| Guinea | 0.18 | 0.14 | 0.13 | 0.12 | 0.10 |
| Guinea-Bissau | 0.56 | 0.58 | 0.78 | 0.80 | 0.90 |
| Guyana | 0.04 | 0.15 | 0.15 | 0.10 | 0.16 |
| Haiti | 0.20 | 0.28 | 0.28 | 0.30 | 0.33 |
| Honduras | 0.17 | 0.21 | 0.22 | 0.25 | 0.30 |
| Hungary | 0.50 | 0.62 | 0.62 | 0.69 | 0.82 |
| Iceland | 1.06 | 1.49 | 1.49 | 1.55 | 1.40 |
| India | 1.32 | 1.21 | 1.09 | 1.14 | 1.22 |
| Indonesia | 0.20 | 0.21 | 0.20 | 0.22 | 0.29 |
| Iran (Islamic Republic of) | 0.54 | 0.77 | 0.69 | 0.66 | 0.68 |
| Iraq | 0.09 | 0.05 | 0.08 | 0.07 | 0.04 |
| Ireland | 0.75 | 0.63 | 0.78 | 1.04 | 1.01 |
| Israel | 0.58 | 0.55 | 0.37 | 0.50 | 0.49 |
| Italy | 0.09 | 0.12 | 0.11 | 0.09 | 0.08 |
| Jamaica | 0.07 | 0.07 | 0.06 | 0.07 | 0.08 |
| Japan | 0.17 | 0.12 | 0.13 | 0.15 | 0.15 |
| Jordan | 0.15 | 0.14 | 0.10 | 0.12 | 0.10 |
| Kazakhstan | 0.20 | 0.18 | 0.19 | 0.19 | 0.22 |
| Kenya | 0.21 | 0.28 | 0.30 | 0.32 | 0.34 |
| Kiribati | 0.28 | 0.19 | 0.12 | 0.11 | 0.14 |
| Kuwait | 0.05 | 0.07 | 0.08 | 0.09 | 0.08 |
| Kyrgyzstan | 0.08 | 0.12 | 0.15 | 0.21 | 0.24 |
| Lao People's Democratic Republic | 0.39 | 0.32 | 0.29 | 0.33 | 0.49 |
| Latvia | 0.02 | 0.09 | 0.09 | 0.06 | 0.11 |
| Lebanon | 0.40 | 0.48 | 0.43 | 0.43 | 0.38 |
| Lesotho | 0.11 | 0.18 | 0.29 | 0.39 | 0.42 |
| Liberia | 0.59 | 0.74 | 0.61 | 0.81 | 0.79 |
| Libya | 0.31 | 0.42 | 0.39 | 0.47 | 0.51 |
| Lithuania | 0.08 | 0.10 | 0.09 | 0.09 | 0.10 |
| Luxembourg | 0.27 | 0.30 | 0.30 | 0.45 | 0.74 |
| North Macedonia | 0.16 | 0.23 | 0.24 | 0.27 | 0.34 |
| Madagascar | 0.03 | 0.04 | 0.04 | 0.05 | 0.05 |
| Malawi | 0.39 | 0.44 | 0.41 | 0.67 | 0.71 |
| Malaysia | 0.11 | 0.07 | 0.03 | 0.05 | 0.07 |
| Maldives | 0.38 | 0.33 | 0.31 | 0.29 | 0.37 |
| Mali | 0.51 | 0.46 | 0.44 | 0.47 | 0.62 |
| Malta | 0.65 | 0.77 | 0.58 | 0.86 | 0.88 |
| Marshall Islands | 0.06 | 0.08 | 0.08 | 0.11 | 0.10 |
| Mauritania | 0.13 | 0.16 | 0.17 | 0.20 | 0.25 |
| Mauritius | 0.05 | 0.05 | 0.02 | 0.03 | 0.03 |
| Mexico | 0.02 | 0.09 | 0.10 | 0.06 | 0.11 |
| Republic of Moldova | 0.27 | 0.34 | 0.36 | 0.41 | 0.47 |
| Mongolia | 0.20 | 0.35 | 0.29 | 0.40 | 0.45 |
| Montenegro | 0.32 | 0.48 | 0.50 | 0.49 | 0.54 |
| Morocco | 0.03 | 0.15 | 0.14 | 0.10 | 0.16 |
| Mozambique | 0.15 | 0.16 | 0.14 | 0.14 | 0.13 |
| Myanmar | 0.04 | 0.05 | 0.06 | 0.09 | 0.11 |
| Namibia | 0.28 | 0.28 | 0.28 | 0.30 | 0.29 |
| Nepal | 0.02 | 0.02 | 0.01 | 0.02 | 0.02 |
| Netherlands | 0.70 | 0.60 | 0.56 | 0.59 | 0.74 |
| New Zealand | 0.45 | 0.53 | 0.41 | 0.51 | 0.49 |
| Nicaragua | 0.19 | 0.25 | 0.25 | 0.37 | 0.51 |
| Niger | 0.11 | 0.42 | 0.40 | 0.30 | 0.51 |
| Nigeria | 0.33 | 0.24 | 0.27 | 0.37 | 0.32 |
| Democratic People's Republic of Korea | 0.52 | 0.52 | 0.36 | 0.41 | 0.46 |
| Northern Mariana Islands | 0.24 | 0.17 | 0.16 | 0.21 | 0.21 |
| Norway | 0.25 | 0.33 | 0.38 | 0.40 | 0.51 |
| Oman | 0.14 | 0.18 | 0.22 | 0.26 | 0.31 |
| Pakistan | 0.11 | 0.16 | 0.21 | 0.26 | 0.33 |
| Palestine | 0.07 | 0.19 | 0.15 | 0.09 | 0.14 |
| Panama | 0.10 | 0.10 | 0.07 | 0.07 | 0.09 |
| Papua New Guinea | 0.01 | 0.06 | 0.07 | 0.05 | 0.08 |
| Paraguay | 0.13 | 0.11 | 0.11 | 0.11 | 0.15 |
| Peru | 0.17 | 0.27 | 0.29 | 0.29 | 0.28 |
| Philippines | 0.77 | 0.58 | 0.74 | 0.82 | 0.81 |
| Poland | 0.01 | 0.02 | 0.00 | 0.00 | 0.00 |
| Portugal | 0.01 | 0.02 | 0.02 | 0.02 | 0.01 |
| Puerto Rico | 0.58 | 0.55 | 0.56 | 0.62 | 0.62 |
| Qatar | 0.04 | 0.09 | 0.08 | 0.07 | 0.11 |
| Romania | 0.23 | 0.21 | 0.20 | 0.23 | 0.26 |
| Russian Federation | 0.26 | 0.50 | 0.55 | 0.67 | 0.77 |
| Rwanda | 0.21 | 0.36 | 0.37 | 0.55 | 0.56 |
| Saint Lucia | 0.66 | 0.72 | 0.67 | 0.68 | 0.74 |
| Saint Vincent and the Grenadines | 0.20 | 0.19 | 0.14 | 0.18 | 0.17 |
| Samoa | 0.42 | 0.69 | 0.66 | 0.69 | 0.69 |
| Sao Tome and Principe | 0.34 | 0.26 | 0.24 | 0.24 | 0.25 |
| Saudi Arabia | 0.22 | 0.13 | 0.14 | 0.13 | 0.16 |
| Senegal | 0.23 | 0.20 | 0.18 | 0.24 | 0.27 |
| Serbia | 0.41 | 0.54 | 0.42 | 0.42 | 0.49 |
| Seychelles | 0.14 | 0.17 | 0.20 | 0.25 | 0.26 |
| Sierra Leone | 1.32 | 1.02 | 0.89 | 0.83 | 1.11 |
| Singapore | 0.44 | 0.34 | 0.24 | 0.23 | 0.31 |
| Slovakia | 0.58 | 0.68 | 0.75 | 0.74 | 0.64 |
| Slovenia | 0.52 | 0.52 | 0.53 | 0.50 | 0.57 |
| Solomon Islands | 0.03 | 0.13 | 0.13 | 0.09 | 0.13 |
| Somalia | 0.42 | 0.45 | 0.36 | 0.36 | 0.32 |
| South Africa | 0.19 | 0.27 | 0.27 | 0.29 | 0.35 |
| Republic of Korea | 0.01 | 0.02 | 0.03 | 0.03 | 0.04 |
| South Sudan | 0.15 | 0.19 | 0.19 | 0.21 | 0.25 |
| Spain | 0.21 | 0.34 | 0.36 | 0.45 | 0.49 |
| Sri Lanka | 0.00 | 0.33 | 0.49 | 0.45 | 0.49 |
| Sudan | 0.17 | 0.24 | 0.29 | 0.35 | 0.40 |
| Suriname | 0.08 | 0.05 | 0.07 | 0.04 | 0.05 |
| Eswatini | 0.52 | 0.57 | 0.55 | 0.60 | 0.56 |
| Sweden | 0.34 | 0.25 | 0.24 | 0.25 | 0.23 |
| Switzerland | 0.03 | 0.11 | 0.12 | 0.08 | 0.15 |
| Syrian Arab Republic | 0.75 | 0.61 | 0.53 | 0.60 | 0.84 |
| Taiwan (Province of China) | 0.12 | 0.30 | 0.42 | 0.63 | 0.62 |
| Tajikistan | 0.60 | 0.39 | 0.34 | 0.35 | 0.53 |
| United Republic of Tanzania | 0.12 | 0.11 | 0.10 | 0.11 | 0.11 |
| Thailand | 0.26 | 0.61 | 0.52 | 0.61 | 0.62 |
| Bahamas | 0.02 | 0.02 | 0.02 | 0.02 | 0.03 |
| Gambia | 1.08 | 1.24 | 0.96 | 1.22 | 1.16 |
| Timor-Leste | 0.09 | 0.10 | 0.08 | 0.08 | 0.08 |
| Togo | 0.24 | 0.17 | 0.16 | 0.14 | 0.14 |
| Tonga | 0.08 | 0.05 | 0.05 | 0.05 | 0.07 |
| Trinidad and Tobago | 0.47 | 0.53 | 0.41 | 0.40 | 0.52 |
| Tunisia | 0.02 | 0.02 | 0.03 | 0.04 | 0.04 |
| Turkey | 0.56 | 0.69 | 0.68 | 0.99 | 0.87 |
| Turkmenistan | 0.28 | 0.34 | 0.26 | 0.34 | 0.34 |
| Uganda | 0.36 | 0.47 | 0.47 | 0.50 | 0.61 |
| Ukraine | 0.05 | 0.13 | 0.11 | 0.07 | 0.08 |
| United Arab Emirates | 0.02 | 0.08 | 0.07 | 0.05 | 0.07 |
| United Kingdom | 0.50 | 0.74 | 0.70 | 0.63 | 0.64 |
| United States of America | 0.22 | 0.32 | 0.36 | 0.44 | 0.48 |
| Uruguay | 0.21 | 0.23 | 0.23 | 0.24 | 0.24 |
| Uzbekistan | 0.08 | 0.10 | 0.11 | 0.13 | 0.13 |
| Vanuatu | 0.04 | 0.17 | 0.17 | 0.12 | 0.19 |
| Venezuela (Bolivarian Republic of) | 1.31 | 1.13 | 1.04 | 1.01 | 1.30 |
| Viet nam | 0.14 | 0.16 | 0.20 | 0.24 | 0.27 |
| United States Virgin Islands | 0.57 | 1.51 | 1.23 | 0.97 | 0.59 |
| Yemen | 0.18 | 0.17 | 0.12 | 0.11 | 0.13 |
| Zambia | 0.41 | 0.41 | 0.43 | 0.44 | 0.57 |
| Zimbabwe | 0.23 | 0.23 | 0.25 | 0.27 | 0.29 |
| Monaco | 0.78 | 1.71 | 1.31 | 0.99 | 0.83 |
| San Marino | 0.24 | 0.31 | 0.25 | 0.27 | 0.31 |
| Saint Kitts and Nevis | 0.24 | 0.32 | 0.45 | 0.55 | 0.51 |
| Cook Islands | 0.04 | 0.16 | 0.17 | 0.11 | 0.18 |
| Nauru | 0.43 | 0.48 | 0.38 | 0.35 | 0.35 |
| Niue | 0.70 | 0.81 | 0.57 | 0.88 | 1.04 |
| Palau | 0.02 | 0.02 | 0.03 | 0.03 | 0.04 |
| Tokelau | 1.29 | 1.20 | 1.08 | 1.11 | 1.49 |
| Tuvalu | 0.42 | 0.44 | 0.41 | 0.54 | 0.74 |

Table S4 Age specific DALYs rate of cyclist road injuries aged 15-39 in 204 countries, 2021

| DALYs (Disability-Adjusted Life Years) | 15-19 years | 20-24 years | 25-29 years | 30-34 years | 35-39 years |
| --- | --- | --- | --- | --- | --- |
| Afghanistan | 5.66 | 7.59 | 9.47 | 11.34 | 13.20 |
| Albania | 16.42 | 24.08 | 26.64 | 31.50 | 38.03 |
| Algeria | 6.19 | 8.40 | 10.01 | 11.75 | 13.70 |
| American Samoa | 3.39 | 9.86 | 11.74 | 10.02 | 12.92 |
| Andorra | 44.41 | 48.40 | 44.09 | 43.88 | 45.22 |
| Angola | 23.06 | 22.75 | 21.38 | 23.27 | 25.08 |
| Antigua and Barbuda | 25.24 | 22.57 | 18.85 | 17.54 | 18.40 |
| Argentina | 26.59 | 32.15 | 33.58 | 34.45 | 37.29 |
| Armenia | 15.35 | 23.95 | 23.59 | 24.34 | 26.18 |
| Australia | 16.60 | 15.73 | 16.45 | 16.78 | 19.37 |
| Austria | 32.17 | 27.09 | 24.15 | 25.72 | 29.07 |
| Azerbaijan | 4.83 | 6.46 | 7.91 | 9.58 | 10.81 |
| Bahrain | 31.03 | 43.04 | 41.33 | 46.61 | 48.22 |
| Bangladesh | 6.59 | 9.44 | 11.43 | 13.10 | 14.14 |
| Barbados | 33.51 | 19.11 | 22.89 | 22.91 | 21.45 |
| Belarus | 30.89 | 26.83 | 28.45 | 31.77 | 27.53 |
| Belgium | 35.51 | 38.02 | 39.72 | 48.10 | 52.78 |
| Belize | 32.63 | 29.15 | 30.41 | 32.67 | 38.34 |
| Benin | 81.94 | 116.58 | 136.07 | 100.10 | 106.89 |
| Bermuda | 15.58 | 19.08 | 20.89 | 23.65 | 26.20 |
| Bhutan | 14.48 | 15.71 | 14.03 | 15.97 | 18.81 |
| Bolivia (Plurinational State of) | 16.40 | 12.11 | 14.29 | 15.96 | 15.33 |
| Bosnia and Herzegovina | 9.58 | 11.09 | 12.29 | 15.23 | 18.06 |
| Botswana | 19.74 | 34.15 | 34.36 | 28.58 | 32.20 |
| Brazil | 12.47 | 13.06 | 11.86 | 13.06 | 15.20 |
| Brunei Darussalam | 42.06 | 46.78 | 48.39 | 50.68 | 57.60 |
| Bulgaria | 30.70 | 35.58 | 48.93 | 50.96 | 60.21 |
| Burkina Faso | 26.96 | 38.64 | 38.78 | 39.81 | 45.18 |
| Burundi | 23.64 | 27.89 | 30.14 | 35.34 | 43.04 |
| Cambodia | 56.12 | 47.10 | 44.09 | 43.17 | 53.29 |
| Cameroon | 17.07 | 21.03 | 18.23 | 22.19 | 26.04 |
| Canada | 60.01 | 69.28 | 53.08 | 65.66 | 64.66 |
| Cabo Verde | 24.26 | 31.24 | 34.86 | 40.13 | 46.00 |
| Central African Republic | 24.04 | 25.51 | 26.10 | 26.68 | 26.68 |
| Chad | 34.79 | 28.66 | 25.09 | 27.67 | 32.32 |
| Chile | 20.13 | 22.39 | 22.25 | 23.58 | 27.24 |
| China | 27.65 | 37.22 | 40.35 | 44.83 | 46.91 |
| Colombia | 60.82 | 82.80 | 79.45 | 91.08 | 103.98 |
| Comoros | 62.29 | 54.26 | 45.21 | 41.87 | 41.66 |
| Congo | 38.17 | 33.73 | 32.14 | 29.23 | 34.19 |
| Costa Rica | 21.93 | 21.19 | 20.91 | 22.35 | 23.63 |
| Côte d'Ivoire | 8.06 | 31.91 | 33.82 | 25.86 | 36.69 |
| Croatia | 41.91 | 54.60 | 49.74 | 51.77 | 58.24 |
| Cuba | 30.82 | 35.04 | 35.20 | 37.94 | 43.12 |
| Cyprus | 75.32 | 75.60 | 71.81 | 78.34 | 83.35 |
| Czechia | 12.90 | 27.68 | 24.60 | 25.53 | 29.34 |
| Democratic Republic of the Congo | 26.70 | 35.33 | 36.04 | 39.16 | 42.30 |
| Denmark | 18.93 | 23.23 | 22.67 | 25.57 | 30.82 |
| Djibouti | 68.11 | 90.59 | 90.08 | 91.21 | 103.41 |
| Dominica | 19.29 | 19.83 | 18.96 | 20.45 | 21.74 |
| Dominican Republic | 32.65 | 32.93 | 27.86 | 27.36 | 31.76 |
| Ecuador | 30.91 | 27.77 | 26.55 | 26.63 | 31.57 |
| Egypt | 56.42 | 50.01 | 43.08 | 44.88 | 48.96 |
| El Salvador | 33.68 | 40.26 | 41.08 | 41.49 | 42.66 |
| Equatorial Guinea | 28.14 | 52.71 | 55.92 | 63.71 | 60.54 |
| Eritrea | 19.53 | 20.62 | 20.49 | 21.50 | 20.29 |
| Estonia | 26.65 | 30.54 | 30.31 | 33.21 | 39.23 |
| Ethiopia | 22.81 | 21.77 | 20.81 | 22.40 | 23.28 |
| Micronesia (Federated States of) | 52.87 | 49.51 | 45.36 | 48.03 | 59.68 |
| Fiji | 20.00 | 20.09 | 20.60 | 23.04 | 28.26 |
| Finland | 25.55 | 32.91 | 33.41 | 44.49 | 59.68 |
| France | 21.16 | 18.82 | 18.39 | 18.77 | 22.73 |
| Gabon | 6.37 | 16.02 | 14.57 | 12.31 | 16.27 |
| Georgia | 48.19 | 35.61 | 27.63 | 29.63 | 32.19 |
| Germany | 17.68 | 23.98 | 23.45 | 27.17 | 29.50 |
| Ghana | 27.59 | 26.30 | 25.12 | 27.95 | 30.86 |
| Greece | 18.06 | 21.67 | 20.22 | 21.50 | 25.00 |
| Greenland | 18.88 | 22.72 | 24.23 | 26.11 | 27.43 |
| Grenada | 31.83 | 26.05 | 25.94 | 27.80 | 29.86 |
| Guam | 47.31 | 48.75 | 43.80 | 46.01 | 56.11 |
| Guatemala | 13.22 | 16.50 | 18.65 | 21.80 | 25.34 |
| Guinea | 17.35 | 15.93 | 16.10 | 16.33 | 16.52 |
| Guinea-Bissau | 45.17 | 46.39 | 58.45 | 58.78 | 62.42 |
| Guyana | 5.13 | 13.75 | 14.28 | 12.38 | 16.25 |
| Haiti | 19.81 | 26.65 | 27.80 | 29.27 | 31.85 |
| Honduras | 15.57 | 18.41 | 19.35 | 20.71 | 23.57 |
| Hungary | 40.12 | 47.49 | 45.35 | 48.14 | 52.87 |
| Iceland | 85.14 | 115.04 | 113.74 | 114.98 | 103.70 |
| India | 108.43 | 99.00 | 89.59 | 90.59 | 93.17 |
| Indonesia | 19.94 | 21.68 | 21.83 | 24.50 | 29.33 |
| Iran (Islamic Republic of) | 46.59 | 63.81 | 58.27 | 56.43 | 57.80 |
| Iraq | 13.21 | 13.20 | 17.27 | 18.15 | 18.82 |
| Ireland | 57.95 | 48.28 | 56.61 | 70.65 | 66.94 |
| Israel | 46.22 | 43.86 | 31.58 | 38.26 | 36.94 |
| Italy | 12.94 | 16.79 | 18.35 | 18.89 | 20.48 |
| Jamaica | 8.17 | 9.41 | 9.98 | 11.47 | 13.38 |
| Japan | 19.93 | 18.74 | 20.88 | 23.65 | 25.42 |
| Jordan | 18.08 | 19.83 | 19.10 | 22.30 | 23.81 |
| Kazakhstan | 23.30 | 24.08 | 26.62 | 28.46 | 32.28 |
| Kenya | 18.41 | 24.69 | 26.45 | 27.88 | 28.79 |
| Kiribati | 25.89 | 21.17 | 18.44 | 19.38 | 22.57 |
| Kuwait | 6.91 | 9.62 | 11.21 | 12.63 | 13.22 |
| Kyrgyzstan | 10.82 | 14.89 | 17.77 | 21.74 | 24.52 |
| Lao People's Democratic Republic | 30.79 | 24.73 | 22.66 | 24.63 | 32.66 |
| Latvia | 3.89 | 10.73 | 11.97 | 11.21 | 15.15 |
| Lebanon | 33.14 | 37.58 | 33.87 | 33.03 | 29.82 |
| Lesotho | 11.29 | 17.12 | 24.54 | 30.45 | 31.76 |
| Liberia | 47.27 | 56.77 | 46.98 | 57.87 | 55.23 |
| Libya | 28.14 | 37.85 | 37.20 | 42.50 | 46.10 |
| Lithuania | 8.63 | 11.12 | 11.38 | 12.21 | 13.42 |
| Luxembourg | 23.05 | 24.99 | 24.03 | 33.04 | 47.33 |
| North Macedonia | 14.15 | 18.43 | 19.14 | 20.29 | 23.48 |
| Madagascar | 6.11 | 7.94 | 9.41 | 11.69 | 13.78 |
| Malawi | 35.01 | 39.64 | 38.81 | 54.87 | 56.83 |
| Malaysia | 15.95 | 16.38 | 16.56 | 20.59 | 24.41 |
| Maldives | 30.65 | 26.68 | 24.55 | 23.44 | 27.39 |
| Mali | 40.45 | 35.84 | 33.01 | 33.89 | 40.58 |
| Malta | 50.89 | 58.49 | 44.74 | 60.40 | 59.56 |
| Marshall Islands | 6.30 | 8.23 | 9.51 | 11.90 | 11.39 |
| Mauritania | 11.71 | 13.92 | 14.89 | 16.28 | 18.64 |
| Mauritius | 11.53 | 14.25 | 14.65 | 17.88 | 20.45 |
| Mexico | 3.61 | 9.88 | 10.89 | 9.90 | 13.36 |
| Republic of Moldova | 22.58 | 27.20 | 28.16 | 29.99 | 32.52 |
| Mongolia | 17.04 | 27.22 | 23.09 | 29.69 | 31.76 |
| Montenegro | 29.84 | 41.92 | 43.38 | 42.65 | 46.25 |
| Morocco | 5.01 | 14.52 | 15.51 | 14.60 | 19.06 |
| Mozambique | 18.82 | 22.42 | 23.18 | 25.40 | 26.91 |
| Myanmar | 7.34 | 9.82 | 11.76 | 14.82 | 17.46 |
| Namibia | 28.98 | 32.22 | 35.30 | 38.27 | 39.99 |
| Nepal | 5.58 | 7.27 | 8.71 | 10.36 | 12.29 |
| Netherlands | 55.12 | 46.11 | 42.29 | 42.01 | 48.82 |
| New Zealand | 36.11 | 41.38 | 32.66 | 38.06 | 36.29 |
| Nicaragua | 16.54 | 21.20 | 20.98 | 28.17 | 35.06 |
| Niger | 11.97 | 35.43 | 34.73 | 29.58 | 41.83 |
| Nigeria | 26.24 | 19.60 | 21.59 | 26.77 | 23.91 |
| Democratic People's Republic of Korea | 46.08 | 46.88 | 37.02 | 40.12 | 43.02 |
| Northern Mariana Islands | 24.08 | 21.56 | 22.25 | 26.83 | 28.04 |
| Norway | 23.70 | 29.31 | 32.49 | 33.41 | 38.65 |
| Oman | 12.99 | 16.57 | 18.66 | 20.93 | 23.02 |
| Pakistan | 10.51 | 14.21 | 16.93 | 19.99 | 23.25 |
| Palestine | 7.61 | 16.87 | 15.07 | 12.12 | 15.56 |
| Panama | 15.51 | 18.87 | 20.29 | 22.93 | 26.93 |
| Papua New Guinea | 3.25 | 7.70 | 9.35 | 9.19 | 12.56 |
| Paraguay | 14.89 | 15.70 | 17.54 | 19.19 | 23.72 |
| Peru | 20.50 | 29.64 | 32.99 | 33.70 | 34.35 |
| Philippines | 59.67 | 45.21 | 54.13 | 56.91 | 53.87 |
| Poland | 3.31 | 5.95 | 6.89 | 8.97 | 10.97 |
| Portugal | 3.71 | 5.25 | 6.15 | 7.09 | 8.02 |
| Puerto Rico | 47.44 | 45.53 | 46.37 | 49.48 | 49.34 |
| Qatar | 5.94 | 9.80 | 9.88 | 10.10 | 13.35 |
| Romania | 21.73 | 21.68 | 21.98 | 24.59 | 27.41 |
| Russian Federation | 21.96 | 38.79 | 41.76 | 47.81 | 51.73 |
| Rwanda | 18.05 | 28.14 | 28.49 | 38.85 | 38.13 |
| Saint Lucia | 56.29 | 62.48 | 60.67 | 61.66 | 66.19 |
| Saint Vincent and the Grenadines | 21.70 | 23.38 | 22.59 | 26.73 | 28.65 |
| Samoa | 35.10 | 54.34 | 51.64 | 52.49 | 51.26 |
| Sao Tome and Principe | 29.59 | 24.96 | 24.58 | 25.64 | 27.53 |
| Saudi Arabia | 22.52 | 19.04 | 21.32 | 23.24 | 26.97 |
| Senegal | 23.11 | 23.09 | 23.36 | 28.70 | 31.45 |
| Serbia | 40.22 | 52.99 | 47.68 | 49.79 | 55.91 |
| Seychelles | 17.34 | 22.04 | 26.22 | 30.84 | 33.37 |
| Sierra Leone | 101.02 | 75.89 | 64.29 | 58.08 | 70.52 |
| Singapore | 37.03 | 31.56 | 26.26 | 27.12 | 32.51 |
| Slovakia | 47.52 | 54.78 | 59.71 | 58.22 | 52.16 |
| Slovenia | 42.36 | 42.65 | 43.19 | 40.71 | 44.79 |
| Solomon Islands | 4.72 | 12.82 | 13.70 | 12.92 | 16.45 |
| Somalia | 38.89 | 43.83 | 39.05 | 40.26 | 40.02 |
| South Africa | 15.63 | 20.73 | 19.92 | 20.87 | 23.59 |
| Republic of Korea | 4.46 | 7.18 | 9.49 | 11.93 | 14.36 |
| South Sudan | 12.95 | 16.19 | 15.95 | 16.98 | 18.65 |
| Spain | 22.69 | 35.59 | 39.89 | 47.65 | 52.19 |
| Sri Lanka | 3.18 | 27.84 | 38.42 | 36.16 | 38.45 |
| Sudan | 15.94 | 20.61 | 23.19 | 26.22 | 28.43 |
| Suriname | 11.41 | 11.33 | 13.87 | 14.13 | 16.28 |
| Eswatini | 46.68 | 52.48 | 52.74 | 56.29 | 55.57 |
| Sweden | 33.83 | 30.62 | 32.34 | 35.02 | 35.39 |
| Switzerland | 4.08 | 11.23 | 13.23 | 12.33 | 17.56 |
| Syrian Arab Republic | 57.88 | 46.51 | 39.77 | 41.75 | 52.92 |
| Taiwan (Province of China) | 11.38 | 24.10 | 32.21 | 44.01 | 41.93 |
| Tajikistan | 46.48 | 29.79 | 25.70 | 25.41 | 34.12 |
| United Republic of Tanzania | 16.20 | 18.16 | 19.59 | 22.33 | 24.73 |
| Thailand | 21.28 | 45.31 | 37.97 | 42.49 | 41.39 |
| Bahamas | 6.29 | 8.08 | 9.75 | 11.39 | 13.01 |
| Gambia | 87.22 | 97.36 | 78.66 | 93.38 | 89.35 |
| Timor-Leste | 12.23 | 15.60 | 15.99 | 17.60 | 20.07 |
| Togo | 25.36 | 22.67 | 23.51 | 24.11 | 25.81 |
| Tonga | 8.99 | 7.83 | 8.22 | 9.43 | 11.44 |
| Trinidad and Tobago | 38.43 | 42.86 | 34.72 | 34.38 | 41.29 |
| Tunisia | 4.22 | 5.85 | 7.69 | 9.12 | 10.28 |
| Turkey | 44.88 | 52.76 | 50.97 | 67.44 | 58.58 |
| Turkmenistan | 23.40 | 28.44 | 22.91 | 27.48 | 27.52 |
| Uganda | 29.28 | 36.39 | 35.17 | 35.66 | 39.74 |
| Ukraine | 5.24 | 11.90 | 11.12 | 9.57 | 11.02 |
| United Arab Emirates | 3.10 | 7.97 | 7.88 | 7.49 | 9.54 |
| United Kingdom | 41.66 | 58.91 | 55.86 | 50.96 | 50.71 |
| United States of America | 20.70 | 28.00 | 30.42 | 35.90 | 38.01 |
| Uruguay | 18.73 | 20.08 | 20.47 | 20.96 | 21.21 |
| Uzbekistan | 9.17 | 11.75 | 13.09 | 14.95 | 15.66 |
| Vanuatu | 5.31 | 15.28 | 16.43 | 14.40 | 19.35 |
| Venezuela (Bolivarian Republic of) | 99.88 | 83.41 | 73.48 | 68.06 | 80.06 |
| Viet nam | 17.57 | 21.69 | 26.54 | 31.36 | 34.82 |
| United States Virgin Islands | 45.46 | 108.99 | 86.83 | 69.05 | 47.92 |
| Yemen | 19.24 | 20.69 | 18.79 | 19.90 | 22.52 |
| Zambia | 32.92 | 32.00 | 32.22 | 31.66 | 37.56 |
| Zimbabwe | 23.98 | 26.30 | 29.37 | 32.25 | 34.91 |
| Monaco | 60.98 | 122.87 | 91.78 | 69.47 | 58.99 |
| San Marino | 26.00 | 33.75 | 31.72 | 34.93 | 39.56 |
| Saint Kitts and Nevis | 21.94 | 27.60 | 36.52 | 41.43 | 39.10 |
| Cook Islands | 5.60 | 15.31 | 17.40 | 15.05 | 20.30 |
| Nauru | 38.25 | 41.67 | 35.05 | 33.36 | 34.61 |
| Niue | 54.74 | 61.17 | 44.51 | 61.79 | 68.89 |
| Palau | 5.91 | 7.71 | 9.59 | 11.64 | 14.01 |
| Tokelau | 97.52 | 86.93 | 75.65 | 73.03 | 89.82 |
| Tuvalu | 33.29 | 33.73 | 31.10 | 37.31 | 46.83 |

Table S5 Cases number, age standardized incidence rate and its trends of cyclist road injuries age 15-39 in 204 countries, 1990-2021

| incidence |  | Incidence No.(95%UI) |  |  | ASIR (per 100000) No.95%UI |  |  |
| --- | --- | --- | --- | --- | --- | --- | --- |
| nation | sex | 1990 | 2021 | 1990-2021 EAPC No.(95%CI) | 1990 | 2021 | 1990-2021 EAPC No.(95%CI) |
| Afghanistan | both | 72.46(69.21,75.85) | 81.82(80.18,83.49) | 0.80(0.60,1.01) | 72.46(69.21,75.85) | 81.82(80.18,83.49) | 0.80(0.60,1.01) |
| Albania | both | 261.25(252.95,269.77) | 254.92(244.76,265.40) | 0.04(-0.06,0.15) | 261.25(252.95,269.77) | 254.92(244.76,265.40) | 0.04(-0.06,0.15) |
| Algeria | both | 131.76(129.52,134.03) | 109.97(108.37,111.59) | -0.51(-0.54,-0.48) | 131.76(129.52,134.03) | 109.97(108.37,111.59) | -0.51(-0.54,-0.48) |
| American Samoa | both | 81.64(47.14,133.76) | 71.13(36.95,125.15) | -0.44(-0.48,-0.40) | 81.64(47.14,133.76) | 71.13(36.95,125.15) | -0.44(-0.48,-0.40) |
| Andorra | both | 481.94(395.68,582.77) | 379.30(304.37,468.23) | -0.72(-0.75,-0.69) | 481.94(395.68,582.77) | 379.30(304.37,468.23) | -0.72(-0.75,-0.69) |
| Angola | both | 55.77(53.46,58.17) | 57.00(55.67,58.37) | 0.33(0.21,0.46) | 55.77(53.46,58.17) | 57.00(55.67,58.37) | 0.33(0.21,0.46) |
| Antigua and Barbuda | both | 183.47(135.14,244.34) | 113.67(80.54,156.42) | -1.77(-1.88,-1.67) | 183.47(135.14,244.34) | 113.67(80.54,156.42) | -1.77(-1.88,-1.67) |
| Argentina | both | 344.19(340.92,347.48) | 317.27(314.62,319.94) | -0.12(-0.27,0.04) | 344.19(340.92,347.48) | 317.27(314.62,319.94) | -0.12(-0.27,0.04) |
| Armenia | both | 77.20(72.69,81.92) | 96.96(90.86,103.38) | 1.25(0.97,1.54) | 77.20(72.69,81.92) | 96.96(90.86,103.38) | 1.25(0.97,1.54) |
| Australia | both | 291.87(287.76,296.03) | 170.77(167.94,173.65) | -1.65(-1.74,-1.57) | 291.87(287.76,296.03) | 170.77(167.94,173.65) | -1.65(-1.74,-1.57) |
| Austria | both | 469.26(461.31,477.33) | 259.84(253.63,266.17) | -1.91(-1.97,-1.84) | 469.26(461.31,477.33) | 259.84(253.63,266.17) | -1.91(-1.97,-1.84) |
| Azerbaijan | both | 88.97(85.70,92.33) | 72.88(70.22,75.62) | -0.62(-0.70,-0.53) | 88.97(85.70,92.33) | 72.88(70.22,75.62) | -0.62(-0.70,-0.53) |
| Bahamas | both | 240.55(213.49,270.34) | 177.27(156.87,199.60) | -1.01(-1.06,-0.96) | 240.55(213.49,270.34) | 177.27(156.87,199.60) | -1.01(-1.06,-0.96) |
| Bahrain | both | 128.61(114.49,144.18) | 107.49(99.64,115.85) | -0.60(-0.65,-0.56) | 128.61(114.49,144.18) | 107.49(99.64,115.85) | -0.60(-0.65,-0.56) |
| Bangladesh | both | 78.66(77.81,79.51) | 59.44(58.87,60.02) | -0.76(-0.85,-0.67) | 78.66(77.81,79.51) | 59.44(58.87,60.02) | -0.76(-0.85,-0.67) |
| Barbados | both | 177.22(153.09,204.13) | 136.53(114.22,162.04) | -1.03(-1.20,-0.87) | 177.22(153.09,204.13) | 136.53(114.22,162.04) | -1.03(-1.20,-0.87) |
| Belarus | both | 297.07(291.60,302.62) | 192.55(187.23,198.00) | -1.49(-1.70,-1.28) | 297.07(291.60,302.62) | 192.55(187.23,198.00) | -1.49(-1.70,-1.28) |
| Belgium | both | 462.49(455.38,469.69) | 312.91(306.92,319.00) | -1.55(-1.70,-1.40) | 462.49(455.38,469.69) | 312.91(306.92,319.00) | -1.55(-1.70,-1.40) |
| Belize | both | 456.86(409.11,509.38) | 327.26(302.04,354.12) | -1.62(-1.86,-1.39) | 456.86(409.11,509.38) | 327.26(302.04,354.12) | -1.62(-1.86,-1.39) |
| Benin | both | 49.64(46.33,53.13) | 38.84(37.15,40.59) | -1.17(-1.32,-1.02) | 49.64(46.33,53.13) | 38.84(37.15,40.59) | -1.17(-1.32,-1.02) |
| Bermuda | both | 258.38(197.86,333.28) | 144.69(92.16,217.75) | -1.91(-1.97,-1.86) | 258.38(197.86,333.28) | 144.69(92.16,217.75) | -1.91(-1.97,-1.86) |
| Bhutan | both | 97.51(85.81,110.57) | 74.78(65.92,84.55) | -0.90(-0.98,-0.82) | 97.51(85.81,110.57) | 74.78(65.92,84.55) | -0.90(-0.98,-0.82) |
| Bolivia (Plurinational State of) | both | 95.38(91.55,99.33) | 85.33(82.77,87.95) | -0.20(-0.35,-0.04) | 95.38(91.55,99.33) | 85.33(82.77,87.95) | -0.20(-0.35,-0.04) |
| Bosnia and Herzegovina | both | 222.19(215.51,229.03) | 215.92(206.66,225.50) | 0.11(0.00,0.23) | 222.19(215.51,229.03) | 215.92(206.66,225.50) | 0.11(0.00,0.23) |
| Botswana | both | 55.25(48.94,62.24) | 62.82(58.12,67.80) | 0.27(0.02,0.52) | 55.25(48.94,62.24) | 62.82(58.12,67.80) | 0.27(0.02,0.52) |
| Brazil | both | 200.43(199.32,201.54) | 198.19(197.24,199.16) | -0.08(-0.32,0.17) | 200.43(199.32,201.54) | 198.19(197.24,199.16) | -0.08(-0.32,0.17) |
| Brunei Darussalam | both | 693.85(647.75,742.44) | 448.61(419.31,479.55) | -1.51(-1.78,-1.23) | 693.85(647.75,742.44) | 448.61(419.31,479.55) | -1.51(-1.78,-1.23) |
| Bulgaria | both | 355.76(348.95,362.68) | 302.01(293.77,310.44) | -0.55(-0.66,-0.43) | 355.76(348.95,362.68) | 302.01(293.77,310.44) | -0.55(-0.66,-0.43) |
| Burkina Faso | both | 45.18(42.85,47.63) | 55.98(54.39,57.60) | 0.86(0.80,0.91) | 45.18(42.85,47.63) | 55.98(54.39,57.60) | 0.86(0.80,0.91) |
| Burundi | both | 86.55(82.58,90.68) | 65.59(63.42,67.81) | -1.09(-1.15,-1.04) | 86.55(82.58,90.68) | 65.59(63.42,67.81) | -1.09(-1.15,-1.04) |
| Cabo Verde | both | 39.42(29.00,53.07) | 41.46(33.85,50.32) | 0.16(-0.11,0.44) | 39.42(29.00,53.07) | 41.46(33.85,50.32) | 0.16(-0.11,0.44) |
| Cambodia | both | 125.45(121.94,129.04) | 116.96(114.47,119.48) | -0.31(-0.34,-0.28) | 125.45(121.94,129.04) | 116.96(114.47,119.48) | -0.31(-0.34,-0.28) |
| Cameroon | both | 53.45(51.11,55.87) | 58.22(56.90,59.57) | 0.23(0.16,0.29) | 53.45(51.11,55.87) | 58.22(56.90,59.57) | 0.23(0.16,0.29) |
| Canada | both | 327.29(323.76,330.84) | 209.01(206.33,211.73) | -1.29(-1.37,-1.22) | 327.29(323.76,330.84) | 209.01(206.33,211.73) | -1.29(-1.37,-1.22) |
| Central African Republic | both | 48.93(44.76,53.42) | 48.63(45.75,51.66) | 0.00(-0.05,0.04) | 48.93(44.76,53.42) | 48.63(45.75,51.66) | 0.00(-0.05,0.04) |
| Chad | both | 34.87(32.37,37.53) | 40.84(39.24,42.49) | 0.56(0.43,0.69) | 34.87(32.37,37.53) | 40.84(39.24,42.49) | 0.56(0.43,0.69) |
| Chile | both | 257.69(253.56,261.88) | 347.97(343.57,352.42) | 0.41(0.17,0.65) | 257.69(253.56,261.88) | 347.97(343.57,352.42) | 0.41(0.17,0.65) |
| China | both | 286.17(285.72,286.62) | 302.79(302.28,303.31) | -0.07(-0.30,0.16) | 286.17(285.72,286.62) | 302.79(302.28,303.31) | -0.07(-0.30,0.16) |
| Colombia | both | 149.17(147.17,151.20) | 184.45(182.57,186.35) | 0.23(-0.06,0.53) | 149.17(147.17,151.20) | 184.45(182.57,186.35) | 0.23(-0.06,0.53) |
| Comoros | both | 68.13(56.27,82.08) | 55.43(47.47,64.40) | -0.87(-0.99,-0.75) | 68.13(56.27,82.08) | 55.43(47.47,64.40) | -0.87(-0.99,-0.75) |
| Congo | both | 47.78(43.44,52.49) | 45.52(42.77,48.42) | -0.21(-0.26,-0.15) | 47.78(43.44,52.49) | 45.52(42.77,48.42) | -0.21(-0.26,-0.15) |
| Cook Islands | both | 104.69(45.18,211.55) | 114.91(45.18,242.53) | 0.43(0.38,0.47) | 104.69(45.18,211.55) | 114.91(45.18,242.53) | 0.43(0.38,0.47) |
| Costa Rica | both | 212.12(204.25,220.24) | 158.03(152.37,163.86) | -1.42(-1.60,-1.24) | 212.12(204.25,220.24) | 158.03(152.37,163.86) | -1.42(-1.60,-1.24) |
| Croatia | both | 367.54(358.57,376.69) | 290.53(280.78,300.55) | -0.59(-1.07,-0.12) | 367.54(358.57,376.69) | 290.53(280.78,300.55) | -0.59(-1.07,-0.12) |
| Cuba | both | 572.86(566.17,579.62) | 179.40(174.98,183.90) | -4.49(-4.81,-4.16) | 572.86(566.17,579.62) | 179.40(174.98,183.90) | -4.49(-4.81,-4.16) |
| Cyprus | both | 521.30(495.82,547.78) | 356.56(338.49,375.43) | -1.53(-1.72,-1.34) | 521.30(495.82,547.78) | 356.56(338.49,375.43) | -1.53(-1.72,-1.34) |
| Czechia | both | 457.07(450.13,464.10) | 266.61(260.41,272.93) | -2.18(-2.55,-1.80) | 457.07(450.13,464.10) | 266.61(260.41,272.93) | -2.18(-2.55,-1.80) |
| Côte d'Ivoire | both | 46.83(44.88,48.85) | 45.79(44.55,47.06) | -0.30(-0.40,-0.21) | 46.83(44.88,48.85) | 45.79(44.55,47.06) | -0.30(-0.40,-0.21) |
| Democratic People's Republic of Korea | both | 223.67(220.46,226.92) | 284.57(281.25,287.91) | 0.79(0.72,0.86) | 223.67(220.46,226.92) | 284.57(281.25,287.91) | 0.79(0.72,0.86) |
| Democratic Republic of the Congo | both | 58.17(56.92,59.43) | 47.76(47.05,48.48) | -0.90(-0.98,-0.81) | 58.17(56.92,59.43) | 47.76(47.05,48.48) | -0.90(-0.98,-0.81) |
| Denmark | both | 589.51(578.46,600.74) | 258.20(250.75,265.83) | -2.95(-3.07,-2.83) | 589.51(578.46,600.74) | 258.20(250.75,265.83) | -2.95(-3.07,-2.83) |
| Djibouti | both | 58.51(47.56,71.53) | 53.60(47.60,60.16) | -0.46(-0.56,-0.36) | 58.51(47.56,71.53) | 53.60(47.60,60.16) | -0.46(-0.56,-0.36) |
| Dominica | both | 244.40(191.10,309.40) | 193.32(143.46,255.02) | -1.09(-1.36,-0.83) | 244.40(191.10,309.40) | 193.32(143.46,255.02) | -1.09(-1.36,-0.83) |
| Dominican Republic | both | 186.37(181.58,191.26) | 187.18(183.22,191.20) | 0.01(-0.20,0.23) | 186.37(181.58,191.26) | 187.18(183.22,191.20) | 0.01(-0.20,0.23) |
| Ecuador | both | 127.26(123.82,130.77) | 178.39(175.35,181.48) | 1.61(1.36,1.85) | 127.26(123.82,130.77) | 178.39(175.35,181.48) | 1.61(1.36,1.85) |
| Egypt | both | 105.32(103.97,106.68) | 106.99(106.00,107.98) | 0.22(0.11,0.33) | 105.32(103.97,106.68) | 106.99(106.00,107.98) | 0.22(0.11,0.33) |
| El Salvador | both | 129.07(124.25,134.04) | 116.64(112.52,120.88) | -0.47(-0.66,-0.28) | 129.07(124.25,134.04) | 116.64(112.52,120.88) | -0.47(-0.66,-0.28) |
| Equatorial Guinea | both | 44.38(34.38,56.61) | 55.13(49.75,61.00) | 1.12(0.79,1.46) | 44.38(34.38,56.61) | 55.13(49.75,61.00) | 1.12(0.79,1.46) |
| Eritrea | both | 65.36(60.98,69.99) | 66.15(63.16,69.25) | -0.10(-0.26,0.07) | 65.36(60.98,69.99) | 66.15(63.16,69.25) | -0.10(-0.26,0.07) |
| Estonia | both | 328.29(313.33,343.80) | 145.99(133.68,159.18) | -3.11(-3.33,-2.89) | 328.29(313.33,343.80) | 145.99(133.68,159.18) | -3.11(-3.33,-2.89) |
| Eswatini | both | 62.19(53.40,72.18) | 73.04(65.79,80.92) | 0.51(0.22,0.80) | 62.19(53.40,72.18) | 73.04(65.79,80.92) | 0.51(0.22,0.80) |
| Ethiopia | both | 58.55(57.44,59.68) | 36.97(36.41,37.54) | -1.57(-1.64,-1.49) | 58.55(57.44,59.68) | 36.97(36.41,37.54) | -1.57(-1.64,-1.49) |
| Fiji | both | 72.19(63.23,82.12) | 63.84(55.80,72.71) | -0.36(-0.41,-0.30) | 72.19(63.23,82.12) | 63.84(55.80,72.71) | -0.36(-0.41,-0.30) |
| Finland | both | 409.51(399.79,419.42) | 230.65(223.16,238.34) | -1.99(-2.25,-1.72) | 409.51(399.79,419.42) | 230.65(223.16,238.34) | -1.99(-2.25,-1.72) |
| France | both | 432.15(429.38,434.94) | 313.68(311.20,316.18) | -1.05(-1.22,-0.87) | 432.15(429.38,434.94) | 313.68(311.20,316.18) | -1.05(-1.22,-0.87) |
| Gabon | both | 56.26(49.00,64.38) | 55.30(50.12,60.91) | -0.09(-0.14,-0.05) | 56.26(49.00,64.38) | 55.30(50.12,60.91) | -0.09(-0.14,-0.05) |
| Gambia | both | 38.18(32.12,45.19) | 36.83(33.13,40.87) | -0.31(-0.40,-0.23) | 38.18(32.12,45.19) | 36.83(33.13,40.87) | -0.31(-0.40,-0.23) |
| Georgia | both | 114.54(110.02,119.21) | 104.55(98.38,111.01) | -0.33(-0.49,-0.17) | 114.54(110.02,119.21) | 104.55(98.38,111.01) | -0.33(-0.49,-0.17) |
| Germany | both | 468.96(466.34,471.60) | 263.10(260.99,265.21) | -2.15(-2.26,-2.04) | 468.96(466.34,471.60) | 263.10(260.99,265.21) | -2.15(-2.26,-2.04) |
| Ghana | both | 57.90(55.93,59.93) | 69.64(68.28,71.02) | 0.67(0.60,0.73) | 57.90(55.93,59.93) | 69.64(68.28,71.02) | 0.67(0.60,0.73) |
| Greece | both | 389.86(383.55,396.25) | 340.61(333.55,347.79) | -0.44(-0.60,-0.28) | 389.86(383.55,396.25) | 340.61(333.55,347.79) | -0.44(-0.60,-0.28) |
| Greenland | both | 220.38(164.61,291.31) | 138.59(91.10,203.30) | -1.63(-1.70,-1.55) | 220.38(164.61,291.31) | 138.59(91.10,203.30) | -1.63(-1.70,-1.55) |
| Grenada | both | 209.04(162.72,265.47) | 142.18(107.81,184.50) | -1.24(-1.29,-1.20) | 209.04(162.72,265.47) | 142.18(107.81,184.50) | -1.24(-1.29,-1.20) |
| Guam | both | 96.46(73.77,124.15) | 80.90(58.98,108.37) | -0.60(-0.65,-0.54) | 96.46(73.77,124.15) | 80.90(58.98,108.37) | -0.60(-0.65,-0.54) |
| Guatemala | both | 123.15(119.17,127.24) | 136.91(134.14,139.72) | 0.47(0.39,0.56) | 123.15(119.17,127.24) | 136.91(134.14,139.72) | 0.47(0.39,0.56) |
| Guinea | both | 41.83(39.08,44.74) | 46.36(44.51,48.27) | 0.29(0.19,0.40) | 41.83(39.08,44.74) | 46.36(44.51,48.27) | 0.29(0.19,0.40) |
| Guinea-Bissau | both | 68.02(59.80,77.16) | 66.97(61.53,72.79) | -0.12(-0.24,0.01) | 68.02(59.80,77.16) | 66.97(61.53,72.79) | -0.12(-0.24,0.01) |
| Guyana | both | 259.05(242.17,276.88) | 276.57(258.42,295.73) | 0.08(-0.09,0.25) | 259.05(242.17,276.88) | 276.57(258.42,295.73) | 0.08(-0.09,0.25) |
| Haiti | both | 237.75(231.70,243.93) | 208.59(204.79,212.44) | -0.28(-0.46,-0.09) | 237.75(231.70,243.93) | 208.59(204.79,212.44) | -0.28(-0.46,-0.09) |
| Honduras | both | 130.74(125.37,136.29) | 115.98(112.82,119.22) | -0.67(-0.93,-0.40) | 130.74(125.37,136.29) | 115.98(112.82,119.22) | -0.67(-0.93,-0.40) |
| Hungary | both | 545.96(538.32,553.68) | 237.93(232.05,243.94) | -3.10(-3.25,-2.95) | 545.96(538.32,553.68) | 237.93(232.05,243.94) | -3.10(-3.25,-2.95) |
| Iceland | both | 342.27(307.36,380.13) | 199.55(174.45,227.41) | -1.88(-2.01,-1.75) | 342.27(307.36,380.13) | 199.55(174.45,227.41) | -1.88(-2.01,-1.75) |
| India | both | 119.45(119.09,119.82) | 131.04(130.75,131.33) | 0.21(0.05,0.38) | 119.45(119.09,119.82) | 131.04(130.75,131.33) | 0.21(0.05,0.38) |
| Indonesia | both | 137.32(136.51,138.14) | 99.98(99.39,100.56) | -1.16(-1.33,-0.99) | 137.32(136.51,138.14) | 99.98(99.39,100.56) | -1.16(-1.33,-0.99) |
| Iran (Islamic Republic of) | both | 166.40(164.68,168.13) | 168.57(167.13,170.02) | -0.03(-0.11,0.06) | 166.40(164.68,168.13) | 168.57(167.13,170.02) | -0.03(-0.11,0.06) |
| Iraq | both | 95.82(93.55,98.14) | 85.87(84.51,87.26) | -0.28(-0.36,-0.20) | 95.82(93.55,98.14) | 85.87(84.51,87.26) | -0.28(-0.36,-0.20) |
| Ireland | both | 533.97(522.00,546.15) | 217.48(210.10,225.07) | -2.98(-3.05,-2.92) | 533.97(522.00,546.15) | 217.48(210.10,225.07) | -2.98(-3.05,-2.92) |
| Israel | both | 304.58(296.85,312.46) | 244.00(238.72,249.37) | -0.74(-0.85,-0.63) | 304.58(296.85,312.46) | 244.00(238.72,249.37) | -0.74(-0.85,-0.63) |
| Italy | both | 543.94(540.80,547.09) | 296.97(294.21,299.74) | -2.40(-2.60,-2.20) | 543.94(540.80,547.09) | 296.97(294.21,299.74) | -2.40(-2.60,-2.20) |
| Jamaica | both | 121.75(114.96,128.88) | 100.36(94.74,106.23) | -0.53(-0.60,-0.45) | 121.75(114.96,128.88) | 100.36(94.74,106.23) | -0.53(-0.60,-0.45) |
| Japan | both | 484.91(482.87,486.95) | 209.39(207.77,211.02) | -3.08(-3.20,-2.95) | 484.91(482.87,486.95) | 209.39(207.77,211.02) | -3.08(-3.20,-2.95) |
| Jordan | both | 99.61(94.56,104.90) | 80.43(78.05,82.86) | -0.71(-0.80,-0.63) | 99.61(94.56,104.90) | 80.43(78.05,82.86) | -0.71(-0.80,-0.63) |
| Kazakhstan | both | 116.96(114.40,119.57) | 104.58(102.12,107.08) | -0.16(-0.31,-0.01) | 116.96(114.40,119.57) | 104.58(102.12,107.08) | -0.16(-0.31,-0.01) |
| Kenya | both | 55.04(53.45,56.68) | 50.41(49.46,51.38) | -0.51(-0.73,-0.28) | 55.04(53.45,56.68) | 50.41(49.46,51.38) | -0.51(-0.73,-0.28) |
| Kiribati | both | 77.11(49.10,116.59) | 79.68(56.83,108.90) | 0.01(-0.24,0.26) | 77.11(49.10,116.59) | 79.68(56.83,108.90) | 0.01(-0.24,0.26) |
| Kuwait | both | 141.49(133.35,150.05) | 96.51(91.85,101.37) | -1.40(-1.57,-1.23) | 141.49(133.35,150.05) | 96.51(91.85,101.37) | -1.40(-1.57,-1.23) |
| Kyrgyzstan | both | 107.10(102.38,111.99) | 86.30(82.82,89.88) | -0.50(-0.61,-0.39) | 107.10(102.38,111.99) | 86.30(82.82,89.88) | -0.50(-0.61,-0.39) |
| Lao People's Democratic Republic | both | 136.51(130.75,142.48) | 114.47(110.80,118.24) | -0.57(-0.61,-0.53) | 136.51(130.75,142.48) | 114.47(110.80,118.24) | -0.57(-0.61,-0.53) |
| Latvia | both | 410.75(397.83,424.00) | 207.81(195.22,221.05) | -2.60(-2.77,-2.43) | 410.75(397.83,424.00) | 207.81(195.22,221.05) | -2.60(-2.77,-2.43) |
| Lebanon | both | 117.62(111.44,124.08) | 92.44(88.41,96.62) | -0.91(-1.00,-0.82) | 117.62(111.44,124.08) | 92.44(88.41,96.62) | -0.91(-1.00,-0.82) |
| Lesotho | both | 49.71(43.90,56.12) | 62.60(57.30,68.29) | 0.75(0.63,0.87) | 49.71(43.90,56.12) | 62.60(57.30,68.29) | 0.75(0.63,0.87) |
| Liberia | both | 41.07(37.03,45.46) | 37.19(34.70,39.83) | -0.51(-0.60,-0.41) | 41.07(37.03,45.46) | 37.19(34.70,39.83) | -0.51(-0.60,-0.41) |
| Libya | both | 137.37(131.76,143.19) | 102.62(98.99,106.34) | -0.72(-0.84,-0.60) | 137.37(131.76,143.19) | 102.62(98.99,106.34) | -0.72(-0.84,-0.60) |
| Lithuania | both | 459.16(447.91,470.64) | 205.03(194.96,215.53) | -2.92(-3.34,-2.51) | 459.16(447.91,470.64) | 205.03(194.96,215.53) | -2.92(-3.34,-2.51) |
| Luxembourg | both | 437.30(402.22,474.87) | 286.34(263.10,311.22) | -1.48(-1.54,-1.41) | 437.30(402.22,474.87) | 286.34(263.10,311.22) | -1.48(-1.54,-1.41) |
| Madagascar | both | 67.17(64.79,69.62) | 54.45(53.11,55.82) | -0.62(-0.71,-0.52) | 67.17(64.79,69.62) | 54.45(53.11,55.82) | -0.62(-0.71,-0.52) |
| Malawi | both | 57.17(54.74,59.69) | 54.00(52.41,55.64) | -0.30(-0.45,-0.14) | 57.17(54.74,59.69) | 54.00(52.41,55.64) | -0.30(-0.45,-0.14) |
| Malaysia | both | 156.81(153.99,159.67) | 138.97(137.00,140.97) | -0.52(-0.60,-0.43) | 156.81(153.99,159.67) | 138.97(137.00,140.97) | -0.52(-0.60,-0.43) |
| Maldives | both | 106.15(84.53,132.60) | 63.40(53.01,75.52) | -1.67(-1.80,-1.55) | 106.15(84.53,132.60) | 63.40(53.01,75.52) | -1.67(-1.80,-1.55) |
| Mali | both | 35.40(33.29,37.62) | 34.85(33.60,36.13) | -0.12(-0.24,0.00) | 35.40(33.29,37.62) | 34.85(33.60,36.13) | -0.12(-0.24,0.00) |
| Malta | both | 317.14(287.47,349.16) | 243.23(215.76,273.50) | -0.85(-1.01,-0.69) | 317.14(287.47,349.16) | 243.23(215.76,273.50) | -0.85(-1.01,-0.69) |
| Marshall Islands | both | 65.40(32.73,119.36) | 60.98(33.69,101.93) | -0.29(-0.35,-0.24) | 65.40(32.73,119.36) | 60.98(33.69,101.93) | -0.29(-0.35,-0.24) |
| Mauritania | both | 69.72(63.89,75.99) | 56.73(53.17,60.48) | -0.69(-0.74,-0.64) | 69.72(63.89,75.99) | 56.73(53.17,60.48) | -0.69(-0.74,-0.64) |
| Mauritius | both | 77.08(69.54,85.23) | 84.90(76.60,93.87) | 0.38(0.31,0.45) | 77.08(69.54,85.23) | 84.90(76.60,93.87) | 0.38(0.31,0.45) |
| Mexico | both | 227.18(225.60,228.76) | 166.67(165.56,167.79) | -0.30(-0.64,0.04) | 227.18(225.60,228.76) | 166.67(165.56,167.79) | -0.30(-0.64,0.04) |
| Micronesia (Federated States of) | both | 82.62(56.66,117.16) | 85.01(59.49,118.35) | 0.05(0.01,0.09) | 82.62(56.66,117.16) | 85.01(59.49,118.35) | 0.05(0.01,0.09) |
| Monaco | both | 374.84(253.71,539.95) | 292.90(191.68,430.07) | -0.72(-0.76,-0.68) | 374.84(253.71,539.95) | 292.90(191.68,430.07) | -0.72(-0.76,-0.68) |
| Mongolia | both | 87.66(81.57,94.16) | 94.76(89.33,100.44) | 0.53(0.42,0.63) | 87.66(81.57,94.16) | 94.76(89.33,100.44) | 0.53(0.42,0.63) |
| Montenegro | both | 341.72(319.18,365.45) | 276.62(253.97,300.80) | -0.80(-0.94,-0.65) | 341.72(319.18,365.45) | 276.62(253.97,300.80) | -0.80(-0.94,-0.65) |
| Morocco | both | 104.08(102.13,106.06) | 95.49(93.91,97.09) | -0.30(-0.38,-0.21) | 104.08(102.13,106.06) | 95.49(93.91,97.09) | -0.30(-0.38,-0.21) |
| Mozambique | both | 63.34(61.09,65.66) | 73.73(72.18,75.31) | 0.63(0.54,0.71) | 63.34(61.09,65.66) | 73.73(72.18,75.31) | 0.63(0.54,0.71) |
| Myanmar | both | 125.09(123.43,126.78) | 96.74(95.46,98.03) | -0.95(-1.00,-0.89) | 125.09(123.43,126.78) | 96.74(95.46,98.03) | -0.95(-1.00,-0.89) |
| Namibia | both | 59.05(52.74,65.99) | 63.17(58.44,68.20) | 0.12(-0.06,0.29) | 59.05(52.74,65.99) | 63.17(58.44,68.20) | 0.12(-0.06,0.29) |
| Nauru | both | 115.04(35.30,284.18) | 127.79(46.41,285.55) | 0.27(0.23,0.31) | 115.04(35.30,284.18) | 127.79(46.41,285.55) | 0.27(0.23,0.31) |
| Nepal | both | 93.26(91.04,95.53) | 65.27(63.91,66.65) | -1.26(-1.30,-1.21) | 93.26(91.04,95.53) | 65.27(63.91,66.65) | -1.26(-1.30,-1.21) |
| Netherlands | both | 603.12(596.71,609.58) | 243.57(239.30,247.90) | -3.14(-3.24,-3.03) | 603.12(596.71,609.58) | 243.57(239.30,247.90) | -3.14(-3.24,-3.03) |
| New Zealand | both | 486.60(475.01,498.41) | 242.71(235.41,250.18) | -3.99(-4.73,-3.24) | 486.60(475.01,498.41) | 242.71(235.41,250.18) | -3.99(-4.73,-3.24) |
| Nicaragua | both | 106.78(101.55,112.25) | 111.11(107.28,115.05) | 0.27(0.16,0.37) | 106.78(101.55,112.25) | 111.11(107.28,115.05) | 0.27(0.16,0.37) |
| Niger | both | 39.50(37.18,41.95) | 41.76(40.39,43.17) | 0.34(0.25,0.44) | 39.50(37.18,41.95) | 41.76(40.39,43.17) | 0.34(0.25,0.44) |
| Nigeria | both | 44.71(44.00,45.44) | 39.60(39.18,40.02) | -0.35(-0.51,-0.19) | 44.71(44.00,45.44) | 39.60(39.18,40.02) | -0.35(-0.51,-0.19) |
| Niue | both | 80.23(0.33,664.53) | 75.94(0.03,847.03) | -0.31(-0.41,-0.22) | 80.23(0.33,664.53) | 75.94(0.03,847.03) | -0.31(-0.41,-0.22) |
| North Macedonia | both | 281.37(269.80,293.30) | 216.62(205.82,227.88) | -0.97(-1.03,-0.91) | 281.37(269.80,293.30) | 216.62(205.82,227.88) | -0.97(-1.03,-0.91) |
| Northern Mariana Islands | both | 104.25(66.80,156.69) | 79.99(42.68,138.11) | -1.05(-1.17,-0.93) | 104.25(66.80,156.69) | 79.99(42.68,138.11) | -1.05(-1.17,-0.93) |
| Norway | both | 388.14(378.49,397.97) | 200.99(194.31,207.86) | -2.21(-2.35,-2.07) | 388.14(378.49,397.97) | 200.99(194.31,207.86) | -2.21(-2.35,-2.07) |
| Oman | both | 151.89(143.52,160.64) | 190.84(184.35,197.52) | 1.15(0.90,1.40) | 151.89(143.52,160.64) | 190.84(184.35,197.52) | 1.15(0.90,1.40) |
| Pakistan | both | 86.97(86.05,87.90) | 93.11(92.51,93.71) | 0.09(-0.09,0.28) | 86.97(86.05,87.90) | 93.11(92.51,93.71) | 0.09(-0.09,0.28) |
| Palau | both | 96.38(37.88,204.46) | 105.58(39.18,231.11) | 0.28(0.22,0.33) | 96.38(37.88,204.46) | 105.58(39.18,231.11) | 0.28(0.22,0.33) |
| Palestine | both | 80.23(73.92,87.03) | 65.42(62.08,68.92) | -0.86(-0.94,-0.78) | 80.23(73.92,87.03) | 65.42(62.08,68.92) | -0.86(-0.94,-0.78) |
| Panama | both | 246.76(237.26,256.57) | 163.67(157.56,169.95) | -1.65(-1.80,-1.51) | 246.76(237.26,256.57) | 163.67(157.56,169.95) | -1.65(-1.80,-1.51) |
| Papua New Guinea | both | 69.62(65.64,73.80) | 56.62(54.39,58.92) | -0.96(-1.08,-0.85) | 69.62(65.64,73.80) | 56.62(54.39,58.92) | -0.96(-1.08,-0.85) |
| Paraguay | both | 143.19(137.35,149.23) | 150.62(146.30,155.03) | 0.30(0.18,0.43) | 143.19(137.35,149.23) | 150.62(146.30,155.03) | 0.30(0.18,0.43) |
| Peru | both | 102.18(100.08,104.33) | 111.54(109.85,113.25) | 0.62(0.51,0.73) | 102.18(100.08,104.33) | 111.54(109.85,113.25) | 0.62(0.51,0.73) |
| Philippines | both | 99.13(97.91,100.35) | 74.78(74.01,75.57) | -0.93(-1.01,-0.86) | 99.13(97.91,100.35) | 74.78(74.01,75.57) | -0.93(-1.01,-0.86) |
| Poland | both | 444.09(440.56,447.64) | 305.55(302.23,308.90) | -1.71(-2.02,-1.40) | 444.09(440.56,447.64) | 305.55(302.23,308.90) | -1.71(-2.02,-1.40) |
| Portugal | both | 599.96(592.20,607.79) | 287.32(281.11,293.63) | -2.60(-2.70,-2.50) | 599.96(592.20,607.79) | 287.32(281.11,293.63) | -2.60(-2.70,-2.50) |
| Puerto Rico | both | 233.80(225.94,241.87) | 179.22(171.11,187.63) | -0.88(-0.94,-0.83) | 233.80(225.94,241.87) | 179.22(171.11,187.63) | -0.88(-0.94,-0.83) |
| Qatar | both | 231.32(210.79,253.64) | 138.28(130.96,146.02) | -1.63(-1.78,-1.48) | 231.32(210.79,253.64) | 138.28(130.96,146.02) | -1.63(-1.78,-1.48) |
| Republic of Korea | both | 776.24(772.47,780.02) | 265.61(262.96,268.28) | -4.23(-4.58,-3.88) | 776.24(772.47,780.02) | 265.61(262.96,268.28) | -4.23(-4.58,-3.88) |
| Republic of Moldova | both | 292.68(284.59,300.95) | 169.50(161.74,177.57) | -1.84(-1.98,-1.69) | 292.68(284.59,300.95) | 169.50(161.74,177.57) | -1.84(-1.98,-1.69) |
| Romania | both | 362.08(358.06,366.14) | 319.27(314.37,324.22) | -0.38(-0.43,-0.34) | 362.08(358.06,366.14) | 319.27(314.37,324.22) | -0.38(-0.43,-0.34) |
| Russian Federation | both | 280.09(278.69,281.49) | 207.73(206.34,209.13) | -0.88(-1.09,-0.67) | 280.09(278.69,281.49) | 207.73(206.34,209.13) | -0.88(-1.09,-0.67) |
| Rwanda | both | 147.57(143.07,152.18) | 92.52(90.04,95.05) | -2.08(-2.28,-1.89) | 147.57(143.07,152.18) | 92.52(90.04,95.05) | -2.08(-2.28,-1.89) |
| Saint Kitts and Nevis | both | 231.29(165.18,317.04) | 150.34(103.41,212.13) | -1.51(-1.63,-1.39) | 231.29(165.18,317.04) | 150.34(103.41,212.13) | -1.51(-1.63,-1.39) |
| Saint Lucia | both | 263.16(222.31,310.24) | 184.10(152.53,220.56) | -1.40(-1.51,-1.28) | 263.16(222.31,310.24) | 184.10(152.53,220.56) | -1.40(-1.51,-1.28) |
| Saint Vincent and the Grenadines | both | 201.43(162.17,248.61) | 135.30(102.15,175.92) | -1.50(-1.63,-1.37) | 201.43(162.17,248.61) | 135.30(102.15,175.92) | -1.50(-1.63,-1.37) |
| Samoa | both | 80.06(59.62,106.19) | 85.41(66.32,108.63) | 0.19(0.14,0.24) | 80.06(59.62,106.19) | 85.41(66.32,108.63) | 0.19(0.14,0.24) |
| San Marino | both | 490.07(357.96,656.07) | 342.54(231.43,489.22) | -1.12(-1.18,-1.07) | 490.07(357.96,656.07) | 342.54(231.43,489.22) | -1.12(-1.18,-1.07) |
| Sao Tome and Principe | both | 37.56(20.99,64.04) | 38.82(27.06,54.18) | -0.16(-0.28,-0.03) | 37.56(20.99,64.04) | 38.82(27.06,54.18) | -0.16(-0.28,-0.03) |
| Saudi Arabia | both | 130.34(127.61,133.12) | 124.93(123.24,126.65) | 0.17(0.06,0.29) | 130.34(127.61,133.12) | 124.93(123.24,126.65) | 0.17(0.06,0.29) |
| Senegal | both | 39.06(36.74,41.52) | 38.32(36.82,39.88) | -0.16(-0.25,-0.06) | 39.06(36.74,41.52) | 38.32(36.82,39.88) | -0.16(-0.25,-0.06) |
| Serbia | both | 490.75(483.45,498.13) | 284.52(278.37,290.79) | -1.86(-1.94,-1.77) | 490.75(483.45,498.13) | 284.52(278.37,290.79) | -1.86(-1.94,-1.77) |
| Seychelles | both | 106.99(73.68,151.87) | 129.67(95.78,172.20) | 0.53(0.46,0.60) | 106.99(73.68,151.87) | 129.67(95.78,172.20) | 0.53(0.46,0.60) |
| Sierra Leone | both | 44.88(41.64,48.32) | 44.23(42.10,46.45) | -0.02(-0.11,0.06) | 44.88(41.64,48.32) | 44.23(42.10,46.45) | -0.02(-0.11,0.06) |
| Singapore | both | 356.64(347.01,366.48) | 187.17(180.21,194.35) | -2.29(-2.37,-2.21) | 356.64(347.01,366.48) | 187.17(180.21,194.35) | -2.29(-2.37,-2.21) |
| Slovakia | both | 502.76(492.96,512.71) | 279.20(270.85,287.76) | -2.31(-2.47,-2.15) | 502.76(492.96,512.71) | 279.20(270.85,287.76) | -2.31(-2.47,-2.15) |
| Slovenia | both | 543.87(527.09,561.06) | 258.85(244.95,273.36) | -2.40(-2.70,-2.10) | 543.87(527.09,561.06) | 258.85(244.95,273.36) | -2.40(-2.70,-2.10) |
| Solomon Islands | both | 74.93(60.27,92.51) | 85.68(74.98,97.55) | 0.41(0.37,0.45) | 74.93(60.27,92.51) | 85.68(74.98,97.55) | 0.41(0.37,0.45) |
| Somalia | both | 62.61(59.76,65.57) | 57.24(55.60,58.92) | -0.36(-0.42,-0.31) | 62.61(59.76,65.57) | 57.24(55.60,58.92) | -0.36(-0.42,-0.31) |
| South Africa | both | 102.72(101.12,104.35) | 79.72(78.60,80.85) | -0.90(-1.05,-0.75) | 102.72(101.12,104.35) | 79.72(78.60,80.85) | -0.90(-1.05,-0.75) |
| South Sudan | both | 59.16(56.01,62.47) | 44.29(42.09,46.59) | -0.99(-1.12,-0.86) | 59.16(56.01,62.47) | 44.29(42.09,46.59) | -0.99(-1.12,-0.86) |
| Spain | both | 422.97(419.68,426.29) | 248.83(245.98,251.71) | -1.87(-2.18,-1.56) | 422.97(419.68,426.29) | 248.83(245.98,251.71) | -1.87(-2.18,-1.56) |
| Sri Lanka | both | 112.32(109.93,114.76) | 99.54(97.37,101.75) | -0.82(-0.99,-0.65) | 112.32(109.93,114.76) | 99.54(97.37,101.75) | -0.82(-0.99,-0.65) |
| Sudan | both | 87.49(85.39,89.63) | 91.26(89.89,92.65) | 0.19(0.13,0.26) | 87.49(85.39,89.63) | 91.26(89.89,92.65) | 0.19(0.13,0.26) |
| Suriname | both | 329.60(302.22,359.08) | 256.32(235.32,278.71) | -0.81(-0.93,-0.69) | 329.60(302.22,359.08) | 256.32(235.32,278.71) | -0.81(-0.93,-0.69) |
| Sweden | both | 444.03(436.32,451.85) | 201.27(196.25,206.38) | -2.54(-2.63,-2.46) | 444.03(436.32,451.85) | 201.27(196.25,206.38) | -2.54(-2.63,-2.46) |
| Switzerland | both | 492.12(483.24,501.13) | 230.94(224.97,237.03) | -2.51(-2.72,-2.31) | 492.12(483.24,501.13) | 230.94(224.97,237.03) | -2.51(-2.72,-2.31) |
| Syrian Arab Republic | both | 92.49(89.75,95.30) | 71.53(69.02,74.12) | -0.73(-0.85,-0.60) | 92.49(89.75,95.30) | 71.53(69.02,74.12) | -0.73(-0.85,-0.60) |
| Taiwan (Province of China) | both | 197.25(194.38,200.15) | 175.33(172.21,178.51) | -0.59(-0.70,-0.48) | 197.25(194.38,200.15) | 175.33(172.21,178.51) | -0.59(-0.70,-0.48) |
| Tajikistan | both | 77.70(73.97,81.59) | 61.15(58.80,63.58) | -0.72(-0.81,-0.63) | 77.70(73.97,81.59) | 61.15(58.80,63.58) | -0.72(-0.81,-0.63) |
| Thailand | both | 216.74(214.96,218.53) | 148.95(147.28,150.63) | -1.82(-2.07,-1.57) | 216.74(214.96,218.53) | 148.95(147.28,150.63) | -1.82(-2.07,-1.57) |
| Timor-Leste | both | 103.89(93.01,115.79) | 84.94(77.49,93.02) | -0.65(-0.77,-0.54) | 103.89(93.01,115.79) | 84.94(77.49,93.02) | -0.65(-0.77,-0.54) |
| Togo | both | 55.77(51.84,59.96) | 56.41(53.90,59.02) | -0.25(-0.34,-0.16) | 55.77(51.84,59.96) | 56.41(53.90,59.02) | -0.25(-0.34,-0.16) |
| Tokelau | both | 70.55(0.01,899.28) | 66.13(0.00,946.82) | -0.20(-0.27,-0.13) | 70.55(0.01,899.28) | 66.13(0.00,946.82) | -0.20(-0.27,-0.13) |
| Tonga | both | 56.40(34.24,89.60) | 53.71(33.13,83.07) | -0.16(-0.19,-0.12) | 56.40(34.24,89.60) | 53.71(33.13,83.07) | -0.16(-0.19,-0.12) |
| Trinidad and Tobago | both | 195.53(183.51,208.16) | 163.33(151.90,175.43) | -0.15(-0.35,0.06) | 195.53(183.51,208.16) | 163.33(151.90,175.43) | -0.15(-0.35,0.06) |
| Tunisia | both | 130.85(127.04,134.75) | 126.06(122.70,129.50) | -0.14(-0.19,-0.10) | 130.85(127.04,134.75) | 126.06(122.70,129.50) | -0.14(-0.19,-0.10) |
| Turkey | both | 98.69(97.43,99.96) | 84.59(83.57,85.61) | -0.62(-0.88,-0.36) | 98.69(97.43,99.96) | 84.59(83.57,85.61) | -0.62(-0.88,-0.36) |
| Turkmenistan | both | 98.08(93.18,103.18) | 78.53(74.76,82.44) | -0.64(-0.77,-0.51) | 98.08(93.18,103.18) | 78.53(74.76,82.44) | -0.64(-0.77,-0.51) |
| Tuvalu | both | 77.82(14.93,238.57) | 83.11(23.19,215.84) | 0.27(0.20,0.33) | 77.82(14.93,238.57) | 83.11(23.19,215.84) | 0.27(0.20,0.33) |
| Uganda | both | 69.37(67.31,71.48) | 87.19(85.78,88.61) | 0.35(0.11,0.59) | 69.37(67.31,71.48) | 87.19(85.78,88.61) | 0.35(0.11,0.59) |
| Ukraine | both | 251.85(249.57,254.15) | 213.56(210.94,216.20) | -0.56(-0.61,-0.51) | 251.85(249.57,254.15) | 213.56(210.94,216.20) | -0.56(-0.61,-0.51) |
| United Arab Emirates | both | 144.42(136.27,152.99) | 157.84(152.66,163.17) | 0.52(0.44,0.60) | 144.42(136.27,152.99) | 157.84(152.66,163.17) | 0.52(0.44,0.60) |
| United Kingdom | both | 374.67(372.01,377.35) | 209.83(207.86,211.83) | -2.00(-2.10,-1.91) | 374.67(372.01,377.35) | 209.83(207.86,211.83) | -2.00(-2.10,-1.91) |
| United Republic of Tanzania | both | 57.83(56.31,59.40) | 52.12(51.19,53.06) | -0.45(-0.49,-0.40) | 57.83(56.31,59.40) | 52.12(51.19,53.06) | -0.45(-0.49,-0.40) |
| United States of America | both | 417.59(416.30,418.89) | 287.15(286.15,288.16) | -1.41(-1.65,-1.18) | 417.59(416.30,418.89) | 287.15(286.15,288.16) | -1.41(-1.65,-1.18) |
| United States Virgin Islands | both | 259.83(212.04,315.49) | 148.41(102.45,208.77) | -2.41(-2.82,-2.00) | 259.83(212.04,315.49) | 148.41(102.45,208.77) | -2.41(-2.82,-2.00) |
| Uruguay | both | 317.00(306.74,327.51) | 328.05(317.81,338.54) | 0.20(0.09,0.30) | 317.00(306.74,327.51) | 328.05(317.81,338.54) | 0.20(0.09,0.30) |
| Uzbekistan | both | 94.31(92.26,96.39) | 99.78(98.09,101.48) | 0.41(0.34,0.49) | 94.31(92.26,96.39) | 99.78(98.09,101.48) | 0.41(0.34,0.49) |
| Vanuatu | both | 90.17(67.36,118.75) | 90.20(74.23,108.78) | -0.08(-0.11,-0.05) | 90.17(67.36,118.75) | 90.20(74.23,108.78) | -0.08(-0.11,-0.05) |
| Venezuela (Bolivarian Republic of) | both | 211.86(208.72,215.05) | 150.78(148.26,153.32) | -0.85(-0.96,-0.74) | 211.86(208.72,215.05) | 150.78(148.26,153.32) | -0.85(-0.96,-0.74) |
| Viet Nam | both | 136.69(135.33,138.06) | 158.84(157.55,160.14) | 1.02(0.72,1.32) | 136.69(135.33,138.06) | 158.84(157.55,160.14) | 1.02(0.72,1.32) |
| Yemen | both | 104.84(101.88,107.86) | 86.19(84.64,87.76) | -0.58(-0.68,-0.48) | 104.84(101.88,107.86) | 86.19(84.64,87.76) | -0.58(-0.68,-0.48) |
| Zambia | both | 79.14(75.93,82.48) | 85.13(83.12,87.18) | -0.01(-0.12,0.10) | 79.14(75.93,82.48) | 85.13(83.12,87.18) | -0.01(-0.12,0.10) |
| Zimbabwe | both | 57.01(54.62,59.48) | 57.61(55.74,59.53) | -0.02(-0.20,0.16) | 57.01(54.62,59.48) | 57.61(55.74,59.53) | -0.02(-0.20,0.16) |
| Afghanistan | female | 53.45(49.81,57.35) | 55.82(53.88,57.82) | 0.53(0.32,0.75) | 53.45(49.81,57.35) | 55.82(53.88,57.82) | 0.53(0.32,0.75) |
| Albania | female | 181.21(171.41,191.46) | 155.67(144.23,167.82) | -0.35(-0.46,-0.23) | 181.21(171.41,191.46) | 155.67(144.23,167.82) | -0.35(-0.46,-0.23) |
| Algeria | female | 91.03(88.37,93.75) | 85.03(83.04,87.07) | -0.19(-0.25,-0.14) | 91.03(88.37,93.75) | 85.03(83.04,87.07) | -0.19(-0.25,-0.14) |
| American Samoa | female | 74.40(31.07,155.46) | 65.85(22.99,150.27) | -0.37(-0.44,-0.30) | 74.40(31.07,155.46) | 65.85(22.99,150.27) | -0.37(-0.44,-0.30) |
| Andorra | female | 261.53(173.27,381.96) | 216.89(138.94,325.51) | -0.64(-0.68,-0.60) | 261.53(173.27,381.96) | 216.89(138.94,325.51) | -0.64(-0.68,-0.60) |
| Angola | female | 44.22(41.31,47.30) | 41.72(40.14,43.34) | 0.17(0.01,0.33) | 44.22(41.31,47.30) | 41.72(40.14,43.34) | 0.17(0.01,0.33) |
| Antigua and Barbuda | female | 102.03(55.23,174.06) | 63.75(31.33,116.85) | -1.68(-1.78,-1.58) | 102.03(55.23,174.06) | 63.75(31.33,116.85) | -1.68(-1.78,-1.58) |
| Argentina | female | 223.46(219.76,227.21) | 220.66(217.54,223.81) | 0.06(-0.01,0.13) | 223.46(219.76,227.21) | 220.66(217.54,223.81) | 0.06(-0.01,0.13) |
| Armenia | female | 54.77(49.47,60.52) | 51.44(45.26,58.31) | 0.22(-0.02,0.47) | 54.77(49.47,60.52) | 51.44(45.26,58.31) | 0.22(-0.02,0.47) |
| Australia | female | 137.86(133.87,141.93) | 95.23(92.23,98.30) | -1.19(-1.23,-1.15) | 137.86(133.87,141.93) | 95.23(92.23,98.30) | -1.19(-1.23,-1.15) |
| Austria | female | 245.71(237.53,254.12) | 144.60(137.94,151.51) | -1.89(-2.03,-1.75) | 245.71(237.53,254.12) | 144.60(137.94,151.51) | -1.89(-2.03,-1.75) |
| Azerbaijan | female | 60.23(56.48,64.18) | 48.10(45.03,51.34) | -0.71(-0.81,-0.62) | 60.23(56.48,64.18) | 48.10(45.03,51.34) | -0.71(-0.81,-0.62) |
| Bahamas | female | 148.89(119.71,183.52) | 104.63(83.23,129.92) | -1.06(-1.16,-0.96) | 148.89(119.71,183.52) | 104.63(83.23,129.92) | -1.06(-1.16,-0.96) |
| Bahrain | female | 95.99(77.57,117.78) | 70.93(60.64,82.54) | -1.21(-1.29,-1.12) | 95.99(77.57,117.78) | 70.93(60.64,82.54) | -1.21(-1.29,-1.12) |
| Bangladesh | female | 34.24(33.45,35.04) | 22.79(22.30,23.29) | -1.23(-1.31,-1.15) | 34.24(33.45,35.04) | 22.79(22.30,23.29) | -1.23(-1.31,-1.15) |
| Barbados | female | 95.45(71.31,125.30) | 78.32(55.31,108.01) | -0.70(-0.81,-0.60) | 95.45(71.31,125.30) | 78.32(55.31,108.01) | -0.70(-0.81,-0.60) |
| Belarus | female | 148.56(143.13,154.15) | 120.05(114.10,126.26) | -0.76(-1.08,-0.43) | 148.56(143.13,154.15) | 120.05(114.10,126.26) | -0.76(-1.08,-0.43) |
| Belgium | female | 240.63(233.34,248.09) | 182.20(175.70,188.89) | -1.40(-1.65,-1.14) | 240.63(233.34,248.09) | 182.20(175.70,188.89) | -1.40(-1.65,-1.14) |
| Belize | female | 144.73(108.48,191.15) | 103.56(84.31,126.05) | -1.20(-1.34,-1.06) | 144.73(108.48,191.15) | 103.56(84.31,126.05) | -1.20(-1.34,-1.06) |
| Benin | female | 35.50(31.77,39.59) | 25.34(23.47,27.34) | -1.51(-1.68,-1.34) | 35.50(31.77,39.59) | 25.34(23.47,27.34) | -1.51(-1.68,-1.34) |
| Bermuda | female | 101.14(52.15,181.57) | 53.28(15.44,136.61) | -2.22(-2.32,-2.13) | 101.14(52.15,181.57) | 53.28(15.44,136.61) | -2.22(-2.32,-2.13) |
| Bhutan | female | 61.98(48.60,78.37) | 40.09(30.99,51.09) | -1.57(-1.63,-1.51) | 61.98(48.60,78.37) | 40.09(30.99,51.09) | -1.57(-1.63,-1.51) |
| Bolivia (Plurinational State of) | female | 59.28(55.10,63.71) | 51.39(48.58,54.32) | -0.41(-0.55,-0.27) | 59.28(55.10,63.71) | 51.39(48.58,54.32) | -0.41(-0.55,-0.27) |
| Bosnia and Herzegovina | female | 141.81(134.15,149.79) | 121.04(111.24,131.53) | -0.41(-0.60,-0.23) | 141.81(134.15,149.79) | 121.04(111.24,131.53) | -0.41(-0.60,-0.23) |
| Botswana | female | 37.58(30.63,45.81) | 45.49(39.91,51.63) | 0.75(0.59,0.92) | 37.58(30.63,45.81) | 45.49(39.91,51.63) | 0.75(0.59,0.92) |
| Brazil | female | 109.60(108.46,110.75) | 83.24(82.36,84.12) | -0.83(-1.04,-0.63) | 109.60(108.46,110.75) | 83.24(82.36,84.12) | -0.83(-1.04,-0.63) |
| Brunei Darussalam | female | 317.36(272.68,367.55) | 245.81(214.18,281.05) | -0.75(-0.91,-0.60) | 317.36(272.68,367.55) | 245.81(214.18,281.05) | -0.75(-0.91,-0.60) |
| Bulgaria | female | 282.06(273.46,290.86) | 216.76(206.77,227.12) | -0.93(-1.09,-0.77) | 282.06(273.46,290.86) | 216.76(206.77,227.12) | -0.93(-1.09,-0.77) |
| Burkina Faso | female | 34.80(32.06,37.73) | 40.48(38.66,42.37) | 0.66(0.60,0.72) | 34.80(32.06,37.73) | 40.48(38.66,42.37) | 0.66(0.60,0.72) |
| Burundi | female | 53.66(49.32,58.32) | 39.61(37.24,42.09) | -1.12(-1.18,-1.07) | 53.66(49.32,58.32) | 39.61(37.24,42.09) | -1.12(-1.18,-1.07) |
| Cabo Verde | female | 23.54(13.39,39.61) | 20.67(13.33,30.66) | -0.32(-0.63,-0.01) | 23.54(13.39,39.61) | 20.67(13.33,30.66) | -0.32(-0.63,-0.01) |
| Cambodia | female | 96.88(92.71,101.20) | 78.43(75.55,81.39) | -0.86(-0.96,-0.76) | 96.88(92.71,101.20) | 78.43(75.55,81.39) | -0.86(-0.96,-0.76) |
| Cameroon | female | 32.17(29.71,34.81) | 27.87(26.60,29.18) | -0.55(-0.62,-0.47) | 32.17(29.71,34.81) | 27.87(26.60,29.18) | -0.55(-0.62,-0.47) |
| Canada | female | 164.92(161.40,168.49) | 128.09(125.10,131.15) | -0.79(-0.86,-0.72) | 164.92(161.40,168.49) | 128.09(125.10,131.15) | -0.79(-0.86,-0.72) |
| Central African Republic | female | 35.55(30.66,41.08) | 25.43(22.59,28.56) | -1.06(-1.15,-0.98) | 35.55(30.66,41.08) | 25.43(22.59,28.56) | -1.06(-1.15,-0.98) |
| Chad | female | 25.47(22.58,28.66) | 27.63(25.84,29.53) | 0.36(0.21,0.51) | 25.47(22.58,28.66) | 27.63(25.84,29.53) | 0.36(0.21,0.51) |
| Chile | female | 144.92(140.58,149.36) | 159.50(155.24,163.86) | -0.26(-0.46,-0.05) | 144.92(140.58,149.36) | 159.50(155.24,163.86) | -0.26(-0.46,-0.05) |
| China | female | 239.78(239.18,240.38) | 220.46(219.83,221.10) | -0.62(-0.92,-0.31) | 239.78(239.18,240.38) | 220.46(219.83,221.10) | -0.62(-0.92,-0.31) |
| Colombia | female | 44.18(42.66,45.74) | 53.67(52.23,55.15) | 0.68(0.63,0.73) | 44.18(42.66,45.74) | 53.67(52.23,55.15) | 0.68(0.63,0.73) |
| Comoros | female | 50.23(36.42,68.18) | 38.08(28.99,49.24) | -1.15(-1.27,-1.03) | 50.23(36.42,68.18) | 38.08(28.99,49.24) | -1.15(-1.27,-1.03) |
| Congo | female | 34.91(29.79,40.76) | 32.66(29.43,36.16) | -0.12(-0.19,-0.05) | 34.91(29.79,40.76) | 32.66(29.43,36.16) | -0.12(-0.19,-0.05) |
| Cook Islands | female | 90.33(20.86,262.69) | 87.03(16.19,271.60) | -0.05(-0.07,-0.02) | 90.33(20.86,262.69) | 87.03(16.19,271.60) | -0.05(-0.07,-0.02) |
| Costa Rica | female | 53.02(47.55,58.98) | 58.62(53.85,63.72) | 0.38(0.33,0.42) | 53.02(47.55,58.98) | 58.62(53.85,63.72) | 0.38(0.33,0.42) |
| Croatia | female | 274.75(263.71,286.13) | 171.17(160.56,182.35) | -1.32(-1.47,-1.17) | 274.75(263.71,286.13) | 171.17(160.56,182.35) | -1.32(-1.47,-1.17) |
| Cuba | female | 283.51(276.90,290.26) | 100.36(95.58,105.34) | -3.95(-4.26,-3.64) | 283.51(276.90,290.26) | 100.36(95.58,105.34) | -3.95(-4.26,-3.64) |
| Cyprus | female | 225.16(201.51,250.89) | 168.82(151.44,187.86) | -0.97(-1.24,-0.70) | 225.16(201.51,250.89) | 168.82(151.44,187.86) | -0.97(-1.24,-0.70) |
| Czechia | female | 330.44(322.05,338.99) | 175.83(168.63,183.27) | -2.30(-2.61,-1.99) | 330.44(322.05,338.99) | 175.83(168.63,183.27) | -2.30(-2.61,-1.99) |
| Côte d'Ivoire | female | 33.92(31.58,36.42) | 31.27(29.81,32.78) | -0.40(-0.45,-0.35) | 33.92(31.58,36.42) | 31.27(29.81,32.78) | -0.40(-0.45,-0.35) |
| Democratic People's Republic of Korea | female | 193.55(189.43,197.74) | 224.23(220.00,228.52) | 0.43(0.37,0.50) | 193.55(189.43,197.74) | 224.23(220.00,228.52) | 0.43(0.37,0.50) |
| Democratic Republic of the Congo | female | 44.81(43.27,46.39) | 33.19(32.35,34.05) | -1.24(-1.34,-1.15) | 44.81(43.27,46.39) | 33.19(32.35,34.05) | -1.24(-1.34,-1.15) |
| Denmark | female | 476.64(462.41,491.21) | 177.36(168.52,186.56) | -3.68(-3.91,-3.45) | 476.64(462.41,491.21) | 177.36(168.52,186.56) | -3.68(-3.91,-3.45) |
| Djibouti | female | 37.19(24.95,54.16) | 28.60(22.34,36.10) | -0.96(-1.04,-0.88) | 37.19(24.95,54.16) | 28.60(22.34,36.10) | -0.96(-1.04,-0.88) |
| Dominica | female | 121.27(70.90,197.66) | 109.34(59.34,184.87) | -0.82(-1.07,-0.57) | 121.27(70.90,197.66) | 109.34(59.34,184.87) | -0.82(-1.07,-0.57) |
| Dominican Republic | female | 102.45(97.56,107.55) | 105.51(101.32,109.84) | 0.05(-0.12,0.21) | 102.45(97.56,107.55) | 105.51(101.32,109.84) | 0.05(-0.12,0.21) |
| Ecuador | female | 66.18(62.74,69.78) | 79.76(76.88,82.71) | 0.81(0.69,0.92) | 66.18(62.74,69.78) | 79.76(76.88,82.71) | 0.81(0.69,0.92) |
| Egypt | female | 75.65(74.01,77.31) | 74.18(73.01,75.37) | -0.01(-0.08,0.06) | 75.65(74.01,77.31) | 74.18(73.01,75.37) | -0.01(-0.08,0.06) |
| El Salvador | female | 44.88(40.98,49.09) | 44.44(40.95,48.16) | -0.17(-0.35,0.01) | 44.88(40.98,49.09) | 44.44(40.95,48.16) | -0.17(-0.35,0.01) |
| Equatorial Guinea | female | 33.06(21.81,48.44) | 40.60(33.76,48.50) | 1.25(0.81,1.68) | 33.06(21.81,48.44) | 40.60(33.76,48.50) | 1.25(0.81,1.68) |
| Eritrea | female | 34.06(29.68,38.95) | 32.63(29.64,35.86) | -0.03(-0.19,0.13) | 34.06(29.68,38.95) | 32.63(29.64,35.86) | -0.03(-0.19,0.13) |
| Estonia | female | 194.37(178.03,211.84) | 104.67(89.83,121.38) | -2.38(-2.60,-2.17) | 194.37(178.03,211.84) | 104.67(89.83,121.38) | -2.38(-2.60,-2.17) |
| Eswatini | female | 46.44(36.51,58.59) | 40.77(33.41,49.34) | -0.37(-0.49,-0.24) | 46.44(36.51,58.59) | 40.77(33.41,49.34) | -0.37(-0.49,-0.24) |
| Ethiopia | female | 45.27(43.92,46.66) | 27.21(26.54,27.90) | -1.81(-1.94,-1.68) | 45.27(43.92,46.66) | 27.21(26.54,27.90) | -1.81(-1.94,-1.68) |
| Fiji | female | 68.69(56.47,82.92) | 59.15(48.28,71.75) | -0.51(-0.54,-0.48) | 68.69(56.47,82.92) | 59.15(48.28,71.75) | -0.51(-0.54,-0.48) |
| Finland | female | 246.20(235.46,257.34) | 136.04(127.84,144.63) | -2.19(-2.43,-1.95) | 246.20(235.46,257.34) | 136.04(127.84,144.63) | -2.19(-2.43,-1.95) |
| France | female | 255.02(252.00,258.08) | 179.03(176.37,181.73) | -1.40(-1.60,-1.21) | 255.02(252.00,258.08) | 179.03(176.37,181.73) | -1.40(-1.60,-1.21) |
| Gabon | female | 40.49(31.91,50.93) | 35.20(29.62,41.59) | -0.37(-0.42,-0.32) | 40.49(31.91,50.93) | 35.20(29.62,41.59) | -0.37(-0.42,-0.32) |
| Gambia | female | 25.41(18.77,33.99) | 21.93(18.06,26.46) | -0.68(-0.78,-0.59) | 25.41(18.77,33.99) | 21.93(18.06,26.46) | -0.68(-0.78,-0.59) |
| Georgia | female | 81.54(76.20,87.16) | 62.11(55.42,69.44) | -1.04(-1.18,-0.89) | 81.54(76.20,87.16) | 62.11(55.42,69.44) | -1.04(-1.18,-0.89) |
| Germany | female | 263.04(260.22,265.89) | 150.77(148.45,153.13) | -2.14(-2.28,-2.00) | 263.04(260.22,265.89) | 150.77(148.45,153.13) | -2.14(-2.28,-2.00) |
| Ghana | female | 43.47(41.11,45.95) | 37.51(36.13,38.94) | -0.59(-0.78,-0.40) | 43.47(41.11,45.95) | 37.51(36.13,38.94) | -0.59(-0.78,-0.40) |
| Greece | female | 215.56(208.91,222.36) | 190.55(183.08,198.26) | -0.50(-0.58,-0.42) | 215.56(208.91,222.36) | 190.55(183.08,198.26) | -0.50(-0.58,-0.42) |
| Greenland | female | 128.96(70.27,222.53) | 84.03(36.08,168.77) | -1.75(-1.89,-1.60) | 128.96(70.27,222.53) | 84.03(36.08,168.77) | -1.75(-1.89,-1.60) |
| Grenada | female | 119.74(73.08,187.56) | 76.10(42.22,127.28) | -1.58(-1.62,-1.54) | 119.74(73.08,187.56) | 76.10(42.22,127.28) | -1.58(-1.62,-1.54) |
| Guam | female | 91.43(59.79,134.24) | 74.99(45.72,116.21) | -0.68(-0.89,-0.46) | 91.43(59.79,134.24) | 74.99(45.72,116.21) | -0.68(-0.89,-0.46) |
| Guatemala | female | 48.91(45.45,52.58) | 49.61(47.31,52.00) | 0.27(0.12,0.42) | 48.91(45.45,52.58) | 49.61(47.31,52.00) | 0.27(0.12,0.42) |
| Guinea | female | 33.57(30.26,37.17) | 33.17(31.07,35.39) | -0.06(-0.19,0.07) | 33.57(30.26,37.17) | 33.17(31.07,35.39) | -0.06(-0.19,0.07) |
| Guinea-Bissau | female | 42.66(33.95,53.11) | 40.20(34.49,46.65) | -0.28(-0.50,-0.05) | 42.66(33.95,53.11) | 40.20(34.49,46.65) | -0.28(-0.50,-0.05) |
| Guyana | female | 117.15(101.65,134.58) | 109.40(93.71,127.09) | -0.58(-0.72,-0.44) | 117.15(101.65,134.58) | 109.40(93.71,127.09) | -0.58(-0.72,-0.44) |
| Haiti | female | 140.85(134.49,147.45) | 124.88(120.79,129.07) | -0.35(-0.47,-0.24) | 140.85(134.49,147.45) | 124.88(120.79,129.07) | -0.35(-0.47,-0.24) |
| Honduras | female | 49.11(44.60,53.98) | 57.96(54.87,61.18) | 0.38(0.15,0.61) | 49.11(44.60,53.98) | 57.96(54.87,61.18) | 0.38(0.15,0.61) |
| Hungary | female | 309.48(301.28,317.85) | 161.71(154.77,168.90) | -2.47(-2.65,-2.29) | 309.48(301.28,317.85) | 161.71(154.77,168.90) | -2.47(-2.65,-2.29) |
| Iceland | female | 196.42(159.57,239.39) | 140.40(110.89,175.66) | -1.30(-1.43,-1.18) | 196.42(159.57,239.39) | 140.40(110.89,175.66) | -1.30(-1.43,-1.18) |
| India | female | 68.26(67.87,68.67) | 54.65(54.38,54.92) | -0.92(-1.00,-0.84) | 68.26(67.87,68.67) | 54.65(54.38,54.92) | -0.92(-1.00,-0.84) |
| Indonesia | female | 111.35(110.31,112.39) | 74.42(73.70,75.15) | -1.43(-1.66,-1.19) | 111.35(110.31,112.39) | 74.42(73.70,75.15) | -1.43(-1.66,-1.19) |
| Iran (Islamic Republic of) | female | 106.50(104.54,108.48) | 97.53(95.97,99.11) | -0.33(-0.40,-0.26) | 106.50(104.54,108.48) | 97.53(95.97,99.11) | -0.33(-0.40,-0.26) |
| Iraq | female | 75.13(72.24,78.13) | 64.40(62.69,66.15) | -0.49(-0.59,-0.39) | 75.13(72.24,78.13) | 64.40(62.69,66.15) | -0.49(-0.59,-0.39) |
| Ireland | female | 272.06(259.92,284.63) | 130.80(122.75,139.25) | -2.52(-2.60,-2.44) | 272.06(259.92,284.63) | 130.80(122.75,139.25) | -2.52(-2.60,-2.44) |
| Israel | female | 172.25(164.07,180.76) | 126.67(121.28,132.24) | -1.10(-1.25,-0.95) | 172.25(164.07,180.76) | 126.67(121.28,132.24) | -1.10(-1.25,-0.95) |
| Italy | female | 292.88(289.61,296.18) | 115.76(113.29,118.28) | -3.85(-4.26,-3.43) | 292.88(289.61,296.18) | 115.76(113.29,118.28) | -3.85(-4.26,-3.43) |
| Jamaica | female | 67.72(60.74,75.36) | 62.84(56.62,69.58) | 0.06(-0.06,0.17) | 67.72(60.74,75.36) | 62.84(56.62,69.58) | 0.06(-0.06,0.17) |
| Japan | female | 311.22(308.89,313.56) | 131.10(129.26,132.96) | -3.29(-3.47,-3.10) | 311.22(308.89,313.56) | 131.10(129.26,132.96) | -3.29(-3.47,-3.10) |
| Jordan | female | 75.57(69.17,82.50) | 58.35(55.36,61.47) | -0.99(-1.10,-0.89) | 75.57(69.17,82.50) | 58.35(55.36,61.47) | -0.99(-1.10,-0.89) |
| Kazakhstan | female | 89.69(86.51,92.96) | 85.24(82.13,88.46) | 0.02(-0.16,0.21) | 89.69(86.51,92.96) | 85.24(82.13,88.46) | 0.02(-0.16,0.21) |
| Kenya | female | 43.71(41.72,45.78) | 32.21(31.14,33.31) | -1.41(-1.61,-1.20) | 43.71(41.72,45.78) | 32.21(31.14,33.31) | -1.41(-1.61,-1.20) |
| Kiribati | female | 55.15(24.67,109.07) | 51.97(27.87,88.89) | -0.19(-0.45,0.07) | 55.15(24.67,109.07) | 51.97(27.87,88.89) | -0.19(-0.45,0.07) |
| Kuwait | female | 93.83(83.94,104.60) | 56.83(51.83,62.25) | -1.95(-2.26,-1.65) | 93.83(83.94,104.60) | 56.83(51.83,62.25) | -1.95(-2.26,-1.65) |
| Kyrgyzstan | female | 78.72(73.04,84.75) | 66.88(62.58,71.42) | -0.29(-0.41,-0.17) | 78.72(73.04,84.75) | 66.88(62.58,71.42) | -0.29(-0.41,-0.17) |
| Lao People's Democratic Republic | female | 106.08(99.04,113.53) | 87.27(82.74,91.99) | -0.70(-0.80,-0.60) | 106.08(99.04,113.53) | 87.27(82.74,91.99) | -0.70(-0.80,-0.60) |
| Latvia | female | 201.98(189.20,215.43) | 121.87(108.17,136.94) | -1.98(-2.29,-1.68) | 201.98(189.20,215.43) | 121.87(108.17,136.94) | -1.98(-2.29,-1.68) |
| Lebanon | female | 81.67(74.59,89.27) | 57.74(53.20,62.60) | -1.32(-1.42,-1.21) | 81.67(74.59,89.27) | 57.74(53.20,62.60) | -1.32(-1.42,-1.21) |
| Lesotho | female | 36.64(30.16,44.17) | 33.74(28.41,39.85) | -0.37(-0.43,-0.30) | 36.64(30.16,44.17) | 33.74(28.41,39.85) | -0.37(-0.43,-0.30) |
| Liberia | female | 31.40(26.57,36.93) | 25.40(22.55,28.53) | -1.00(-1.12,-0.87) | 31.40(26.57,36.93) | 25.40(22.55,28.53) | -1.00(-1.12,-0.87) |
| Libya | female | 101.88(94.76,109.47) | 70.00(65.74,74.47) | -0.99(-1.13,-0.85) | 101.88(94.76,109.47) | 70.00(65.74,74.47) | -0.99(-1.13,-0.85) |
| Lithuania | female | 192.36(182.05,203.11) | 121.76(110.73,133.68) | -1.59(-1.95,-1.23) | 192.36(182.05,203.11) | 121.76(110.73,133.68) | -1.59(-1.95,-1.23) |
| Luxembourg | female | 241.49(205.07,283.09) | 159.64(135.16,187.62) | -1.55(-1.70,-1.40) | 241.49(205.07,283.09) | 159.64(135.16,187.62) | -1.55(-1.70,-1.40) |
| Madagascar | female | 46.48(43.69,49.41) | 37.57(36.02,39.18) | -0.65(-0.78,-0.52) | 46.48(43.69,49.41) | 37.57(36.02,39.18) | -0.65(-0.78,-0.52) |
| Malawi | female | 41.98(39.07,45.07) | 30.66(28.99,32.40) | -1.10(-1.17,-1.04) | 41.98(39.07,45.07) | 30.66(28.99,32.40) | -1.10(-1.17,-1.04) |
| Malaysia | female | 108.21(104.90,111.61) | 88.65(86.38,90.97) | -0.60(-0.65,-0.55) | 108.21(104.90,111.61) | 88.65(86.38,90.97) | -0.60(-0.65,-0.55) |
| Maldives | female | 78.67(53.27,114.41) | 37.00(24.96,53.31) | -2.43(-2.53,-2.33) | 78.67(53.27,114.41) | 37.00(24.96,53.31) | -2.43(-2.53,-2.33) |
| Mali | female | 25.83(23.39,28.49) | 22.37(21.00,23.80) | -0.53(-0.66,-0.40) | 25.83(23.39,28.49) | 22.37(21.00,23.80) | -0.53(-0.66,-0.40) |
| Malta | female | 170.03(139.74,205.21) | 129.53(101.67,163.30) | -0.89(-1.02,-0.77) | 170.03(139.74,205.21) | 129.53(101.67,163.30) | -0.89(-1.02,-0.77) |
| Marshall Islands | female | 61.76(20.32,149.79) | 59.19(23.41,124.38) | -0.24(-0.32,-0.16) | 61.76(20.32,149.79) | 59.19(23.41,124.38) | -0.24(-0.32,-0.16) |
| Mauritania | female | 45.44(39.02,52.70) | 39.80(35.74,44.23) | -0.47(-0.52,-0.41) | 45.44(39.02,52.70) | 39.80(35.74,44.23) | -0.47(-0.52,-0.41) |
| Mauritius | female | 48.78(40.39,58.43) | 49.25(40.46,59.44) | 0.11(0.05,0.17) | 48.78(40.39,58.43) | 49.25(40.46,59.44) | 0.11(0.05,0.17) |
| Mexico | female | 75.94(74.68,77.22) | 66.99(66.00,67.99) | 0.15(-0.48,0.79) | 75.94(74.68,77.22) | 66.99(66.00,67.99) | 0.15(-0.48,0.79) |
| Micronesia (Federated States of) | female | 77.46(43.36,129.96) | 78.92(45.25,129.25) | 0.05(-0.02,0.13) | 77.46(43.36,129.96) | 78.92(45.25,129.25) | 0.05(-0.02,0.13) |
| Monaco | female | 188.21(78.50,396.02) | 142.41(53.56,309.98) | -0.90(-0.96,-0.85) | 188.21(78.50,396.02) | 142.41(53.56,309.98) | -0.90(-0.96,-0.85) |
| Mongolia | female | 68.95(61.36,77.37) | 75.44(68.65,82.75) | 0.60(0.49,0.71) | 68.95(61.36,77.37) | 75.44(68.65,82.75) | 0.60(0.49,0.71) |
| Montenegro | female | 243.64(216.55,273.20) | 205.43(177.73,236.38) | -0.67(-0.86,-0.47) | 243.64(216.55,273.20) | 205.43(177.73,236.38) | -0.67(-0.86,-0.47) |
| Morocco | female | 72.19(69.92,74.53) | 67.63(65.75,69.55) | -0.27(-0.38,-0.17) | 72.19(69.92,74.53) | 67.63(65.75,69.55) | -0.27(-0.38,-0.17) |
| Mozambique | female | 45.35(42.78,48.05) | 36.88(35.37,38.44) | -0.55(-0.62,-0.48) | 45.35(42.78,48.05) | 36.88(35.37,38.44) | -0.55(-0.62,-0.48) |
| Myanmar | female | 104.91(102.77,107.09) | 71.69(70.15,73.26) | -1.42(-1.51,-1.32) | 104.91(102.77,107.09) | 71.69(70.15,73.26) | -1.42(-1.51,-1.32) |
| Namibia | female | 44.08(36.67,52.74) | 40.75(35.52,46.56) | -0.18(-0.26,-0.10) | 44.08(36.67,52.74) | 40.75(35.52,46.56) | -0.18(-0.26,-0.10) |
| Nauru | female | 95.53(10.82,372.34) | 96.73(13.73,342.91) | 0.02(-0.07,0.11) | 95.53(10.82,372.34) | 96.73(13.73,342.91) | 0.02(-0.07,0.11) |
| Nepal | female | 46.84(44.67,49.09) | 27.67(26.47,28.90) | -1.93(-2.05,-1.81) | 46.84(44.67,49.09) | 27.67(26.47,28.90) | -1.93(-2.05,-1.81) |
| Netherlands | female | 398.74(391.26,406.33) | 155.44(150.57,160.43) | -3.27(-3.56,-2.99) | 398.74(391.26,406.33) | 155.44(150.57,160.43) | -3.27(-3.56,-2.99) |
| New Zealand | female | 254.83(243.02,267.07) | 115.67(108.51,123.21) | -4.13(-4.81,-3.45) | 254.83(243.02,267.07) | 115.67(108.51,123.21) | -4.13(-4.81,-3.45) |
| Nicaragua | female | 41.01(36.59,45.87) | 36.43(33.35,39.71) | -0.31(-0.46,-0.16) | 41.01(36.59,45.87) | 36.43(33.35,39.71) | -0.31(-0.46,-0.16) |
| Niger | female | 29.31(26.57,32.29) | 29.08(27.50,30.75) | 0.11(0.03,0.20) | 29.31(26.57,32.29) | 29.08(27.50,30.75) | 0.11(0.03,0.20) |
| Nigeria | female | 37.34(36.41,38.28) | 28.97(28.49,29.47) | -0.80(-0.94,-0.66) | 37.34(36.41,38.28) | 28.97(28.49,29.47) | -0.80(-0.94,-0.66) |
| Niue | female | 68.16(0.00,1173.77) | 66.61(0.00,1518.65) | -0.12(-0.19,-0.05) | 68.16(0.00,1173.77) | 66.61(0.00,1518.65) | -0.12(-0.19,-0.05) |
| North Macedonia | female | 201.11(187.24,215.74) | 144.78(132.16,158.37) | -1.23(-1.29,-1.18) | 201.11(187.24,215.74) | 144.78(132.16,158.37) | -1.23(-1.29,-1.18) |
| Northern Mariana Islands | female | 119.19(64.95,202.76) | 86.48(34.03,184.46) | -1.24(-1.31,-1.18) | 119.19(64.95,202.76) | 86.48(34.03,184.46) | -1.24(-1.31,-1.18) |
| Norway | female | 231.65(221.03,242.66) | 113.73(106.55,121.29) | -2.47(-2.61,-2.32) | 231.65(221.03,242.66) | 113.73(106.55,121.29) | -2.47(-2.61,-2.32) |
| Oman | female | 118.51(106.24,131.90) | 107.65(100.26,115.46) | -0.27(-0.37,-0.17) | 118.51(106.24,131.90) | 107.65(100.26,115.46) | -0.27(-0.37,-0.17) |
| Pakistan | female | 66.80(65.62,67.99) | 60.19(59.51,60.88) | -0.42(-0.59,-0.25) | 66.80(65.62,67.99) | 60.19(59.51,60.88) | -0.42(-0.59,-0.25) |
| Palau | female | 79.96(14.37,254.62) | 78.22(8.28,311.92) | -0.07(-0.11,-0.04) | 79.96(14.37,254.62) | 78.22(8.28,311.92) | -0.07(-0.11,-0.04) |
| Palestine | female | 50.87(43.80,58.94) | 41.49(37.72,45.57) | -0.85(-0.98,-0.72) | 50.87(43.80,58.94) | 41.49(37.72,45.57) | -0.85(-0.98,-0.72) |
| Panama | female | 52.06(45.97,58.78) | 48.67(43.98,53.72) | -0.30(-0.35,-0.24) | 52.06(45.97,58.78) | 48.67(43.98,53.72) | -0.30(-0.35,-0.24) |
| Papua New Guinea | female | 68.11(62.47,74.17) | 53.69(50.60,56.93) | -1.05(-1.18,-0.91) | 68.11(62.47,74.17) | 53.69(50.60,56.93) | -1.05(-1.18,-0.91) |
| Paraguay | female | 77.58(71.55,84.00) | 86.35(81.72,91.19) | 0.49(0.40,0.57) | 77.58(71.55,84.00) | 86.35(81.72,91.19) | 0.49(0.40,0.57) |
| Peru | female | 56.17(53.99,58.41) | 57.11(55.38,58.88) | 0.22(0.15,0.30) | 56.17(53.99,58.41) | 57.11(55.38,58.88) | 0.22(0.15,0.30) |
| Philippines | female | 77.86(76.33,79.40) | 52.46(51.53,53.41) | -1.38(-1.46,-1.29) | 77.86(76.33,79.40) | 52.46(51.53,53.41) | -1.38(-1.46,-1.29) |
| Poland | female | 269.94(266.03,273.90) | 180.28(176.61,184.02) | -1.57(-1.87,-1.28) | 269.94(266.03,273.90) | 180.28(176.61,184.02) | -1.57(-1.87,-1.28) |
| Portugal | female | 265.41(258.14,272.83) | 147.52(141.22,154.04) | -1.99(-2.20,-1.78) | 265.41(258.14,272.83) | 147.52(141.22,154.04) | -1.99(-2.20,-1.78) |
| Puerto Rico | female | 123.61(115.68,131.93) | 95.29(87.04,104.12) | -1.01(-1.12,-0.90) | 123.61(115.68,131.93) | 95.29(87.04,104.12) | -1.01(-1.12,-0.90) |
| Qatar | female | 117.21(91.97,147.65) | 90.11(80.18,101.08) | -0.99(-1.06,-0.91) | 117.21(91.97,147.65) | 90.11(80.18,101.08) | -0.99(-1.06,-0.91) |
| Republic of Korea | female | 351.22(347.59,354.87) | 156.94(153.99,159.93) | -3.20(-3.44,-2.96) | 351.22(347.59,354.87) | 156.94(153.99,159.93) | -3.20(-3.44,-2.96) |
| Republic of Moldova | female | 159.82(151.42,168.58) | 98.75(90.29,107.85) | -1.53(-1.68,-1.38) | 159.82(151.42,168.58) | 98.75(90.29,107.85) | -1.53(-1.68,-1.38) |
| Romania | female | 257.48(252.67,262.37) | 198.18(192.67,203.80) | -0.94(-1.07,-0.82) | 257.48(252.67,262.37) | 198.18(192.67,203.80) | -0.94(-1.07,-0.82) |
| Russian Federation | female | 184.45(182.83,186.07) | 157.16(155.43,158.91) | -0.44(-0.71,-0.17) | 184.45(182.83,186.07) | 157.16(155.43,158.91) | -0.44(-0.71,-0.17) |
| Rwanda | female | 79.79(75.12,84.70) | 46.14(43.70,48.68) | -2.26(-2.49,-2.03) | 79.79(75.12,84.70) | 46.14(43.70,48.68) | -2.26(-2.49,-2.03) |
| Saint Kitts and Nevis | female | 116.29(56.07,217.96) | 57.28(21.48,125.35) | -2.64(-2.77,-2.51) | 116.29(56.07,217.96) | 57.28(21.48,125.35) | -2.64(-2.77,-2.51) |
| Saint Lucia | female | 129.59(91.41,180.40) | 89.33(59.41,129.76) | -1.29(-1.36,-1.22) | 129.59(91.41,180.40) | 89.33(59.41,129.76) | -1.29(-1.36,-1.22) |
| Saint Vincent and the Grenadines | female | 101.11(64.17,154.88) | 72.40(40.16,120.52) | -1.26(-1.41,-1.12) | 101.11(64.17,154.88) | 72.40(40.16,120.52) | -1.26(-1.41,-1.12) |
| Samoa | female | 75.67(47.73,115.86) | 80.12(54.49,114.39) | 0.23(0.19,0.27) | 75.67(47.73,115.86) | 80.12(54.49,114.39) | 0.23(0.19,0.27) |
| San Marino | female | 200.97(93.75,379.72) | 167.80(69.35,343.17) | -0.63(-0.68,-0.57) | 200.97(93.75,379.72) | 167.80(69.35,343.17) | -0.63(-0.68,-0.57) |
| Sao Tome and Principe | female | 23.51(7.51,59.75) | 21.56(10.22,40.60) | -0.71(-0.91,-0.50) | 23.51(7.51,59.75) | 21.56(10.22,40.60) | -0.71(-0.91,-0.50) |
| Saudi Arabia | female | 102.58(98.82,106.46) | 97.87(95.57,100.21) | 0.15(0.03,0.28) | 102.58(98.82,106.46) | 97.87(95.57,100.21) | 0.15(0.03,0.28) |
| Senegal | female | 28.29(25.60,31.23) | 24.84(23.15,26.63) | -0.55(-0.65,-0.45) | 28.29(25.60,31.23) | 24.84(23.15,26.63) | -0.55(-0.65,-0.45) |
| Serbia | female | 306.19(297.94,314.61) | 167.43(160.57,174.52) | -2.20(-2.32,-2.08) | 306.19(297.94,314.61) | 167.43(160.57,174.52) | -2.20(-2.32,-2.08) |
| Seychelles | female | 71.77(35.69,134.26) | 77.46(41.43,132.68) | 0.30(0.14,0.46) | 71.77(35.69,134.26) | 77.46(41.43,132.68) | 0.30(0.14,0.46) |
| Sierra Leone | female | 30.80(27.18,34.80) | 28.56(26.21,31.09) | -0.26(-0.30,-0.22) | 30.80(27.18,34.80) | 28.56(26.21,31.09) | -0.26(-0.30,-0.22) |
| Singapore | female | 188.23(178.25,198.65) | 113.13(105.50,121.25) | -1.67(-1.74,-1.60) | 188.23(178.25,198.65) | 113.13(105.50,121.25) | -1.67(-1.74,-1.60) |
| Slovakia | female | 289.07(278.51,299.94) | 187.18(177.38,197.42) | -1.69(-1.79,-1.59) | 289.07(278.51,299.94) | 187.18(177.38,197.42) | -1.69(-1.79,-1.59) |
| Slovenia | female | 381.76(361.86,402.51) | 171.41(155.28,188.85) | -2.78(-3.20,-2.35) | 381.76(361.86,402.51) | 171.41(155.28,188.85) | -2.78(-3.20,-2.35) |
| Solomon Islands | female | 69.20(49.57,95.02) | 73.50(59.73,89.59) | 0.18(0.13,0.22) | 69.20(49.57,95.02) | 73.50(59.73,89.59) | 0.18(0.13,0.22) |
| Somalia | female | 40.77(37.47,44.29) | 29.57(27.87,31.35) | -1.07(-1.11,-1.03) | 40.77(37.47,44.29) | 29.57(27.87,31.35) | -1.07(-1.11,-1.03) |
| South Africa | female | 72.68(70.82,74.57) | 53.52(52.21,54.86) | -0.89(-1.01,-0.76) | 72.68(70.82,74.57) | 53.52(52.21,54.86) | -0.89(-1.01,-0.76) |
| South Sudan | female | 42.99(39.13,47.17) | 28.16(25.77,30.74) | -1.37(-1.45,-1.29) | 42.99(39.13,47.17) | 28.16(25.77,30.74) | -1.37(-1.45,-1.29) |
| Spain | female | 238.68(235.17,242.24) | 138.80(135.76,141.89) | -1.98(-2.27,-1.68) | 238.68(235.17,242.24) | 138.80(135.76,141.89) | -1.98(-2.27,-1.68) |
| Sri Lanka | female | 74.89(72.13,77.73) | 60.63(58.27,63.07) | -0.84(-0.91,-0.77) | 74.89(72.13,77.73) | 60.63(58.27,63.07) | -0.84(-0.91,-0.77) |
| Sudan | female | 62.96(60.50,65.51) | 59.52(57.96,61.11) | -0.17(-0.27,-0.08) | 62.96(60.50,65.51) | 59.52(57.96,61.11) | -0.17(-0.27,-0.08) |
| Suriname | female | 153.32(127.49,183.41) | 136.72(115.49,160.76) | -0.44(-0.68,-0.20) | 153.32(127.49,183.41) | 136.72(115.49,160.76) | -0.44(-0.68,-0.20) |
| Sweden | female | 324.37(314.96,334.00) | 121.53(115.93,127.34) | -3.29(-3.45,-3.14) | 324.37(314.96,334.00) | 121.53(115.93,127.34) | -3.29(-3.45,-3.14) |
| Switzerland | female | 317.91(307.66,328.43) | 139.42(132.79,146.32) | -2.77(-2.94,-2.60) | 317.91(307.66,328.43) | 139.42(132.79,146.32) | -2.77(-2.94,-2.60) |
| Syrian Arab Republic | female | 67.58(64.24,71.07) | 52.24(49.44,55.18) | -0.84(-0.89,-0.79) | 67.58(64.24,71.07) | 52.24(49.44,55.18) | -0.84(-0.89,-0.79) |
| Taiwan (Province of China) | female | 166.62(162.86,170.45) | 150.35(146.21,154.60) | -0.61(-0.76,-0.47) | 166.62(162.86,170.45) | 150.35(146.21,154.60) | -0.61(-0.76,-0.47) |
| Tajikistan | female | 55.19(50.78,59.93) | 43.19(40.39,46.13) | -0.76(-0.90,-0.62) | 55.19(50.78,59.93) | 43.19(40.39,46.13) | -0.76(-0.90,-0.62) |
| Thailand | female | 133.13(131.17,135.12) | 104.05(102.10,106.04) | -1.00(-1.15,-0.86) | 133.13(131.17,135.12) | 104.05(102.10,106.04) | -1.00(-1.15,-0.86) |
| Timor-Leste | female | 87.40(73.29,103.65) | 64.81(55.74,75.13) | -1.06(-1.23,-0.89) | 87.40(73.29,103.65) | 64.81(55.74,75.13) | -1.06(-1.23,-0.89) |
| Togo | female | 42.32(37.68,47.44) | 35.15(32.42,38.05) | -0.93(-1.05,-0.82) | 42.32(37.68,47.44) | 35.15(32.42,38.05) | -0.93(-1.05,-0.82) |
| Tokelau | female | 74.44(0.00,1653.40) | 69.17(0.00,1781.05) | -0.22(-0.26,-0.19) | 74.44(0.00,1653.40) | 69.17(0.00,1781.05) | -0.22(-0.26,-0.19) |
| Tonga | female | 53.40(24.78,103.96) | 45.85(21.04,88.01) | -0.54(-0.59,-0.49) | 53.40(24.78,103.96) | 45.85(21.04,88.01) | -0.54(-0.59,-0.49) |
| Trinidad and Tobago | female | 98.17(86.27,111.31) | 82.90(71.53,95.62) | -0.28(-0.48,-0.08) | 98.17(86.27,111.31) | 82.90(71.53,95.62) | -0.28(-0.48,-0.08) |
| Tunisia | female | 102.04(97.27,107.00) | 97.19(93.06,101.48) | -0.14(-0.19,-0.09) | 102.04(97.27,107.00) | 97.19(93.06,101.48) | -0.14(-0.19,-0.09) |
| Turkey | female | 65.90(64.44,67.38) | 51.62(50.49,52.77) | -1.04(-1.20,-0.88) | 65.90(64.44,67.38) | 51.62(50.49,52.77) | -1.04(-1.20,-0.88) |
| Turkmenistan | female | 66.54(60.91,72.60) | 52.47(48.01,57.24) | -0.73(-0.94,-0.53) | 66.54(60.91,72.60) | 52.47(48.01,57.24) | -0.73(-0.94,-0.53) |
| Tuvalu | female | 74.78(4.84,337.11) | 70.47(5.94,304.88) | -0.17(-0.19,-0.15) | 74.78(4.84,337.11) | 70.47(5.94,304.88) | -0.17(-0.19,-0.15) |
| Uganda | female | 33.84(31.83,35.95) | 36.53(35.27,37.84) | -0.06(-0.24,0.13) | 33.84(31.83,35.95) | 36.53(35.27,37.84) | -0.06(-0.24,0.13) |
| Ukraine | female | 166.29(163.66,168.95) | 140.35(137.31,143.44) | -0.65(-0.78,-0.52) | 166.29(163.66,168.95) | 140.35(137.31,143.44) | -0.65(-0.78,-0.52) |
| United Arab Emirates | female | 103.41(91.97,115.94) | 103.62(96.94,110.68) | 0.07(0.00,0.14) | 103.41(91.97,115.94) | 103.62(96.94,110.68) | 0.07(0.00,0.14) |
| United Kingdom | female | 202.53(199.76,205.34) | 114.21(112.15,116.31) | -2.03(-2.15,-1.92) | 202.53(199.76,205.34) | 114.21(112.15,116.31) | -2.03(-2.15,-1.92) |
| United Republic of Tanzania | female | 37.48(35.79,39.24) | 32.96(31.95,34.01) | -0.35(-0.41,-0.30) | 37.48(35.79,39.24) | 32.96(31.95,34.01) | -0.35(-0.41,-0.30) |
| United States of America | female | 217.98(216.65,219.32) | 174.25(173.14,175.37) | -0.91(-1.47,-0.34) | 217.98(216.65,219.32) | 174.25(173.14,175.37) | -0.91(-1.47,-0.34) |
| United States Virgin Islands | female | 134.56(89.19,195.45) | 90.77(44.13,167.40) | -1.33(-1.46,-1.19) | 134.56(89.19,195.45) | 90.77(44.13,167.40) | -1.33(-1.46,-1.19) |
| Uruguay | female | 212.30(200.53,224.58) | 236.83(224.60,249.56) | 0.42(0.34,0.49) | 212.30(200.53,224.58) | 236.83(224.60,249.56) | 0.42(0.34,0.49) |
| Uzbekistan | female | 61.71(59.38,64.12) | 66.96(65.00,68.96) | 0.48(0.32,0.65) | 61.71(59.38,64.12) | 66.96(65.00,68.96) | 0.48(0.32,0.65) |
| Vanuatu | female | 80.77(51.57,121.89) | 74.03(54.29,98.98) | -0.33(-0.36,-0.29) | 80.77(51.57,121.89) | 74.03(54.29,98.98) | -0.33(-0.36,-0.29) |
| Venezuela (Bolivarian Republic of) | female | 81.75(79.00,84.58) | 76.63(74.17,79.15) | 0.12(0.03,0.22) | 81.75(79.00,84.58) | 76.63(74.17,79.15) | 0.12(0.03,0.22) |
| Viet Nam | female | 107.60(105.93,109.30) | 109.37(107.84,110.91) | 0.60(0.33,0.87) | 107.60(105.93,109.30) | 109.37(107.84,110.91) | 0.60(0.33,0.87) |
| Yemen | female | 76.61(73.09,80.26) | 58.58(56.78,60.42) | -0.87(-1.01,-0.73) | 76.61(73.09,80.26) | 58.58(56.78,60.42) | -0.87(-1.01,-0.73) |
| Zambia | female | 55.44(51.65,59.47) | 43.20(41.18,45.29) | -1.03(-1.13,-0.94) | 55.44(51.65,59.47) | 43.20(41.18,45.29) | -1.03(-1.13,-0.94) |
| Zimbabwe | female | 36.17(33.55,38.95) | 29.18(27.36,31.09) | -0.48(-0.79,-0.17) | 36.17(33.55,38.95) | 29.18(27.36,31.09) | -0.48(-0.79,-0.17) |
| Afghanistan | male | 97.31(91.40,103.61) | 106.53(103.92,109.20) | 0.73(0.55,0.91) | 97.31(91.40,103.61) | 106.53(103.92,109.20) | 0.73(0.55,0.91) |
| Albania | male | 339.96(326.68,353.67) | 344.14(327.91,360.99) | 0.12(-0.01,0.25) | 339.96(326.68,353.67) | 344.14(327.91,360.99) | 0.12(-0.01,0.25) |
| Algeria | male | 171.15(167.59,174.78) | 134.23(131.74,136.74) | -0.68(-0.73,-0.63) | 171.15(167.59,174.78) | 134.23(131.74,136.74) | -0.68(-0.73,-0.63) |
| American Samoa | male | 88.88(40.66,173.05) | 76.23(29.66,163.83) | -0.50(-0.62,-0.37) | 88.88(40.66,173.05) | 76.23(29.66,163.83) | -0.50(-0.62,-0.37) |
| Andorra | male | 674.62(535.63,841.51) | 530.37(408.94,678.80) | -0.71(-0.73,-0.68) | 674.62(535.63,841.51) | 530.37(408.94,678.80) | -0.71(-0.73,-0.68) |
| Angola | male | 67.14(63.58,70.86) | 73.71(71.51,75.96) | 0.51(0.39,0.63) | 67.14(63.58,70.86) | 73.71(71.51,75.96) | 0.51(0.39,0.63) |
| Antigua and Barbuda | male | 269.46(186.65,378.77) | 163.73(108.53,238.14) | -1.88(-2.08,-1.69) | 269.46(186.65,378.77) | 163.73(108.53,238.14) | -1.88(-2.08,-1.69) |
| Argentina | male | 467.00(461.58,472.46) | 414.14(409.86,418.46) | -0.22(-0.43,-0.01) | 467.00(461.58,472.46) | 414.14(409.86,418.46) | -0.22(-0.43,-0.01) |
| Armenia | male | 100.05(92.80,107.73) | 140.81(130.55,151.72) | 1.68(1.38,1.99) | 100.05(92.80,107.73) | 140.81(130.55,151.72) | 1.68(1.38,1.99) |
| Australia | male | 442.58(435.45,449.80) | 244.88(240.12,249.73) | -1.81(-1.91,-1.71) | 442.58(435.45,449.80) | 244.88(240.12,249.73) | -1.81(-1.91,-1.71) |
| Austria | male | 683.77(670.34,697.42) | 367.14(356.92,377.60) | -1.93(-1.99,-1.87) | 683.77(670.34,697.42) | 367.14(356.92,377.60) | -1.93(-1.99,-1.87) |
| Azerbaijan | male | 118.69(113.33,124.27) | 96.05(91.81,100.45) | -0.67(-0.74,-0.60) | 118.69(113.33,124.27) | 96.05(91.81,100.45) | -0.67(-0.74,-0.60) |
| Bahamas | male | 334.96(289.72,385.85) | 253.06(218.46,291.61) | -0.97(-1.08,-0.86) | 334.96(289.72,385.85) | 253.06(218.46,291.61) | -0.97(-1.08,-0.86) |
| Bahrain | male | 152.05(131.54,175.36) | 128.86(117.91,140.66) | -0.53(-0.56,-0.50) | 152.05(131.54,175.36) | 128.86(117.91,140.66) | -0.53(-0.56,-0.50) |
| Bangladesh | male | 122.88(121.39,124.38) | 99.49(98.42,100.57) | -0.53(-0.62,-0.43) | 122.88(121.39,124.38) | 99.49(98.42,100.57) | -0.53(-0.62,-0.43) |
| Barbados | male | 260.57(219.29,307.48) | 195.59(158.13,239.43) | -1.15(-1.34,-0.95) | 260.57(219.29,307.48) | 195.59(158.13,239.43) | -1.15(-1.34,-0.95) |
| Belarus | male | 445.31(435.84,454.94) | 261.69(253.02,270.61) | -1.82(-1.99,-1.65) | 445.31(435.84,454.94) | 261.69(253.02,270.61) | -1.82(-1.99,-1.65) |
| Belgium | male | 675.39(663.36,687.59) | 440.55(430.58,450.71) | -1.58(-1.70,-1.47) | 675.39(663.36,687.59) | 440.55(430.58,450.71) | -1.58(-1.70,-1.47) |
| Belize | male | 765.09(678.22,861.32) | 558.46(511.51,608.76) | -1.64(-1.91,-1.38) | 765.09(678.22,861.32) | 558.46(511.51,608.76) | -1.64(-1.91,-1.38) |
| Benin | male | 66.87(61.16,73.00) | 53.34(50.48,56.34) | -1.08(-1.22,-0.94) | 66.87(61.16,73.00) | 53.34(50.48,56.34) | -1.08(-1.22,-0.94) |
| Bermuda | male | 416.99(309.27,553.41) | 238.21(143.77,373.90) | -1.81(-1.84,-1.77) | 416.99(309.27,553.41) | 238.21(143.77,373.90) | -1.81(-1.84,-1.77) |
| Bhutan | male | 127.47(109.36,148.15) | 106.37(91.80,122.68) | -0.61(-0.71,-0.51) | 127.47(109.36,148.15) | 106.37(91.80,122.68) | -0.61(-0.71,-0.51) |
| Bolivia (Plurinational State of) | male | 133.26(126.80,139.99) | 118.84(114.59,123.22) | -0.17(-0.33,-0.01) | 133.26(126.80,139.99) | 118.84(114.59,123.22) | -0.17(-0.33,-0.01) |
| Bosnia and Herzegovina | male | 296.85(286.15,307.87) | 306.68(291.28,322.72) | 0.31(0.23,0.39) | 296.85(286.15,307.87) | 306.68(291.28,322.72) | 0.31(0.23,0.39) |
| Botswana | male | 75.77(64.96,88.06) | 80.28(72.80,88.34) | -0.11(-0.40,0.19) | 75.77(64.96,88.06) | 80.28(72.80,88.34) | -0.11(-0.40,0.19) |
| Brazil | male | 294.39(292.48,296.31) | 313.63(311.93,315.34) | 0.10(-0.24,0.45) | 294.39(292.48,296.31) | 313.63(311.93,315.34) | 0.10(-0.24,0.45) |
| Brunei Darussalam | male | 1032.75(955.43,1114.84) | 621.41(574.67,671.19) | -1.85(-2.14,-1.56) | 1032.75(955.43,1114.84) | 621.41(574.67,671.19) | -1.85(-2.14,-1.56) |
| Bulgaria | male | 427.79(417.30,438.47) | 382.14(369.24,395.39) | -0.34(-0.43,-0.25) | 427.79(417.30,438.47) | 382.14(369.24,395.39) | -0.34(-0.43,-0.25) |
| Burkina Faso | male | 57.98(54.01,62.19) | 74.02(71.34,76.78) | 0.93(0.88,0.99) | 57.98(54.01,62.19) | 74.02(71.34,76.78) | 0.93(0.88,0.99) |
| Burundi | male | 121.66(114.93,128.72) | 92.33(88.68,96.09) | -1.14(-1.22,-1.06) | 121.66(114.93,128.72) | 92.33(88.68,96.09) | -1.14(-1.22,-1.06) |
| Cabo Verde | male | 58.46(39.58,85.30) | 60.74(48.04,75.89) | 0.08(-0.15,0.32) | 58.46(39.58,85.30) | 60.74(48.04,75.89) | 0.08(-0.15,0.32) |
| Cambodia | male | 158.86(153.01,164.90) | 154.58(150.56,158.67) | -0.10(-0.12,-0.07) | 158.86(153.01,164.90) | 154.58(150.56,158.67) | -0.10(-0.12,-0.07) |
| Cameroon | male | 77.26(73.18,81.53) | 89.88(87.53,92.28) | 0.43(0.36,0.50) | 77.26(73.18,81.53) | 89.88(87.53,92.28) | 0.43(0.36,0.50) |
| Canada | male | 486.03(479.97,492.14) | 286.87(282.48,291.32) | -1.50(-1.60,-1.40) | 486.03(479.97,492.14) | 286.87(282.48,291.32) | -1.50(-1.60,-1.40) |
| Central African Republic | male | 63.08(56.33,70.51) | 73.98(68.83,79.43) | 0.53(0.47,0.59) | 63.08(56.33,70.51) | 73.98(68.83,79.43) | 0.53(0.47,0.59) |
| Chad | male | 45.60(41.42,50.13) | 55.47(52.75,58.30) | 0.66(0.54,0.79) | 45.60(41.42,50.13) | 55.47(52.75,58.30) | 0.66(0.54,0.79) |
| Chile | male | 373.47(366.39,380.65) | 528.76(521.18,536.43) | 0.58(0.33,0.84) | 373.47(366.39,380.65) | 528.76(521.18,536.43) | 0.58(0.33,0.84) |
| China | male | 329.98(329.31,330.66) | 376.96(376.16,377.75) | 0.27(0.08,0.47) | 329.98(329.31,330.66) | 376.96(376.16,377.75) | 0.27(0.08,0.47) |
| Colombia | male | 260.79(256.98,264.64) | 311.90(308.46,315.36) | 0.04(-0.31,0.39) | 260.79(256.98,264.64) | 311.90(308.46,315.36) | 0.04(-0.31,0.39) |
| Comoros | male | 86.97(67.98,110.37) | 72.70(59.94,87.47) | -0.75(-0.87,-0.63) | 86.97(67.98,110.37) | 72.70(59.94,87.47) | -0.75(-0.87,-0.63) |
| Congo | male | 61.10(54.13,68.82) | 59.04(54.56,63.80) | -0.23(-0.29,-0.16) | 61.10(54.13,68.82) | 59.04(54.56,63.80) | -0.23(-0.29,-0.16) |
| Cook Islands | male | 118.25(36.36,297.71) | 147.24(40.01,383.83) | 0.88(0.81,0.96) | 118.25(36.36,297.71) | 147.24(40.01,383.83) | 0.88(0.81,0.96) |
| Costa Rica | male | 374.28(359.44,389.62) | 262.25(251.83,273.00) | -1.69(-1.89,-1.48) | 374.28(359.44,389.62) | 262.25(251.83,273.00) | -1.69(-1.89,-1.48) |
| Croatia | male | 457.14(443.14,471.48) | 404.46(388.39,421.05) | -0.29(-0.91,0.34) | 457.14(443.14,471.48) | 404.46(388.39,421.05) | -0.29(-0.91,0.34) |
| Cuba | male | 858.39(846.81,870.09) | 253.82(246.54,261.26) | -4.72(-5.05,-4.38) | 858.39(846.81,870.09) | 253.82(246.54,261.26) | -4.72(-5.05,-4.38) |
| Cyprus | male | 798.71(754.85,844.50) | 534.50(503.65,566.91) | -1.67(-1.87,-1.46) | 798.71(754.85,844.50) | 534.50(503.65,566.91) | -1.67(-1.87,-1.46) |
| Czechia | male | 579.10(568.15,590.21) | 352.79(342.86,362.96) | -2.13(-2.54,-1.72) | 579.10(568.15,590.21) | 352.79(342.86,362.96) | -2.13(-2.54,-1.72) |
| Côte d'Ivoire | male | 59.47(56.40,62.69) | 60.01(58.01,62.07) | -0.26(-0.38,-0.15) | 59.47(56.40,62.69) | 60.01(58.01,62.07) | -0.26(-0.38,-0.15) |
| Democratic People's Republic of Korea | male | 259.06(254.03,264.16) | 340.99(335.94,346.09) | 0.91(0.85,0.97) | 259.06(254.03,264.16) | 340.99(335.94,346.09) | 0.91(0.85,0.97) |
| Democratic Republic of the Congo | male | 71.67(69.71,73.68) | 61.87(60.74,63.03) | -0.72(-0.80,-0.64) | 71.67(69.71,73.68) | 61.87(60.74,63.03) | -0.72(-0.80,-0.64) |
| Denmark | male | 696.96(680.21,714.03) | 335.32(323.49,347.49) | -2.53(-2.66,-2.40) | 696.96(680.21,714.03) | 335.32(323.49,347.49) | -2.53(-2.66,-2.40) |
| Djibouti | male | 77.59(60.50,98.58) | 74.90(65.27,85.57) | -0.31(-0.44,-0.17) | 77.59(60.50,98.58) | 74.90(65.27,85.57) | -0.31(-0.44,-0.17) |
| Dominica | male | 357.99(269.34,469.19) | 272.92(191.41,377.57) | -1.14(-1.43,-0.85) | 357.99(269.34,469.19) | 272.92(191.41,377.57) | -1.14(-1.43,-0.85) |
| Dominican Republic | male | 277.51(269.08,286.16) | 267.30(260.65,274.07) | -0.14(-0.35,0.08) | 277.51(269.08,286.16) | 267.30(260.65,274.07) | -0.14(-0.35,0.08) |
| Ecuador | male | 191.01(184.99,197.18) | 276.64(271.28,282.07) | 1.82(1.52,2.11) | 191.01(184.99,197.18) | 276.64(271.28,282.07) | 1.82(1.52,2.11) |
| Egypt | male | 133.51(131.39,135.66) | 138.08(136.52,139.66) | 0.34(0.21,0.47) | 133.51(131.39,135.66) | 138.08(136.52,139.66) | 0.34(0.21,0.47) |
| El Salvador | male | 221.65(212.50,231.13) | 195.78(188.03,203.78) | -0.54(-0.75,-0.32) | 221.65(212.50,231.13) | 195.78(188.03,203.78) | -0.54(-0.75,-0.32) |
| Equatorial Guinea | male | 57.85(41.19,79.70) | 66.34(58.47,75.12) | 0.73(0.45,1.01) | 57.85(41.19,79.70) | 66.34(58.47,75.12) | 0.73(0.45,1.01) |
| Eritrea | male | 97.75(90.11,105.94) | 97.30(92.27,102.54) | -0.24(-0.41,-0.08) | 97.75(90.11,105.94) | 97.30(92.27,102.54) | -0.24(-0.41,-0.08) |
| Estonia | male | 458.73(434.02,484.50) | 184.51(165.42,205.34) | -3.51(-3.75,-3.28) | 458.73(434.02,484.50) | 184.51(165.42,205.34) | -3.51(-3.75,-3.28) |
| Eswatini | male | 81.79(66.76,99.58) | 107.47(94.85,121.37) | 0.79(0.40,1.18) | 81.79(66.76,99.58) | 107.47(94.85,121.37) | 0.79(0.40,1.18) |
| Ethiopia | male | 72.99(71.19,74.83) | 46.90(46.01,47.81) | -1.44(-1.49,-1.40) | 72.99(71.19,74.83) | 46.90(46.01,47.81) | -1.44(-1.49,-1.40) |
| Fiji | male | 75.60(62.87,90.30) | 68.32(56.84,81.47) | -0.23(-0.33,-0.13) | 75.60(62.87,90.30) | 68.32(56.84,81.47) | -0.23(-0.33,-0.13) |
| Finland | male | 565.77(549.81,582.09) | 320.12(307.84,332.79) | -1.92(-2.20,-1.63) | 565.77(549.81,582.09) | 320.12(307.84,332.79) | -1.92(-2.20,-1.63) |
| France | male | 606.08(601.46,610.73) | 447.13(442.95,451.34) | -0.90(-1.08,-0.72) | 606.08(601.46,610.73) | 447.13(442.95,451.34) | -0.90(-1.08,-0.72) |
| Gabon | male | 71.74(60.33,84.79) | 78.08(69.14,87.92) | 0.18(0.11,0.24) | 71.74(60.33,84.79) | 78.08(69.14,87.92) | 0.18(0.11,0.24) |
| Gambia | male | 52.13(42.02,64.24) | 53.03(46.64,60.12) | -0.12(-0.21,-0.03) | 52.13(42.02,64.24) | 53.03(46.64,60.12) | -0.12(-0.21,-0.03) |
| Georgia | male | 148.37(141.06,155.96) | 142.87(132.91,153.41) | -0.11(-0.28,0.06) | 148.37(141.06,155.96) | 142.87(132.91,153.41) | -0.11(-0.28,0.06) |
| Germany | male | 664.06(659.70,668.44) | 363.19(359.80,366.60) | -2.19(-2.28,-2.10) | 664.06(659.70,668.44) | 363.19(359.80,366.60) | -2.19(-2.28,-2.10) |
| Ghana | male | 73.43(70.24,76.74) | 104.32(101.92,106.78) | 1.33(1.28,1.39) | 73.43(70.24,76.74) | 104.32(101.92,106.78) | 1.33(1.28,1.39) |
| Greece | male | 561.07(550.41,571.88) | 487.92(476.04,500.02) | -0.41(-0.57,-0.24) | 561.07(550.41,571.88) | 487.92(476.04,500.02) | -0.41(-0.57,-0.24) |
| Greenland | male | 297.87(210.80,413.74) | 190.86(114.44,302.01) | -1.48(-1.55,-1.40) | 297.87(210.80,413.74) | 190.86(114.44,302.01) | -1.48(-1.55,-1.40) |
| Grenada | male | 296.61(219.63,394.01) | 202.46(146.39,274.03) | -1.17(-1.24,-1.09) | 296.61(219.63,394.01) | 202.46(146.39,274.03) | -1.17(-1.24,-1.09) |
| Guam | male | 100.62(69.98,140.70) | 86.30(55.84,127.68) | -0.53(-0.62,-0.43) | 100.62(69.98,140.70) | 86.30(55.84,127.68) | -0.53(-0.62,-0.43) |
| Guatemala | male | 204.06(196.67,211.68) | 231.13(225.95,236.42) | 0.51(0.39,0.63) | 204.06(196.67,211.68) | 231.13(225.95,236.42) | 0.51(0.39,0.63) |
| Guinea | male | 51.75(47.23,56.62) | 62.08(58.90,65.40) | 0.57(0.48,0.67) | 51.75(47.23,56.62) | 62.08(58.90,65.40) | 0.57(0.48,0.67) |
| Guinea-Bissau | male | 96.86(82.57,113.19) | 96.48(87.02,106.77) | -0.04(-0.11,0.02) | 96.86(82.57,113.19) | 96.48(87.02,106.77) | -0.04(-0.11,0.02) |
| Guyana | male | 405.35(375.27,437.38) | 448.31(415.42,483.29) | 0.26(0.08,0.45) | 405.35(375.27,437.38) | 448.31(415.42,483.29) | 0.26(0.08,0.45) |
| Haiti | male | 345.96(335.32,356.86) | 296.89(290.40,303.48) | -0.30(-0.51,-0.09) | 345.96(335.32,356.86) | 296.89(290.40,303.48) | -0.30(-0.51,-0.09) |
| Honduras | male | 218.30(208.31,228.67) | 179.17(173.48,185.01) | -0.93(-1.21,-0.64) | 218.30(208.31,228.67) | 179.17(173.48,185.01) | -0.93(-1.21,-0.64) |
| Hungary | male | 775.90(763.14,788.82) | 310.86(301.49,320.47) | -3.40(-3.56,-3.24) | 775.90(763.14,788.82) | 310.86(301.49,320.47) | -3.40(-3.56,-3.24) |
| Iceland | male | 482.42(424.65,545.98) | 255.21(215.94,299.91) | -2.17(-2.30,-2.03) | 482.42(424.65,545.98) | 255.21(215.94,299.91) | -2.17(-2.30,-2.03) |
| India | male | 167.18(166.57,167.79) | 202.97(202.47,203.47) | 0.56(0.34,0.78) | 167.18(166.57,167.79) | 202.97(202.47,203.47) | 0.56(0.34,0.78) |
| Indonesia | male | 164.05(162.78,165.33) | 124.24(123.33,125.15) | -1.04(-1.17,-0.91) | 164.05(162.78,165.33) | 124.24(123.33,125.15) | -1.04(-1.17,-0.91) |
| Iran (Islamic Republic of) | male | 225.10(222.29,227.93) | 236.62(234.22,239.04) | 0.09(-0.05,0.23) | 225.10(222.29,227.93) | 236.62(234.22,239.04) | 0.09(-0.05,0.23) |
| Iraq | male | 114.85(111.42,118.38) | 105.68(103.58,107.81) | -0.15(-0.22,-0.09) | 114.85(111.42,118.38) | 105.68(103.58,107.81) | -0.15(-0.22,-0.09) |
| Ireland | male | 788.58(768.18,809.40) | 303.66(291.35,316.36) | -3.12(-3.21,-3.04) | 788.58(768.18,809.40) | 303.66(291.35,316.36) | -3.12(-3.21,-3.04) |
| Israel | male | 435.90(422.84,449.27) | 357.62(348.65,366.76) | -0.63(-0.73,-0.54) | 435.90(422.84,449.27) | 357.62(348.65,366.76) | -0.63(-0.73,-0.54) |
| Italy | male | 788.74(783.43,794.08) | 467.97(463.16,472.83) | -2.00(-2.15,-1.86) | 788.74(783.43,794.08) | 467.97(463.16,472.83) | -2.00(-2.15,-1.86) |
| Jamaica | male | 178.87(167.09,191.34) | 138.08(128.79,147.89) | -0.83(-0.93,-0.72) | 178.87(167.09,191.34) | 138.08(128.79,147.89) | -0.83(-0.93,-0.72) |
| Japan | male | 652.44(649.13,655.76) | 284.22(281.58,286.87) | -2.99(-3.09,-2.89) | 652.44(649.13,655.76) | 284.22(281.58,286.87) | -2.99(-3.09,-2.89) |
| Jordan | male | 120.65(113.06,128.70) | 99.10(95.53,102.77) | -0.60(-0.70,-0.50) | 120.65(113.06,128.70) | 99.10(95.53,102.77) | -0.60(-0.70,-0.50) |
| Kazakhstan | male | 143.87(139.86,147.96) | 123.85(120.08,127.72) | -0.27(-0.41,-0.13) | 143.87(139.86,147.96) | 123.85(120.08,127.72) | -0.27(-0.41,-0.13) |
| Kenya | male | 66.91(64.42,69.49) | 69.48(67.88,71.11) | 0.00(-0.27,0.26) | 66.91(64.42,69.49) | 69.48(67.88,71.11) | 0.00(-0.27,0.26) |
| Kiribati | male | 100.14(55.77,168.88) | 108.81(71.31,159.65) | 0.11(-0.13,0.34) | 100.14(55.77,168.88) | 108.81(71.31,159.65) | 0.11(-0.13,0.34) |
| Kuwait | male | 179.67(167.18,192.97) | 134.67(126.97,142.78) | -1.00(-1.06,-0.95) | 179.67(167.18,192.97) | 134.67(126.97,142.78) | -1.00(-1.06,-0.95) |
| Kyrgyzstan | male | 135.45(127.96,143.28) | 105.44(100.04,111.06) | -0.63(-0.78,-0.48) | 135.45(127.96,143.28) | 105.44(100.04,111.06) | -0.63(-0.78,-0.48) |
| Lao People's Democratic Republic | male | 169.65(160.44,179.29) | 141.22(135.49,147.13) | -0.54(-0.59,-0.48) | 169.65(160.44,179.29) | 141.22(135.49,147.13) | -0.54(-0.59,-0.48) |
| Latvia | male | 615.25(593.04,638.10) | 289.35(268.73,311.25) | -2.88(-3.05,-2.70) | 615.25(593.04,638.10) | 289.35(268.73,311.25) | -2.88(-3.05,-2.70) |
| Lebanon | male | 156.35(146.05,167.26) | 124.07(117.62,130.80) | -0.87(-0.95,-0.79) | 156.35(146.05,167.26) | 124.07(117.62,130.80) | -0.87(-0.95,-0.79) |
| Lesotho | male | 67.08(56.81,78.78) | 91.83(82.73,101.70) | 1.03(0.87,1.19) | 67.08(56.81,78.78) | 91.83(82.73,101.70) | 1.03(0.87,1.19) |
| Liberia | male | 51.45(44.99,58.62) | 49.27(45.20,53.63) | -0.35(-0.43,-0.27) | 51.45(44.99,58.62) | 49.27(45.20,53.63) | -0.35(-0.43,-0.27) |
| Libya | male | 168.38(159.97,177.14) | 133.52(127.75,139.48) | -0.58(-0.68,-0.49) | 168.38(159.97,177.14) | 133.52(127.75,139.48) | -0.58(-0.68,-0.49) |
| Lithuania | male | 721.19(701.37,741.43) | 284.19(267.69,301.53) | -3.41(-3.86,-2.95) | 721.19(701.37,741.43) | 284.19(267.69,301.53) | -3.41(-3.86,-2.95) |
| Luxembourg | male | 625.84(567.26,689.30) | 407.46(368.97,449.16) | -1.46(-1.58,-1.33) | 625.84(567.26,689.30) | 407.46(368.97,449.16) | -1.46(-1.58,-1.33) |
| Madagascar | male | 88.48(84.62,92.48) | 72.20(70.00,74.45) | -0.58(-0.67,-0.49) | 88.48(84.62,92.48) | 72.20(70.00,74.45) | -0.58(-0.67,-0.49) |
| Malawi | male | 73.19(69.28,77.28) | 79.40(76.59,82.29) | 0.13(-0.08,0.35) | 73.19(69.28,77.28) | 79.40(76.59,82.29) | 0.13(-0.08,0.35) |
| Malaysia | male | 205.30(200.75,209.93) | 184.94(181.79,188.13) | -0.56(-0.68,-0.44) | 205.30(200.75,209.93) | 184.94(181.79,188.13) | -0.56(-0.68,-0.44) |
| Maldives | male | 133.94(100.18,177.38) | 79.77(64.56,98.15) | -1.77(-1.84,-1.71) | 133.94(100.18,177.38) | 79.77(64.56,98.15) | -1.77(-1.84,-1.71) |
| Mali | male | 46.38(42.83,50.15) | 48.54(46.41,50.76) | 0.10(0.00,0.20) | 46.38(42.83,50.15) | 48.54(46.41,50.76) | 0.10(0.00,0.20) |
| Malta | male | 458.11(408.42,512.41) | 350.48(304.66,401.80) | -0.85(-1.02,-0.68) | 458.11(408.42,512.41) | 350.48(304.66,401.80) | -0.85(-1.02,-0.68) |
| Marshall Islands | male | 69.03(25.25,155.67) | 62.65(26.40,126.17) | -0.35(-0.40,-0.30) | 69.03(25.25,155.67) | 62.65(26.40,126.17) | -0.35(-0.40,-0.30) |
| Mauritania | male | 95.74(85.92,106.45) | 75.24(69.32,81.57) | -0.80(-0.87,-0.73) | 95.74(85.92,106.45) | 75.24(69.32,81.57) | -0.80(-0.87,-0.73) |
| Mauritius | male | 104.39(92.16,117.83) | 119.78(105.99,134.93) | 0.50(0.41,0.60) | 104.39(92.16,117.83) | 119.78(105.99,134.93) | 0.50(0.41,0.60) |
| Mexico | male | 388.42(385.46,391.40) | 269.50(267.49,271.53) | -0.47(-0.87,-0.06) | 388.42(385.46,391.40) | 269.50(267.49,271.53) | -0.47(-0.87,-0.06) |
| Micronesia (Federated States of) | male | 87.70(51.43,141.04) | 90.75(55.19,141.84) | 0.03(-0.01,0.06) | 87.70(51.43,141.04) | 90.75(55.19,141.84) | 0.03(-0.01,0.06) |
| Monaco | male | 559.89(355.14,852.01) | 440.52(269.07,683.02) | -0.63(-0.70,-0.57) | 559.89(355.14,852.01) | 440.52(269.07,683.02) | -0.63(-0.70,-0.57) |
| Mongolia | male | 106.30(96.88,116.52) | 113.74(105.39,122.60) | 0.47(0.37,0.57) | 106.30(96.88,116.52) | 113.74(105.39,122.60) | 0.47(0.37,0.57) |
| Montenegro | male | 432.96(397.85,470.39) | 343.73(308.82,381.64) | -0.86(-1.00,-0.73) | 432.96(397.85,470.39) | 343.73(308.82,381.64) | -0.86(-1.00,-0.73) |
| Morocco | male | 136.56(133.39,139.80) | 122.89(120.37,125.45) | -0.34(-0.43,-0.26) | 136.56(133.39,139.80) | 122.89(120.37,125.45) | -0.34(-0.43,-0.26) |
| Mozambique | male | 85.15(81.28,89.17) | 115.69(112.87,118.58) | 1.10(0.99,1.21) | 85.15(81.28,89.17) | 115.69(112.87,118.58) | 1.10(0.99,1.21) |
| Myanmar | male | 146.09(143.54,148.69) | 122.67(120.62,124.75) | -0.63(-0.68,-0.59) | 146.09(143.54,148.69) | 122.67(120.62,124.75) | -0.63(-0.68,-0.59) |
| Namibia | male | 75.10(64.90,86.62) | 86.69(78.78,95.22) | 0.25(0.02,0.48) | 75.10(64.90,86.62) | 86.69(78.78,95.22) | 0.25(0.02,0.48) |
| Nauru | male | 134.35(24.73,428.31) | 158.37(40.01,434.67) | 0.43(0.33,0.52) | 134.35(24.73,428.31) | 158.37(40.01,434.67) | 0.43(0.33,0.52) |
| Nepal | male | 142.98(139.02,147.02) | 109.93(107.31,112.61) | -0.85(-0.90,-0.79) | 142.98(139.02,147.02) | 109.93(107.31,112.61) | -0.85(-0.90,-0.79) |
| Netherlands | male | 799.11(788.82,809.49) | 328.80(321.86,335.85) | -3.06(-3.18,-2.95) | 799.11(788.82,809.49) | 328.80(321.86,335.85) | -3.06(-3.18,-2.95) |
| New Zealand | male | 716.80(696.95,737.09) | 361.87(349.46,374.62) | -3.98(-4.73,-3.22) | 716.80(696.95,737.09) | 361.87(349.46,374.62) | -3.98(-4.73,-3.22) |
| Nicaragua | male | 179.06(169.23,189.37) | 185.05(178.08,192.24) | 0.25(0.15,0.35) | 179.06(169.23,189.37) | 185.05(178.08,192.24) | 0.25(0.15,0.35) |
| Niger | male | 50.71(46.91,54.76) | 54.91(52.66,57.23) | 0.45(0.35,0.56) | 50.71(46.91,54.76) | 54.91(52.66,57.23) | 0.45(0.35,0.56) |
| Nigeria | male | 52.30(51.21,53.41) | 51.82(51.11,52.53) | 0.01(-0.14,0.17) | 52.30(51.21,53.41) | 51.82(51.11,52.53) | 0.01(-0.14,0.17) |
| Niue | male | 91.24(0.01,1197.02) | 84.73(0.00,1587.30) | -0.44(-0.55,-0.32) | 91.24(0.01,1197.02) | 84.73(0.00,1587.30) | -0.44(-0.55,-0.32) |
| North Macedonia | male | 358.44(340.21,377.40) | 283.37(266.27,301.37) | -0.87(-0.94,-0.79) | 358.44(340.21,377.40) | 283.37(266.27,301.37) | -0.87(-0.94,-0.79) |
| Northern Mariana Islands | male | 89.39(42.91,169.22) | 74.07(28.09,161.29) | -0.75(-0.96,-0.53) | 89.39(42.91,169.22) | 74.07(28.09,161.29) | -0.75(-0.96,-0.53) |
| Norway | male | 537.02(521.19,553.21) | 283.12(272.09,294.49) | -2.12(-2.27,-1.98) | 537.02(521.19,553.21) | 283.12(272.09,294.49) | -2.12(-2.27,-1.98) |
| Oman | male | 172.35(161.02,184.31) | 252.70(242.48,263.29) | 1.78(1.43,2.13) | 172.35(161.02,184.31) | 252.70(242.48,263.29) | 1.78(1.43,2.13) |
| Pakistan | male | 105.22(103.82,106.63) | 125.39(124.41,126.38) | 0.44(0.23,0.64) | 105.22(103.82,106.63) | 125.39(124.41,126.38) | 0.44(0.23,0.64) |
| Palau | male | 111.35(30.74,291.02) | 123.64(35.22,323.76) | 0.33(0.26,0.40) | 111.35(30.74,291.02) | 123.64(35.22,323.76) | 0.33(0.26,0.40) |
| Palestine | male | 108.68(98.41,119.94) | 88.37(82.94,94.09) | -0.84(-0.91,-0.77) | 108.68(98.41,119.94) | 88.37(82.94,94.09) | -0.84(-0.91,-0.77) |
| Panama | male | 439.80(421.92,458.30) | 274.68(263.59,286.12) | -1.87(-2.01,-1.72) | 439.80(421.92,458.30) | 274.68(263.59,286.12) | -1.87(-2.01,-1.72) |
| Papua New Guinea | male | 71.00(65.45,76.93) | 59.24(56.06,62.55) | -0.89(-1.00,-0.79) | 71.00(65.45,76.93) | 59.24(56.06,62.55) | -0.89(-1.00,-0.79) |
| Paraguay | male | 208.48(198.55,218.81) | 212.53(205.36,219.90) | 0.20(0.06,0.33) | 208.48(198.55,218.81) | 212.53(205.36,219.90) | 0.20(0.06,0.33) |
| Peru | male | 150.00(146.35,153.72) | 164.77(161.89,167.69) | 0.72(0.59,0.86) | 150.00(146.35,153.72) | 164.77(161.89,167.69) | 0.72(0.59,0.86) |
| Philippines | male | 120.33(118.44,122.25) | 96.10(94.87,97.35) | -0.69(-0.82,-0.57) | 120.33(118.44,122.25) | 96.10(94.87,97.35) | -0.69(-0.82,-0.57) |
| Poland | male | 612.14(606.32,618.01) | 425.87(420.41,431.38) | -1.78(-2.11,-1.44) | 612.14(606.32,618.01) | 425.87(420.41,431.38) | -1.78(-2.11,-1.44) |
| Portugal | male | 934.59(920.90,948.43) | 426.56(415.93,437.41) | -2.78(-2.92,-2.65) | 934.59(920.90,948.43) | 426.56(415.93,437.41) | -2.78(-2.92,-2.65) |
| Puerto Rico | male | 349.50(335.73,363.70) | 264.63(250.67,279.18) | -0.87(-0.90,-0.83) | 349.50(335.73,363.70) | 264.63(250.67,279.18) | -0.87(-0.90,-0.83) |
| Qatar | male | 284.33(255.66,316.05) | 162.87(152.20,174.35) | -1.95(-2.17,-1.74) | 284.33(255.66,316.05) | 162.87(152.20,174.35) | -1.95(-2.17,-1.74) |
| Republic of Korea | male | 1183.07(1176.57,1189.60) | 364.16(359.88,368.49) | -4.65(-5.05,-4.25) | 1183.07(1176.57,1189.60) | 364.16(359.88,368.49) | -4.65(-5.05,-4.25) |
| Republic of Moldova | male | 430.11(416.19,444.40) | 236.99(224.24,250.35) | -2.04(-2.30,-1.79) | 430.11(416.19,444.40) | 236.99(224.24,250.35) | -2.04(-2.30,-1.79) |
| Romania | male | 464.25(457.85,470.74) | 433.90(425.95,441.97) | -0.13(-0.18,-0.08) | 464.25(457.85,470.74) | 433.90(425.95,441.97) | -0.13(-0.18,-0.08) |
| Russian Federation | male | 373.27(371.01,375.53) | 256.87(254.70,259.05) | -1.12(-1.31,-0.94) | 373.27(371.01,375.53) | 256.87(254.70,259.05) | -1.12(-1.31,-0.94) |
| Rwanda | male | 218.43(210.67,226.42) | 140.71(136.35,145.17) | -2.12(-2.36,-1.87) | 218.43(210.67,226.42) | 140.71(136.35,145.17) | -2.12(-2.36,-1.87) |
| Saint Kitts and Nevis | male | 347.36(233.95,500.97) | 243.41(159.92,356.81) | -1.20(-1.34,-1.07) | 347.36(233.95,500.97) | 243.41(159.92,356.81) | -1.20(-1.34,-1.07) |
| Saint Lucia | male | 403.87(331.66,489.06) | 275.14(221.57,338.30) | -1.54(-1.68,-1.39) | 403.87(331.66,489.06) | 275.14(221.57,338.30) | -1.54(-1.68,-1.39) |
| Saint Vincent and the Grenadines | male | 298.66(231.83,381.35) | 195.91(140.70,266.00) | -1.55(-1.68,-1.43) | 298.66(231.83,381.35) | 195.91(140.70,266.00) | -1.55(-1.68,-1.43) |
| Samoa | male | 83.83(55.90,123.11) | 90.46(63.63,125.55) | 0.17(0.10,0.25) | 83.83(55.90,123.11) | 90.46(63.63,125.55) | 0.17(0.10,0.25) |
| San Marino | male | 783.14(548.49,1086.27) | 517.62(327.58,778.89) | -1.31(-1.36,-1.25) | 783.14(548.49,1086.27) | 517.62(327.58,778.89) | -1.31(-1.36,-1.25) |
| Sao Tome and Principe | male | 52.54(25.11,102.06) | 56.04(36.41,82.89) | 0.01(-0.09,0.10) | 52.54(25.11,102.06) | 56.04(36.41,82.89) | 0.01(-0.09,0.10) |
| Saudi Arabia | male | 150.45(146.62,154.37) | 144.53(142.12,146.97) | 0.18(0.08,0.29) | 150.45(146.62,154.37) | 144.53(142.12,146.97) | 0.18(0.08,0.29) |
| Senegal | male | 51.14(47.28,55.27) | 51.97(49.48,54.57) | -0.02(-0.11,0.07) | 51.14(47.28,55.27) | 51.97(49.48,54.57) | -0.02(-0.11,0.07) |
| Serbia | male | 666.04(654.19,678.06) | 390.10(380.18,400.22) | -1.74(-1.83,-1.64) | 666.04(654.19,678.06) | 390.10(380.18,400.22) | -1.74(-1.83,-1.64) |
| Seychelles | male | 140.87(88.54,215.45) | 172.90(120.45,241.95) | 0.44(0.35,0.53) | 140.87(88.54,215.45) | 172.90(120.45,241.95) | 0.44(0.35,0.53) |
| Sierra Leone | male | 61.14(55.62,67.09) | 60.86(57.27,64.63) | -0.01(-0.12,0.09) | 61.14(55.62,67.09) | 60.86(57.27,64.63) | -0.01(-0.12,0.09) |
| Singapore | male | 515.66(499.55,532.17) | 260.04(248.57,271.95) | -2.45(-2.54,-2.35) | 515.66(499.55,532.17) | 260.04(248.57,271.95) | -2.45(-2.54,-2.35) |
| Slovakia | male | 709.58(693.28,726.19) | 366.83(353.53,380.54) | -2.60(-2.79,-2.40) | 709.58(693.28,726.19) | 366.83(353.53,380.54) | -2.60(-2.79,-2.40) |
| Slovenia | male | 701.62(674.90,729.15) | 340.46(318.39,363.75) | -2.25(-2.48,-2.01) | 701.62(674.90,729.15) | 340.46(318.39,363.75) | -2.25(-2.48,-2.01) |
| Solomon Islands | male | 80.54(59.70,107.16) | 97.88(81.85,116.28) | 0.62(0.58,0.66) | 80.54(59.70,107.16) | 97.88(81.85,116.28) | 0.62(0.58,0.66) |
| Somalia | male | 82.69(78.15,87.45) | 83.05(80.30,85.87) | -0.13(-0.21,-0.05) | 82.69(78.15,87.45) | 83.05(80.30,85.87) | -0.13(-0.21,-0.05) |
| South Africa | male | 134.03(131.41,136.70) | 105.47(103.66,107.30) | -0.99(-1.18,-0.81) | 134.03(131.41,136.70) | 105.47(103.66,107.30) | -0.99(-1.18,-0.81) |
| South Sudan | male | 74.11(69.24,79.27) | 62.23(58.40,66.27) | -0.65(-0.80,-0.50) | 74.11(69.24,79.27) | 62.23(58.40,66.27) | -0.65(-0.80,-0.50) |
| Spain | male | 602.14(596.62,607.69) | 356.07(351.29,360.90) | -1.83(-2.14,-1.53) | 602.14(596.62,607.69) | 356.07(351.29,360.90) | -1.83(-2.14,-1.53) |
| Sri Lanka | male | 149.73(145.82,153.71) | 140.17(136.49,143.92) | -0.77(-0.99,-0.55) | 149.73(145.82,153.71) | 140.17(136.49,143.92) | -0.77(-0.99,-0.55) |
| Sudan | male | 113.66(110.21,117.20) | 123.19(120.92,125.49) | 0.37(0.30,0.43) | 113.66(110.21,117.20) | 123.19(120.92,125.49) | 0.37(0.30,0.43) |
| Suriname | male | 496.82(449.72,548.15) | 378.05(341.98,416.95) | -0.84(-0.93,-0.75) | 496.82(449.72,548.15) | 378.05(341.98,416.95) | -0.84(-0.93,-0.75) |
| Sweden | male | 558.08(546.00,570.36) | 275.46(267.33,283.79) | -2.20(-2.27,-2.13) | 558.08(546.00,570.36) | 275.46(267.33,283.79) | -2.20(-2.27,-2.13) |
| Switzerland | male | 655.25(641.02,669.73) | 316.82(307.11,326.79) | -2.40(-2.66,-2.15) | 655.25(641.02,669.73) | 316.82(307.11,326.79) | -2.40(-2.66,-2.15) |
| Syrian Arab Republic | male | 116.60(112.31,121.04) | 97.44(92.78,102.33) | -0.42(-0.53,-0.30) | 116.60(112.31,121.04) | 97.44(92.78,102.33) | -0.42(-0.53,-0.30) |
| Taiwan (Province of China) | male | 226.45(222.15,230.81) | 198.61(193.98,203.33) | -0.58(-0.72,-0.44) | 226.45(222.15,230.81) | 198.61(193.98,203.33) | -0.58(-0.72,-0.44) |
| Tajikistan | male | 100.49(94.51,106.79) | 78.51(74.78,82.39) | -0.73(-0.80,-0.67) | 100.49(94.51,106.79) | 78.51(74.78,82.39) | -0.73(-0.80,-0.67) |
| Thailand | male | 301.46(298.48,304.46) | 195.51(192.79,198.26) | -2.22(-2.56,-1.88) | 301.46(298.48,304.46) | 195.51(192.79,198.26) | -2.22(-2.56,-1.88) |
| Timor-Leste | male | 119.37(103.27,137.47) | 105.35(93.63,118.35) | -0.34(-0.43,-0.25) | 119.37(103.27,137.47) | 105.35(93.63,118.35) | -0.34(-0.43,-0.25) |
| Togo | male | 70.98(64.50,78.01) | 79.57(75.24,84.10) | 0.13(0.03,0.23) | 70.98(64.50,78.01) | 79.57(75.24,84.10) | 0.13(0.03,0.23) |
| Tokelau | male | 66.51(0.00,1697.24) | 63.22(0.00,1760.32) | -0.16(-0.27,-0.05) | 66.51(0.00,1697.24) | 63.22(0.00,1760.32) | -0.16(-0.27,-0.05) |
| Tonga | male | 59.23(28.66,114.53) | 61.84(31.51,111.36) | 0.18(0.09,0.27) | 59.23(28.66,114.53) | 61.84(31.51,111.36) | 0.18(0.09,0.27) |
| Trinidad and Tobago | male | 291.60(270.96,313.46) | 241.71(222.22,262.51) | -0.12(-0.33,0.09) | 291.60(270.96,313.46) | 241.71(222.22,262.51) | -0.12(-0.33,0.09) |
| Tunisia | male | 159.24(153.33,165.33) | 155.22(149.93,160.67) | -0.12(-0.18,-0.07) | 159.24(153.33,165.33) | 155.22(149.93,160.67) | -0.12(-0.18,-0.07) |
| Turkey | male | 130.27(128.25,132.32) | 116.33(114.67,118.02) | -0.42(-0.73,-0.10) | 130.27(128.25,132.32) | 116.33(114.67,118.02) | -0.42(-0.73,-0.10) |
| Turkmenistan | male | 129.90(121.94,138.30) | 101.43(95.58,107.56) | -0.72(-0.80,-0.63) | 129.90(121.94,138.30) | 101.43(95.58,107.56) | -0.72(-0.80,-0.63) |
| Tuvalu | male | 81.07(4.87,385.69) | 94.04(15.54,311.85) | 0.54(0.43,0.65) | 81.07(4.87,385.69) | 94.04(15.54,311.85) | 0.54(0.43,0.65) |
| Uganda | male | 107.65(103.98,111.43) | 141.42(138.83,144.04) | 0.48(0.21,0.75) | 107.65(103.98,111.43) | 141.42(138.83,144.04) | 0.48(0.21,0.75) |
| Ukraine | male | 337.55(333.83,341.31) | 283.78(279.57,288.04) | -0.55(-0.65,-0.46) | 337.55(333.83,341.31) | 283.78(279.57,288.04) | -0.55(-0.65,-0.46) |
| United Arab Emirates | male | 167.24(155.97,179.25) | 189.71(181.98,197.73) | 0.58(0.52,0.64) | 167.24(155.97,179.25) | 189.71(181.98,197.73) | 0.58(0.52,0.64) |
| United Kingdom | male | 543.71(539.19,548.25) | 306.09(302.72,309.48) | -1.98(-2.07,-1.89) | 543.71(539.19,548.25) | 306.09(302.72,309.48) | -1.98(-2.07,-1.89) |
| United Republic of Tanzania | male | 80.92(78.29,83.62) | 73.49(71.89,75.12) | -0.50(-0.57,-0.42) | 80.92(78.29,83.62) | 73.49(71.89,75.12) | -0.50(-0.57,-0.42) |
| United States of America | male | 611.85(609.65,614.05) | 397.24(395.57,398.90) | -1.59(-1.72,-1.47) | 611.85(609.65,614.05) | 397.24(395.57,398.90) | -1.59(-1.72,-1.47) |
| United States Virgin Islands | male | 395.93(311.33,497.18) | 208.46(132.35,314.07) | -2.85(-3.40,-2.30) | 395.93(311.33,497.18) | 208.46(132.35,314.07) | -2.85(-3.40,-2.30) |
| Uruguay | male | 422.58(405.83,439.85) | 419.35(402.99,436.20) | 0.08(-0.05,0.21) | 422.58(405.83,439.85) | 419.35(402.99,436.20) | 0.08(-0.05,0.21) |
| Uzbekistan | male | 127.10(123.73,130.53) | 131.88(129.17,134.65) | 0.35(0.22,0.48) | 127.10(123.73,130.53) | 131.88(129.17,134.65) | 0.35(0.22,0.48) |
| Vanuatu | male | 99.84(66.56,144.96) | 106.96(82.50,136.79) | 0.12(0.08,0.15) | 99.84(66.56,144.96) | 106.96(82.50,136.79) | 0.12(0.08,0.15) |
| Venezuela (Bolivarian Republic of) | male | 343.91(338.23,349.68) | 233.61(229.05,238.25) | -1.12(-1.21,-1.02) | 343.91(338.23,349.68) | 233.61(229.05,238.25) | -1.12(-1.21,-1.02) |
| Viet Nam | male | 167.70(165.54,169.89) | 205.82(203.76,207.90) | 1.19(0.88,1.51) | 167.70(165.54,169.89) | 205.82(203.76,207.90) | 1.19(0.88,1.51) |
| Yemen | male | 134.06(129.28,138.99) | 113.88(111.36,116.44) | -0.43(-0.50,-0.36) | 134.06(129.28,138.99) | 113.88(111.36,116.44) | -0.43(-0.50,-0.36) |
| Zambia | male | 104.96(99.69,110.47) | 129.56(126.02,133.18) | 0.41(0.25,0.58) | 104.96(99.69,110.47) | 129.56(126.02,133.18) | 0.41(0.25,0.58) |
| Zimbabwe | male | 79.96(75.87,84.25) | 89.22(85.84,92.71) | 0.19(-0.14,0.52) | 79.96(75.87,84.25) | 89.22(85.84,92.71) | 0.19(-0.14,0.52) |

Table S6 Cases number, age standardized deaths rate and its trends of cyclist road injuries age 15-39 in 204 countries, 1990-2021

| deaths |  | Deaths No.(95%UI) |  |  | ASDR (per 100000) No.95%UI |  |  |
| --- | --- | --- | --- | --- | --- | --- | --- |
| nation | sex | 1990 | 2021 | 1990-2021 EAPC No.(95%CI) | 1990 | 2021 | 1990-2021 EAPC No.(95%CI) |
| Afghanistan | both | 0.01(0.00,0.30) | 0.03(0.01,0.09) | 4.22(3.79,4.65) | 0.01(0.00,0.30) | 0.03(0.01,0.09) | 4.22(3.79,4.65) |
| Albania | both | 0.16(0.02,0.57) | 0.15(0.01,0.73) | -0.28(-1.23,0.67) | 0.16(0.02,0.57) | 0.15(0.01,0.73) | -0.28(-1.23,0.67) |
| Algeria | both | 0.01(0.00,0.07) | 0.02(0.01,0.06) | 2.27(1.84,2.70) | 0.01(0.00,0.07) | 0.02(0.01,0.06) | 2.27(1.84,2.70) |
| American Samoa | both | 0.01(0.00,23.31) | 0.08(0.00,24.78) | 8.78(6.35,11.25) | 0.01(0.00,23.31) | 0.08(0.00,24.78) | 8.78(6.35,11.25) |
| Andorra | both | 0.72(0.00,21.68) | 0.44(0.00,19.97) | -1.45(-1.89,-1.02) | 0.72(0.00,21.68) | 0.44(0.00,19.97) | -1.45(-1.89,-1.02) |
| Angola | both | 0.24(0.11,0.47) | 0.27(0.18,0.39) | 1.11(0.69,1.53) | 0.24(0.11,0.47) | 0.27(0.18,0.39) | 1.11(0.69,1.53) |
| Antigua and Barbuda | both | 0.71(0.00,17.94) | 0.20(0.00,12.89) | -4.34(-5.05,-3.63) | 0.71(0.00,17.94) | 0.20(0.00,12.89) | -4.34(-5.05,-3.63) |
| Argentina | both | 0.47(0.35,0.60) | 0.26(0.19,0.34) | -2.16(-2.78,-1.54) | 0.47(0.35,0.60) | 0.26(0.19,0.34) | -2.16(-2.78,-1.54) |
| Armenia | both | 0.12(0.01,0.49) | 0.26(0.05,0.87) | 4.07(2.31,5.86) | 0.12(0.01,0.49) | 0.26(0.05,0.87) | 4.07(2.31,5.86) |
| Australia | both | 0.46(0.31,0.66) | 0.14(0.07,0.25) | -3.65(-3.94,-3.36) | 0.46(0.31,0.66) | 0.14(0.07,0.25) | -3.65(-3.94,-3.36) |
| Austria | both | 0.65(0.39,1.03) | 0.22(0.08,0.50) | -3.93(-4.18,-3.68) | 0.65(0.39,1.03) | 0.22(0.08,0.50) | -3.93(-4.18,-3.68) |
| Azerbaijan | both | 0.08(0.01,0.29) | 0.03(0.00,0.17) | -4.48(-6.15,-2.78) | 0.08(0.01,0.29) | 0.03(0.00,0.17) | -4.48(-6.15,-2.78) |
| Bahamas | both | 1.03(0.04,5.65) | 0.49(0.00,3.38) | -2.55(-2.86,-2.24) | 1.03(0.04,5.65) | 0.49(0.00,3.38) | -2.55(-2.86,-2.24) |
| Bahrain | both | 0.05(0.00,2.28) | 0.06(0.00,0.85) | 0.19(-0.79,1.17) | 0.05(0.00,2.28) | 0.06(0.00,0.85) | 0.19(-0.79,1.17) |
| Bangladesh | both | 0.38(0.32,0.44) | 0.30(0.26,0.35) | -0.17(-0.51,0.16) | 0.38(0.32,0.44) | 0.30(0.26,0.35) | -0.17(-0.51,0.16) |
| Barbados | both | 0.53(0.00,4.58) | 0.32(0.00,4.80) | -2.46(-3.01,-1.90) | 0.53(0.00,4.58) | 0.32(0.00,4.80) | -2.46(-3.01,-1.90) |
| Belarus | both | 1.32(0.98,1.74) | 0.51(0.28,0.87) | -3.46(-4.32,-2.58) | 1.32(0.98,1.74) | 0.51(0.28,0.87) | -3.46(-4.32,-2.58) |
| Belgium | both | 0.76(0.50,1.11) | 0.27(0.13,0.52) | -3.63(-4.00,-3.26) | 0.76(0.50,1.11) | 0.27(0.13,0.52) | -3.63(-4.00,-3.26) |
| Belize | both | 2.48(0.25,11.22) | 1.40(0.25,4.54) | -3.11(-3.82,-2.39) | 2.48(0.25,11.22) | 1.40(0.25,4.54) | -3.11(-3.82,-2.39) |
| Benin | both | 0.33(0.11,0.79) | 0.27(0.14,0.47) | -0.95(-1.27,-0.62) | 0.33(0.11,0.79) | 0.27(0.14,0.47) | -0.95(-1.27,-0.62) |
| Bermuda | both | 1.19(0.00,22.30) | 0.13(0.00,26.65) | -8.16(-8.78,-7.53) | 1.19(0.00,22.30) | 0.13(0.00,26.65) | -8.16(-8.78,-7.53) |
| Bhutan | both | 0.37(0.01,2.66) | 0.14(0.00,1.46) | -3.92(-4.25,-3.59) | 0.37(0.01,2.66) | 0.14(0.00,1.46) | -3.92(-4.25,-3.59) |
| Bolivia (Plurinational State of) | both | 0.18(0.05,0.47) | 0.09(0.03,0.22) | -2.30(-2.70,-1.91) | 0.18(0.05,0.47) | 0.09(0.03,0.22) | -2.30(-2.70,-1.91) |
| Bosnia and Herzegovina | both | 0.26(0.09,0.62) | 0.24(0.04,0.85) | -0.15(-0.59,0.29) | 0.26(0.09,0.62) | 0.24(0.04,0.85) | -0.15(-0.59,0.29) |
| Botswana | both | 0.15(0.00,1.26) | 0.11(0.00,0.57) | -1.35(-1.76,-0.93) | 0.15(0.00,1.26) | 0.11(0.00,0.57) | -1.35(-1.76,-0.93) |
| Brazil | both | 0.50(0.44,0.55) | 0.58(0.53,0.63) | 0.76(-0.16,1.69) | 0.50(0.44,0.55) | 0.58(0.53,0.63) | 0.76(-0.16,1.69) |
| Brunei Darussalam | both | 1.06(0.05,5.20) | 0.38(0.00,2.96) | -3.55(-4.27,-2.82) | 1.06(0.05,5.20) | 0.38(0.00,2.96) | -3.55(-4.27,-2.82) |
| Bulgaria | both | 0.26(0.11,0.52) | 0.28(0.09,0.69) | -0.17(-0.66,0.31) | 0.26(0.11,0.52) | 0.28(0.09,0.69) | -0.17(-0.66,0.31) |
| Burkina Faso | both | 0.37(0.18,0.68) | 0.41(0.28,0.58) | 0.78(0.48,1.08) | 0.37(0.18,0.68) | 0.41(0.28,0.58) | 0.78(0.48,1.08) |
| Burundi | both | 1.38(0.92,2.02) | 0.67(0.46,0.93) | -2.88(-3.19,-2.56) | 1.38(0.92,2.02) | 0.67(0.46,0.93) | -2.88(-3.19,-2.56) |
| Cabo Verde | both | 0.24(0.00,5.32) | 0.29(0.00,2.15) | 0.32(-0.28,0.92) | 0.24(0.00,5.32) | 0.29(0.00,2.15) | 0.32(-0.28,0.92) |
| Cambodia | both | 0.53(0.32,0.83) | 0.87(0.67,1.11) | 1.08(0.82,1.34) | 0.53(0.32,0.83) | 0.87(0.67,1.11) | 1.08(0.82,1.34) |
| Cameroon | both | 0.44(0.25,0.73) | 0.48(0.36,0.62) | 0.33(0.10,0.56) | 0.44(0.25,0.73) | 0.48(0.36,0.62) | 0.33(0.10,0.56) |
| Canada | both | 0.44(0.32,0.59) | 0.24(0.16,0.35) | -1.76(-2.08,-1.44) | 0.44(0.32,0.59) | 0.24(0.16,0.35) | -1.76(-2.08,-1.44) |
| Central African Republic | both | 0.46(0.14,1.18) | 0.38(0.16,0.77) | -0.59(-0.70,-0.47) | 0.46(0.14,1.18) | 0.38(0.16,0.77) | -0.59(-0.70,-0.47) |
| Chad | both | 0.17(0.04,0.49) | 0.29(0.17,0.48) | 2.06(1.63,2.49) | 0.17(0.04,0.49) | 0.29(0.17,0.48) | 2.06(1.63,2.49) |
| Chile | both | 0.24(0.13,0.41) | 0.35(0.22,0.52) | 1.40(0.46,2.36) | 0.24(0.13,0.41) | 0.35(0.22,0.52) | 1.40(0.46,2.36) |
| China | both | 0.79(0.76,0.81) | 1.08(1.05,1.11) | 0.85(0.17,1.53) | 0.79(0.76,0.81) | 1.08(1.05,1.11) | 0.85(0.17,1.53) |
| Colombia | both | 0.63(0.50,0.78) | 0.60(0.50,0.72) | -0.99(-1.68,-0.31) | 0.63(0.50,0.78) | 0.60(0.50,0.72) | -0.99(-1.68,-0.31) |
| Comoros | both | 0.48(0.01,3.89) | 0.44(0.02,2.11) | -1.35(-2.32,-0.37) | 0.48(0.01,3.89) | 0.44(0.02,2.11) | -1.35(-2.32,-0.37) |
| Congo | both | 0.38(0.09,1.14) | 0.28(0.10,0.61) | -1.28(-1.74,-0.80) | 0.38(0.09,1.14) | 0.28(0.10,0.61) | -1.28(-1.74,-0.80) |
| Cook Islands | both | 0.11(0.00,59.04) | 0.34(0.00,68.68) | 4.74(4.32,5.17) | 0.11(0.00,59.04) | 0.34(0.00,68.68) | 4.74(4.32,5.17) |
| Costa Rica | both | 0.76(0.36,1.45) | 0.67(0.36,1.17) | -1.25(-1.68,-0.81) | 0.76(0.36,1.45) | 0.67(0.36,1.17) | -1.25(-1.68,-0.81) |
| Croatia | both | 0.45(0.20,0.90) | 0.29(0.07,0.83) | -2.41(-3.25,-1.57) | 0.45(0.20,0.90) | 0.29(0.07,0.83) | -2.41(-3.25,-1.57) |
| Cuba | both | 6.54(5.84,7.31) | 1.06(0.75,1.46) | -7.18(-7.83,-6.52) | 6.54(5.84,7.31) | 1.06(0.75,1.46) | -7.18(-7.83,-6.52) |
| Cyprus | both | 0.30(0.01,1.86) | 0.12(0.00,1.40) | -3.74(-4.61,-2.85) | 0.30(0.01,1.86) | 0.12(0.00,1.40) | -3.74(-4.61,-2.85) |
| Czechia | both | 0.41(0.23,0.68) | 0.31(0.14,0.60) | -2.91(-4.19,-1.62) | 0.41(0.23,0.68) | 0.31(0.14,0.60) | -2.91(-4.19,-1.62) |
| Côte d'Ivoire | both | 0.26(0.13,0.47) | 0.31(0.22,0.44) | 0.43(0.20,0.66) | 0.26(0.13,0.47) | 0.31(0.22,0.44) | 0.43(0.20,0.66) |
| Democratic People's Republic of Korea | both | 0.72(0.55,0.93) | 1.17(0.97,1.41) | 1.42(1.20,1.63) | 0.72(0.55,0.93) | 1.17(0.97,1.41) | 1.42(1.20,1.63) |
| Democratic Republic of the Congo | both | 0.21(0.14,0.31) | 0.24(0.19,0.30) | 0.49(0.21,0.78) | 0.21(0.14,0.31) | 0.24(0.19,0.30) | 0.49(0.21,0.78) |
| Denmark | both | 0.98(0.59,1.56) | 0.25(0.08,0.64) | -4.86(-5.23,-4.49) | 0.98(0.59,1.56) | 0.25(0.08,0.64) | -4.86(-5.23,-4.49) |
| Djibouti | both | 0.31(0.00,3.60) | 0.37(0.05,1.35) | 0.42(0.12,0.72) | 0.31(0.00,3.60) | 0.37(0.05,1.35) | 0.42(0.12,0.72) |
| Dominica | both | 0.68(0.00,17.85) | 0.55(0.00,15.75) | -1.02(-1.26,-0.79) | 0.68(0.00,17.85) | 0.55(0.00,15.75) | -1.02(-1.26,-0.79) |
| Dominican Republic | both | 0.38(0.19,0.69) | 0.41(0.25,0.65) | 0.79(0.16,1.43) | 0.38(0.19,0.69) | 0.41(0.25,0.65) | 0.79(0.16,1.43) |
| Ecuador | both | 0.44(0.26,0.71) | 0.66(0.49,0.87) | 2.61(1.66,3.57) | 0.44(0.26,0.71) | 0.66(0.49,0.87) | 2.61(1.66,3.57) |
| Egypt | both | 0.17(0.12,0.24) | 0.19(0.15,0.24) | 1.30(0.68,1.93) | 0.17(0.12,0.24) | 0.19(0.15,0.24) | 1.30(0.68,1.93) |
| El Salvador | both | 0.39(0.16,0.80) | 0.38(0.18,0.71) | -0.37(-0.88,0.15) | 0.39(0.16,0.80) | 0.38(0.18,0.71) | -0.37(-0.88,0.15) |
| Equatorial Guinea | both | 0.36(0.00,3.76) | 0.28(0.03,1.17) | -0.80(-1.42,-0.16) | 0.36(0.00,3.76) | 0.28(0.03,1.17) | -0.80(-1.42,-0.16) |
| Eritrea | both | 0.78(0.37,1.49) | 0.72(0.44,1.12) | -0.86(-1.16,-0.56) | 0.78(0.37,1.49) | 0.72(0.44,1.12) | -0.86(-1.16,-0.56) |
| Estonia | both | 1.05(0.38,2.35) | 0.21(0.00,1.61) | -5.12(-5.98,-4.26) | 1.05(0.38,2.35) | 0.21(0.00,1.61) | -5.12(-5.98,-4.26) |
| Eswatini | both | 0.23(0.00,2.20) | 0.53(0.09,1.73) | 3.32(2.29,4.37) | 0.23(0.00,2.20) | 0.53(0.09,1.73) | 3.32(2.29,4.37) |
| Ethiopia | both | 0.58(0.47,0.70) | 0.26(0.21,0.31) | -3.05(-3.47,-2.63) | 0.58(0.47,0.70) | 0.26(0.21,0.31) | -3.05(-3.47,-2.63) |
| Fiji | both | 0.11(0.00,1.56) | 0.12(0.00,1.30) | 1.22(0.81,1.62) | 0.11(0.00,1.56) | 0.12(0.00,1.30) | 1.22(0.81,1.62) |
| Finland | both | 1.14(0.69,1.80) | 0.34(0.11,0.79) | -3.39(-3.69,-3.09) | 1.14(0.69,1.80) | 0.34(0.11,0.79) | -3.39(-3.69,-3.09) |
| France | both | 0.33(0.26,0.42) | 0.15(0.10,0.21) | -2.98(-3.36,-2.60) | 0.33(0.26,0.42) | 0.15(0.10,0.21) | -2.98(-3.36,-2.60) |
| Gabon | both | 0.46(0.04,2.00) | 0.36(0.06,1.18) | -0.98(-1.14,-0.82) | 0.46(0.04,2.00) | 0.36(0.06,1.18) | -0.98(-1.14,-0.82) |
| Gambia | both | 0.20(0.00,1.78) | 0.29(0.05,0.95) | 0.87(0.51,1.24) | 0.20(0.00,1.78) | 0.29(0.05,0.95) | 0.87(0.51,1.24) |
| Georgia | both | 0.46(0.22,0.86) | 0.25(0.05,0.82) | -1.60(-2.70,-0.48) | 0.46(0.22,0.86) | 0.25(0.05,0.82) | -1.60(-2.70,-0.48) |
| Germany | both | 0.64(0.54,0.74) | 0.23(0.17,0.30) | -3.92(-4.17,-3.67) | 0.64(0.54,0.74) | 0.23(0.17,0.30) | -3.92(-4.17,-3.67) |
| Ghana | both | 0.66(0.47,0.92) | 0.67(0.55,0.83) | 0.22(0.06,0.38) | 0.66(0.47,0.92) | 0.67(0.55,0.83) | 0.22(0.06,0.38) |
| Greece | both | 0.08(0.01,0.23) | 0.05(0.00,0.25) | -1.12(-1.36,-0.89) | 0.08(0.01,0.23) | 0.05(0.00,0.25) | -1.12(-1.36,-0.89) |
| Greenland | both | 0.48(0.00,22.81) | 0.13(0.00,22.59) | -4.39(-4.83,-3.94) | 0.48(0.00,22.81) | 0.13(0.00,22.59) | -4.39(-4.83,-3.94) |
| Grenada | both | 1.45(0.00,16.63) | 0.72(0.00,11.82) | -2.89(-3.41,-2.37) | 1.45(0.00,16.63) | 0.72(0.00,11.82) | -2.89(-3.41,-2.37) |
| Guam | both | 0.04(0.00,6.58) | 0.12(0.00,7.11) | 5.08(3.41,6.78) | 0.04(0.00,6.58) | 0.12(0.00,7.11) | 5.08(3.41,6.78) |
| Guatemala | both | 0.32(0.15,0.63) | 0.27(0.16,0.44) | -0.63(-0.97,-0.28) | 0.32(0.15,0.63) | 0.27(0.16,0.44) | -0.63(-0.97,-0.28) |
| Guinea | both | 0.19(0.05,0.51) | 0.23(0.11,0.41) | 0.55(0.46,0.65) | 0.19(0.05,0.51) | 0.23(0.11,0.41) | 0.55(0.46,0.65) |
| Guinea-Bissau | both | 0.77(0.14,2.56) | 0.64(0.21,1.53) | -0.53(-0.58,-0.48) | 0.77(0.14,2.56) | 0.64(0.21,1.53) | -0.53(-0.58,-0.48) |
| Guyana | both | 1.26(0.36,3.34) | 1.39(0.40,3.60) | -0.25(-0.94,0.43) | 1.26(0.36,3.34) | 1.39(0.40,3.60) | -0.25(-0.94,0.43) |
| Haiti | both | 1.75(1.27,2.37) | 1.20(0.93,1.53) | -1.04(-1.28,-0.80) | 1.75(1.27,2.37) | 1.20(0.93,1.53) | -1.04(-1.28,-0.80) |
| Honduras | both | 0.46(0.19,0.95) | 0.22(0.10,0.42) | -3.16(-3.64,-2.68) | 0.46(0.19,0.95) | 0.22(0.10,0.42) | -3.16(-3.64,-2.68) |
| Hungary | both | 1.64(1.25,2.11) | 0.67(0.39,1.07) | -4.04(-4.62,-3.47) | 1.64(1.25,2.11) | 0.67(0.39,1.07) | -4.04(-4.62,-3.47) |
| Iceland | both | 0.16(0.00,4.15) | 0.07(0.00,3.87) | -3.14(-3.61,-2.67) | 0.16(0.00,4.15) | 0.07(0.00,3.87) | -3.14(-3.61,-2.67) |
| India | both | 0.93(0.90,0.96) | 0.83(0.81,0.86) | -0.43(-0.80,-0.06) | 0.93(0.90,0.96) | 0.83(0.81,0.86) | -0.43(-0.80,-0.06) |
| Indonesia | both | 0.51(0.46,0.56) | 0.50(0.46,0.54) | -0.06(-0.25,0.13) | 0.51(0.46,0.56) | 0.50(0.46,0.54) | -0.06(-0.25,0.13) |
| Iran (Islamic Republic of) | both | 0.03(0.01,0.07) | 0.10(0.07,0.14) | 4.84(2.99,6.72) | 0.03(0.01,0.07) | 0.10(0.07,0.14) | 4.84(2.99,6.72) |
| Iraq | both | 0.11(0.05,0.23) | 0.07(0.04,0.12) | -1.23(-1.45,-1.01) | 0.11(0.05,0.23) | 0.07(0.04,0.12) | -1.23(-1.45,-1.01) |
| Ireland | both | 0.63(0.29,1.21) | 0.15(0.02,0.51) | -5.11(-5.62,-4.59) | 0.63(0.29,1.21) | 0.15(0.02,0.51) | -5.11(-5.62,-4.59) |
| Israel | both | 0.18(0.04,0.51) | 0.12(0.03,0.31) | -1.96(-2.64,-1.27) | 0.18(0.04,0.51) | 0.12(0.03,0.31) | -1.96(-2.64,-1.27) |
| Italy | both | 0.58(0.48,0.69) | 0.20(0.13,0.28) | -3.54(-3.76,-3.32) | 0.58(0.48,0.69) | 0.20(0.13,0.28) | -3.54(-3.76,-3.32) |
| Jamaica | both | 0.26(0.04,0.93) | 0.29(0.07,0.81) | -1.30(-2.31,-0.27) | 0.26(0.04,0.93) | 0.29(0.07,0.81) | -1.30(-2.31,-0.27) |
| Japan | both | 0.61(0.54,0.68) | 0.17(0.13,0.22) | -4.40(-4.70,-4.09) | 0.61(0.54,0.68) | 0.17(0.13,0.22) | -4.40(-4.70,-4.09) |
| Jordan | both | 0.05(0.00,0.47) | 0.07(0.02,0.19) | 2.65(1.88,3.43) | 0.05(0.00,0.47) | 0.07(0.02,0.19) | 2.65(1.88,3.43) |
| Kazakhstan | both | 0.34(0.21,0.51) | 0.16(0.08,0.29) | -1.69(-2.43,-0.95) | 0.34(0.21,0.51) | 0.16(0.08,0.29) | -1.69(-2.43,-0.95) |
| Kenya | both | 0.26(0.16,0.40) | 0.36(0.29,0.46) | 1.78(1.37,2.20) | 0.26(0.16,0.40) | 0.36(0.29,0.46) | 1.78(1.37,2.20) |
| Kiribati | both | 0.08(0.00,15.51) | 0.07(0.00,8.23) | -0.59(-0.82,-0.36) | 0.08(0.00,15.51) | 0.07(0.00,8.23) | -0.59(-0.82,-0.36) |
| Kuwait | both | 0.42(0.10,1.25) | 0.43(0.18,0.92) | 0.27(-2.01,2.61) | 0.42(0.10,1.25) | 0.43(0.18,0.92) | 0.27(-2.01,2.61) |
| Kyrgyzstan | both | 0.46(0.20,0.93) | 0.27(0.11,0.55) | -1.33(-2.26,-0.40) | 0.46(0.20,0.93) | 0.27(0.11,0.55) | -1.33(-2.26,-0.40) |
| Lao People's Democratic Republic | both | 0.71(0.35,1.31) | 0.70(0.44,1.06) | -0.26(-0.36,-0.16) | 0.71(0.35,1.31) | 0.70(0.44,1.06) | -0.26(-0.36,-0.16) |
| Latvia | both | 1.39(0.74,2.39) | 0.41(0.06,1.60) | -4.04(-4.53,-3.56) | 1.39(0.74,2.39) | 0.41(0.06,1.60) | -4.04(-4.53,-3.56) |
| Lebanon | both | 0.18(0.02,0.70) | 0.09(0.01,0.36) | -1.81(-2.37,-1.24) | 0.18(0.02,0.70) | 0.09(0.01,0.36) | -1.81(-2.37,-1.24) |
| Lesotho | both | 0.12(0.00,1.05) | 0.40(0.09,1.19) | 5.15(4.36,5.95) | 0.12(0.00,1.05) | 0.40(0.09,1.19) | 5.15(4.36,5.95) |
| Liberia | both | 0.23(0.03,0.87) | 0.25(0.08,0.58) | 0.88(0.49,1.26) | 0.23(0.03,0.87) | 0.25(0.08,0.58) | 0.88(0.49,1.26) |
| Libya | both | 0.02(0.00,0.34) | 0.04(0.00,0.21) | 4.69(3.95,5.44) | 0.02(0.00,0.34) | 0.04(0.00,0.21) | 4.69(3.95,5.44) |
| Lithuania | both | 1.40(0.85,2.19) | 0.52(0.15,1.40) | -2.80(-3.68,-1.90) | 1.40(0.85,2.19) | 0.52(0.15,1.40) | -2.80(-3.68,-1.90) |
| Luxembourg | both | 0.31(0.00,4.19) | 0.07(0.00,2.51) | -5.44(-5.75,-5.12) | 0.31(0.00,4.19) | 0.07(0.00,2.51) | -5.44(-5.75,-5.12) |
| Madagascar | both | 0.41(0.24,0.66) | 0.34(0.24,0.46) | -0.55(-0.75,-0.35) | 0.41(0.24,0.66) | 0.34(0.24,0.46) | -0.55(-0.75,-0.35) |
| Malawi | both | 0.42(0.23,0.71) | 0.50(0.35,0.69) | 0.51(0.21,0.80) | 0.42(0.23,0.71) | 0.50(0.35,0.69) | 0.51(0.21,0.80) |
| Malaysia | both | 0.73(0.55,0.96) | 0.74(0.61,0.90) | -0.61(-0.97,-0.24) | 0.73(0.55,0.96) | 0.74(0.61,0.90) | -0.61(-0.97,-0.24) |
| Maldives | both | 0.49(0.00,8.32) | 0.09(0.00,2.80) | -6.64(-7.43,-5.84) | 0.49(0.00,8.32) | 0.09(0.00,2.80) | -6.64(-7.43,-5.84) |
| Mali | both | 0.22(0.08,0.49) | 0.18(0.10,0.31) | -0.51(-0.69,-0.33) | 0.22(0.08,0.49) | 0.18(0.10,0.31) | -0.51(-0.69,-0.33) |
| Malta | both | 0.08(0.00,3.33) | 0.04(0.00,4.12) | -3.13(-3.46,-2.80) | 0.08(0.00,3.33) | 0.04(0.00,4.12) | -3.13(-3.46,-2.80) |
| Marshall Islands | both | 0.07(0.00,27.37) | 0.08(0.00,16.66) | 0.53(0.26,0.81) | 0.07(0.00,27.37) | 0.08(0.00,16.66) | 0.53(0.26,0.81) |
| Mauritania | both | 0.56(0.16,1.49) | 0.36(0.13,0.83) | -1.48(-1.67,-1.30) | 0.56(0.16,1.49) | 0.36(0.13,0.83) | -1.48(-1.67,-1.30) |
| Mauritius | both | 0.01(0.00,0.81) | 0.33(0.03,1.48) | 15.04(11.91,18.25) | 0.01(0.00,0.81) | 0.33(0.03,1.48) | 15.04(11.91,18.25) |
| Mexico | both | 0.67(0.58,0.76) | 0.46(0.41,0.53) | -1.07(-1.45,-0.70) | 0.67(0.58,0.76) | 0.46(0.41,0.53) | -1.07(-1.45,-0.70) |
| Micronesia (Federated States of) | both | 0.11(0.00,11.65) | 0.12(0.00,10.52) | 0.16(-0.02,0.33) | 0.11(0.00,11.65) | 0.12(0.00,10.52) | 0.16(-0.02,0.33) |
| Monaco | both | 0.20(0.00,61.61) | 0.14(0.00,45.43) | -1.30(-1.37,-1.23) | 0.20(0.00,61.61) | 0.14(0.00,45.43) | -1.30(-1.37,-1.23) |
| Mongolia | both | 0.01(0.00,0.67) | 0.07(0.00,0.47) | 5.99(5.32,6.66) | 0.01(0.00,0.67) | 0.07(0.00,0.47) | 5.99(5.32,6.66) |
| Montenegro | both | 0.47(0.02,2.39) | 0.29(0.00,2.59) | -1.62(-2.17,-1.07) | 0.47(0.02,2.39) | 0.29(0.00,2.59) | -1.62(-2.17,-1.07) |
| Morocco | both | 0.01(0.00,0.06) | 0.02(0.00,0.05) | 2.41(1.90,2.92) | 0.01(0.00,0.06) | 0.02(0.00,0.05) | 2.41(1.90,2.92) |
| Mozambique | both | 0.34(0.20,0.56) | 0.64(0.50,0.81) | 2.66(2.26,3.06) | 0.34(0.20,0.56) | 0.64(0.50,0.81) | 2.66(2.26,3.06) |
| Myanmar | both | 0.68(0.56,0.82) | 0.48(0.39,0.58) | -1.29(-1.50,-1.07) | 0.68(0.56,0.82) | 0.48(0.39,0.58) | -1.29(-1.50,-1.07) |
| Namibia | both | 0.16(0.00,1.23) | 0.31(0.07,0.91) | 1.72(1.01,2.45) | 0.16(0.00,1.23) | 0.31(0.07,0.91) | 1.72(1.01,2.45) |
| Nauru | both | 0.21(0.00,105.26) | 0.34(0.00,91.44) | 1.56(1.16,1.96) | 0.21(0.00,105.26) | 0.34(0.00,91.44) | 1.56(1.16,1.96) |
| Nepal | both | 0.42(0.29,0.61) | 0.31(0.22,0.42) | -1.01(-1.21,-0.80) | 0.42(0.29,0.61) | 0.31(0.22,0.42) | -1.01(-1.21,-0.80) |
| Netherlands | both | 1.36(1.08,1.71) | 0.46(0.29,0.68) | -3.96(-4.21,-3.70) | 1.36(1.08,1.71) | 0.46(0.29,0.68) | -3.96(-4.21,-3.70) |
| New Zealand | both | 0.64(0.29,1.23) | 0.20(0.05,0.57) | -3.42(-3.98,-2.85) | 0.64(0.29,1.23) | 0.20(0.05,0.57) | -3.42(-3.98,-2.85) |
| Nicaragua | both | 0.44(0.16,1.01) | 0.37(0.18,0.67) | -0.44(-0.70,-0.19) | 0.44(0.16,1.01) | 0.37(0.18,0.67) | -0.44(-0.70,-0.19) |
| Niger | both | 0.21(0.07,0.49) | 0.22(0.12,0.36) | 0.37(0.24,0.51) | 0.21(0.07,0.49) | 0.22(0.12,0.36) | 0.37(0.24,0.51) |
| Nigeria | both | 0.21(0.16,0.27) | 0.21(0.18,0.24) | 0.09(-0.09,0.26) | 0.21(0.16,0.27) | 0.21(0.18,0.24) | 0.09(-0.09,0.26) |
| Niue | both | 0.08(0.00,518.34) | 0.13(0.00,703.47) | 0.67(0.32,1.03) | 0.08(0.00,518.34) | 0.13(0.00,703.47) | 0.67(0.32,1.03) |
| North Macedonia | both | 0.13(0.00,0.72) | 0.09(0.00,0.82) | -1.81(-2.31,-1.31) | 0.13(0.00,0.72) | 0.09(0.00,0.82) | -1.81(-2.31,-1.31) |
| Northern Mariana Islands | both | 0.10(0.00,20.11) | 0.05(0.00,26.09) | -2.83(-3.73,-1.92) | 0.10(0.00,20.11) | 0.05(0.00,26.09) | -2.83(-3.73,-1.92) |
| Norway | both | 0.35(0.12,0.79) | 0.12(0.02,0.46) | -2.99(-3.54,-2.43) | 0.35(0.12,0.79) | 0.12(0.02,0.46) | -2.99(-3.54,-2.43) |
| Oman | both | 0.15(0.01,0.77) | 0.26(0.09,0.66) | 3.28(2.25,4.32) | 0.15(0.01,0.77) | 0.26(0.09,0.66) | 3.28(2.25,4.32) |
| Pakistan | both | 0.46(0.39,0.53) | 0.74(0.69,0.80) | 1.38(1.02,1.74) | 0.46(0.39,0.53) | 0.74(0.69,0.80) | 1.38(1.02,1.74) |
| Palau | both | 0.00(0.00,59.03) | 0.01(0.00,69.24) | 3.07(2.63,3.51) | 0.00(0.00,59.03) | 0.01(0.00,69.24) | 3.07(2.63,3.51) |
| Palestine | both | 0.02(0.00,0.82) | 0.02(0.00,0.25) | 0.04(-0.87,0.97) | 0.02(0.00,0.82) | 0.02(0.00,0.25) | 0.04(-0.87,0.97) |
| Panama | both | 0.87(0.40,1.71) | 0.58(0.27,1.09) | -1.63(-1.99,-1.28) | 0.87(0.40,1.71) | 0.58(0.27,1.09) | -1.63(-1.99,-1.28) |
| Papua New Guinea | both | 0.09(0.01,0.45) | 0.08(0.02,0.22) | -0.80(-0.98,-0.62) | 0.09(0.01,0.45) | 0.08(0.02,0.22) | -0.80(-0.98,-0.62) |
| Paraguay | both | 0.12(0.01,0.49) | 0.23(0.09,0.47) | 2.07(1.56,2.59) | 0.12(0.01,0.49) | 0.23(0.09,0.47) | 2.07(1.56,2.59) |
| Peru | both | 0.36(0.25,0.52) | 0.54(0.43,0.67) | 2.30(1.66,2.95) | 0.36(0.25,0.52) | 0.54(0.43,0.67) | 2.30(1.66,2.95) |
| Philippines | both | 0.37(0.30,0.45) | 0.40(0.35,0.46) | 0.59(0.36,0.82) | 0.37(0.30,0.45) | 0.40(0.35,0.46) | 0.59(0.36,0.82) |
| Poland | both | 0.97(0.81,1.15) | 0.69(0.55,0.87) | -2.34(-2.99,-1.69) | 0.97(0.81,1.15) | 0.69(0.55,0.87) | -2.34(-2.99,-1.69) |
| Portugal | both | 0.64(0.41,0.95) | 0.18(0.06,0.42) | -5.11(-5.47,-4.75) | 0.64(0.41,0.95) | 0.18(0.06,0.42) | -5.11(-5.47,-4.75) |
| Puerto Rico | both | 0.84(0.43,1.48) | 0.62(0.24,1.34) | -2.32(-3.11,-1.53) | 0.84(0.43,1.48) | 0.62(0.24,1.34) | -2.32(-3.11,-1.53) |
| Qatar | both | 0.30(0.00,3.35) | 0.27(0.05,1.13) | 0.89(0.19,1.59) | 0.30(0.00,3.35) | 0.27(0.05,1.13) | 0.89(0.19,1.59) |
| Republic of Korea | both | 1.37(1.21,1.54) | 0.16(0.10,0.24) | -9.02(-9.89,-8.13) | 1.37(1.21,1.54) | 0.16(0.10,0.24) | -9.02(-9.89,-8.13) |
| Republic of Moldova | both | 0.73(0.38,1.28) | 0.22(0.04,0.81) | -2.68(-3.30,-2.04) | 0.73(0.38,1.28) | 0.22(0.04,0.81) | -2.68(-3.30,-2.04) |
| Romania | both | 0.82(0.64,1.03) | 0.45(0.29,0.68) | -1.57(-1.79,-1.36) | 0.82(0.64,1.03) | 0.45(0.29,0.68) | -1.57(-1.79,-1.36) |
| Russian Federation | both | 0.37(0.32,0.42) | 0.20(0.16,0.25) | -1.50(-2.44,-0.56) | 0.37(0.32,0.42) | 0.20(0.16,0.25) | -1.50(-2.44,-0.56) |
| Rwanda | both | 2.71(2.13,3.41) | 1.04(0.79,1.35) | -4.26(-4.75,-3.76) | 2.71(2.13,3.41) | 1.04(0.79,1.35) | -4.26(-4.75,-3.76) |
| Saint Kitts and Nevis | both | 1.25(0.00,29.02) | 0.32(0.00,19.78) | -5.83(-6.66,-5.00) | 1.25(0.00,29.02) | 0.32(0.00,19.78) | -5.83(-6.66,-5.00) |
| Saint Lucia | both | 1.63(0.02,12.04) | 0.68(0.00,7.81) | -3.66(-4.09,-3.23) | 1.63(0.02,12.04) | 0.68(0.00,7.81) | -3.66(-4.09,-3.23) |
| Saint Vincent and the Grenadines | both | 0.98(0.00,13.50) | 0.53(0.00,10.40) | -3.00(-3.49,-2.52) | 0.98(0.00,13.50) | 0.53(0.00,10.40) | -3.00(-3.49,-2.52) |
| Samoa | both | 0.06(0.00,8.46) | 0.10(0.00,5.82) | 1.96(1.73,2.19) | 0.06(0.00,8.46) | 0.10(0.00,5.82) | 1.96(1.73,2.19) |
| San Marino | both | 0.65(0.00,44.18) | 0.39(0.00,44.68) | -1.28(-1.50,-1.05) | 0.65(0.00,44.18) | 0.39(0.00,44.68) | -1.28(-1.50,-1.05) |
| Sao Tome and Principe | both | 0.16(0.00,14.02) | 0.27(0.00,5.19) | 1.60(1.08,2.13) | 0.16(0.00,14.02) | 0.27(0.00,5.19) | 1.60(1.08,2.13) |
| Saudi Arabia | both | 0.00(0.00,0.08) | 0.03(0.01,0.07) | 8.34(7.38,9.31) | 0.00(0.00,0.08) | 0.03(0.01,0.07) | 8.34(7.38,9.31) |
| Senegal | both | 0.21(0.07,0.51) | 0.20(0.10,0.35) | 0.01(-0.22,0.23) | 0.21(0.07,0.51) | 0.20(0.10,0.35) | 0.01(-0.22,0.23) |
| Serbia | both | 0.97(0.67,1.35) | 0.36(0.18,0.66) | -3.20(-3.39,-3.00) | 0.97(0.67,1.35) | 0.36(0.18,0.66) | -3.20(-3.39,-3.00) |
| Seychelles | both | 0.32(0.00,16.80) | 0.34(0.00,11.96) | 0.74(0.04,1.45) | 0.32(0.00,16.80) | 0.34(0.00,11.96) | 0.74(0.04,1.45) |
| Sierra Leone | both | 0.29(0.08,0.74) | 0.29(0.14,0.54) | 0.30(0.12,0.48) | 0.29(0.08,0.74) | 0.29(0.14,0.54) | 0.30(0.12,0.48) |
| Singapore | both | 0.41(0.15,0.91) | 0.06(0.00,0.44) | -6.88(-7.15,-6.60) | 0.41(0.15,0.91) | 0.06(0.00,0.44) | -6.88(-7.15,-6.60) |
| Slovakia | both | 1.38(0.92,2.00) | 0.56(0.26,1.10) | -3.14(-3.52,-2.76) | 1.38(0.92,2.00) | 0.56(0.26,1.10) | -3.14(-3.52,-2.76) |
| Slovenia | both | 0.77(0.28,1.74) | 0.26(0.02,1.31) | -4.60(-5.33,-3.87) | 0.77(0.28,1.74) | 0.26(0.02,1.31) | -4.60(-5.33,-3.87) |
| Solomon Islands | both | 0.07(0.00,4.42) | 0.09(0.00,1.75) | 1.14(0.87,1.41) | 0.07(0.00,4.42) | 0.09(0.00,1.75) | 1.14(0.87,1.41) |
| Somalia | both | 0.51(0.28,0.85) | 0.66(0.49,0.87) | 1.33(1.11,1.56) | 0.51(0.28,0.85) | 0.66(0.49,0.87) | 1.33(1.11,1.56) |
| South Africa | both | 0.55(0.44,0.69) | 0.41(0.33,0.49) | -1.64(-2.33,-0.95) | 0.55(0.44,0.69) | 0.41(0.33,0.49) | -1.64(-2.33,-0.95) |
| South Sudan | both | 0.31(0.12,0.69) | 0.44(0.25,0.74) | 1.06(0.66,1.46) | 0.31(0.12,0.69) | 0.44(0.25,0.74) | 1.06(0.66,1.46) |
| Spain | both | 0.33(0.24,0.44) | 0.11(0.06,0.19) | -4.47(-4.97,-3.97) | 0.33(0.24,0.44) | 0.11(0.06,0.19) | -4.47(-4.97,-3.97) |
| Sri Lanka | both | 0.63(0.46,0.84) | 0.52(0.37,0.70) | -1.39(-1.96,-0.82) | 0.63(0.46,0.84) | 0.52(0.37,0.70) | -1.39(-1.96,-0.82) |
| Sudan | both | 0.01(0.00,0.08) | 0.02(0.01,0.06) | 3.14(2.58,3.70) | 0.01(0.00,0.08) | 0.02(0.01,0.06) | 3.14(2.58,3.70) |
| Suriname | both | 2.42(0.62,6.88) | 1.13(0.18,3.73) | -2.87(-3.28,-2.47) | 2.42(0.62,6.88) | 1.13(0.18,3.73) | -2.87(-3.28,-2.47) |
| Sweden | both | 0.56(0.32,0.91) | 0.09(0.02,0.28) | -5.53(-5.94,-5.13) | 0.56(0.32,0.91) | 0.09(0.02,0.28) | -5.53(-5.94,-5.13) |
| Switzerland | both | 0.93(0.58,1.41) | 0.17(0.05,0.45) | -5.55(-5.91,-5.19) | 0.93(0.58,1.41) | 0.17(0.05,0.45) | -5.55(-5.91,-5.19) |
| Syrian Arab Republic | both | 0.11(0.03,0.28) | 0.06(0.01,0.24) | -1.44(-1.80,-1.07) | 0.11(0.03,0.28) | 0.06(0.01,0.24) | -1.44(-1.80,-1.07) |
| Taiwan (Province of China) | both | 0.01(0.00,0.06) | 0.47(0.32,0.66) | 16.39(13.41,19.46) | 0.01(0.00,0.06) | 0.47(0.32,0.66) | 16.39(13.41,19.46) |
| Tajikistan | both | 0.06(0.00,0.36) | 0.03(0.00,0.15) | -3.39(-4.57,-2.21) | 0.06(0.00,0.36) | 0.03(0.00,0.15) | -3.39(-4.57,-2.21) |
| Thailand | both | 0.53(0.45,0.63) | 0.75(0.64,0.87) | -0.82(-1.75,0.11) | 0.53(0.45,0.63) | 0.75(0.64,0.87) | -0.82(-1.75,0.11) |
| Timor-Leste | both | 0.24(0.00,1.89) | 0.31(0.03,1.48) | 0.76(0.14,1.39) | 0.24(0.00,1.89) | 0.31(0.03,1.48) | 0.76(0.14,1.39) |
| Togo | both | 0.43(0.15,1.03) | 0.48(0.27,0.79) | 0.42(0.19,0.64) | 0.43(0.15,1.03) | 0.48(0.27,0.79) | 0.42(0.19,0.64) |
| Tokelau | both | 0.05(0.00,773.66) | 0.09(0.00,821.67) | 0.80(0.43,1.17) | 0.05(0.00,773.66) | 0.09(0.00,821.67) | 0.80(0.43,1.17) |
| Tonga | both | 0.04(0.00,15.54) | 0.06(0.00,11.49) | 0.63(0.25,1.01) | 0.04(0.00,15.54) | 0.06(0.00,11.49) | 0.63(0.25,1.01) |
| Trinidad and Tobago | both | 0.84(0.24,2.16) | 0.64(0.14,1.92) | -1.10(-1.46,-0.74) | 0.84(0.24,2.16) | 0.64(0.14,1.92) | -1.10(-1.46,-0.74) |
| Tunisia | both | 0.34(0.17,0.62) | 0.36(0.20,0.59) | 0.03(-0.09,0.15) | 0.34(0.17,0.62) | 0.36(0.20,0.59) | 0.03(-0.09,0.15) |
| Turkey | both | 0.19(0.14,0.26) | 0.23(0.18,0.29) | 1.64(0.41,2.87) | 0.19(0.14,0.26) | 0.23(0.18,0.29) | 1.64(0.41,2.87) |
| Turkmenistan | both | 0.21(0.04,0.65) | 0.11(0.02,0.37) | -1.64(-2.35,-0.91) | 0.21(0.04,0.65) | 0.11(0.02,0.37) | -1.64(-2.35,-0.91) |
| Tuvalu | both | 0.12(0.00,108.58) | 0.14(0.00,84.64) | 0.56(0.35,0.76) | 0.12(0.00,108.58) | 0.14(0.00,84.64) | 0.56(0.35,0.76) |
| Uganda | both | 0.60(0.42,0.84) | 1.16(1.00,1.34) | 1.28(0.67,1.89) | 0.60(0.42,0.84) | 1.16(1.00,1.34) | 1.28(0.67,1.89) |
| Ukraine | both | 0.37(0.28,0.46) | 0.20(0.13,0.29) | -1.58(-2.34,-0.81) | 0.37(0.28,0.46) | 0.20(0.13,0.29) | -1.58(-2.34,-0.81) |
| United Arab Emirates | both | 0.90(0.36,1.95) | 0.98(0.60,1.54) | 0.75(-0.11,1.63) | 0.90(0.36,1.95) | 0.98(0.60,1.54) | 0.75(-0.11,1.63) |
| United Kingdom | both | 0.35(0.27,0.44) | 0.15(0.10,0.21) | -3.71(-4.27,-3.14) | 0.35(0.27,0.44) | 0.15(0.10,0.21) | -3.71(-4.27,-3.14) |
| United Republic of Tanzania | both | 0.49(0.36,0.67) | 0.45(0.36,0.55) | -0.52(-0.70,-0.34) | 0.49(0.36,0.67) | 0.45(0.36,0.55) | -0.52(-0.70,-0.34) |
| United States of America | both | 0.48(0.44,0.52) | 0.25(0.22,0.28) | -2.05(-2.53,-1.57) | 0.48(0.44,0.52) | 0.25(0.22,0.28) | -2.05(-2.53,-1.57) |
| United States Virgin Islands | both | 0.97(0.00,12.10) | 1.13(0.00,20.68) | 0.34(-0.18,0.86) | 0.97(0.00,12.10) | 1.13(0.00,20.68) | 0.34(-0.18,0.86) |
| Uruguay | both | 0.28(0.06,0.80) | 0.27(0.06,0.78) | -0.34(-0.73,0.06) | 0.28(0.06,0.80) | 0.27(0.06,0.78) | -0.34(-0.73,0.06) |
| Uzbekistan | both | 0.21(0.12,0.34) | 0.41(0.31,0.53) | 3.63(2.38,4.90) | 0.21(0.12,0.34) | 0.41(0.31,0.53) | 3.63(2.38,4.90) |
| Vanuatu | both | 0.08(0.00,8.06) | 0.13(0.00,3.78) | 1.07(0.94,1.20) | 0.08(0.00,8.06) | 0.13(0.00,3.78) | 1.07(0.94,1.20) |
| Venezuela (Bolivarian Republic of) | both | 0.52(0.38,0.71) | 0.40(0.28,0.56) | -1.11(-1.49,-0.74) | 0.52(0.38,0.71) | 0.40(0.28,0.56) | -1.11(-1.49,-0.74) |
| Viet Nam | both | 0.52(0.43,0.61) | 0.79(0.70,0.89) | 1.91(1.60,2.23) | 0.52(0.43,0.61) | 0.79(0.70,0.89) | 1.91(1.60,2.23) |
| Yemen | both | 0.02(0.00,0.13) | 0.03(0.01,0.08) | 2.23(1.70,2.77) | 0.02(0.00,0.13) | 0.03(0.01,0.08) | 2.23(1.70,2.77) |
| Zambia | both | 1.02(0.68,1.50) | 1.23(0.99,1.51) | 0.20(-0.07,0.46) | 1.02(0.68,1.50) | 1.23(0.99,1.51) | 0.20(-0.07,0.46) |
| Zimbabwe | both | 0.06(0.01,0.23) | 0.50(0.34,0.72) | 9.16(6.72,11.65) | 0.06(0.01,0.23) | 0.50(0.34,0.72) | 9.16(6.72,11.65) |
| Afghanistan | female | 0.00(0.00,0.46) | 0.00(0.00,0.11) | 3.49(2.94,4.04) | 0.00(0.00,0.46) | 0.00(0.00,0.11) | 3.49(2.94,4.04) |
| Albania | female | 0.06(0.00,0.79) | 0.06(0.00,1.10) | 0.46(-0.58,1.51) | 0.06(0.00,0.79) | 0.06(0.00,1.10) | 0.46(-0.58,1.51) |
| Algeria | female | 0.00(0.00,0.11) | 0.00(0.00,0.05) | 3.14(2.49,3.79) | 0.00(0.00,0.11) | 0.00(0.00,0.05) | 3.14(2.49,3.79) |
| American Samoa | female | 0.01(0.00,47.79) | 0.02(0.00,48.97) | 4.38(2.17,6.64) | 0.01(0.00,47.79) | 0.02(0.00,48.97) | 4.38(2.17,6.64) |
| Andorra | female | 0.31(0.00,43.30) | 0.16(0.00,40.53) | -2.05(-2.24,-1.86) | 0.31(0.00,43.30) | 0.16(0.00,40.53) | -2.05(-2.24,-1.86) |
| Angola | female | 0.02(0.00,0.28) | 0.03(0.00,0.12) | 1.51(0.72,2.31) | 0.02(0.00,0.28) | 0.03(0.00,0.12) | 1.51(0.72,2.31) |
| Antigua and Barbuda | female | 0.23(0.00,31.85) | 0.07(0.00,25.72) | -4.49(-5.00,-3.99) | 0.23(0.00,31.85) | 0.07(0.00,25.72) | -4.49(-5.00,-3.99) |
| Argentina | female | 0.28(0.17,0.45) | 0.13(0.06,0.23) | -2.91(-3.23,-2.59) | 0.28(0.17,0.45) | 0.13(0.06,0.23) | -2.91(-3.23,-2.59) |
| Armenia | female | 0.05(0.00,0.68) | 0.06(0.00,1.10) | 2.70(1.55,3.85) | 0.05(0.00,0.68) | 0.06(0.00,1.10) | 2.70(1.55,3.85) |
| Australia | female | 0.15(0.05,0.35) | 0.04(0.00,0.18) | -3.86(-4.15,-3.57) | 0.15(0.05,0.35) | 0.04(0.00,0.18) | -3.86(-4.15,-3.57) |
| Austria | female | 0.40(0.14,0.92) | 0.11(0.01,0.55) | -4.10(-4.47,-3.74) | 0.40(0.14,0.92) | 0.11(0.01,0.55) | -4.10(-4.47,-3.74) |
| Azerbaijan | female | 0.00(0.00,0.30) | 0.00(0.00,0.25) | -1.79(-2.64,-0.93) | 0.00(0.00,0.30) | 0.00(0.00,0.25) | -1.79(-2.64,-0.93) |
| Bahamas | female | 0.27(0.00,8.04) | 0.12(0.00,5.13) | -3.19(-3.65,-2.73) | 0.27(0.00,8.04) | 0.12(0.00,5.13) | -3.19(-3.65,-2.73) |
| Bahrain | female | 0.01(0.00,4.67) | 0.00(0.00,1.76) | -1.06(-2.30,0.20) | 0.01(0.00,4.67) | 0.00(0.00,1.76) | -1.06(-2.30,0.20) |
| Bangladesh | female | 0.03(0.01,0.06) | 0.01(0.00,0.03) | -1.60(-2.29,-0.90) | 0.03(0.01,0.06) | 0.01(0.00,0.03) | -1.60(-2.29,-0.90) |
| Barbados | female | 0.11(0.00,7.48) | 0.09(0.00,8.74) | -1.28(-1.83,-0.72) | 0.11(0.00,7.48) | 0.09(0.00,8.74) | -1.28(-1.83,-0.72) |
| Belarus | female | 0.35(0.14,0.75) | 0.17(0.03,0.62) | -3.00(-4.07,-1.92) | 0.35(0.14,0.75) | 0.17(0.03,0.62) | -3.00(-4.07,-1.92) |
| Belgium | female | 0.40(0.16,0.85) | 0.12(0.02,0.46) | -4.19(-4.64,-3.74) | 0.40(0.16,0.85) | 0.12(0.02,0.46) | -4.19(-4.64,-3.74) |
| Belize | female | 0.66(0.00,16.33) | 0.20(0.00,4.69) | -4.50(-4.94,-4.05) | 0.66(0.00,16.33) | 0.20(0.00,4.69) | -4.50(-4.94,-4.05) |
| Benin | female | 0.06(0.00,0.63) | 0.06(0.00,0.28) | -0.23(-0.52,0.06) | 0.06(0.00,0.63) | 0.06(0.00,0.28) | -0.23(-0.52,0.06) |
| Bermuda | female | 0.23(0.00,41.63) | 0.03(0.00,53.55) | -7.16(-7.69,-6.62) | 0.23(0.00,41.63) | 0.03(0.00,53.55) | -7.16(-7.69,-6.62) |
| Bhutan | female | 0.14(0.00,4.62) | 0.04(0.00,2.49) | -5.22(-5.65,-4.79) | 0.14(0.00,4.62) | 0.04(0.00,2.49) | -5.22(-5.65,-4.79) |
| Bolivia (Plurinational State of) | female | 0.10(0.00,0.53) | 0.04(0.00,0.23) | -3.17(-3.41,-2.94) | 0.10(0.00,0.53) | 0.04(0.00,0.23) | -3.17(-3.41,-2.94) |
| Bosnia and Herzegovina | female | 0.09(0.00,0.59) | 0.07(0.00,1.07) | -1.20(-1.54,-0.86) | 0.09(0.00,0.59) | 0.07(0.00,1.07) | -1.20(-1.54,-0.86) |
| Botswana | female | 0.02(0.00,1.93) | 0.03(0.00,0.79) | 1.81(0.69,2.94) | 0.02(0.00,1.93) | 0.03(0.00,0.79) | 1.81(0.69,2.94) |
| Brazil | female | 0.15(0.11,0.20) | 0.20(0.16,0.25) | 0.51(-0.46,1.49) | 0.15(0.11,0.20) | 0.20(0.16,0.25) | 0.51(-0.46,1.49) |
| Brunei Darussalam | female | 0.32(0.00,8.03) | 0.10(0.00,5.21) | -3.18(-3.40,-2.95) | 0.32(0.00,8.03) | 0.10(0.00,5.21) | -3.18(-3.40,-2.95) |
| Bulgaria | female | 0.07(0.00,0.40) | 0.08(0.00,0.66) | -0.74(-1.49,0.01) | 0.07(0.00,0.40) | 0.08(0.00,0.66) | -0.74(-1.49,0.01) |
| Burkina Faso | female | 0.09(0.01,0.44) | 0.07(0.02,0.22) | -0.12(-0.54,0.29) | 0.09(0.01,0.44) | 0.07(0.02,0.22) | -0.12(-0.54,0.29) |
| Burundi | female | 0.52(0.18,1.23) | 0.29(0.12,0.60) | -2.12(-2.38,-1.87) | 0.52(0.18,1.23) | 0.29(0.12,0.60) | -2.12(-2.38,-1.87) |
| Cabo Verde | female | 0.08(0.00,8.42) | 0.04(0.00,3.38) | -2.30(-2.66,-1.93) | 0.08(0.00,8.42) | 0.04(0.00,3.38) | -2.30(-2.66,-1.93) |
| Cambodia | female | 0.20(0.05,0.53) | 0.34(0.17,0.59) | 0.95(0.60,1.31) | 0.20(0.05,0.53) | 0.34(0.17,0.59) | 0.95(0.60,1.31) |
| Cameroon | female | 0.11(0.01,0.44) | 0.12(0.05,0.25) | 0.44(0.31,0.57) | 0.11(0.01,0.44) | 0.12(0.05,0.25) | 0.44(0.31,0.57) |
| Canada | female | 0.20(0.10,0.37) | 0.12(0.05,0.26) | -1.52(-1.83,-1.20) | 0.20(0.10,0.37) | 0.12(0.05,0.26) | -1.52(-1.83,-1.20) |
| Central African Republic | female | 0.02(0.00,1.02) | 0.02(0.00,0.45) | -0.21(-0.33,-0.10) | 0.02(0.00,1.02) | 0.02(0.00,0.45) | -0.21(-0.33,-0.10) |
| Chad | female | 0.04(0.00,0.49) | 0.05(0.00,0.25) | 1.49(1.27,1.71) | 0.04(0.00,0.49) | 0.05(0.00,0.25) | 1.49(1.27,1.71) |
| Chile | female | 0.08(0.01,0.29) | 0.12(0.03,0.31) | 0.85(-0.08,1.79) | 0.08(0.01,0.29) | 0.12(0.03,0.31) | 0.85(-0.08,1.79) |
| China | female | 0.58(0.55,0.61) | 0.60(0.57,0.63) | -0.21(-0.88,0.46) | 0.58(0.55,0.61) | 0.60(0.57,0.63) | -0.21(-0.88,0.46) |
| Colombia | female | 0.09(0.03,0.19) | 0.09(0.04,0.18) | -0.04(-0.53,0.46) | 0.09(0.03,0.19) | 0.09(0.04,0.18) | -0.04(-0.53,0.46) |
| Comoros | female | 0.16(0.00,6.19) | 0.18(0.00,3.06) | -0.53(-1.59,0.54) | 0.16(0.00,6.19) | 0.18(0.00,3.06) | -0.53(-1.59,0.54) |
| Congo | female | 0.02(0.00,1.12) | 0.03(0.00,0.40) | 1.16(0.92,1.41) | 0.02(0.00,1.12) | 0.03(0.00,0.40) | 1.16(0.92,1.41) |
| Cook Islands | female | 0.04(0.00,119.54) | 0.12(0.00,126.50) | 4.55(4.00,5.10) | 0.04(0.00,119.54) | 0.12(0.00,126.50) | 4.55(4.00,5.10) |
| Costa Rica | female | 0.10(0.00,0.87) | 0.10(0.00,0.64) | -0.52(-0.79,-0.24) | 0.10(0.00,0.87) | 0.10(0.00,0.64) | -0.52(-0.79,-0.24) |
| Croatia | female | 0.15(0.01,0.74) | 0.10(0.00,0.97) | -2.44(-3.35,-1.53) | 0.15(0.01,0.74) | 0.10(0.00,0.97) | -2.44(-3.35,-1.53) |
| Cuba | female | 1.55(1.09,2.14) | 0.29(0.09,0.71) | -6.71(-7.38,-6.04) | 1.55(1.09,2.14) | 0.29(0.09,0.71) | -6.71(-7.38,-6.04) |
| Cyprus | female | 0.09(0.00,2.92) | 0.02(0.00,2.56) | -4.03(-5.22,-2.82) | 0.09(0.00,2.92) | 0.02(0.00,2.56) | -4.03(-5.22,-2.82) |
| Czechia | female | 0.14(0.03,0.47) | 0.13(0.01,0.55) | -2.26(-3.39,-1.12) | 0.14(0.03,0.47) | 0.13(0.01,0.55) | -2.26(-3.39,-1.12) |
| Côte d'Ivoire | female | 0.05(0.00,0.32) | 0.08(0.02,0.20) | 2.00(1.77,2.23) | 0.05(0.00,0.32) | 0.08(0.02,0.20) | 2.00(1.77,2.23) |
| Democratic People's Republic of Korea | female | 0.36(0.20,0.60) | 0.53(0.34,0.78) | 1.13(0.91,1.36) | 0.36(0.20,0.60) | 0.53(0.34,0.78) | 1.13(0.91,1.36) |
| Democratic Republic of the Congo | female | 0.02(0.00,0.10) | 0.02(0.01,0.06) | 0.80(0.25,1.35) | 0.02(0.00,0.10) | 0.02(0.01,0.06) | 0.80(0.25,1.35) |
| Denmark | female | 0.80(0.33,1.65) | 0.19(0.02,0.82) | -4.92(-5.25,-4.60) | 0.80(0.33,1.65) | 0.19(0.02,0.82) | -4.92(-5.25,-4.60) |
| Djibouti | female | 0.06(0.00,6.85) | 0.06(0.00,1.71) | 0.03(-0.38,0.45) | 0.06(0.00,6.85) | 0.06(0.00,1.71) | 0.03(-0.38,0.45) |
| Dominica | female | 0.16(0.00,35.52) | 0.13(0.00,30.71) | -0.92(-1.18,-0.66) | 0.16(0.00,35.52) | 0.13(0.00,30.71) | -0.92(-1.18,-0.66) |
| Dominican Republic | female | 0.08(0.00,0.45) | 0.06(0.00,0.28) | -1.04(-1.49,-0.60) | 0.08(0.00,0.45) | 0.06(0.00,0.28) | -1.04(-1.49,-0.60) |
| Ecuador | female | 0.09(0.01,0.38) | 0.17(0.06,0.37) | 3.07(2.02,4.13) | 0.09(0.01,0.38) | 0.17(0.06,0.37) | 3.07(2.02,4.13) |
| Egypt | female | 0.03(0.01,0.09) | 0.02(0.00,0.04) | -1.41(-1.88,-0.93) | 0.03(0.01,0.09) | 0.02(0.00,0.04) | -1.41(-1.88,-0.93) |
| El Salvador | female | 0.12(0.01,0.65) | 0.12(0.01,0.52) | -0.26(-0.70,0.18) | 0.12(0.01,0.65) | 0.12(0.01,0.52) | -0.26(-0.70,0.18) |
| Equatorial Guinea | female | 0.01(0.00,5.52) | 0.03(0.00,1.53) | 1.57(1.20,1.94) | 0.01(0.00,5.52) | 0.03(0.00,1.53) | 1.57(1.20,1.94) |
| Eritrea | female | 0.10(0.00,0.93) | 0.12(0.01,0.53) | 0.41(0.17,0.65) | 0.10(0.00,0.93) | 0.12(0.01,0.53) | 0.41(0.17,0.65) |
| Estonia | female | 0.29(0.00,2.04) | 0.08(0.00,2.74) | -3.38(-4.27,-2.47) | 0.29(0.00,2.04) | 0.08(0.00,2.74) | -3.38(-4.27,-2.47) |
| Eswatini | female | 0.03(0.00,3.37) | 0.05(0.00,1.71) | 1.80(1.08,2.53) | 0.03(0.00,3.37) | 0.05(0.00,1.71) | 1.80(1.08,2.53) |
| Ethiopia | female | 0.19(0.11,0.31) | 0.10(0.06,0.15) | -2.97(-3.37,-2.57) | 0.19(0.11,0.31) | 0.10(0.06,0.15) | -2.97(-3.37,-2.57) |
| Fiji | female | 0.05(0.00,2.85) | 0.07(0.00,2.30) | 2.20(1.71,2.68) | 0.05(0.00,2.85) | 0.07(0.00,2.30) | 2.20(1.71,2.68) |
| Finland | female | 0.67(0.23,1.56) | 0.21(0.02,0.91) | -3.27(-3.60,-2.94) | 0.67(0.23,1.56) | 0.21(0.02,0.91) | -3.27(-3.60,-2.94) |
| France | female | 0.16(0.09,0.26) | 0.07(0.03,0.15) | -3.01(-3.52,-2.50) | 0.16(0.09,0.26) | 0.07(0.03,0.15) | -3.01(-3.52,-2.50) |
| Gabon | female | 0.03(0.00,2.77) | 0.02(0.00,1.18) | -1.34(-1.64,-1.05) | 0.03(0.00,2.77) | 0.02(0.00,1.18) | -1.34(-1.64,-1.05) |
| Gambia | female | 0.03(0.00,2.92) | 0.05(0.00,1.03) | 1.16(0.83,1.50) | 0.03(0.00,2.92) | 0.05(0.00,1.03) | 1.16(0.83,1.50) |
| Georgia | female | 0.13(0.01,0.61) | 0.05(0.00,0.98) | -3.15(-3.70,-2.61) | 0.13(0.01,0.61) | 0.05(0.00,0.98) | -3.15(-3.70,-2.61) |
| Germany | female | 0.38(0.29,0.51) | 0.14(0.07,0.23) | -3.92(-4.17,-3.67) | 0.38(0.29,0.51) | 0.14(0.07,0.23) | -3.92(-4.17,-3.67) |
| Ghana | female | 0.39(0.20,0.72) | 0.22(0.12,0.36) | -2.14(-2.56,-1.72) | 0.39(0.20,0.72) | 0.22(0.12,0.36) | -2.14(-2.56,-1.72) |
| Greece | female | 0.03(0.00,0.27) | 0.02(0.00,0.35) | -1.94(-2.24,-1.64) | 0.03(0.00,0.27) | 0.02(0.00,0.35) | -1.94(-2.24,-1.64) |
| Greenland | female | 0.12(0.00,46.98) | 0.03(0.00,44.58) | -4.76(-5.40,-4.11) | 0.12(0.00,46.98) | 0.03(0.00,44.58) | -4.76(-5.40,-4.11) |
| Grenada | female | 0.41(0.00,28.94) | 0.19(0.00,21.84) | -3.40(-3.79,-3.01) | 0.41(0.00,28.94) | 0.19(0.00,21.84) | -3.40(-3.79,-3.01) |
| Guam | female | 0.02(0.00,13.92) | 0.01(0.00,14.50) | -1.79(-3.80,0.28) | 0.02(0.00,13.92) | 0.01(0.00,14.50) | -1.79(-3.80,0.28) |
| Guatemala | female | 0.09(0.00,0.46) | 0.06(0.01,0.22) | -1.40(-1.79,-1.01) | 0.09(0.00,0.46) | 0.06(0.01,0.22) | -1.40(-1.79,-1.01) |
| Guinea | female | 0.05(0.00,0.49) | 0.05(0.00,0.26) | 0.19(0.12,0.25) | 0.05(0.00,0.49) | 0.05(0.00,0.26) | 0.19(0.12,0.25) |
| Guinea-Bissau | female | 0.22(0.00,2.85) | 0.14(0.00,1.27) | -1.21(-1.53,-0.88) | 0.22(0.00,2.85) | 0.14(0.00,1.27) | -1.21(-1.53,-0.88) |
| Guyana | female | 0.48(0.01,3.62) | 0.23(0.00,3.18) | -3.27(-3.77,-2.77) | 0.48(0.01,3.62) | 0.23(0.00,3.18) | -3.27(-3.77,-2.77) |
| Haiti | female | 0.80(0.39,1.48) | 0.58(0.33,0.94) | -0.85(-1.02,-0.69) | 0.80(0.39,1.48) | 0.58(0.33,0.94) | -0.85(-1.02,-0.69) |
| Honduras | female | 0.14(0.01,0.78) | 0.10(0.01,0.36) | -1.65(-2.21,-1.08) | 0.14(0.01,0.78) | 0.10(0.01,0.36) | -1.65(-2.21,-1.08) |
| Hungary | female | 0.43(0.18,0.87) | 0.31(0.09,0.82) | -2.47(-3.06,-1.87) | 0.43(0.18,0.87) | 0.31(0.09,0.82) | -2.47(-3.06,-1.87) |
| Iceland | female | 0.03(0.00,7.92) | 0.01(0.00,7.63) | -3.61(-3.99,-3.24) | 0.03(0.00,7.92) | 0.01(0.00,7.63) | -3.61(-3.99,-3.24) |
| India | female | 0.18(0.16,0.20) | 0.11(0.10,0.13) | -2.51(-2.96,-2.07) | 0.18(0.16,0.20) | 0.11(0.10,0.13) | -2.51(-2.96,-2.07) |
| Indonesia | female | 0.23(0.18,0.28) | 0.16(0.13,0.20) | -1.28(-1.57,-0.98) | 0.23(0.18,0.28) | 0.16(0.13,0.20) | -1.28(-1.57,-0.98) |
| Iran (Islamic Republic of) | female | 0.01(0.00,0.06) | 0.01(0.00,0.05) | 2.01(0.84,3.19) | 0.01(0.00,0.06) | 0.01(0.00,0.05) | 2.01(0.84,3.19) |
| Iraq | female | 0.01(0.00,0.17) | 0.01(0.00,0.06) | -1.92(-2.53,-1.31) | 0.01(0.00,0.17) | 0.01(0.00,0.06) | -1.92(-2.53,-1.31) |
| Ireland | female | 0.30(0.04,1.09) | 0.07(0.00,0.67) | -4.68(-5.12,-4.23) | 0.30(0.04,1.09) | 0.07(0.00,0.67) | -4.68(-5.12,-4.23) |
| Israel | female | 0.04(0.00,0.49) | 0.01(0.00,0.26) | -3.17(-3.60,-2.74) | 0.04(0.00,0.49) | 0.01(0.00,0.26) | -3.17(-3.60,-2.74) |
| Italy | female | 0.27(0.18,0.39) | 0.05(0.01,0.14) | -5.41(-5.88,-4.95) | 0.27(0.18,0.39) | 0.05(0.01,0.14) | -5.41(-5.88,-4.95) |
| Jamaica | female | 0.08(0.00,1.14) | 0.07(0.00,0.84) | -1.26(-1.97,-0.53) | 0.08(0.00,1.14) | 0.07(0.00,0.84) | -1.26(-1.97,-0.53) |
| Japan | female | 0.43(0.35,0.52) | 0.14(0.08,0.21) | -4.58(-5.06,-4.10) | 0.43(0.35,0.52) | 0.14(0.08,0.21) | -4.58(-5.06,-4.10) |
| Jordan | female | 0.00(0.00,0.83) | 0.00(0.00,0.17) | 2.56(1.81,3.32) | 0.00(0.00,0.83) | 0.00(0.00,0.17) | 2.56(1.81,3.32) |
| Kazakhstan | female | 0.10(0.02,0.28) | 0.04(0.00,0.20) | -2.53(-3.29,-1.76) | 0.10(0.02,0.28) | 0.04(0.00,0.20) | -2.53(-3.29,-1.76) |
| Kenya | female | 0.10(0.03,0.29) | 0.10(0.05,0.18) | 0.65(0.25,1.05) | 0.10(0.03,0.29) | 0.10(0.05,0.18) | 0.65(0.25,1.05) |
| Kiribati | female | 0.00(0.00,29.52) | 0.00(0.00,15.53) | -4.88(-5.41,-4.34) | 0.00(0.00,29.52) | 0.00(0.00,15.53) | -4.88(-5.41,-4.34) |
| Kuwait | female | 0.09(0.00,1.36) | 0.04(0.00,0.73) | -3.56(-7.64,0.69) | 0.09(0.00,1.36) | 0.04(0.00,0.73) | -3.56(-7.64,0.69) |
| Kyrgyzstan | female | 0.15(0.01,0.75) | 0.07(0.00,0.43) | -2.05(-3.14,-0.96) | 0.15(0.01,0.75) | 0.07(0.00,0.43) | -2.05(-3.14,-0.96) |
| Lao People's Democratic Republic | female | 0.29(0.04,1.07) | 0.30(0.09,0.73) | -0.08(-0.39,0.23) | 0.29(0.04,1.07) | 0.30(0.09,0.73) | -0.08(-0.39,0.23) |
| Latvia | female | 0.32(0.02,1.45) | 0.14(0.00,2.18) | -2.65(-3.38,-1.92) | 0.32(0.02,1.45) | 0.14(0.00,2.18) | -2.65(-3.38,-1.92) |
| Lebanon | female | 0.01(0.00,0.77) | 0.01(0.00,0.45) | -3.57(-3.88,-3.27) | 0.01(0.00,0.77) | 0.01(0.00,0.45) | -3.57(-3.88,-3.27) |
| Lesotho | female | 0.02(0.00,1.49) | 0.05(0.00,1.17) | 4.53(3.55,5.52) | 0.02(0.00,1.49) | 0.05(0.00,1.17) | 4.53(3.55,5.52) |
| Liberia | female | 0.05(0.00,1.07) | 0.06(0.00,0.50) | 0.55(0.19,0.92) | 0.05(0.00,1.07) | 0.06(0.00,0.50) | 0.55(0.19,0.92) |
| Libya | female | 0.00(0.00,0.75) | 0.00(0.00,0.28) | 5.86(4.82,6.91) | 0.00(0.00,0.75) | 0.00(0.00,0.28) | 5.86(4.82,6.91) |
| Lithuania | female | 0.31(0.04,1.13) | 0.11(0.00,1.48) | -2.16(-3.31,-0.99) | 0.31(0.04,1.13) | 0.11(0.00,1.48) | -2.16(-3.31,-0.99) |
| Luxembourg | female | 0.08(0.00,7.66) | 0.02(0.00,5.01) | -5.85(-6.38,-5.31) | 0.08(0.00,7.66) | 0.02(0.00,5.01) | -5.85(-6.38,-5.31) |
| Madagascar | female | 0.14(0.03,0.44) | 0.12(0.05,0.26) | -0.39(-0.58,-0.20) | 0.14(0.03,0.44) | 0.12(0.05,0.26) | -0.39(-0.58,-0.20) |
| Malawi | female | 0.13(0.02,0.49) | 0.14(0.05,0.32) | 0.07(-0.04,0.19) | 0.13(0.02,0.49) | 0.14(0.05,0.32) | 0.07(-0.04,0.19) |
| Malaysia | female | 0.26(0.12,0.49) | 0.19(0.10,0.33) | -1.04(-1.30,-0.79) | 0.26(0.12,0.49) | 0.19(0.10,0.33) | -1.04(-1.30,-0.79) |
| Maldives | female | 0.30(0.00,15.95) | 0.03(0.00,5.80) | -9.02(-9.88,-8.15) | 0.30(0.00,15.95) | 0.03(0.00,5.80) | -9.02(-9.88,-8.15) |
| Mali | female | 0.08(0.00,0.44) | 0.05(0.01,0.19) | -1.93(-2.22,-1.64) | 0.08(0.00,0.44) | 0.05(0.01,0.19) | -1.93(-2.22,-1.64) |
| Malta | female | 0.02(0.00,6.52) | 0.00(0.00,8.32) | -3.34(-3.89,-2.79) | 0.02(0.00,6.52) | 0.00(0.00,8.32) | -3.34(-3.89,-2.79) |
| Marshall Islands | female | 0.03(0.00,56.86) | 0.03(0.00,34.57) | 0.02(-0.54,0.58) | 0.03(0.00,56.86) | 0.03(0.00,34.57) | 0.02(-0.54,0.58) |
| Mauritania | female | 0.12(0.00,1.40) | 0.08(0.00,0.69) | -1.08(-1.48,-0.67) | 0.12(0.00,1.40) | 0.08(0.00,0.69) | -1.08(-1.48,-0.67) |
| Mauritius | female | 0.00(0.00,1.63) | 0.06(0.00,1.93) | 11.09(8.65,13.59) | 0.00(0.00,1.63) | 0.06(0.00,1.93) | 11.09(8.65,13.59) |
| Mexico | female | 0.09(0.05,0.15) | 0.08(0.05,0.12) | 0.03(-0.50,0.57) | 0.09(0.05,0.15) | 0.08(0.05,0.12) | 0.03(-0.50,0.57) |
| Micronesia (Federated States of) | female | 0.09(0.00,24.26) | 0.06(0.00,21.38) | -1.61(-1.87,-1.35) | 0.09(0.00,24.26) | 0.06(0.00,21.38) | -1.61(-1.87,-1.35) |
| Monaco | female | 0.09(0.00,125.76) | 0.05(0.00,92.98) | -1.87(-2.19,-1.54) | 0.09(0.00,125.76) | 0.05(0.00,92.98) | -1.87(-2.19,-1.54) |
| Mongolia | female | 0.00(0.00,1.31) | 0.00(0.00,0.73) | 2.55(2.13,2.97) | 0.00(0.00,1.31) | 0.00(0.00,0.73) | 2.55(2.13,2.97) |
| Montenegro | female | 0.16(0.00,3.46) | 0.11(0.00,4.53) | -1.12(-1.85,-0.38) | 0.16(0.00,3.46) | 0.11(0.00,4.53) | -1.12(-1.85,-0.38) |
| Morocco | female | 0.00(0.00,0.09) | 0.00(0.00,0.05) | 4.64(3.78,5.50) | 0.00(0.00,0.09) | 0.00(0.00,0.05) | 4.64(3.78,5.50) |
| Mozambique | female | 0.09(0.01,0.32) | 0.09(0.03,0.22) | 0.31(0.19,0.43) | 0.09(0.01,0.32) | 0.09(0.03,0.22) | 0.31(0.19,0.43) |
| Myanmar | female | 0.40(0.27,0.56) | 0.16(0.10,0.26) | -3.27(-3.84,-2.69) | 0.40(0.27,0.56) | 0.16(0.10,0.26) | -3.27(-3.84,-2.69) |
| Namibia | female | 0.03(0.00,1.90) | 0.04(0.00,0.86) | 1.15(0.94,1.37) | 0.03(0.00,1.90) | 0.04(0.00,0.86) | 1.15(0.94,1.37) |
| Nauru | female | 0.09(0.00,211.84) | 0.16(0.00,181.07) | 1.63(0.99,2.27) | 0.09(0.00,211.84) | 0.16(0.00,181.07) | 1.63(0.99,2.27) |
| Nepal | female | 0.08(0.02,0.26) | 0.05(0.01,0.13) | -2.05(-2.17,-1.93) | 0.08(0.02,0.26) | 0.05(0.01,0.13) | -2.05(-2.17,-1.93) |
| Netherlands | female | 1.05(0.71,1.52) | 0.28(0.12,0.59) | -4.52(-4.82,-4.22) | 1.05(0.71,1.52) | 0.28(0.12,0.59) | -4.52(-4.82,-4.22) |
| New Zealand | female | 0.26(0.03,1.01) | 0.06(0.00,0.63) | -3.02(-4.46,-1.55) | 0.26(0.03,1.01) | 0.06(0.00,0.63) | -3.02(-4.46,-1.55) |
| Nicaragua | female | 0.10(0.00,0.84) | 0.06(0.00,0.38) | -1.72(-1.93,-1.51) | 0.10(0.00,0.84) | 0.06(0.00,0.38) | -1.72(-1.93,-1.51) |
| Niger | female | 0.05(0.00,0.45) | 0.04(0.00,0.20) | -0.51(-0.71,-0.31) | 0.05(0.00,0.45) | 0.04(0.00,0.20) | -0.51(-0.71,-0.31) |
| Nigeria | female | 0.04(0.02,0.09) | 0.04(0.03,0.07) | 0.02(-0.27,0.32) | 0.04(0.02,0.09) | 0.04(0.03,0.07) | 0.02(-0.27,0.32) |
| Niue | female | 0.03(0.00,1042.24) | 0.07(0.00,1386.03) | 0.20(-0.58,0.98) | 0.03(0.00,1042.24) | 0.07(0.00,1386.03) | 0.20(-0.58,0.98) |
| North Macedonia | female | 0.05(0.00,1.07) | 0.03(0.00,1.46) | -2.04(-2.43,-1.64) | 0.05(0.00,1.07) | 0.03(0.00,1.46) | -2.04(-2.43,-1.64) |
| Northern Mariana Islands | female | 0.17(0.00,37.33) | 0.01(0.00,55.01) | -11.39(-13.17,-9.57) | 0.17(0.00,37.33) | 0.01(0.00,55.01) | -11.39(-13.17,-9.57) |
| Norway | female | 0.13(0.00,0.76) | 0.07(0.00,0.65) | -3.14(-4.03,-2.24) | 0.13(0.00,0.76) | 0.07(0.00,0.65) | -3.14(-4.03,-2.24) |
| Oman | female | 0.00(0.00,1.60) | 0.01(0.00,0.63) | 8.24(6.21,10.30) | 0.00(0.00,1.60) | 0.01(0.00,0.63) | 8.24(6.21,10.30) |
| Pakistan | female | 0.08(0.05,0.14) | 0.12(0.10,0.16) | 0.68(0.26,1.11) | 0.08(0.05,0.14) | 0.12(0.10,0.16) | 0.68(0.26,1.11) |
| Palau | female | 0.01(0.00,121.50) | 0.02(0.00,179.81) | 3.50(3.09,3.90) | 0.01(0.00,121.50) | 0.02(0.00,179.81) | 3.50(3.09,3.90) |
| Palestine | female | 0.00(0.00,1.57) | 0.00(0.00,0.45) | 1.20(-0.25,2.66) | 0.00(0.00,1.57) | 0.00(0.00,0.45) | 1.20(-0.25,2.66) |
| Panama | female | 0.07(0.00,1.05) | 0.06(0.00,0.59) | -0.84(-1.17,-0.51) | 0.07(0.00,1.05) | 0.06(0.00,0.59) | -0.84(-1.17,-0.51) |
| Papua New Guinea | female | 0.07(0.00,0.73) | 0.05(0.00,0.29) | -0.97(-1.14,-0.81) | 0.07(0.00,0.73) | 0.05(0.00,0.29) | -0.97(-1.14,-0.81) |
| Paraguay | female | 0.08(0.00,0.72) | 0.07(0.00,0.39) | -0.25(-0.56,0.06) | 0.08(0.00,0.72) | 0.07(0.00,0.39) | -0.25(-0.56,0.06) |
| Peru | female | 0.09(0.03,0.25) | 0.12(0.05,0.23) | 1.28(0.79,1.77) | 0.09(0.03,0.25) | 0.12(0.05,0.23) | 1.28(0.79,1.77) |
| Philippines | female | 0.16(0.09,0.25) | 0.11(0.07,0.16) | -1.26(-1.52,-0.99) | 0.16(0.09,0.25) | 0.11(0.07,0.16) | -1.26(-1.52,-0.99) |
| Poland | female | 0.21(0.12,0.36) | 0.28(0.15,0.47) | -0.30(-1.02,0.42) | 0.21(0.12,0.36) | 0.28(0.15,0.47) | -0.30(-1.02,0.42) |
| Portugal | female | 0.25(0.08,0.59) | 0.06(0.00,0.41) | -4.92(-5.44,-4.39) | 0.25(0.08,0.59) | 0.06(0.00,0.41) | -4.92(-5.44,-4.39) |
| Puerto Rico | female | 0.09(0.00,0.69) | 0.09(0.00,0.96) | -1.52(-2.51,-0.53) | 0.09(0.00,0.69) | 0.09(0.00,0.96) | -1.52(-2.51,-0.53) |
| Qatar | female | 0.02(0.00,7.11) | 0.01(0.00,1.61) | -1.11(-3.70,1.55) | 0.02(0.00,7.11) | 0.01(0.00,1.61) | -1.11(-3.70,1.55) |
| Republic of Korea | female | 0.45(0.33,0.61) | 0.08(0.03,0.19) | -7.47(-8.18,-6.74) | 0.45(0.33,0.61) | 0.08(0.03,0.19) | -7.47(-8.18,-6.74) |
| Republic of Moldova | female | 0.11(0.00,0.70) | 0.06(0.00,1.06) | -1.48(-2.33,-0.62) | 0.11(0.00,0.70) | 0.06(0.00,1.06) | -1.48(-2.33,-0.62) |
| Romania | female | 0.21(0.10,0.41) | 0.09(0.01,0.32) | -2.45(-2.73,-2.17) | 0.21(0.10,0.41) | 0.09(0.01,0.32) | -2.45(-2.73,-2.17) |
| Russian Federation | female | 0.05(0.03,0.09) | 0.05(0.03,0.10) | 0.74(-0.57,2.07) | 0.05(0.03,0.09) | 0.05(0.03,0.10) | 0.74(-0.57,2.07) |
| Rwanda | female | 1.26(0.74,2.05) | 0.48(0.26,0.81) | -3.42(-3.77,-3.07) | 1.26(0.74,2.05) | 0.48(0.26,0.81) | -3.42(-3.77,-3.07) |
| Saint Kitts and Nevis | female | 0.44(0.00,53.46) | 0.05(0.00,38.49) | -7.98(-8.66,-7.29) | 0.44(0.00,53.46) | 0.05(0.00,38.49) | -7.98(-8.66,-7.29) |
| Saint Lucia | female | 0.50(0.00,18.98) | 0.20(0.00,13.99) | -3.91(-4.36,-3.46) | 0.50(0.00,18.98) | 0.20(0.00,13.99) | -3.91(-4.36,-3.46) |
| Saint Vincent and the Grenadines | female | 0.15(0.00,24.30) | 0.10(0.00,19.22) | -3.11(-3.89,-2.32) | 0.15(0.00,24.30) | 0.10(0.00,19.22) | -3.11(-3.89,-2.32) |
| Samoa | female | 0.04(0.00,16.97) | 0.06(0.00,11.55) | 1.75(1.38,2.11) | 0.04(0.00,16.97) | 0.06(0.00,11.55) | 1.75(1.38,2.11) |
| San Marino | female | 0.04(0.00,85.84) | 0.01(0.00,90.81) | -6.40(-7.33,-5.46) | 0.04(0.00,85.84) | 0.01(0.00,90.81) | -6.40(-7.33,-5.46) |
| Sao Tome and Principe | female | 0.03(0.00,25.40) | 0.04(0.00,9.59) | 0.10(-0.88,1.08) | 0.03(0.00,25.40) | 0.04(0.00,9.59) | 0.10(-0.88,1.08) |
| Saudi Arabia | female | 0.00(0.00,0.19) | 0.00(0.00,0.07) | 5.77(4.82,6.72) | 0.00(0.00,0.19) | 0.00(0.00,0.07) | 5.77(4.82,6.72) |
| Senegal | female | 0.06(0.00,0.46) | 0.05(0.00,0.22) | -0.96(-1.37,-0.55) | 0.06(0.00,0.46) | 0.05(0.00,0.22) | -0.96(-1.37,-0.55) |
| Serbia | female | 0.31(0.11,0.71) | 0.11(0.01,0.50) | -3.85(-4.13,-3.56) | 0.31(0.11,0.71) | 0.11(0.01,0.50) | -3.85(-4.13,-3.56) |
| Seychelles | female | 0.16(0.00,35.90) | 0.24(0.00,23.65) | 1.10(0.59,1.61) | 0.16(0.00,35.90) | 0.24(0.00,23.65) | 1.10(0.59,1.61) |
| Sierra Leone | female | 0.06(0.00,0.67) | 0.07(0.00,0.38) | 0.85(0.55,1.16) | 0.06(0.00,0.67) | 0.07(0.00,0.38) | 0.85(0.55,1.16) |
| Singapore | female | 0.07(0.00,0.76) | 0.02(0.00,0.79) | -6.03(-6.65,-5.41) | 0.07(0.00,0.76) | 0.02(0.00,0.79) | -6.03(-6.65,-5.41) |
| Slovakia | female | 0.41(0.11,1.07) | 0.22(0.02,0.98) | -1.92(-2.32,-1.52) | 0.41(0.11,1.07) | 0.22(0.02,0.98) | -1.92(-2.32,-1.52) |
| Slovenia | female | 0.33(0.01,1.72) | 0.11(0.00,1.99) | -4.25(-5.10,-3.40) | 0.33(0.01,1.72) | 0.11(0.00,1.99) | -4.25(-5.10,-3.40) |
| Solomon Islands | female | 0.02(0.00,8.96) | 0.02(0.00,3.13) | -0.15(-0.65,0.34) | 0.02(0.00,8.96) | 0.02(0.00,3.13) | -0.15(-0.65,0.34) |
| Somalia | female | 0.10(0.01,0.48) | 0.08(0.02,0.25) | -0.19(-0.70,0.33) | 0.10(0.01,0.48) | 0.08(0.02,0.25) | -0.19(-0.70,0.33) |
| South Africa | female | 0.09(0.03,0.19) | 0.04(0.01,0.09) | -3.00(-4.27,-1.72) | 0.09(0.03,0.19) | 0.04(0.01,0.09) | -3.00(-4.27,-1.72) |
| South Sudan | female | 0.08(0.00,0.63) | 0.10(0.01,0.43) | 1.00(0.45,1.54) | 0.08(0.00,0.63) | 0.10(0.01,0.43) | 1.00(0.45,1.54) |
| Spain | female | 0.12(0.06,0.24) | 0.04(0.00,0.13) | -4.59(-5.06,-4.12) | 0.12(0.06,0.24) | 0.04(0.00,0.13) | -4.59(-5.06,-4.12) |
| Sri Lanka | female | 0.18(0.07,0.38) | 0.11(0.03,0.27) | -2.01(-2.33,-1.68) | 0.18(0.07,0.38) | 0.11(0.03,0.27) | -2.01(-2.33,-1.68) |
| Sudan | female | 0.00(0.00,0.13) | 0.00(0.00,0.05) | 1.96(1.17,2.75) | 0.00(0.00,0.13) | 0.00(0.00,0.05) | 1.96(1.17,2.75) |
| Suriname | female | 0.70(0.00,7.47) | 0.40(0.00,4.35) | -2.28(-2.95,-1.61) | 0.70(0.00,7.47) | 0.40(0.00,4.35) | -2.28(-2.95,-1.61) |
| Sweden | female | 0.42(0.15,0.93) | 0.04(0.00,0.35) | -7.13(-7.61,-6.65) | 0.42(0.15,0.93) | 0.04(0.00,0.35) | -7.13(-7.61,-6.65) |
| Switzerland | female | 0.54(0.20,1.19) | 0.10(0.00,0.56) | -5.82(-6.12,-5.51) | 0.54(0.20,1.19) | 0.10(0.00,0.56) | -5.82(-6.12,-5.51) |
| Syrian Arab Republic | female | 0.00(0.00,0.24) | 0.00(0.00,0.24) | -1.86(-2.36,-1.36) | 0.00(0.00,0.24) | 0.00(0.00,0.24) | -1.86(-2.36,-1.36) |
| Taiwan (Province of China) | female | 0.00(0.00,0.10) | 0.30(0.15,0.58) | 17.72(14.69,20.84) | 0.00(0.00,0.10) | 0.30(0.15,0.58) | 17.72(14.69,20.84) |
| Tajikistan | female | 0.06(0.00,0.60) | 0.02(0.00,0.24) | -3.31(-4.46,-2.15) | 0.06(0.00,0.60) | 0.02(0.00,0.24) | -3.31(-4.46,-2.15) |
| Thailand | female | 0.23(0.15,0.33) | 0.24(0.16,0.36) | -1.05(-2.12,0.03) | 0.23(0.15,0.33) | 0.24(0.16,0.36) | -1.05(-2.12,0.03) |
| Timor-Leste | female | 0.09(0.00,3.20) | 0.12(0.00,2.12) | 0.61(-0.24,1.46) | 0.09(0.00,3.20) | 0.12(0.00,2.12) | 0.61(-0.24,1.46) |
| Togo | female | 0.16(0.01,0.97) | 0.14(0.02,0.47) | -0.08(-0.22,0.06) | 0.16(0.01,0.97) | 0.14(0.02,0.47) | -0.08(-0.22,0.06) |
| Tokelau | female | 0.04(0.00,1516.38) | 0.07(0.00,1645.67) | -0.09(-0.76,0.59) | 0.04(0.00,1516.38) | 0.07(0.00,1645.67) | -0.09(-0.76,0.59) |
| Tonga | female | 0.04(0.00,28.54) | 0.03(0.00,21.20) | -2.21(-2.77,-1.66) | 0.04(0.00,28.54) | 0.03(0.00,21.20) | -2.21(-2.77,-1.66) |
| Trinidad and Tobago | female | 0.12(0.00,1.90) | 0.07(0.00,1.94) | -2.75(-3.26,-2.25) | 0.12(0.00,1.90) | 0.07(0.00,1.94) | -2.75(-3.26,-2.25) |
| Tunisia | female | 0.21(0.05,0.62) | 0.17(0.04,0.46) | -0.56(-0.75,-0.37) | 0.21(0.05,0.62) | 0.17(0.04,0.46) | -0.56(-0.75,-0.37) |
| Turkey | female | 0.02(0.00,0.07) | 0.02(0.00,0.06) | 2.34(0.88,3.82) | 0.02(0.00,0.07) | 0.02(0.00,0.06) | 2.34(0.88,3.82) |
| Turkmenistan | female | 0.04(0.00,0.69) | 0.02(0.00,0.44) | -1.02(-1.67,-0.36) | 0.04(0.00,0.69) | 0.02(0.00,0.44) | -1.02(-1.67,-0.36) |
| Tuvalu | female | 0.10(0.00,203.64) | 0.06(0.00,185.18) | -1.68(-1.95,-1.40) | 0.10(0.00,203.64) | 0.06(0.00,185.18) | -1.68(-1.95,-1.40) |
| Uganda | female | 0.17(0.05,0.42) | 0.44(0.31,0.61) | 2.19(1.56,2.82) | 0.17(0.05,0.42) | 0.44(0.31,0.61) | 2.19(1.56,2.82) |
| Ukraine | female | 0.06(0.02,0.14) | 0.04(0.01,0.15) | -0.80(-1.63,0.04) | 0.06(0.02,0.14) | 0.04(0.01,0.15) | -0.80(-1.63,0.04) |
| United Arab Emirates | female | 0.57(0.05,2.47) | 0.45(0.11,1.29) | -1.12(-1.97,-0.26) | 0.57(0.05,2.47) | 0.45(0.11,1.29) | -1.12(-1.97,-0.26) |
| United Kingdom | female | 0.10(0.05,0.19) | 0.05(0.01,0.11) | -3.94(-4.71,-3.16) | 0.10(0.05,0.19) | 0.05(0.01,0.11) | -3.94(-4.71,-3.16) |
| United Republic of Tanzania | female | 0.11(0.04,0.27) | 0.11(0.06,0.19) | -0.05(-0.13,0.03) | 0.11(0.04,0.27) | 0.11(0.06,0.19) | -0.05(-0.13,0.03) |
| United States of America | female | 0.12(0.09,0.16) | 0.08(0.05,0.10) | -1.27(-1.94,-0.59) | 0.12(0.09,0.16) | 0.08(0.05,0.10) | -1.27(-1.94,-0.59) |
| United States Virgin Islands | female | 0.18(0.00,19.80) | 0.11(0.00,37.00) | -1.25(-1.83,-0.66) | 0.18(0.00,19.80) | 0.11(0.00,37.00) | -1.25(-1.83,-0.66) |
| Uruguay | female | 0.31(0.03,1.21) | 0.21(0.01,1.04) | -1.80(-2.09,-1.51) | 0.31(0.03,1.21) | 0.21(0.01,1.04) | -1.80(-2.09,-1.51) |
| Uzbekistan | female | 0.07(0.01,0.21) | 0.06(0.02,0.16) | 0.73(0.13,1.34) | 0.07(0.01,0.21) | 0.06(0.02,0.16) | 0.73(0.13,1.34) |
| Vanuatu | female | 0.03(0.00,16.03) | 0.04(0.00,6.98) | -0.23(-0.49,0.04) | 0.03(0.00,16.03) | 0.04(0.00,6.98) | -0.23(-0.49,0.04) |
| Venezuela (Bolivarian Republic of) | female | 0.16(0.06,0.35) | 0.18(0.08,0.36) | -0.02(-0.41,0.37) | 0.16(0.06,0.35) | 0.18(0.08,0.36) | -0.02(-0.41,0.37) |
| Viet Nam | female | 0.26(0.18,0.36) | 0.32(0.25,0.42) | 1.19(0.77,1.62) | 0.26(0.18,0.36) | 0.32(0.25,0.42) | 1.19(0.77,1.62) |
| Yemen | female | 0.00(0.00,0.20) | 0.00(0.00,0.06) | 3.80(3.09,4.50) | 0.00(0.00,0.20) | 0.00(0.00,0.06) | 3.80(3.09,4.50) |
| Zambia | female | 0.52(0.22,1.13) | 0.52(0.32,0.81) | -0.01(-0.42,0.40) | 0.52(0.22,1.13) | 0.52(0.32,0.81) | -0.01(-0.42,0.40) |
| Zimbabwe | female | 0.03(0.00,0.31) | 0.23(0.09,0.47) | 10.10(7.70,12.56) | 0.03(0.00,0.31) | 0.23(0.09,0.47) | 10.10(7.70,12.56) |
| Afghanistan | male | 0.02(0.00,0.76) | 0.05(0.01,0.18) | 3.57(3.22,3.93) | 0.02(0.00,0.76) | 0.05(0.01,0.18) | 3.57(3.22,3.93) |
| Albania | male | 0.25(0.02,1.02) | 0.24(0.01,1.29) | -0.60(-1.59,0.39) | 0.25(0.02,1.02) | 0.24(0.01,1.29) | -0.60(-1.59,0.39) |
| Algeria | male | 0.02(0.00,0.14) | 0.04(0.01,0.12) | 2.26(1.85,2.68) | 0.02(0.00,0.14) | 0.04(0.01,0.12) | 2.26(1.85,2.68) |
| American Samoa | male | 0.02(0.00,45.45) | 0.14(0.00,49.84) | 9.75(7.19,12.38) | 0.02(0.00,45.45) | 0.14(0.00,49.84) | 9.75(7.19,12.38) |
| Andorra | male | 1.06(0.00,40.98) | 0.69(0.00,37.95) | -1.24(-1.78,-0.69) | 1.06(0.00,40.98) | 0.69(0.00,37.95) | -1.24(-1.78,-0.69) |
| Angola | male | 0.46(0.21,0.90) | 0.54(0.36,0.78) | 1.29(0.87,1.71) | 0.46(0.21,0.90) | 0.54(0.36,0.78) | 1.29(0.87,1.71) |
| Antigua and Barbuda | male | 1.23(0.00,37.53) | 0.33(0.00,25.13) | -4.43(-5.21,-3.65) | 1.23(0.00,37.53) | 0.33(0.00,25.13) | -4.43(-5.21,-3.65) |
| Argentina | male | 0.65(0.46,0.89) | 0.38(0.26,0.54) | -1.90(-2.63,-1.16) | 0.65(0.46,0.89) | 0.38(0.26,0.54) | -1.90(-2.63,-1.16) |
| Armenia | male | 0.19(0.01,0.92) | 0.45(0.07,1.61) | 4.29(2.37,6.26) | 0.19(0.01,0.92) | 0.45(0.07,1.61) | 4.29(2.37,6.26) |
| Australia | male | 0.77(0.51,1.14) | 0.24(0.12,0.45) | -3.61(-3.92,-3.30) | 0.77(0.51,1.14) | 0.24(0.12,0.45) | -3.61(-3.92,-3.30) |
| Austria | male | 0.89(0.47,1.54) | 0.32(0.09,0.83) | -3.87(-4.11,-3.64) | 0.89(0.47,1.54) | 0.32(0.09,0.83) | -3.87(-4.11,-3.64) |
| Azerbaijan | male | 0.17(0.03,0.59) | 0.06(0.00,0.32) | -4.70(-6.34,-3.03) | 0.17(0.03,0.59) | 0.06(0.00,0.32) | -4.70(-6.34,-3.03) |
| Bahamas | male | 1.82(0.05,11.18) | 0.88(0.00,6.64) | -2.44(-2.76,-2.13) | 1.82(0.05,11.18) | 0.88(0.00,6.64) | -2.44(-2.76,-2.13) |
| Bahrain | male | 0.08(0.00,4.55) | 0.08(0.00,1.46) | -0.17(-1.11,0.79) | 0.08(0.00,4.55) | 0.08(0.00,1.46) | -0.17(-1.11,0.79) |
| Bangladesh | male | 0.73(0.62,0.86) | 0.62(0.53,0.71) | 0.02(-0.31,0.36) | 0.73(0.62,0.86) | 0.62(0.53,0.71) | 0.02(-0.31,0.36) |
| Barbados | male | 0.95(0.00,9.03) | 0.55(0.00,9.27) | -2.61(-3.17,-2.04) | 0.95(0.00,9.03) | 0.55(0.00,9.27) | -2.61(-3.17,-2.04) |
| Belarus | male | 2.28(1.66,3.07) | 0.83(0.43,1.50) | -3.59(-4.43,-2.74) | 2.28(1.66,3.07) | 0.83(0.43,1.50) | -3.59(-4.43,-2.74) |
| Belgium | male | 1.10(0.67,1.71) | 0.42(0.17,0.88) | -3.42(-3.77,-3.07) | 1.10(0.67,1.71) | 0.42(0.17,0.88) | -3.42(-3.77,-3.07) |
| Belize | male | 4.27(0.33,21.01) | 2.65(0.42,8.99) | -2.89(-3.64,-2.13) | 4.27(0.33,21.01) | 2.65(0.42,8.99) | -2.89(-3.64,-2.13) |
| Benin | male | 0.67(0.21,1.65) | 0.50(0.25,0.90) | -1.22(-1.56,-0.87) | 0.67(0.21,1.65) | 0.50(0.25,0.90) | -1.22(-1.56,-0.87) |
| Bermuda | male | 2.17(0.00,43.69) | 0.22(0.00,52.62) | -8.28(-8.95,-7.60) | 2.17(0.00,43.69) | 0.22(0.00,52.62) | -8.28(-8.95,-7.60) |
| Bhutan | male | 0.57(0.01,4.85) | 0.24(0.00,2.80) | -3.62(-3.96,-3.29) | 0.57(0.01,4.85) | 0.24(0.00,2.80) | -3.62(-3.96,-3.29) |
| Bolivia (Plurinational State of) | male | 0.27(0.06,0.81) | 0.14(0.04,0.40) | -2.09(-2.62,-1.55) | 0.27(0.06,0.81) | 0.14(0.04,0.40) | -2.09(-2.62,-1.55) |
| Bosnia and Herzegovina | male | 0.43(0.12,1.09) | 0.41(0.05,1.57) | 0.01(-0.47,0.50) | 0.43(0.12,1.09) | 0.41(0.05,1.57) | 0.01(-0.47,0.50) |
| Botswana | male | 0.30(0.00,2.68) | 0.20(0.01,1.11) | -1.86(-2.31,-1.41) | 0.30(0.00,2.68) | 0.20(0.01,1.11) | -1.86(-2.31,-1.41) |
| Brazil | male | 0.86(0.76,0.97) | 0.96(0.87,1.06) | 0.79(-0.13,1.71) | 0.86(0.76,0.97) | 0.96(0.87,1.06) | 0.79(-0.13,1.71) |
| Brunei Darussalam | male | 1.74(0.06,9.51) | 0.61(0.00,5.38) | -3.73(-4.53,-2.93) | 1.74(0.06,9.51) | 0.61(0.00,5.38) | -3.73(-4.53,-2.93) |
| Bulgaria | male | 0.44(0.17,0.93) | 0.48(0.14,1.23) | -0.15(-0.59,0.29) | 0.44(0.17,0.93) | 0.48(0.14,1.23) | -0.15(-0.59,0.29) |
| Burkina Faso | male | 0.71(0.33,1.38) | 0.79(0.53,1.14) | 0.76(0.45,1.07) | 0.71(0.33,1.38) | 0.79(0.53,1.14) | 0.76(0.45,1.07) |
| Burundi | male | 2.30(1.46,3.51) | 1.05(0.69,1.53) | -3.17(-3.52,-2.82) | 2.30(1.46,3.51) | 1.05(0.69,1.53) | -3.17(-3.52,-2.82) |
| Cabo Verde | male | 0.45(0.00,12.87) | 0.51(0.00,4.16) | 0.19(-0.41,0.79) | 0.45(0.00,12.87) | 0.51(0.00,4.16) | 0.19(-0.41,0.79) |
| Cambodia | male | 0.94(0.53,1.57) | 1.39(1.03,1.83) | 0.85(0.60,1.10) | 0.94(0.53,1.57) | 1.39(1.03,1.83) | 0.85(0.60,1.10) |
| Cameroon | male | 0.81(0.43,1.40) | 0.85(0.63,1.13) | 0.20(-0.04,0.44) | 0.81(0.43,1.40) | 0.85(0.63,1.13) | 0.20(-0.04,0.44) |
| Canada | male | 0.68(0.47,0.95) | 0.35(0.22,0.54) | -1.84(-2.16,-1.51) | 0.68(0.47,0.95) | 0.35(0.22,0.54) | -1.84(-2.16,-1.51) |
| Central African Republic | male | 0.91(0.27,2.39) | 0.78(0.33,1.59) | -0.51(-0.60,-0.43) | 0.91(0.27,2.39) | 0.78(0.33,1.59) | -0.51(-0.60,-0.43) |
| Chad | male | 0.32(0.06,1.01) | 0.55(0.30,0.94) | 2.09(1.63,2.56) | 0.32(0.06,1.01) | 0.55(0.30,0.94) | 2.09(1.63,2.56) |
| Chile | male | 0.40(0.20,0.73) | 0.57(0.35,0.88) | 1.46(0.50,2.43) | 0.40(0.20,0.73) | 0.57(0.35,0.88) | 1.46(0.50,2.43) |
| China | male | 0.98(0.94,1.02) | 1.52(1.48,1.57) | 1.33(0.61,2.05) | 0.98(0.94,1.02) | 1.52(1.48,1.57) | 1.33(0.61,2.05) |
| Colombia | male | 1.21(0.96,1.50) | 1.10(0.90,1.32) | -1.20(-1.91,-0.48) | 1.21(0.96,1.50) | 1.10(0.90,1.32) | -1.20(-1.91,-0.48) |
| Comoros | male | 0.82(0.00,7.93) | 0.69(0.02,3.91) | -1.60(-2.56,-0.62) | 0.82(0.00,7.93) | 0.69(0.02,3.91) | -1.60(-2.56,-0.62) |
| Congo | male | 0.75(0.17,2.32) | 0.54(0.19,1.21) | -1.31(-1.81,-0.82) | 0.75(0.17,2.32) | 0.54(0.19,1.21) | -1.31(-1.81,-0.82) |
| Cook Islands | male | 0.17(0.00,117.95) | 0.61(0.00,148.55) | 5.11(4.68,5.55) | 0.17(0.00,117.95) | 0.61(0.00,148.55) | 5.11(4.68,5.55) |
| Costa Rica | male | 1.44(0.66,2.80) | 1.28(0.66,2.27) | -1.23(-1.68,-0.77) | 1.44(0.66,2.80) | 1.28(0.66,2.27) | -1.23(-1.68,-0.77) |
| Croatia | male | 0.74(0.29,1.58) | 0.47(0.09,1.49) | -2.44(-3.28,-1.59) | 0.74(0.29,1.58) | 0.47(0.09,1.49) | -2.44(-3.28,-1.59) |
| Cuba | male | 11.47(10.16,12.92) | 1.78(1.23,2.53) | -7.30(-7.95,-6.64) | 11.47(10.16,12.92) | 1.78(1.23,2.53) | -7.30(-7.95,-6.64) |
| Cyprus | male | 0.49(0.00,3.46) | 0.21(0.00,2.67) | -3.65(-4.49,-2.79) | 0.49(0.00,3.46) | 0.21(0.00,2.67) | -3.65(-4.49,-2.79) |
| Czechia | male | 0.66(0.35,1.16) | 0.47(0.19,1.01) | -3.08(-4.38,-1.76) | 0.66(0.35,1.16) | 0.47(0.19,1.01) | -3.08(-4.38,-1.76) |
| Côte d'Ivoire | male | 0.46(0.22,0.86) | 0.55(0.37,0.78) | 0.26(0.01,0.51) | 0.46(0.22,0.86) | 0.55(0.37,0.78) | 0.26(0.01,0.51) |
| Democratic People's Republic of Korea | male | 1.13(0.82,1.52) | 1.76(1.42,2.16) | 1.19(0.99,1.40) | 1.13(0.82,1.52) | 1.76(1.42,2.16) | 1.19(0.99,1.40) |
| Democratic Republic of the Congo | male | 0.41(0.28,0.60) | 0.45(0.35,0.56) | 0.41(0.12,0.70) | 0.41(0.28,0.60) | 0.45(0.35,0.56) | 0.41(0.12,0.70) |
| Denmark | male | 1.16(0.58,2.09) | 0.31(0.06,0.98) | -4.81(-5.23,-4.39) | 1.16(0.58,2.09) | 0.31(0.06,0.98) | -4.81(-5.23,-4.39) |
| Djibouti | male | 0.54(0.00,6.60) | 0.64(0.07,2.46) | 0.45(0.13,0.77) | 0.54(0.00,6.60) | 0.64(0.07,2.46) | 0.45(0.13,0.77) |
| Dominica | male | 1.16(0.00,33.71) | 0.95(0.00,30.11) | -0.98(-1.18,-0.77) | 1.16(0.00,33.71) | 0.95(0.00,30.11) | -0.98(-1.18,-0.77) |
| Dominican Republic | male | 0.70(0.34,1.34) | 0.76(0.45,1.21) | 0.78(0.11,1.46) | 0.70(0.34,1.34) | 0.76(0.45,1.21) | 0.78(0.11,1.46) |
| Ecuador | male | 0.80(0.45,1.33) | 1.15(0.83,1.56) | 2.49(1.54,3.45) | 0.80(0.45,1.33) | 1.15(0.83,1.56) | 2.49(1.54,3.45) |
| Egypt | male | 0.31(0.21,0.43) | 0.36(0.28,0.45) | 1.45(0.84,2.06) | 0.31(0.21,0.43) | 0.36(0.28,0.45) | 1.45(0.84,2.06) |
| El Salvador | male | 0.69(0.27,1.51) | 0.66(0.28,1.35) | -0.35(-0.91,0.22) | 0.69(0.27,1.51) | 0.66(0.28,1.35) | -0.35(-0.91,0.22) |
| Equatorial Guinea | male | 0.78(0.00,8.61) | 0.47(0.04,2.16) | -1.56(-2.15,-0.97) | 0.78(0.00,8.61) | 0.47(0.04,2.16) | -1.56(-2.15,-0.97) |
| Eritrea | male | 1.48(0.68,2.93) | 1.28(0.76,2.04) | -1.14(-1.45,-0.82) | 1.48(0.68,2.93) | 1.28(0.76,2.04) | -1.14(-1.45,-0.82) |
| Estonia | male | 1.80(0.60,4.23) | 0.32(0.00,3.00) | -5.57(-6.43,-4.70) | 1.80(0.60,4.23) | 0.32(0.00,3.00) | -5.57(-6.43,-4.70) |
| Eswatini | male | 0.47(0.00,4.86) | 1.06(0.17,3.52) | 3.11(2.06,4.17) | 0.47(0.00,4.86) | 1.06(0.17,3.52) | 3.11(2.06,4.17) |
| Ethiopia | male | 1.00(0.80,1.24) | 0.42(0.34,0.52) | -3.11(-3.56,-2.67) | 1.00(0.80,1.24) | 0.42(0.34,0.52) | -3.11(-3.56,-2.67) |
| Fiji | male | 0.16(0.00,3.01) | 0.18(0.00,2.41) | 0.86(0.47,1.25) | 0.16(0.00,3.01) | 0.18(0.00,2.41) | 0.86(0.47,1.25) |
| Finland | male | 1.60(0.88,2.72) | 0.46(0.12,1.26) | -3.45(-3.79,-3.12) | 1.60(0.88,2.72) | 0.46(0.12,1.26) | -3.45(-3.79,-3.12) |
| France | male | 0.50(0.38,0.65) | 0.22(0.14,0.34) | -2.95(-3.29,-2.61) | 0.50(0.38,0.65) | 0.22(0.14,0.34) | -2.95(-3.29,-2.61) |
| Gabon | male | 0.88(0.08,3.75) | 0.75(0.13,2.46) | -0.72(-0.89,-0.54) | 0.88(0.08,3.75) | 0.75(0.13,2.46) | -0.72(-0.89,-0.54) |
| Gambia | male | 0.39(0.00,3.58) | 0.55(0.09,1.91) | 0.88(0.51,1.25) | 0.39(0.00,3.58) | 0.55(0.09,1.91) | 0.88(0.51,1.25) |
| Georgia | male | 0.82(0.36,1.58) | 0.45(0.08,1.50) | -1.51(-2.78,-0.22) | 0.82(0.36,1.58) | 0.45(0.08,1.50) | -1.51(-2.78,-0.22) |
| Germany | male | 0.87(0.72,1.05) | 0.31(0.22,0.43) | -3.94(-4.19,-3.68) | 0.87(0.72,1.05) | 0.31(0.22,0.43) | -3.94(-4.19,-3.68) |
| Ghana | male | 0.95(0.62,1.42) | 1.17(0.93,1.47) | 1.00(0.87,1.13) | 0.95(0.62,1.42) | 1.17(0.93,1.47) | 1.00(0.87,1.13) |
| Greece | male | 0.12(0.02,0.41) | 0.09(0.00,0.46) | -0.94(-1.20,-0.69) | 0.12(0.02,0.41) | 0.09(0.00,0.46) | -0.94(-1.20,-0.69) |
| Greenland | male | 0.77(0.00,42.92) | 0.23(0.00,45.27) | -4.18(-4.63,-3.73) | 0.77(0.00,42.92) | 0.23(0.00,45.27) | -4.18(-4.63,-3.73) |
| Grenada | male | 2.46(0.00,32.50) | 1.20(0.00,22.71) | -2.85(-3.40,-2.30) | 2.46(0.00,32.50) | 1.20(0.00,22.71) | -2.85(-3.40,-2.30) |
| Guam | male | 0.07(0.00,12.34) | 0.22(0.00,13.88) | 5.96(4.15,7.79) | 0.07(0.00,12.34) | 0.22(0.00,13.88) | 5.96(4.15,7.79) |
| Guatemala | male | 0.58(0.25,1.18) | 0.51(0.29,0.84) | -0.51(-0.90,-0.11) | 0.58(0.25,1.18) | 0.51(0.29,0.84) | -0.51(-0.90,-0.11) |
| Guinea | male | 0.35(0.08,1.04) | 0.43(0.20,0.84) | 0.70(0.60,0.79) | 0.35(0.08,1.04) | 0.43(0.20,0.84) | 0.70(0.60,0.79) |
| Guinea-Bissau | male | 1.40(0.21,5.21) | 1.20(0.36,3.06) | -0.42(-0.48,-0.35) | 1.40(0.21,5.21) | 1.20(0.36,3.06) | -0.42(-0.48,-0.35) |
| Guyana | male | 2.06(0.48,6.08) | 2.59(0.69,7.05) | 0.23(-0.54,0.99) | 2.06(0.48,6.08) | 2.59(0.69,7.05) | 0.23(-0.54,0.99) |
| Haiti | male | 2.82(1.93,4.00) | 1.86(1.37,2.46) | -1.16(-1.44,-0.88) | 2.82(1.93,4.00) | 1.86(1.37,2.46) | -1.16(-1.44,-0.88) |
| Honduras | male | 0.80(0.30,1.78) | 0.36(0.14,0.75) | -3.48(-3.95,-3.01) | 0.80(0.30,1.78) | 0.36(0.14,0.75) | -3.48(-3.95,-3.01) |
| Hungary | male | 2.82(2.11,3.70) | 1.01(0.55,1.72) | -4.41(-5.00,-3.83) | 2.82(2.11,3.70) | 1.01(0.55,1.72) | -4.41(-5.00,-3.83) |
| Iceland | male | 0.29(0.00,8.06) | 0.12(0.00,7.61) | -3.16(-3.68,-2.63) | 0.29(0.00,8.06) | 0.12(0.00,7.61) | -3.16(-3.68,-2.63) |
| India | male | 1.63(1.57,1.69) | 1.51(1.47,1.55) | -0.23(-0.63,0.16) | 1.63(1.57,1.69) | 1.51(1.47,1.55) | -0.23(-0.63,0.16) |
| Indonesia | male | 0.79(0.70,0.89) | 0.82(0.75,0.90) | 0.14(-0.05,0.32) | 0.79(0.70,0.89) | 0.82(0.75,0.90) | 0.14(-0.05,0.32) |
| Iran (Islamic Republic of) | male | 0.05(0.02,0.12) | 0.19(0.13,0.27) | 5.03(3.13,6.96) | 0.05(0.02,0.12) | 0.19(0.13,0.27) | 5.03(3.13,6.96) |
| Iraq | male | 0.20(0.08,0.43) | 0.13(0.07,0.23) | -1.19(-1.40,-0.99) | 0.20(0.08,0.43) | 0.13(0.07,0.23) | -1.19(-1.40,-0.99) |
| Ireland | male | 0.95(0.37,2.02) | 0.23(0.02,0.92) | -5.18(-5.71,-4.64) | 0.95(0.37,2.02) | 0.23(0.02,0.92) | -5.18(-5.71,-4.64) |
| Israel | male | 0.33(0.07,0.96) | 0.22(0.06,0.59) | -1.89(-2.61,-1.17) | 0.33(0.07,0.96) | 0.22(0.06,0.59) | -1.89(-2.61,-1.17) |
| Italy | male | 0.88(0.71,1.08) | 0.33(0.22,0.49) | -3.16(-3.35,-2.96) | 0.88(0.71,1.08) | 0.33(0.22,0.49) | -3.16(-3.35,-2.96) |
| Jamaica | male | 0.46(0.06,1.79) | 0.51(0.10,1.52) | -1.35(-2.44,-0.26) | 0.46(0.06,1.79) | 0.51(0.10,1.52) | -1.35(-2.44,-0.26) |
| Japan | male | 0.78(0.67,0.90) | 0.20(0.14,0.29) | -4.30(-4.54,-4.06) | 0.78(0.67,0.90) | 0.20(0.14,0.29) | -4.30(-4.54,-4.06) |
| Jordan | male | 0.09(0.00,0.92) | 0.13(0.04,0.35) | 2.55(1.82,3.28) | 0.09(0.00,0.92) | 0.13(0.04,0.35) | 2.55(1.82,3.28) |
| Kazakhstan | male | 0.58(0.35,0.90) | 0.27(0.13,0.52) | -1.51(-2.28,-0.74) | 0.58(0.35,0.90) | 0.27(0.13,0.52) | -1.51(-2.28,-0.74) |
| Kenya | male | 0.42(0.24,0.69) | 0.64(0.50,0.82) | 2.00(1.59,2.42) | 0.42(0.24,0.69) | 0.64(0.50,0.82) | 2.00(1.59,2.42) |
| Kiribati | male | 0.16(0.00,32.36) | 0.15(0.00,17.18) | -0.60(-0.83,-0.36) | 0.16(0.00,32.36) | 0.15(0.00,17.18) | -0.60(-0.83,-0.36) |
| Kuwait | male | 0.68(0.14,2.26) | 0.80(0.33,1.74) | 0.88(-1.48,3.29) | 0.68(0.14,2.26) | 0.80(0.33,1.74) | 0.88(-1.48,3.29) |
| Kyrgyzstan | male | 0.78(0.31,1.66) | 0.46(0.18,1.00) | -1.21(-2.28,-0.13) | 0.78(0.31,1.66) | 0.46(0.18,1.00) | -1.21(-2.28,-0.13) |
| Lao People's Democratic Republic | male | 1.17(0.51,2.33) | 1.10(0.65,1.75) | -0.43(-0.54,-0.33) | 1.17(0.51,2.33) | 1.10(0.65,1.75) | -0.43(-0.54,-0.33) |
| Latvia | male | 2.45(1.25,4.34) | 0.68(0.07,2.90) | -4.34(-4.81,-3.87) | 2.45(1.25,4.34) | 0.68(0.07,2.90) | -4.34(-4.81,-3.87) |
| Lebanon | male | 0.37(0.04,1.50) | 0.17(0.02,0.67) | -2.05(-2.61,-1.50) | 0.37(0.04,1.50) | 0.17(0.02,0.67) | -2.05(-2.61,-1.50) |
| Lesotho | male | 0.25(0.00,2.43) | 0.76(0.16,2.30) | 4.71(3.94,5.49) | 0.25(0.00,2.43) | 0.76(0.16,2.30) | 4.71(3.94,5.49) |
| Liberia | male | 0.42(0.04,1.67) | 0.43(0.13,1.08) | 0.65(0.25,1.05) | 0.42(0.04,1.67) | 0.43(0.13,1.08) | 0.65(0.25,1.05) |
| Libya | male | 0.03(0.00,0.59) | 0.08(0.00,0.41) | 4.71(4.01,5.42) | 0.03(0.00,0.59) | 0.08(0.00,0.41) | 4.71(4.01,5.42) |
| Lithuania | male | 2.48(1.46,3.97) | 0.90(0.23,2.59) | -2.95(-3.81,-2.08) | 2.48(1.46,3.97) | 0.90(0.23,2.59) | -2.95(-3.81,-2.08) |
| Luxembourg | male | 0.54(0.00,8.02) | 0.11(0.00,4.83) | -5.39(-5.75,-5.03) | 0.54(0.00,8.02) | 0.11(0.00,4.83) | -5.39(-5.75,-5.03) |
| Madagascar | male | 0.70(0.39,1.17) | 0.56(0.38,0.80) | -0.55(-0.75,-0.34) | 0.70(0.39,1.17) | 0.56(0.38,0.80) | -0.55(-0.75,-0.34) |
| Malawi | male | 0.72(0.38,1.27) | 0.90(0.62,1.28) | 0.65(0.33,0.97) | 0.72(0.38,1.27) | 0.90(0.62,1.28) | 0.65(0.33,0.97) |
| Malaysia | male | 1.20(0.88,1.62) | 1.24(1.00,1.53) | -0.67(-1.08,-0.27) | 1.20(0.88,1.62) | 1.24(1.00,1.53) | -0.67(-1.08,-0.27) |
| Maldives | male | 0.67(0.00,15.78) | 0.12(0.00,5.18) | -6.52(-7.37,-5.66) | 0.67(0.00,15.78) | 0.12(0.00,5.18) | -6.52(-7.37,-5.66) |
| Mali | male | 0.38(0.12,0.92) | 0.32(0.17,0.58) | -0.22(-0.49,0.05) | 0.38(0.12,0.92) | 0.32(0.17,0.58) | -0.22(-0.49,0.05) |
| Malta | male | 0.15(0.00,6.49) | 0.07(0.00,8.03) | -3.15(-3.50,-2.81) | 0.15(0.00,6.49) | 0.07(0.00,8.03) | -3.15(-3.50,-2.81) |
| Marshall Islands | male | 0.12(0.00,52.54) | 0.12(0.00,32.18) | 0.63(0.23,1.04) | 0.12(0.00,52.54) | 0.12(0.00,32.18) | 0.63(0.23,1.04) |
| Mauritania | male | 1.03(0.26,2.89) | 0.67(0.22,1.62) | -1.51(-1.69,-1.34) | 1.03(0.26,2.89) | 0.67(0.22,1.62) | -1.51(-1.69,-1.34) |
| Mauritius | male | 0.01(0.00,1.60) | 0.60(0.04,2.84) | 15.63(12.34,19.01) | 0.01(0.00,1.60) | 0.60(0.04,2.84) | 15.63(12.34,19.01) |
| Mexico | male | 1.28(1.11,1.47) | 0.86(0.75,0.98) | -1.22(-1.59,-0.84) | 1.28(1.11,1.47) | 0.86(0.75,0.98) | -1.22(-1.59,-0.84) |
| Micronesia (Federated States of) | male | 0.13(0.00,22.02) | 0.17(0.00,20.29) | 0.91(0.72,1.10) | 0.13(0.00,22.02) | 0.17(0.00,20.29) | 0.91(0.72,1.10) |
| Monaco | male | 0.31(0.00,120.14) | 0.24(0.00,88.33) | -1.09(-1.17,-1.01) | 0.31(0.00,120.14) | 0.24(0.00,88.33) | -1.09(-1.17,-1.01) |
| Mongolia | male | 0.03(0.00,1.33) | 0.13(0.00,0.93) | 5.96(5.30,6.63) | 0.03(0.00,1.33) | 0.13(0.00,0.93) | 5.96(5.30,6.63) |
| Montenegro | male | 0.76(0.02,4.39) | 0.45(0.00,4.82) | -1.71(-2.27,-1.15) | 0.76(0.02,4.39) | 0.45(0.00,4.82) | -1.71(-2.27,-1.15) |
| Morocco | male | 0.02(0.00,0.13) | 0.03(0.00,0.11) | 2.32(1.83,2.81) | 0.02(0.00,0.13) | 0.03(0.00,0.11) | 2.32(1.83,2.81) |
| Mozambique | male | 0.64(0.35,1.10) | 1.26(0.97,1.61) | 2.82(2.40,3.24) | 0.64(0.35,1.10) | 1.26(0.97,1.61) | 2.82(2.40,3.24) |
| Myanmar | male | 0.97(0.77,1.21) | 0.81(0.65,1.00) | -0.64(-0.77,-0.50) | 0.97(0.77,1.21) | 0.81(0.65,1.00) | -0.64(-0.77,-0.50) |
| Namibia | male | 0.31(0.00,2.51) | 0.59(0.12,1.82) | 1.71(0.97,2.46) | 0.31(0.00,2.51) | 0.59(0.12,1.82) | 1.71(0.97,2.46) |
| Nauru | male | 0.33(0.00,208.40) | 0.53(0.00,183.37) | 1.53(1.10,1.97) | 0.33(0.00,208.40) | 0.53(0.00,183.37) | 1.53(1.10,1.97) |
| Nepal | male | 0.79(0.52,1.16) | 0.62(0.44,0.87) | -0.60(-0.86,-0.35) | 0.79(0.52,1.16) | 0.62(0.44,0.87) | -0.60(-0.86,-0.35) |
| Netherlands | male | 1.66(1.23,2.21) | 0.62(0.36,1.01) | -3.64(-3.96,-3.32) | 1.66(1.23,2.21) | 0.62(0.36,1.01) | -3.64(-3.96,-3.32) |
| New Zealand | male | 1.03(0.42,2.12) | 0.33(0.07,1.01) | -3.48(-4.07,-2.89) | 1.03(0.42,2.12) | 0.33(0.07,1.01) | -3.48(-4.07,-2.89) |
| Nicaragua | male | 0.81(0.27,1.97) | 0.68(0.32,1.28) | -0.46(-0.73,-0.19) | 0.81(0.27,1.97) | 0.68(0.32,1.28) | -0.46(-0.73,-0.19) |
| Niger | male | 0.38(0.12,0.95) | 0.39(0.22,0.67) | 0.46(0.32,0.60) | 0.38(0.12,0.95) | 0.39(0.22,0.67) | 0.46(0.32,0.60) |
| Nigeria | male | 0.37(0.28,0.48) | 0.40(0.34,0.47) | 0.29(0.13,0.45) | 0.37(0.28,0.48) | 0.40(0.34,0.47) | 0.29(0.13,0.45) |
| Niue | male | 0.12(0.00,1030.84) | 0.18(0.00,1427.94) | 0.80(0.50,1.11) | 0.12(0.00,1030.84) | 0.18(0.00,1427.94) | 0.80(0.50,1.11) |
| North Macedonia | male | 0.21(0.00,1.33) | 0.14(0.00,1.54) | -1.82(-2.37,-1.27) | 0.21(0.00,1.33) | 0.14(0.00,1.54) | -1.82(-2.37,-1.27) |
| Northern Mariana Islands | male | 0.03(0.00,43.24) | 0.09(0.00,49.46) | 4.70(2.81,6.63) | 0.03(0.00,43.24) | 0.09(0.00,49.46) | 4.70(2.81,6.63) |
| Norway | male | 0.55(0.17,1.37) | 0.17(0.01,0.79) | -2.97(-3.55,-2.38) | 0.55(0.17,1.37) | 0.17(0.01,0.79) | -2.97(-3.55,-2.38) |
| Oman | male | 0.24(0.01,1.31) | 0.40(0.13,1.13) | 3.31(2.34,4.29) | 0.24(0.01,1.31) | 0.40(0.13,1.13) | 3.31(2.34,4.29) |
| Pakistan | male | 0.79(0.67,0.93) | 1.36(1.25,1.46) | 1.63(1.27,1.98) | 0.79(0.67,0.93) | 1.36(1.25,1.46) | 1.63(1.27,1.98) |
| Palau | male | 0.00(0.00,114.81) | 0.00(0.00,132.96) | 1.16(1.01,1.30) | 0.00(0.00,114.81) | 0.00(0.00,132.96) | 1.16(1.01,1.30) |
| Palestine | male | 0.03(0.00,1.68) | 0.03(0.00,0.50) | 0.07(-0.84,0.99) | 0.03(0.00,1.68) | 0.03(0.00,0.50) | 0.07(-0.84,0.99) |
| Panama | male | 1.67(0.74,3.31) | 1.09(0.50,2.07) | -1.69(-2.05,-1.32) | 1.67(0.74,3.31) | 1.09(0.50,2.07) | -1.69(-2.05,-1.32) |
| Papua New Guinea | male | 0.12(0.00,0.77) | 0.10(0.01,0.36) | -0.68(-0.88,-0.49) | 0.12(0.00,0.77) | 0.10(0.01,0.36) | -0.68(-0.88,-0.49) |
| Paraguay | male | 0.17(0.01,0.85) | 0.37(0.13,0.82) | 2.66(2.05,3.27) | 0.17(0.01,0.85) | 0.37(0.13,0.82) | 2.66(2.05,3.27) |
| Peru | male | 0.64(0.42,0.95) | 0.95(0.75,1.20) | 2.40(1.72,3.09) | 0.64(0.42,0.95) | 0.95(0.75,1.20) | 2.40(1.72,3.09) |
| Philippines | male | 0.58(0.45,0.73) | 0.68(0.58,0.79) | 0.94(0.66,1.22) | 0.58(0.45,0.73) | 0.68(0.58,0.79) | 0.94(0.66,1.22) |
| Poland | male | 1.71(1.42,2.05) | 1.09(0.84,1.40) | -2.73(-3.39,-2.07) | 1.71(1.42,2.05) | 1.09(0.84,1.40) | -2.73(-3.39,-2.07) |
| Portugal | male | 1.04(0.63,1.61) | 0.29(0.08,0.74) | -5.15(-5.52,-4.77) | 1.04(0.63,1.61) | 0.29(0.08,0.74) | -5.15(-5.52,-4.77) |
| Puerto Rico | male | 1.64(0.82,2.94) | 1.17(0.43,2.57) | -2.43(-3.23,-1.62) | 1.64(0.82,2.94) | 1.17(0.43,2.57) | -2.43(-3.23,-1.62) |
| Qatar | male | 0.44(0.00,5.83) | 0.40(0.05,2.06) | 0.62(0.08,1.15) | 0.44(0.00,5.83) | 0.40(0.05,2.06) | 0.62(0.08,1.15) |
| Republic of Korea | male | 2.25(1.97,2.55) | 0.23(0.13,0.37) | -9.46(-10.37,-8.53) | 2.25(1.97,2.55) | 0.23(0.13,0.37) | -9.46(-10.37,-8.53) |
| Republic of Moldova | male | 1.37(0.70,2.45) | 0.38(0.05,1.48) | -2.95(-3.57,-2.33) | 1.37(0.70,2.45) | 0.38(0.05,1.48) | -2.95(-3.57,-2.33) |
| Romania | male | 1.41(1.08,1.82) | 0.80(0.50,1.22) | -1.50(-1.73,-1.27) | 1.41(1.08,1.82) | 0.80(0.50,1.22) | -1.50(-1.73,-1.27) |
| Russian Federation | male | 0.68(0.58,0.78) | 0.34(0.27,0.43) | -1.76(-2.66,-0.84) | 0.68(0.58,0.78) | 0.34(0.27,0.43) | -1.76(-2.66,-0.84) |
| Rwanda | male | 4.22(3.20,5.49) | 1.63(1.19,2.18) | -4.57(-5.20,-3.94) | 4.22(3.20,5.49) | 1.63(1.19,2.18) | -4.57(-5.20,-3.94) |
| Saint Kitts and Nevis | male | 2.08(0.00,58.13) | 0.58(0.00,39.42) | -5.55(-6.41,-4.68) | 2.08(0.00,58.13) | 0.58(0.00,39.42) | -5.55(-6.41,-4.68) |
| Saint Lucia | male | 2.82(0.02,24.05) | 1.14(0.00,14.84) | -3.76(-4.20,-3.31) | 2.82(0.02,24.05) | 1.14(0.00,14.84) | -3.76(-4.20,-3.31) |
| Saint Vincent and the Grenadines | male | 1.78(0.00,26.49) | 0.94(0.00,20.52) | -2.95(-3.40,-2.50) | 1.78(0.00,26.49) | 0.94(0.00,20.52) | -2.95(-3.40,-2.50) |
| Samoa | male | 0.08(0.00,16.65) | 0.14(0.00,11.36) | 2.12(1.93,2.31) | 0.08(0.00,16.65) | 0.14(0.00,11.36) | 2.12(1.93,2.31) |
| San Marino | male | 1.28(0.00,88.33) | 0.77(0.00,88.12) | -1.17(-1.43,-0.92) | 1.28(0.00,88.33) | 0.77(0.00,88.12) | -1.17(-1.43,-0.92) |
| Sao Tome and Principe | male | 0.31(0.00,30.49) | 0.50(0.00,10.16) | 1.57(1.10,2.05) | 0.31(0.00,30.49) | 0.50(0.00,10.16) | 1.57(1.10,2.05) |
| Saudi Arabia | male | 0.01(0.00,0.13) | 0.04(0.01,0.12) | 8.37(7.43,9.31) | 0.01(0.00,0.13) | 0.04(0.01,0.12) | 8.37(7.43,9.31) |
| Senegal | male | 0.39(0.12,0.99) | 0.35(0.17,0.65) | 0.02(-0.21,0.26) | 0.39(0.12,0.99) | 0.35(0.17,0.65) | 0.02(-0.21,0.26) |
| Serbia | male | 1.60(1.07,2.30) | 0.59(0.28,1.13) | -3.12(-3.34,-2.90) | 1.60(1.07,2.30) | 0.59(0.28,1.13) | -3.12(-3.34,-2.90) |
| Seychelles | male | 0.46(0.00,30.69) | 0.42(0.00,22.95) | 0.37(-0.48,1.24) | 0.46(0.00,30.69) | 0.42(0.00,22.95) | 0.37(-0.48,1.24) |
| Sierra Leone | male | 0.55(0.14,1.47) | 0.52(0.23,1.02) | 0.01(-0.14,0.16) | 0.55(0.14,1.47) | 0.52(0.23,1.02) | 0.01(-0.14,0.16) |
| Singapore | male | 0.73(0.25,1.67) | 0.10(0.00,0.81) | -6.92(-7.17,-6.67) | 0.73(0.25,1.67) | 0.10(0.00,0.81) | -6.92(-7.17,-6.67) |
| Slovakia | male | 2.32(1.49,3.47) | 0.89(0.37,1.87) | -3.42(-3.81,-3.03) | 2.32(1.49,3.47) | 0.89(0.37,1.87) | -3.42(-3.81,-3.03) |
| Slovenia | male | 1.21(0.37,2.98) | 0.41(0.01,2.38) | -4.78(-5.51,-4.04) | 1.21(0.37,2.98) | 0.41(0.01,2.38) | -4.78(-5.51,-4.04) |
| Solomon Islands | male | 0.11(0.00,8.49) | 0.16(0.00,3.53) | 1.46(1.23,1.70) | 0.11(0.00,8.49) | 0.16(0.00,3.53) | 1.46(1.23,1.70) |
| Somalia | male | 0.88(0.47,1.53) | 1.21(0.89,1.61) | 1.40(1.19,1.61) | 0.88(0.47,1.53) | 1.21(0.89,1.61) | 1.40(1.19,1.61) |
| South Africa | male | 1.03(0.81,1.31) | 0.77(0.62,0.94) | -1.71(-2.38,-1.04) | 1.03(0.81,1.31) | 0.77(0.62,0.94) | -1.71(-2.38,-1.04) |
| South Sudan | male | 0.53(0.19,1.23) | 0.82(0.43,1.44) | 1.35(0.94,1.75) | 0.53(0.19,1.23) | 0.82(0.43,1.44) | 1.35(0.94,1.75) |
| Spain | male | 0.53(0.38,0.72) | 0.19(0.10,0.34) | -4.46(-4.96,-3.95) | 0.53(0.38,0.72) | 0.19(0.10,0.34) | -4.46(-4.96,-3.95) |
| Sri Lanka | male | 1.08(0.77,1.47) | 0.94(0.66,1.30) | -1.22(-1.84,-0.60) | 1.08(0.77,1.47) | 0.94(0.66,1.30) | -1.22(-1.84,-0.60) |
| Sudan | male | 0.02(0.00,0.18) | 0.04(0.01,0.12) | 3.19(2.62,3.76) | 0.02(0.00,0.18) | 0.04(0.01,0.12) | 3.19(2.62,3.76) |
| Suriname | male | 4.06(0.88,12.74) | 1.88(0.23,6.94) | -2.88(-3.27,-2.50) | 4.06(0.88,12.74) | 1.88(0.23,6.94) | -2.88(-3.27,-2.50) |
| Sweden | male | 0.69(0.33,1.28) | 0.13(0.02,0.48) | -4.85(-5.49,-4.20) | 0.69(0.33,1.28) | 0.13(0.02,0.48) | -4.85(-5.49,-4.20) |
| Switzerland | male | 1.29(0.74,2.12) | 0.24(0.05,0.74) | -5.43(-5.83,-5.04) | 1.29(0.74,2.12) | 0.24(0.05,0.74) | -5.43(-5.83,-5.04) |
| Syrian Arab Republic | male | 0.21(0.07,0.54) | 0.15(0.02,0.63) | -0.88(-1.36,-0.40) | 0.21(0.07,0.54) | 0.15(0.02,0.63) | -0.88(-1.36,-0.40) |
| Taiwan (Province of China) | male | 0.01(0.00,0.11) | 0.62(0.39,0.95) | 15.92(12.92,18.99) | 0.01(0.00,0.11) | 0.62(0.39,0.95) | 15.92(12.92,18.99) |
| Tajikistan | male | 0.07(0.00,0.63) | 0.03(0.00,0.25) | -3.46(-4.69,-2.22) | 0.07(0.00,0.63) | 0.03(0.00,0.25) | -3.46(-4.69,-2.22) |
| Thailand | male | 0.84(0.69,1.01) | 1.27(1.07,1.51) | -0.73(-1.66,0.20) | 0.84(0.69,1.01) | 1.27(1.07,1.51) | -0.73(-1.66,0.20) |
| Timor-Leste | male | 0.39(0.00,3.54) | 0.51(0.03,2.84) | 0.91(0.33,1.49) | 0.39(0.00,3.54) | 0.51(0.03,2.84) | 0.91(0.33,1.49) |
| Togo | male | 0.75(0.22,1.97) | 0.85(0.45,1.48) | 0.53(0.26,0.80) | 0.75(0.22,1.97) | 0.85(0.45,1.48) | 0.53(0.26,0.80) |
| Tokelau | male | 0.06(0.00,1579.35) | 0.10(0.00,1640.69) | 1.23(1.00,1.47) | 0.06(0.00,1579.35) | 0.10(0.00,1640.69) | 1.23(1.00,1.47) |
| Tonga | male | 0.04(0.00,33.95) | 0.09(0.00,24.83) | 2.33(1.86,2.81) | 0.04(0.00,33.95) | 0.09(0.00,24.83) | 2.33(1.86,2.81) |
| Trinidad and Tobago | male | 1.55(0.41,4.13) | 1.20(0.24,3.70) | -1.01(-1.37,-0.64) | 1.55(0.41,4.13) | 1.20(0.24,3.70) | -1.01(-1.37,-0.64) |
| Tunisia | male | 0.46(0.19,0.96) | 0.56(0.29,0.98) | 0.30(0.15,0.45) | 0.46(0.19,0.96) | 0.56(0.29,0.98) | 0.30(0.15,0.45) |
| Turkey | male | 0.36(0.26,0.48) | 0.43(0.34,0.55) | 1.62(0.40,2.87) | 0.36(0.26,0.48) | 0.43(0.34,0.55) | 1.62(0.40,2.87) |
| Turkmenistan | male | 0.39(0.07,1.27) | 0.18(0.02,0.67) | -1.96(-2.72,-1.21) | 0.39(0.07,1.27) | 0.18(0.02,0.67) | -1.96(-2.72,-1.21) |
| Tuvalu | male | 0.14(0.00,245.18) | 0.20(0.00,155.47) | 1.36(1.10,1.62) | 0.14(0.00,245.18) | 0.20(0.00,155.47) | 1.36(1.10,1.62) |
| Uganda | male | 1.07(0.73,1.54) | 1.94(1.64,2.28) | 1.12(0.51,1.75) | 1.07(0.73,1.54) | 1.94(1.64,2.28) | 1.12(0.51,1.75) |
| Ukraine | male | 0.67(0.51,0.86) | 0.35(0.22,0.52) | -1.73(-2.50,-0.96) | 0.67(0.51,0.86) | 0.35(0.22,0.52) | -1.73(-2.50,-0.96) |
| United Arab Emirates | male | 1.10(0.36,2.82) | 1.34(0.74,2.27) | 1.33(0.26,2.41) | 1.10(0.36,2.82) | 1.34(0.74,2.27) | 1.33(0.26,2.41) |
| United Kingdom | male | 0.59(0.45,0.77) | 0.25(0.16,0.36) | -3.64(-4.17,-3.11) | 0.59(0.45,0.77) | 0.25(0.16,0.36) | -3.64(-4.17,-3.11) |
| United Republic of Tanzania | male | 0.93(0.66,1.27) | 0.83(0.66,1.02) | -0.59(-0.76,-0.41) | 0.93(0.66,1.27) | 0.83(0.66,1.02) | -0.59(-0.76,-0.41) |
| United States of America | male | 0.83(0.75,0.91) | 0.43(0.37,0.48) | -2.16(-2.62,-1.70) | 0.83(0.75,0.91) | 0.43(0.37,0.48) | -2.16(-2.62,-1.70) |
| United States Virgin Islands | male | 1.86(0.00,25.23) | 2.20(0.00,41.59) | 0.39(-0.12,0.90) | 1.86(0.00,25.23) | 2.20(0.00,41.59) | 0.39(-0.12,0.90) |
| Uruguay | male | 0.25(0.02,1.14) | 0.34(0.04,1.25) | 0.93(0.37,1.48) | 0.25(0.02,1.14) | 0.34(0.04,1.25) | 0.93(0.37,1.48) |
| Uzbekistan | male | 0.35(0.20,0.60) | 0.75(0.56,0.98) | 4.02(2.61,5.45) | 0.35(0.20,0.60) | 0.75(0.56,0.98) | 4.02(2.61,5.45) |
| Vanuatu | male | 0.14(0.00,15.90) | 0.22(0.00,7.65) | 1.38(1.28,1.47) | 0.14(0.00,15.90) | 0.22(0.00,7.65) | 1.38(1.28,1.47) |
| Venezuela (Bolivarian Republic of) | male | 0.89(0.62,1.25) | 0.64(0.42,0.94) | -1.34(-1.73,-0.96) | 0.89(0.62,1.25) | 0.64(0.42,0.94) | -1.34(-1.73,-0.96) |
| Viet Nam | male | 0.80(0.65,0.97) | 1.24(1.08,1.40) | 1.98(1.68,2.27) | 0.80(0.65,0.97) | 1.24(1.08,1.40) | 1.98(1.68,2.27) |
| Yemen | male | 0.04(0.00,0.28) | 0.05(0.01,0.15) | 2.14(1.59,2.68) | 0.04(0.00,0.28) | 0.05(0.01,0.15) | 2.14(1.59,2.68) |
| Zambia | male | 1.56(0.98,2.44) | 1.98(1.55,2.49) | 0.22(-0.14,0.59) | 1.56(0.98,2.44) | 1.98(1.55,2.49) | 0.22(-0.14,0.59) |
| Zimbabwe | male | 0.08(0.01,0.42) | 0.81(0.51,1.23) | 8.99(6.51,11.52) | 0.08(0.01,0.42) | 0.81(0.51,1.23) | 8.99(6.51,11.52) |

Table S7 Cases number, age standardized DALYs rate and its trends of cyclist road injuries age 15-39 in 204 countries, 1990-2021

| DALYs (Disability-Adjusted Life Years) |  | DALY No.(95%UI) |  |  | Age-standardized DALY rate (per 100000) No.95%UI |  |  |
| --- | --- | --- | --- | --- | --- | --- | --- |
| nation | sex | 1990 | 2021 | 1990-2021 EAPC No.(95%CI) | 1990 | 2021 | 1990-2021 EAPC No.(95%CI) |
| Afghanistan | both | 9.13(7.91,10.52) | 9.28(8.70,9.89) | 0.47(0.23,0.71) | 9.13(7.91,10.52) | 9.28(8.70,9.89) | 0.47(0.23,0.71) |
| Albania | both | 33.83(30.84,37.05) | 26.85(23.66,30.37) | -0.78(-1.18,-0.39) | 33.83(30.84,37.05) | 26.85(23.66,30.37) | -0.78(-1.18,-0.39) |
| Algeria | both | 14.73(13.95,15.55) | 9.84(9.37,10.32) | -1.24(-1.31,-1.17) | 14.73(13.95,15.55) | 9.84(9.37,10.32) | -1.24(-1.31,-1.17) |
| American Samoa | both | 6.88(0.36,34.98) | 9.40(0.71,41.05) | 1.89(1.20,2.58) | 6.88(0.36,34.98) | 9.40(0.71,41.05) | 1.89(1.20,2.58) |
| Andorra | both | 70.03(40.32,115.14) | 45.22(22.59,82.76) | -1.31(-1.61,-1.01) | 70.03(40.32,115.14) | 45.22(22.59,82.76) | -1.31(-1.61,-1.01) |
| Angola | both | 22.95(21.46,24.51) | 23.06(22.20,23.95) | 0.62(0.28,0.95) | 22.95(21.46,24.51) | 23.06(22.20,23.95) | 0.62(0.28,0.95) |
| Antigua and Barbuda | both | 58.02(32.36,96.89) | 20.69(8.31,43.46) | -3.74(-4.28,-3.19) | 58.02(32.36,96.89) | 20.69(8.31,43.46) | -3.74(-4.28,-3.19) |
| Argentina | both | 51.81(50.54,53.11) | 32.59(31.75,33.44) | -1.60(-2.00,-1.19) | 51.81(50.54,53.11) | 32.59(31.75,33.44) | -1.60(-2.00,-1.19) |
| Armenia | both | 13.38(11.56,15.42) | 22.47(19.68,25.57) | 2.69(1.67,3.73) | 13.38(11.56,15.42) | 22.47(19.68,25.57) | 2.69(1.67,3.73) |
| Australia | both | 45.53(43.93,47.18) | 16.92(16.06,17.82) | -3.03(-3.26,-2.80) | 45.53(43.93,47.18) | 16.92(16.06,17.82) | -3.03(-3.26,-2.80) |
| Austria | both | 65.01(62.10,68.03) | 27.70(25.75,29.77) | -3.02(-3.17,-2.88) | 65.01(62.10,68.03) | 27.70(25.75,29.77) | -3.02(-3.17,-2.88) |
| Azerbaijan | both | 13.51(12.23,14.89) | 7.78(6.97,8.67) | -2.40(-3.04,-1.76) | 13.51(12.23,14.89) | 7.78(6.97,8.67) | -2.40(-3.04,-1.76) |
| Bahamas | both | 82.70(67.02,101.20) | 41.68(32.13,53.21) | -2.30(-2.53,-2.07) | 82.70(67.02,101.20) | 41.68(32.13,53.21) | -2.30(-2.53,-2.07) |
| Bahrain | both | 15.34(10.90,21.27) | 10.76(8.49,13.55) | -1.18(-1.44,-0.91) | 15.34(10.90,21.27) | 10.76(8.49,13.55) | -1.18(-1.44,-0.91) |
| Bangladesh | both | 32.22(31.68,32.77) | 24.18(23.81,24.55) | -0.45(-0.71,-0.18) | 32.22(31.68,32.77) | 24.18(23.81,24.55) | -0.45(-0.71,-0.18) |
| Barbados | both | 46.38(34.49,61.13) | 29.13(19.44,42.12) | -2.16(-2.56,-1.75) | 46.38(34.49,61.13) | 29.13(19.44,42.12) | -2.16(-2.56,-1.75) |
| Belarus | both | 102.74(99.57,105.99) | 42.40(40.03,44.90) | -3.15(-3.87,-2.42) | 102.74(99.57,105.99) | 42.40(40.03,44.90) | -3.15(-3.87,-2.42) |
| Belgium | both | 71.77(69.02,74.60) | 32.50(30.63,34.46) | -2.86(-3.12,-2.60) | 71.77(69.02,74.60) | 32.50(30.63,34.46) | -2.86(-3.12,-2.60) |
| Belize | both | 194.65(163.62,230.59) | 107.99(93.59,124.04) | -3.07(-3.70,-2.43) | 194.65(163.62,230.59) | 107.99(93.59,124.04) | -3.07(-3.70,-2.43) |
| Benin | both | 27.13(24.66,29.80) | 20.84(19.57,22.17) | -1.12(-1.39,-0.85) | 27.13(24.66,29.80) | 20.84(19.57,22.17) | -1.12(-1.39,-0.85) |
| Bermuda | both | 90.83(57.12,139.33) | 15.71(2.84,51.62) | -6.19(-6.68,-5.69) | 90.83(57.12,139.33) | 15.71(2.84,51.62) | -6.19(-6.68,-5.69) |
| Bhutan | both | 31.70(25.14,39.65) | 14.81(11.03,19.52) | -3.08(-3.30,-2.87) | 31.70(25.14,39.65) | 14.81(11.03,19.52) | -3.08(-3.30,-2.87) |
| Bolivia (Plurinational State of) | both | 22.09(20.22,24.08) | 13.05(12.06,14.11) | -1.67(-1.77,-1.56) | 22.09(20.22,24.08) | 13.05(12.06,14.11) | -1.67(-1.77,-1.56) |
| Bosnia and Herzegovina | both | 33.99(31.43,36.71) | 29.61(26.33,33.21) | -0.32(-0.55,-0.09) | 33.99(31.43,36.71) | 29.61(26.33,33.21) | -0.32(-0.55,-0.09) |
| Botswana | both | 14.99(11.76,18.92) | 13.07(10.99,15.44) | -0.65(-0.95,-0.35) | 14.99(11.76,18.92) | 13.07(10.99,15.44) | -0.65(-0.95,-0.35) |
| Brazil | both | 47.20(46.66,47.75) | 48.76(48.30,49.24) | 0.28(-0.47,1.03) | 47.20(46.66,47.75) | 48.76(48.30,49.24) | 0.28(-0.47,1.03) |
| Brunei Darussalam | both | 108.55(90.80,128.84) | 44.58(35.94,54.86) | -3.03(-3.55,-2.51) | 108.55(90.80,128.84) | 44.58(35.94,54.86) | -3.03(-3.55,-2.51) |
| Bulgaria | both | 44.13(41.78,46.58) | 37.50(34.76,40.43) | -0.73(-1.01,-0.45) | 44.13(41.78,46.58) | 37.50(34.76,40.43) | -0.73(-1.01,-0.45) |
| Burkina Faso | both | 29.22(27.31,31.24) | 31.57(30.36,32.82) | 0.66(0.40,0.91) | 29.22(27.31,31.24) | 31.57(30.36,32.82) | 0.66(0.40,0.91) |
| Burundi | both | 98.77(94.53,103.16) | 48.82(46.94,50.77) | -2.75(-3.02,-2.47) | 98.77(94.53,103.16) | 48.82(46.94,50.77) | -2.75(-3.02,-2.47) |
| Cabo Verde | both | 18.15(11.08,28.71) | 20.72(15.47,27.23) | 0.22(-0.29,0.74) | 18.15(11.08,28.71) | 20.72(15.47,27.23) | 0.22(-0.29,0.74) |
| Cambodia | both | 45.33(43.19,47.56) | 62.46(60.65,64.31) | 0.58(0.35,0.81) | 45.33(43.19,47.56) | 62.46(60.65,64.31) | 0.58(0.35,0.81) |
| Cameroon | both | 33.09(31.23,35.05) | 34.80(33.76,35.87) | 0.21(0.00,0.42) | 33.09(31.23,35.05) | 34.80(33.76,35.87) | 0.21(0.00,0.42) |
| Canada | both | 47.55(46.24,48.89) | 25.74(24.83,26.68) | -1.79(-2.01,-1.58) | 47.55(46.24,48.89) | 25.74(24.83,26.68) | -1.79(-2.01,-1.58) |
| Central African Republic | both | 35.10(31.56,38.97) | 29.75(27.48,32.18) | -0.52(-0.61,-0.43) | 35.10(31.56,38.97) | 29.75(27.48,32.18) | -0.52(-0.61,-0.43) |
| Chad | both | 15.45(13.78,17.29) | 22.97(21.74,24.25) | 1.53(1.19,1.86) | 15.45(13.78,17.29) | 22.97(21.74,24.25) | 1.53(1.19,1.86) |
| Chile | both | 31.01(29.57,32.50) | 38.96(37.52,40.44) | 0.41(-0.16,0.98) | 31.01(29.57,32.50) | 38.96(37.52,40.44) | 0.41(-0.16,0.98) |
| China | both | 71.39(71.16,71.61) | 82.71(82.44,82.97) | 0.24(-0.29,0.78) | 71.39(71.16,71.61) | 82.71(82.44,82.97) | 0.24(-0.29,0.78) |
| Colombia | both | 52.50(51.30,53.73) | 49.55(48.57,50.53) | -0.97(-1.55,-0.38) | 52.50(51.30,53.73) | 49.55(48.57,50.53) | -0.97(-1.55,-0.38) |
| Comoros | both | 39.28(30.35,50.34) | 33.60(27.45,40.77) | -1.37(-2.11,-0.63) | 39.28(30.35,50.34) | 33.60(27.45,40.77) | -1.37(-2.11,-0.63) |
| Congo | both | 29.06(25.62,32.88) | 21.96(20.04,24.01) | -1.11(-1.45,-0.77) | 29.06(25.62,32.88) | 21.96(20.04,24.01) | -1.11(-1.45,-0.77) |
| Cook Islands | both | 14.22(0.41,83.60) | 26.76(2.06,115.19) | 2.57(2.26,2.88) | 14.22(0.41,83.60) | 26.76(2.06,115.19) | 2.57(2.26,2.88) |
| Costa Rica | both | 62.50(58.22,67.04) | 50.95(47.79,54.27) | -1.41(-1.78,-1.03) | 62.50(58.22,67.04) | 50.95(47.79,54.27) | -1.41(-1.78,-1.03) |
| Croatia | both | 55.77(52.38,59.33) | 36.16(32.88,39.70) | -1.92(-2.54,-1.29) | 55.77(52.38,59.33) | 36.16(32.88,39.70) | -1.92(-2.54,-1.29) |
| Cuba | both | 455.14(449.15,461.20) | 76.70(73.83,79.65) | -6.99(-7.60,-6.37) | 455.14(449.15,461.20) | 76.70(73.83,79.65) | -6.99(-7.60,-6.37) |
| Cyprus | both | 45.31(38.09,53.55) | 23.70(19.57,28.61) | -2.58(-3.04,-2.12) | 45.31(38.09,53.55) | 23.70(19.57,28.61) | -2.58(-3.04,-2.12) |
| Czechia | both | 59.52(57.06,62.07) | 35.57(33.42,37.83) | -2.84(-3.66,-2.01) | 59.52(57.06,62.07) | 35.57(33.42,37.83) | -2.84(-3.66,-2.01) |
| Côte d'Ivoire | both | 21.37(20.03,22.78) | 23.99(23.08,24.92) | 0.19(0.01,0.38) | 21.37(20.03,22.78) | 23.99(23.08,24.92) | 0.19(0.01,0.38) |
| Democratic People's Republic of Korea | both | 60.67(59.00,62.38) | 87.98(86.16,89.83) | 1.10(0.91,1.28) | 60.67(59.00,62.38) | 87.98(86.16,89.83) | 1.10(0.91,1.28) |
| Democratic Republic of the Congo | both | 21.05(20.29,21.83) | 20.00(19.53,20.47) | -0.14(-0.30,0.01) | 21.05(20.29,21.83) | 20.00(19.53,20.47) | -0.14(-0.30,0.01) |
| Denmark | both | 100.57(96.09,105.21) | 30.56(28.06,33.24) | -4.28(-4.53,-4.03) | 100.57(96.09,105.21) | 30.56(28.06,33.24) | -4.28(-4.53,-4.03) |
| Djibouti | both | 26.25(19.05,35.61) | 28.67(24.33,33.57) | 0.14(-0.12,0.40) | 26.25(19.05,35.61) | 28.67(24.33,33.57) | 0.14(-0.12,0.40) |
| Dominica | both | 61.85(36.57,99.59) | 48.85(25.75,84.29) | -1.11(-1.25,-0.97) | 61.85(36.57,99.59) | 48.85(25.75,84.29) | -1.11(-1.25,-0.97) |
| Dominican Republic | both | 41.13(38.83,43.53) | 39.65(37.84,41.52) | 0.27(-0.12,0.67) | 41.13(38.83,43.53) | 39.65(37.84,41.52) | 0.27(-0.12,0.67) |
| Ecuador | both | 38.21(36.30,40.20) | 51.50(49.86,53.17) | 2.08(1.29,2.87) | 38.21(36.30,40.20) | 51.50(49.86,53.17) | 2.08(1.29,2.87) |
| Egypt | both | 23.26(22.61,23.91) | 20.47(20.04,20.90) | 0.19(-0.19,0.57) | 23.26(22.61,23.91) | 20.47(20.04,20.90) | 0.19(-0.19,0.57) |
| El Salvador | both | 39.44(36.74,42.30) | 31.72(29.56,33.99) | -0.96(-1.31,-0.60) | 39.44(36.74,42.30) | 31.72(29.56,33.99) | -0.96(-1.31,-0.60) |
| Equatorial Guinea | both | 28.76(20.82,38.99) | 22.20(18.78,26.12) | -0.73(-1.14,-0.32) | 28.76(20.82,38.99) | 22.20(18.78,26.12) | -0.73(-1.14,-0.32) |
| Eritrea | both | 56.32(52.28,60.62) | 50.95(48.32,53.70) | -0.87(-1.13,-0.60) | 56.32(52.28,60.62) | 50.95(48.32,53.70) | -0.87(-1.13,-0.60) |
| Estonia | both | 90.66(82.94,98.92) | 22.21(17.73,27.57) | -4.63(-5.25,-4.02) | 90.66(82.94,98.92) | 22.21(17.73,27.57) | -4.63(-5.25,-4.02) |
| Eswatini | both | 20.29(15.27,26.60) | 38.44(33.16,44.35) | 2.54(1.72,3.37) | 20.29(15.27,26.60) | 38.44(33.16,44.35) | 2.54(1.72,3.37) |
| Ethiopia | both | 44.19(43.23,45.17) | 19.94(19.52,20.36) | -2.97(-3.29,-2.65) | 44.19(43.23,45.17) | 19.94(19.52,20.36) | -2.97(-3.29,-2.65) |
| Fiji | both | 12.59(8.98,17.25) | 12.94(9.48,17.26) | 0.42(0.23,0.61) | 12.59(8.98,17.25) | 12.94(9.48,17.26) | 0.42(0.23,0.61) |
| Finland | both | 100.06(95.37,104.93) | 34.99(32.15,38.03) | -3.01(-3.24,-2.79) | 100.06(95.37,104.93) | 34.99(32.15,38.03) | -3.01(-3.24,-2.79) |
| France | both | 41.91(41.06,42.78) | 24.10(23.42,24.79) | -1.92(-2.14,-1.69) | 41.91(41.06,42.78) | 24.10(23.42,24.79) | -1.92(-2.14,-1.69) |
| Gabon | both | 35.00(29.24,41.65) | 27.48(23.82,31.57) | -0.93(-1.06,-0.81) | 35.00(29.24,41.65) | 27.48(23.82,31.57) | -0.93(-1.06,-0.81) |
| Gambia | both | 16.97(12.94,21.99) | 21.15(18.33,24.32) | 0.47(0.17,0.78) | 16.97(12.94,21.99) | 21.15(18.33,24.32) | 0.47(0.17,0.78) |
| Georgia | both | 37.23(34.69,39.91) | 23.68(20.92,26.74) | -1.18(-1.98,-0.37) | 37.23(34.69,39.91) | 23.68(20.92,26.74) | -1.18(-1.98,-0.37) |
| Germany | both | 66.14(65.18,67.11) | 28.32(27.65,29.00) | -3.16(-3.33,-2.99) | 66.14(65.18,67.11) | 28.32(27.65,29.00) | -3.16(-3.33,-2.99) |
| Ghana | both | 47.57(45.77,49.42) | 48.23(47.09,49.39) | 0.21(0.07,0.35) | 47.57(45.77,49.42) | 48.23(47.09,49.39) | 0.21(0.07,0.35) |
| Greece | both | 23.42(21.90,25.02) | 18.82(17.25,20.51) | -0.68(-0.80,-0.56) | 23.42(21.90,25.02) | 18.82(17.25,20.51) | -0.68(-0.80,-0.56) |
| Greenland | both | 46.85(23.65,86.42) | 16.46(3.74,48.40) | -3.64(-3.93,-3.35) | 46.85(23.65,86.42) | 16.46(3.74,48.40) | -3.64(-3.93,-3.35) |
| Grenada | both | 106.75(74.24,149.71) | 53.82(33.51,82.39) | -2.65(-3.05,-2.25) | 106.75(74.24,149.71) | 53.82(33.51,82.39) | -2.65(-3.05,-2.25) |
| Guam | both | 9.52(3.50,21.02) | 12.15(4.79,25.47) | 1.63(0.89,2.38) | 9.52(3.50,21.02) | 12.15(4.79,25.47) | 1.63(0.89,2.38) |
| Guatemala | both | 31.98(29.92,34.16) | 26.82(25.58,28.11) | -0.55(-0.78,-0.32) | 31.98(29.92,34.16) | 26.82(25.58,28.11) | -0.55(-0.78,-0.32) |
| Guinea | both | 18.04(16.23,20.00) | 19.35(18.13,20.62) | 0.21(0.15,0.28) | 18.04(16.23,20.00) | 19.35(18.13,20.62) | 0.21(0.15,0.28) |
| Guinea-Bissau | both | 57.92(50.34,66.43) | 46.54(41.98,51.49) | -0.67(-0.72,-0.62) | 57.92(50.34,66.43) | 46.54(41.98,51.49) | -0.67(-0.72,-0.62) |
| Guyana | both | 101.10(90.57,112.60) | 106.18(94.96,118.44) | -0.30(-0.86,0.27) | 101.10(90.57,112.60) | 106.18(94.96,118.44) | -0.30(-0.86,0.27) |
| Haiti | both | 138.94(134.30,143.71) | 96.50(93.92,99.14) | -1.02(-1.28,-0.75) | 138.94(134.30,143.71) | 96.50(93.92,99.14) | -1.02(-1.28,-0.75) |
| Honduras | both | 42.75(39.63,46.08) | 23.25(21.82,24.75) | -2.57(-2.93,-2.22) | 42.75(39.63,46.08) | 23.25(21.82,24.75) | -2.57(-2.93,-2.22) |
| Hungary | both | 138.74(134.96,142.61) | 56.42(53.62,59.34) | -3.83(-4.27,-3.38) | 138.74(134.96,142.61) | 56.42(53.62,59.34) | -3.83(-4.27,-3.38) |
| Iceland | both | 28.00(18.75,40.32) | 15.98(9.65,25.19) | -1.92(-2.03,-1.82) | 28.00(18.75,40.32) | 15.98(9.65,25.19) | -1.92(-2.03,-1.82) |
| India | both | 68.44(68.16,68.72) | 59.74(59.55,59.94) | -0.54(-0.88,-0.19) | 68.44(68.16,68.72) | 59.74(59.55,59.94) | -0.54(-0.88,-0.19) |
| Indonesia | both | 47.33(46.84,47.82) | 39.59(39.22,39.96) | -0.60(-0.74,-0.46) | 47.33(46.84,47.82) | 39.59(39.22,39.96) | -0.60(-0.74,-0.46) |
| Iran (Islamic Republic of) | both | 17.95(17.36,18.54) | 17.33(16.88,17.78) | -0.10(-0.47,0.27) | 17.95(17.36,18.54) | 17.33(16.88,17.78) | -0.10(-0.47,0.27) |
| Iraq | both | 16.13(15.18,17.14) | 10.36(9.89,10.86) | -1.34(-1.46,-1.21) | 16.13(15.18,17.14) | 10.36(9.89,10.86) | -1.34(-1.46,-1.21) |
| Ireland | both | 71.00(66.63,75.59) | 21.58(19.33,24.02) | -4.11(-4.36,-3.85) | 71.00(66.63,75.59) | 21.58(19.33,24.02) | -4.11(-4.36,-3.85) |
| Israel | both | 29.28(26.91,31.82) | 20.49(18.98,22.09) | -1.39(-1.69,-1.08) | 29.28(26.91,31.82) | 20.49(18.98,22.09) | -1.39(-1.69,-1.08) |
| Italy | both | 64.23(63.16,65.32) | 26.74(25.94,27.56) | -3.00(-3.11,-2.89) | 64.23(63.16,65.32) | 26.74(25.94,27.56) | -3.00(-3.11,-2.89) |
| Jamaica | both | 26.59(23.41,30.14) | 25.02(22.26,28.03) | -1.27(-1.99,-0.55) | 26.59(23.41,30.14) | 25.02(22.26,28.03) | -1.27(-1.99,-0.55) |
| Japan | both | 66.00(65.25,66.75) | 21.56(21.05,22.08) | -3.96(-4.17,-3.76) | 66.00(65.25,66.75) | 21.56(21.05,22.08) | -3.96(-4.17,-3.76) |
| Jordan | both | 12.22(10.39,14.31) | 10.57(9.71,11.49) | -0.28(-0.46,-0.10) | 12.22(10.39,14.31) | 10.57(9.71,11.49) | -0.28(-0.46,-0.10) |
| Kazakhstan | both | 31.01(29.70,32.36) | 17.63(16.66,18.64) | -1.33(-1.79,-0.86) | 31.01(29.70,32.36) | 17.63(16.66,18.64) | -1.33(-1.79,-0.86) |
| Kenya | both | 21.44(20.44,22.48) | 27.05(26.35,27.76) | 1.30(0.96,1.65) | 21.44(20.44,22.48) | 27.05(26.35,27.76) | 1.30(0.96,1.65) |
| Kiribati | both | 11.48(2.66,33.49) | 10.37(3.40,24.34) | -0.53(-0.75,-0.31) | 11.48(2.66,33.49) | 10.37(3.40,24.34) | -0.53(-0.75,-0.31) |
| Kuwait | both | 38.24(34.13,42.77) | 33.59(30.95,36.43) | -0.39(-1.72,0.96) | 38.24(34.13,42.77) | 33.59(30.95,36.43) | -0.39(-1.72,0.96) |
| Kyrgyzstan | both | 37.61(34.79,40.60) | 22.53(20.79,24.38) | -1.27(-1.89,-0.65) | 37.61(34.79,40.60) | 22.53(20.79,24.38) | -1.27(-1.89,-0.65) |
| Lao People's Democratic Republic | both | 59.04(55.21,63.08) | 52.66(50.17,55.23) | -0.56(-0.65,-0.48) | 59.04(55.21,63.08) | 52.66(50.17,55.23) | -0.56(-0.65,-0.48) |
| Latvia | both | 115.75(109.00,122.84) | 37.97(32.87,43.72) | -3.75(-4.12,-3.38) | 115.75(109.00,122.84) | 37.97(32.87,43.72) | -3.75(-4.12,-3.38) |
| Lebanon | both | 20.44(17.87,23.30) | 11.25(9.91,12.73) | -1.79(-2.08,-1.50) | 20.44(17.87,23.30) | 11.25(9.91,12.73) | -1.79(-2.08,-1.50) |
| Lesotho | both | 12.55(9.68,16.05) | 29.94(26.25,34.01) | 3.69(3.16,4.22) | 12.55(9.68,16.05) | 29.94(26.25,34.01) | 3.69(3.16,4.22) |
| Liberia | both | 20.28(17.44,23.47) | 18.90(17.11,20.83) | 0.14(-0.16,0.44) | 20.28(17.44,23.47) | 18.90(17.11,20.83) | 0.14(-0.16,0.44) |
| Libya | both | 13.87(12.05,15.92) | 9.61(8.54,10.78) | -0.85(-0.99,-0.71) | 13.87(12.05,15.92) | 9.61(8.54,10.78) | -0.85(-0.99,-0.71) |
| Lithuania | both | 118.50(112.84,124.38) | 44.48(39.99,49.40) | -2.90(-3.67,-2.14) | 118.50(112.84,124.38) | 44.48(39.99,49.40) | -2.90(-3.67,-2.14) |
| Luxembourg | both | 41.99(31.86,54.67) | 18.58(13.28,25.54) | -2.75(-2.93,-2.58) | 41.99(31.86,54.67) | 18.58(13.28,25.54) | -2.75(-2.93,-2.58) |
| Madagascar | both | 34.55(32.84,36.34) | 26.62(25.69,27.59) | -0.73(-0.89,-0.58) | 34.55(32.84,36.34) | 26.62(25.69,27.59) | -0.73(-0.89,-0.58) |
| Malawi | both | 33.42(31.56,35.37) | 36.76(35.42,38.13) | 0.25(0.00,0.50) | 33.42(31.56,35.37) | 36.76(35.42,38.13) | 0.25(0.00,0.50) |
| Malaysia | both | 57.64(55.92,59.40) | 54.62(53.40,55.87) | -0.75(-1.07,-0.43) | 57.64(55.92,59.40) | 54.62(53.40,55.87) | -0.75(-1.07,-0.43) |
| Maldives | both | 38.51(25.34,56.98) | 9.34(5.90,14.56) | -5.24(-5.79,-4.68) | 38.51(25.34,56.98) | 9.34(5.90,14.56) | -5.24(-5.79,-4.68) |
| Mali | both | 18.71(17.16,20.38) | 14.93(14.10,15.81) | -0.60(-0.72,-0.48) | 18.71(17.16,20.38) | 14.93(14.10,15.81) | -0.60(-0.72,-0.48) |
| Malta | both | 22.03(14.90,31.58) | 15.55(9.57,24.41) | -1.23(-1.39,-1.07) | 22.03(14.90,31.58) | 15.55(9.57,24.41) | -1.23(-1.39,-1.07) |
| Marshall Islands | both | 10.20(0.88,44.35) | 9.34(1.29,32.61) | -0.15(-0.29,-0.01) | 10.20(0.88,44.35) | 9.34(1.29,32.61) | -0.15(-0.29,-0.01) |
| Mauritania | both | 43.18(38.57,48.22) | 27.87(25.36,30.59) | -1.50(-1.63,-1.36) | 43.18(38.57,48.22) | 27.87(25.36,30.59) | -1.50(-1.63,-1.36) |
| Mauritius | both | 5.97(4.01,8.56) | 25.45(21.04,30.54) | 6.01(5.06,6.96) | 5.97(4.01,8.56) | 25.45(21.04,30.54) | 6.01(5.06,6.96) |
| Mexico | both | 61.83(60.99,62.68) | 40.48(39.94,41.04) | -0.99(-1.42,-0.56) | 61.83(60.99,62.68) | 40.48(39.94,41.04) | -0.99(-1.42,-0.56) |
| Micronesia (Federated States of) | both | 14.57(5.01,33.77) | 13.46(4.64,31.02) | -0.28(-0.36,-0.19) | 14.57(5.01,33.77) | 13.46(4.64,31.02) | -0.28(-0.36,-0.19) |
| Monaco | both | 30.08(5.28,106.79) | 23.17(3.15,83.89) | -0.89(-0.93,-0.85) | 30.08(5.28,106.79) | 23.17(3.15,83.89) | -0.89(-0.93,-0.85) |
| Mongolia | both | 10.16(8.06,12.69) | 12.00(10.18,14.07) | 0.95(0.81,1.09) | 10.16(8.06,12.69) | 12.00(10.18,14.07) | 0.95(0.81,1.09) |
| Montenegro | both | 53.36(44.72,63.20) | 34.69(27.11,43.83) | -1.49(-1.88,-1.10) | 53.36(44.72,63.20) | 34.69(27.11,43.83) | -1.49(-1.88,-1.10) |
| Morocco | both | 12.12(11.44,12.83) | 8.69(8.22,9.18) | -1.11(-1.21,-1.00) | 12.12(11.44,12.83) | 8.69(8.22,9.18) | -1.11(-1.21,-1.00) |
| Mozambique | both | 29.29(27.78,30.88) | 47.01(45.77,48.29) | 2.11(1.78,2.44) | 29.29(27.78,30.88) | 47.01(45.77,48.29) | 2.11(1.78,2.44) |
| Myanmar | both | 54.11(53.01,55.23) | 36.92(36.13,37.72) | -1.41(-1.59,-1.22) | 54.11(53.01,55.23) | 36.92(36.13,37.72) | -1.41(-1.59,-1.22) |
| Namibia | both | 16.18(12.87,20.16) | 23.97(21.05,27.19) | 1.11(0.61,1.62) | 16.18(12.87,20.16) | 23.97(21.05,27.19) | 1.11(0.61,1.62) |
| Nauru | both | 22.32(0.36,145.07) | 30.17(1.75,143.65) | 0.97(0.67,1.27) | 22.32(0.36,145.07) | 30.17(1.75,143.65) | 0.97(0.67,1.27) |
| Nepal | both | 34.11(32.76,35.51) | 23.61(22.79,24.46) | -1.17(-1.35,-0.99) | 34.11(32.76,35.51) | 23.61(22.79,24.46) | -1.17(-1.35,-0.99) |
| Netherlands | both | 128.58(125.67,131.53) | 42.72(40.97,44.54) | -3.92(-4.11,-3.72) | 128.58(125.67,131.53) | 42.72(40.97,44.54) | -3.92(-4.11,-3.72) |
| New Zealand | both | 69.95(65.61,74.51) | 24.43(22.19,26.85) | -4.21(-4.66,-3.76) | 69.95(65.61,74.51) | 24.43(22.19,26.85) | -4.21(-4.66,-3.76) |
| Nicaragua | both | 39.48(36.22,42.98) | 31.19(29.17,33.32) | -0.64(-0.83,-0.45) | 39.48(36.22,42.98) | 31.19(29.17,33.32) | -0.64(-0.83,-0.45) |
| Niger | both | 19.12(17.49,20.88) | 18.21(17.27,19.19) | -0.02(-0.12,0.07) | 19.12(17.49,20.88) | 18.21(17.27,19.19) | -0.02(-0.12,0.07) |
| Nigeria | both | 18.63(18.16,19.11) | 16.68(16.40,16.97) | -0.31(-0.47,-0.15) | 18.63(18.16,19.11) | 16.68(16.40,16.97) | -0.31(-0.47,-0.15) |
| Niue | both | 10.90(0.00,539.66) | 13.33(0.00,729.70) | 0.10(-0.13,0.32) | 10.90(0.00,539.66) | 13.33(0.00,729.70) | 0.10(-0.13,0.32) |
| North Macedonia | both | 33.35(29.46,37.62) | 20.65(17.54,24.21) | -1.80(-1.95,-1.64) | 33.35(29.46,37.62) | 20.65(17.54,24.21) | -1.80(-1.95,-1.64) |
| Northern Mariana Islands | both | 13.08(2.83,40.26) | 8.21(0.47,40.14) | -1.80(-2.23,-1.37) | 13.08(2.83,40.26) | 8.21(0.47,40.14) | -1.80(-2.23,-1.37) |
| Norway | both | 39.09(36.09,42.28) | 18.00(16.09,20.10) | -2.38(-2.72,-2.04) | 39.09(36.09,42.28) | 18.00(16.09,20.10) | -2.38(-2.72,-2.04) |
| Oman | both | 23.77(20.57,27.36) | 29.94(27.65,32.41) | 1.89(1.17,2.60) | 23.77(20.57,27.36) | 29.94(27.65,32.41) | 1.89(1.17,2.60) |
| Pakistan | both | 36.10(35.50,36.70) | 53.99(53.53,54.45) | 1.14(0.81,1.46) | 36.10(35.50,36.70) | 53.99(53.53,54.45) | 1.14(0.81,1.46) |
| Palau | both | 7.30(0.01,72.70) | 7.04(0.00,82.21) | -0.01(-0.04,0.03) | 7.30(0.01,72.70) | 7.04(0.00,82.21) | -0.01(-0.04,0.03) |
| Palestine | both | 8.71(6.60,11.36) | 5.95(4.94,7.11) | -1.36(-1.45,-1.26) | 8.71(6.60,11.36) | 5.95(4.94,7.11) | -1.36(-1.45,-1.26) |
| Panama | both | 76.12(70.83,81.73) | 47.56(44.29,51.02) | -1.81(-2.07,-1.55) | 76.12(70.83,81.73) | 47.56(44.29,51.02) | -1.81(-2.07,-1.55) |
| Papua New Guinea | both | 12.26(10.58,14.15) | 9.66(8.75,10.65) | -0.95(-1.07,-0.83) | 12.26(10.58,14.15) | 9.66(8.75,10.65) | -0.95(-1.07,-0.83) |
| Paraguay | both | 18.76(16.64,21.09) | 23.34(21.66,25.12) | 0.89(0.59,1.18) | 18.76(16.64,21.09) | 23.34(21.66,25.12) | 0.89(0.59,1.18) |
| Peru | both | 32.00(30.81,33.24) | 39.76(38.76,40.79) | 1.53(1.04,2.01) | 32.00(30.81,33.24) | 39.76(38.76,40.79) | 1.53(1.04,2.01) |
| Philippines | both | 30.97(30.28,31.68) | 29.86(29.36,30.36) | 0.11(-0.08,0.30) | 30.97(30.28,31.68) | 29.86(29.36,30.36) | 0.11(-0.08,0.30) |
| Poland | both | 95.33(93.71,96.97) | 61.26(59.83,62.73) | -2.40(-2.91,-1.90) | 95.33(93.71,96.97) | 61.26(59.83,62.73) | -2.40(-2.91,-1.90) |
| Portugal | both | 68.37(65.77,71.06) | 24.45(22.69,26.31) | -3.84(-4.02,-3.67) | 68.37(65.77,71.06) | 24.45(22.69,26.31) | -3.84(-4.02,-3.67) |
| Puerto Rico | both | 69.29(65.01,73.77) | 48.67(44.52,53.12) | -2.24(-2.85,-1.62) | 69.29(65.01,73.77) | 48.67(44.52,53.12) | -2.24(-2.85,-1.62) |
| Qatar | both | 38.64(30.66,48.45) | 26.49(23.45,29.96) | -0.42(-0.84,-0.01) | 38.64(30.66,48.45) | 26.49(23.45,29.96) | -0.42(-0.84,-0.01) |
| Republic of Korea | both | 123.95(122.44,125.47) | 22.49(21.75,23.26) | -7.00(-7.65,-6.34) | 123.95(122.44,125.47) | 22.49(21.75,23.26) | -7.00(-7.65,-6.34) |
| Republic of Moldova | both | 67.51(63.69,71.51) | 25.73(22.90,28.87) | -2.43(-2.83,-2.03) | 67.51(63.69,71.51) | 25.73(22.90,28.87) | -2.43(-2.83,-2.03) |
| Romania | both | 80.63(78.74,82.56) | 49.03(47.16,50.95) | -1.45(-1.58,-1.33) | 80.63(78.74,82.56) | 49.03(47.16,50.95) | -1.45(-1.58,-1.33) |
| Russian Federation | both | 44.49(43.95,45.04) | 25.58(25.12,26.05) | -1.56(-2.19,-0.93) | 44.49(43.95,45.04) | 25.58(25.12,26.05) | -1.56(-2.19,-0.93) |
| Rwanda | both | 192.09(186.97,197.32) | 74.66(72.44,76.94) | -4.18(-4.64,-3.71) | 192.09(186.97,197.32) | 74.66(72.44,76.94) | -4.18(-4.64,-3.71) |
| Saint Kitts and Nevis | both | 95.90(54.78,157.78) | 31.01(12.37,65.52) | -4.65(-5.22,-4.08) | 95.90(54.78,157.78) | 31.01(12.37,65.52) | -4.65(-5.22,-4.08) |
| Saint Lucia | both | 121.91(94.05,156.30) | 54.37(38.04,75.71) | -3.32(-3.69,-2.95) | 121.91(94.05,156.30) | 54.37(38.04,75.71) | -3.32(-3.69,-2.95) |
| Saint Vincent and the Grenadines | both | 76.47(52.75,108.56) | 42.70(25.16,67.97) | -2.75(-3.15,-2.34) | 76.47(52.75,108.56) | 42.70(25.16,67.97) | -2.75(-3.15,-2.34) |
| Samoa | both | 10.18(3.71,23.36) | 11.89(5.45,22.91) | 0.60(0.50,0.70) | 10.18(3.71,23.36) | 11.89(5.45,22.91) | 0.60(0.50,0.70) |
| San Marino | both | 66.87(25.10,145.27) | 40.41(10.04,109.80) | -1.38(-1.51,-1.25) | 66.87(25.10,145.27) | 40.41(10.04,109.80) | -1.38(-1.51,-1.25) |
| Sao Tome and Principe | both | 14.20(4.56,35.08) | 19.99(11.78,31.94) | 1.03(0.61,1.44) | 14.20(4.56,35.08) | 19.99(11.78,31.94) | 1.03(0.61,1.44) |
| Saudi Arabia | both | 13.23(12.34,14.16) | 9.25(8.83,9.70) | -0.95(-1.06,-0.83) | 13.23(12.34,14.16) | 9.25(8.83,9.70) | -0.95(-1.06,-0.83) |
| Senegal | both | 18.41(16.79,20.16) | 16.03(15.04,17.07) | -0.22(-0.39,-0.06) | 18.41(16.79,20.16) | 16.03(15.04,17.07) | -0.22(-0.39,-0.06) |
| Serbia | both | 100.84(97.58,104.19) | 38.93(36.74,41.24) | -3.18(-3.32,-3.04) | 100.84(97.58,104.19) | 38.93(36.74,41.24) | -3.18(-3.32,-3.04) |
| Seychelles | both | 26.73(11.43,54.85) | 28.07(14.10,50.97) | 0.68(0.13,1.24) | 26.73(11.43,54.85) | 28.07(14.10,50.97) | 0.68(0.13,1.24) |
| Sierra Leone | both | 24.75(22.33,27.38) | 22.59(21.05,24.23) | -0.08(-0.22,0.06) | 24.75(22.33,27.38) | 22.59(21.05,24.23) | -0.08(-0.22,0.06) |
| Singapore | both | 42.33(39.09,45.79) | 13.29(11.62,15.17) | -4.09(-4.25,-3.93) | 42.33(39.09,45.79) | 13.29(11.62,15.17) | -4.09(-4.25,-3.93) |
| Slovakia | both | 122.11(117.36,127.00) | 52.55(49.09,56.21) | -2.99(-3.28,-2.70) | 122.11(117.36,127.00) | 52.55(49.09,56.21) | -2.99(-3.28,-2.70) |
| Slovenia | both | 87.98(81.44,94.92) | 33.37(28.69,38.67) | -3.84(-4.34,-3.33) | 87.98(81.44,94.92) | 33.37(28.69,38.67) | -3.84(-4.34,-3.33) |
| Solomon Islands | both | 9.95(4.95,18.29) | 11.41(7.68,16.39) | 0.53(0.38,0.68) | 9.95(4.95,18.29) | 11.41(7.68,16.39) | 0.53(0.38,0.68) |
| Somalia | both | 39.25(37.00,41.60) | 47.88(46.36,49.43) | 1.02(0.82,1.22) | 39.25(37.00,41.60) | 47.88(46.36,49.43) | 1.02(0.82,1.22) |
| South Africa | both | 41.42(40.37,42.49) | 29.99(29.31,30.67) | -1.59(-2.12,-1.05) | 41.42(40.37,42.49) | 29.99(29.31,30.67) | -1.59(-2.12,-1.05) |
| South Sudan | both | 26.62(24.50,28.90) | 32.56(30.69,34.51) | 0.57(0.25,0.89) | 26.62(24.50,28.90) | 32.56(30.69,34.51) | 0.57(0.25,0.89) |
| Spain | both | 41.40(40.37,42.45) | 20.00(19.22,20.81) | -2.75(-3.02,-2.48) | 41.40(40.37,42.45) | 20.00(19.22,20.81) | -2.75(-3.02,-2.48) |
| Sri Lanka | both | 47.31(45.75,48.90) | 37.32(36.00,38.68) | -1.47(-1.96,-0.97) | 47.31(45.75,48.90) | 37.32(36.00,38.68) | -1.47(-1.96,-0.97) |
| Sudan | both | 11.63(10.84,12.46) | 9.55(9.09,10.02) | -0.52(-0.61,-0.42) | 11.63(10.84,12.46) | 9.55(9.09,10.02) | -0.52(-0.61,-0.42) |
| Suriname | both | 177.94(157.61,200.43) | 89.17(76.99,102.76) | -2.61(-2.97,-2.25) | 177.94(157.61,200.43) | 89.17(76.99,102.76) | -2.61(-2.97,-2.25) |
| Sweden | both | 60.33(57.53,63.23) | 16.13(14.77,17.58) | -4.04(-4.30,-3.79) | 60.33(57.53,63.23) | 16.13(14.77,17.58) | -4.04(-4.30,-3.79) |
| Switzerland | both | 86.07(82.44,89.83) | 24.27(22.43,26.23) | -3.96(-4.07,-3.85) | 86.07(82.44,89.83) | 24.27(22.43,26.23) | -3.96(-4.07,-3.85) |
| Syrian Arab Republic | both | 15.72(14.56,16.96) | 9.12(8.22,10.11) | -1.58(-1.75,-1.41) | 15.72(14.56,16.96) | 9.12(8.22,10.11) | -1.58(-1.75,-1.41) |
| Taiwan (Province of China) | both | 12.61(11.90,13.35) | 38.33(36.91,39.80) | 5.11(4.13,6.11) | 12.61(11.90,13.35) | 38.33(36.91,39.80) | 5.11(4.13,6.11) |
| Tajikistan | both | 12.12(10.62,13.80) | 7.29(6.49,8.16) | -1.84(-2.19,-1.48) | 12.12(10.62,13.80) | 7.29(6.49,8.16) | -1.84(-2.19,-1.48) |
| Thailand | both | 48.21(47.37,49.07) | 54.55(53.56,55.56) | -1.19(-1.94,-0.43) | 48.21(47.37,49.07) | 54.55(53.56,55.56) | -1.19(-1.94,-0.43) |
| Timor-Leste | both | 25.63(20.31,32.02) | 25.88(21.72,30.69) | -0.01(-0.48,0.46) | 25.63(20.31,32.02) | 25.88(21.72,30.69) | -0.01(-0.48,0.46) |
| Togo | both | 34.00(30.89,37.36) | 35.05(33.05,37.13) | 0.15(-0.03,0.34) | 34.00(30.89,37.36) | 35.05(33.05,37.13) | 0.15(-0.03,0.34) |
| Tokelau | both | 8.79(0.00,790.15) | 9.67(0.00,840.55) | -0.18(-0.39,0.03) | 8.79(0.00,790.15) | 9.67(0.00,840.55) | -0.18(-0.39,0.03) |
| Tonga | both | 6.94(0.95,26.47) | 7.07(1.23,23.07) | -0.04(-0.25,0.16) | 6.94(0.95,26.47) | 7.07(1.23,23.07) | -0.04(-0.25,0.16) |
| Trinidad and Tobago | both | 70.13(62.97,77.91) | 51.51(45.30,58.38) | -1.08(-1.34,-0.82) | 70.13(62.97,77.91) | 51.51(45.30,58.38) | -1.08(-1.34,-0.82) |
| Tunisia | both | 33.51(31.56,35.57) | 30.21(28.60,31.88) | -0.44(-0.52,-0.37) | 33.51(31.56,35.57) | 30.21(28.60,31.88) | -0.44(-0.52,-0.37) |
| Turkey | both | 21.97(21.37,22.59) | 20.24(19.74,20.74) | 0.35(-0.51,1.22) | 21.97(21.37,22.59) | 20.24(19.74,20.74) | 0.35(-0.51,1.22) |
| Turkmenistan | both | 22.17(19.82,24.74) | 12.77(11.29,14.41) | -1.52(-1.88,-1.15) | 22.17(19.82,24.74) | 12.77(11.29,14.41) | -1.52(-1.88,-1.15) |
| Tuvalu | both | 14.59(0.02,136.14) | 13.89(0.07,109.80) | -0.09(-0.20,0.03) | 14.59(0.02,136.14) | 13.89(0.07,109.80) | -0.09(-0.20,0.03) |
| Uganda | both | 45.53(43.86,47.26) | 81.46(80.09,82.85) | 1.11(0.55,1.68) | 45.53(43.86,47.26) | 81.46(80.09,82.85) | 1.11(0.55,1.68) |
| Ukraine | both | 41.21(40.30,42.13) | 25.98(25.13,26.86) | -1.32(-1.72,-0.91) | 41.21(40.30,42.13) | 25.98(25.13,26.86) | -1.32(-1.72,-0.91) |
| United Arab Emirates | both | 71.02(65.30,77.17) | 71.94(68.32,75.73) | 0.51(-0.19,1.22) | 71.02(65.30,77.17) | 71.94(68.32,75.73) | 0.51(-0.19,1.22) |
| United Kingdom | both | 43.04(42.15,43.95) | 20.17(19.57,20.78) | -2.99(-3.32,-2.65) | 43.04(42.15,43.95) | 20.17(19.57,20.78) | -2.99(-3.32,-2.65) |
| United Republic of Tanzania | both | 37.30(36.07,38.57) | 33.18(32.43,33.94) | -0.54(-0.69,-0.40) | 37.30(36.07,38.57) | 33.18(32.43,33.94) | -0.54(-0.69,-0.40) |
| United States of America | both | 55.87(55.40,56.34) | 29.10(28.79,29.42) | -2.18(-2.52,-1.85) | 55.87(55.40,56.34) | 29.10(28.79,29.42) | -2.18(-2.52,-1.85) |
| United States Virgin Islands | both | 80.60(55.03,114.28) | 81.28(48.27,129.10) | -0.15(-0.60,0.31) | 80.60(55.03,114.28) | 81.28(48.27,129.10) | -0.15(-0.60,0.31) |
| Uruguay | both | 36.69(33.25,40.40) | 32.91(29.75,36.33) | -0.45(-0.69,-0.20) | 36.69(33.25,40.40) | 32.91(29.75,36.33) | -0.45(-0.69,-0.20) |
| Uzbekistan | both | 21.36(20.37,22.39) | 32.89(31.94,33.85) | 2.20(1.45,2.96) | 21.36(20.37,22.39) | 32.89(31.94,33.85) | 2.20(1.45,2.96) |
| Vanuatu | both | 12.40(4.90,26.41) | 14.45(8.46,23.23) | 0.33(0.25,0.40) | 12.40(4.90,26.41) | 14.45(8.46,23.23) | 0.33(0.25,0.40) |
| Venezuela (Bolivarian Republic of) | both | 54.00(52.40,55.64) | 36.72(35.48,37.99) | -1.33(-1.58,-1.08) | 54.00(52.40,55.64) | 36.72(35.48,37.99) | -1.33(-1.58,-1.08) |
| Viet Nam | both | 41.73(40.97,42.51) | 57.92(57.15,58.70) | 1.60(1.30,1.89) | 41.73(40.97,42.51) | 57.92(57.15,58.70) | 1.60(1.30,1.89) |
| Yemen | both | 13.33(12.26,14.48) | 9.58(9.06,10.13) | -1.04(-1.11,-0.97) | 13.33(12.26,14.48) | 9.58(9.06,10.13) | -1.04(-1.11,-0.97) |
| Zambia | both | 74.74(71.63,77.98) | 84.82(82.80,86.88) | 0.02(-0.23,0.27) | 74.74(71.63,77.98) | 84.82(82.80,86.88) | 0.02(-0.23,0.27) |
| Zimbabwe | both | 8.15(7.23,9.16) | 36.15(34.66,37.69) | 6.50(4.79,8.24) | 8.15(7.23,9.16) | 36.15(34.66,37.69) | 6.50(4.79,8.24) |
| Afghanistan | female | 7.04(5.68,8.70) | 5.67(5.03,6.39) | -0.39(-0.64,-0.13) | 7.04(5.68,8.70) | 5.67(5.03,6.39) | -0.39(-0.64,-0.13) |
| Albania | female | 23.42(19.92,27.40) | 16.72(13.17,21.01) | -0.84(-1.15,-0.53) | 23.42(19.92,27.40) | 16.72(13.17,21.01) | -0.84(-1.15,-0.53) |
| Algeria | female | 10.25(9.33,11.24) | 6.95(6.40,7.53) | -1.26(-1.30,-1.21) | 10.25(9.33,11.24) | 6.95(6.40,7.53) | -1.26(-1.30,-1.21) |
| American Samoa | female | 6.18(0.02,58.58) | 5.59(0.00,59.53) | -0.11(-0.44,0.22) | 6.18(0.02,58.58) | 5.59(0.00,59.53) | -0.11(-0.44,0.22) |
| Andorra | female | 35.16(9.24,96.96) | 21.99(3.79,75.09) | -1.49(-1.60,-1.39) | 35.16(9.24,96.96) | 21.99(3.79,75.09) | -1.49(-1.60,-1.39) |
| Angola | female | 7.77(6.55,9.16) | 6.60(5.97,7.29) | -0.03(-0.33,0.27) | 7.77(6.55,9.16) | 6.60(5.97,7.29) | -0.03(-0.33,0.27) |
| Antigua and Barbuda | female | 23.26(4.92,69.32) | 9.58(0.82,41.69) | -3.33(-3.64,-3.01) | 23.26(4.92,69.32) | 9.58(0.82,41.69) | -3.33(-3.64,-3.01) |
| Argentina | female | 34.64(33.18,36.14) | 21.53(20.57,22.52) | -1.69(-1.84,-1.54) | 34.64(33.18,36.14) | 21.53(20.57,22.52) | -1.69(-1.84,-1.54) |
| Armenia | female | 8.00(6.08,10.37) | 7.65(5.48,10.51) | 0.98(0.43,1.52) | 8.00(6.08,10.37) | 7.65(5.48,10.51) | 0.98(0.43,1.52) |
| Australia | female | 17.22(15.84,18.69) | 7.59(6.79,8.47) | -2.54(-2.72,-2.37) | 17.22(15.84,18.69) | 7.59(6.79,8.47) | -2.54(-2.72,-2.37) |
| Austria | female | 40.44(37.17,43.92) | 15.93(13.83,18.28) | -3.12(-3.36,-2.88) | 40.44(37.17,43.92) | 15.93(13.83,18.28) | -3.12(-3.36,-2.88) |
| Azerbaijan | female | 6.67(5.45,8.11) | 4.48(3.62,5.51) | -1.32(-1.38,-1.27) | 6.67(5.45,8.11) | 4.48(3.62,5.51) | -1.32(-1.38,-1.27) |
| Bahamas | female | 30.09(17.75,48.26) | 15.73(8.25,27.31) | -2.33(-2.56,-2.09) | 30.09(17.75,48.26) | 15.73(8.25,27.31) | -2.33(-2.56,-2.09) |
| Bahrain | female | 9.47(4.39,18.26) | 5.00(2.61,8.78) | -2.32(-2.45,-2.19) | 9.47(4.39,18.26) | 5.00(2.61,8.78) | -2.32(-2.45,-2.19) |
| Bangladesh | female | 5.82(5.48,6.17) | 3.22(3.03,3.41) | -1.66(-1.93,-1.40) | 5.82(5.48,6.17) | 3.22(3.03,3.41) | -1.66(-1.93,-1.40) |
| Barbados | female | 15.92(7.19,30.74) | 11.98(4.36,26.78) | -1.19(-1.41,-0.97) | 15.92(7.19,30.74) | 11.98(4.36,26.78) | -1.19(-1.41,-0.97) |
| Belarus | female | 34.50(31.94,37.22) | 17.96(15.82,20.34) | -2.51(-3.31,-1.71) | 34.50(31.94,37.22) | 17.96(15.82,20.34) | -2.51(-3.31,-1.71) |
| Belgium | female | 39.69(36.79,42.76) | 17.95(16.00,20.09) | -2.95(-3.26,-2.64) | 39.69(36.79,42.76) | 17.95(16.00,20.09) | -2.95(-3.26,-2.64) |
| Belize | female | 57.41(35.15,90.55) | 20.79(12.65,32.39) | -3.79(-4.13,-3.46) | 57.41(35.15,90.55) | 20.79(12.65,32.39) | -3.79(-4.13,-3.46) |
| Benin | female | 8.92(7.06,11.15) | 7.12(6.12,8.24) | -1.06(-1.24,-0.89) | 8.92(7.06,11.15) | 7.12(6.12,8.24) | -1.06(-1.24,-0.89) |
| Bermuda | female | 21.71(3.97,74.48) | 5.94(0.01,63.75) | -4.80(-5.14,-4.45) | 21.71(3.97,74.48) | 5.94(0.01,63.75) | -4.80(-5.14,-4.45) |
| Bhutan | female | 15.20(9.04,24.53) | 6.18(2.98,11.39) | -3.72(-3.98,-3.45) | 15.20(9.04,24.53) | 6.18(2.98,11.39) | -3.72(-3.98,-3.45) |
| Bolivia (Plurinational State of) | female | 14.49(12.42,16.82) | 7.70(6.63,8.89) | -2.05(-2.19,-1.91) | 14.49(12.42,16.82) | 7.70(6.63,8.89) | -2.05(-2.19,-1.91) |
| Bosnia and Herzegovina | female | 19.02(16.30,22.07) | 14.19(11.06,18.01) | -1.04(-1.14,-0.95) | 19.02(16.30,22.07) | 14.19(11.06,18.01) | -1.04(-1.14,-0.95) |
| Botswana | female | 5.84(3.25,9.87) | 6.52(4.54,9.08) | 1.07(0.69,1.46) | 5.84(3.25,9.87) | 6.52(4.54,9.08) | 1.07(0.69,1.46) |
| Brazil | female | 19.82(19.33,20.32) | 18.98(18.57,19.40) | -0.40(-1.01,0.23) | 19.82(19.33,20.32) | 18.98(18.57,19.40) | -0.40(-1.01,0.23) |
| Brunei Darussalam | female | 40.71(25.77,61.49) | 20.10(12.06,31.92) | -2.04(-2.17,-1.92) | 40.71(25.77,61.49) | 20.10(12.06,31.92) | -2.04(-2.17,-1.92) |
| Bulgaria | female | 29.43(26.74,32.33) | 20.89(18.01,24.13) | -1.30(-1.62,-0.98) | 29.43(26.74,32.33) | 20.89(18.01,24.13) | -1.30(-1.62,-0.98) |
| Burkina Faso | female | 11.45(9.87,13.23) | 10.53(9.60,11.54) | 0.14(-0.07,0.36) | 11.45(9.87,13.23) | 10.53(9.60,11.54) | 0.14(-0.07,0.36) |
| Burundi | female | 40.01(36.29,44.05) | 23.48(21.67,25.41) | -1.90(-2.10,-1.69) | 40.01(36.29,44.05) | 23.48(21.67,25.41) | -1.90(-2.10,-1.69) |
| Cabo Verde | female | 7.76(2.49,19.66) | 4.63(1.62,10.48) | -1.82(-2.13,-1.51) | 7.76(2.49,19.66) | 4.63(1.62,10.48) | -1.82(-2.13,-1.51) |
| Cambodia | female | 22.23(20.22,24.39) | 27.03(25.36,28.79) | 0.00(-0.26,0.26) | 22.23(20.22,24.39) | 27.03(25.36,28.79) | 0.00(-0.26,0.26) |
| Cameroon | female | 10.99(9.54,12.63) | 10.94(10.14,11.79) | 0.07(-0.04,0.19) | 10.99(9.54,12.63) | 10.94(10.14,11.79) | 0.07(-0.04,0.19) |
| Canada | female | 23.03(21.75,24.36) | 15.08(14.09,16.13) | -1.37(-1.53,-1.20) | 23.03(21.75,24.36) | 15.08(14.09,16.13) | -1.37(-1.53,-1.20) |
| Central African Republic | female | 6.45(4.43,9.17) | 4.97(3.73,6.50) | -0.68(-0.80,-0.57) | 6.45(4.43,9.17) | 4.97(3.73,6.50) | -0.68(-0.80,-0.57) |
| Chad | female | 6.29(4.86,8.02) | 7.44(6.50,8.49) | 0.72(0.57,0.87) | 6.29(4.86,8.02) | 7.44(6.50,8.49) | 0.72(0.57,0.87) |
| Chile | female | 16.02(14.59,17.56) | 16.82(15.48,18.25) | -0.50(-1.01,0.00) | 16.02(14.59,17.56) | 16.82(15.48,18.25) | -0.50(-1.01,0.00) |
| China | female | 55.24(54.96,55.53) | 49.14(48.85,49.44) | -0.77(-1.28,-0.25) | 55.24(54.96,55.53) | 49.14(48.85,49.44) | -0.77(-1.28,-0.25) |
| Colombia | female | 11.65(10.87,12.47) | 10.70(10.07,11.37) | -0.48(-0.73,-0.22) | 11.65(10.87,12.47) | 10.70(10.07,11.37) | -0.48(-0.73,-0.22) |
| Comoros | female | 16.92(9.28,29.00) | 16.31(10.57,24.19) | -0.85(-1.47,-0.23) | 16.92(9.28,29.00) | 16.31(10.57,24.19) | -0.85(-1.47,-0.23) |
| Congo | female | 5.75(3.73,8.57) | 5.41(4.13,6.96) | -0.05(-0.15,0.04) | 5.75(3.73,8.57) | 5.41(4.13,6.96) | -0.05(-0.15,0.04) |
| Cook Islands | female | 9.23(0.00,136.51) | 12.11(0.00,149.50) | 1.24(0.97,1.51) | 9.23(0.00,136.51) | 12.11(0.00,149.50) | 1.24(0.97,1.51) |
| Costa Rica | female | 12.42(9.83,15.51) | 11.16(9.17,13.49) | -0.67(-0.84,-0.50) | 12.42(9.83,15.51) | 11.16(9.17,13.49) | -0.67(-0.84,-0.50) |
| Croatia | female | 32.22(28.59,36.19) | 18.62(15.34,22.47) | -2.16(-2.58,-1.73) | 32.22(28.59,36.19) | 18.62(15.34,22.47) | -2.16(-2.58,-1.73) |
| Cuba | female | 120.89(116.53,125.37) | 25.20(22.85,27.74) | -6.21(-6.80,-5.61) | 120.89(116.53,125.37) | 25.20(22.85,27.74) | -6.21(-6.80,-5.61) |
| Cyprus | female | 19.11(12.75,27.64) | 11.05(7.27,16.51) | -1.83(-2.29,-1.37) | 19.11(12.75,27.64) | 11.05(7.27,16.51) | -1.83(-2.29,-1.37) |
| Czechia | female | 37.09(34.34,40.00) | 20.82(18.49,23.39) | -2.57(-3.08,-2.05) | 37.09(34.34,40.00) | 20.82(18.49,23.39) | -2.57(-3.08,-2.05) |
| Côte d'Ivoire | female | 7.49(6.38,8.76) | 8.58(7.82,9.40) | 0.67(0.58,0.77) | 7.49(6.38,8.76) | 8.58(7.82,9.40) | 0.67(0.58,0.77) |
| Democratic People's Republic of Korea | female | 35.88(34.10,37.74) | 44.58(42.71,46.51) | 0.62(0.45,0.79) | 35.88(34.10,37.74) | 44.58(42.71,46.51) | 0.62(0.45,0.79) |
| Democratic Republic of the Congo | female | 7.08(6.46,7.75) | 5.39(5.04,5.75) | -1.06(-1.13,-0.99) | 7.08(6.46,7.75) | 5.39(5.04,5.75) | -1.06(-1.13,-0.99) |
| Denmark | female | 87.25(81.29,93.55) | 23.61(20.51,27.08) | -4.60(-4.81,-4.38) | 87.25(81.29,93.55) | 23.61(20.51,27.08) | -4.60(-4.81,-4.38) |
| Djibouti | female | 8.48(3.22,19.08) | 7.30(4.35,11.54) | -0.58(-0.82,-0.33) | 8.48(3.22,19.08) | 7.30(4.35,11.54) | -0.58(-0.82,-0.33) |
| Dominica | female | 21.12(4.09,68.41) | 17.96(2.62,61.37) | -0.85(-0.97,-0.74) | 21.12(4.09,68.41) | 17.96(2.62,61.37) | -0.85(-0.97,-0.74) |
| Dominican Republic | female | 16.44(14.45,18.64) | 12.33(10.92,13.87) | -0.88(-1.03,-0.73) | 16.44(14.45,18.64) | 12.33(10.92,13.87) | -0.88(-1.03,-0.73) |
| Ecuador | female | 13.32(11.77,15.04) | 17.00(15.69,18.40) | 1.56(0.89,2.23) | 13.32(11.77,15.04) | 17.00(15.69,18.40) | 1.56(0.89,2.23) |
| Egypt | female | 11.33(10.69,12.00) | 6.99(6.63,7.36) | -1.41(-1.56,-1.25) | 11.33(10.69,12.00) | 6.99(6.63,7.36) | -1.41(-1.56,-1.25) |
| El Salvador | female | 15.94(13.61,18.58) | 12.43(10.62,14.47) | -1.10(-1.38,-0.82) | 15.94(13.61,18.58) | 12.43(10.62,14.47) | -1.10(-1.38,-0.82) |
| Equatorial Guinea | female | 5.60(1.64,14.25) | 5.53(3.18,9.04) | 0.06(-0.21,0.34) | 5.60(1.64,14.25) | 5.53(3.18,9.04) | 0.06(-0.21,0.34) |
| Eritrea | female | 10.71(8.29,13.67) | 11.16(9.43,13.13) | 0.18(0.09,0.26) | 10.71(8.29,13.67) | 11.16(9.43,13.13) | 0.18(0.09,0.26) |
| Estonia | female | 34.37(27.82,42.05) | 12.93(8.22,19.56) | -3.08(-3.57,-2.59) | 34.37(27.82,42.05) | 12.93(8.22,19.56) | -3.08(-3.57,-2.59) |
| Eswatini | female | 7.55(3.79,13.85) | 7.39(4.45,11.63) | 0.20(-0.02,0.42) | 7.55(3.79,13.85) | 7.39(4.45,11.63) | 0.20(-0.02,0.42) |
| Ethiopia | female | 17.92(17.06,18.80) | 9.35(8.95,9.76) | -2.71(-2.94,-2.48) | 17.92(17.06,18.80) | 9.35(8.95,9.76) | -2.71(-2.94,-2.48) |
| Fiji | female | 8.89(4.84,15.14) | 9.47(5.47,15.28) | 0.50(0.33,0.67) | 8.89(4.84,15.14) | 9.47(5.47,15.28) | 0.50(0.33,0.67) |
| Finland | female | 60.86(55.65,66.47) | 22.19(18.99,25.80) | -2.92(-3.19,-2.65) | 60.86(55.65,66.47) | 22.19(18.99,25.80) | -2.92(-3.19,-2.65) |
| France | female | 23.81(22.91,24.75) | 14.32(13.58,15.08) | -1.86(-2.15,-1.58) | 23.81(22.91,24.75) | 14.32(13.58,15.08) | -1.86(-2.15,-1.58) |
| Gabon | female | 6.94(3.62,12.35) | 4.73(2.81,7.53) | -1.18(-1.27,-1.10) | 6.94(3.62,12.35) | 4.73(2.81,7.53) | -1.18(-1.27,-1.10) |
| Gambia | female | 5.41(2.56,10.42) | 5.59(3.70,8.17) | -0.08(-0.28,0.12) | 5.41(2.56,10.42) | 5.59(3.70,8.17) | -0.08(-0.28,0.12) |
| Georgia | female | 15.02(12.82,17.51) | 8.01(5.82,10.83) | -1.99(-2.26,-1.73) | 15.02(12.82,17.51) | 8.01(5.82,10.83) | -1.99(-2.26,-1.73) |
| Germany | female | 41.73(40.63,42.85) | 17.82(17.05,18.63) | -3.16(-3.33,-2.98) | 41.73(40.63,42.85) | 17.82(17.05,18.63) | -3.16(-3.33,-2.98) |
| Ghana | female | 29.35(27.40,31.42) | 17.44(16.49,18.42) | -1.86(-2.22,-1.49) | 29.35(27.40,31.42) | 17.44(16.49,18.42) | -1.86(-2.22,-1.49) |
| Greece | female | 13.24(11.65,15.00) | 10.75(9.10,12.63) | -0.73(-0.81,-0.64) | 13.24(11.65,15.00) | 10.75(9.10,12.63) | -0.73(-0.81,-0.64) |
| Greenland | female | 17.71(2.07,74.25) | 7.30(0.05,57.39) | -3.59(-3.90,-3.28) | 17.71(2.07,74.25) | 7.30(0.05,57.39) | -3.59(-3.90,-3.28) |
| Grenada | female | 37.10(13.59,83.69) | 18.02(4.26,50.68) | -2.92(-3.19,-2.65) | 37.10(13.59,83.69) | 18.02(4.26,50.68) | -2.92(-3.19,-2.65) |
| Guam | female | 7.45(0.99,26.64) | 5.30(0.33,24.04) | -1.02(-1.27,-0.76) | 7.45(0.99,26.64) | 5.30(0.33,24.04) | -1.02(-1.27,-0.76) |
| Guatemala | female | 12.21(10.48,14.16) | 9.00(8.03,10.07) | -0.93(-1.16,-0.70) | 12.21(10.48,14.16) | 9.00(8.03,10.07) | -0.93(-1.16,-0.70) |
| Guinea | female | 8.85(7.17,10.82) | 7.93(6.90,9.08) | -0.30(-0.34,-0.26) | 8.85(7.17,10.82) | 7.93(6.90,9.08) | -0.30(-0.34,-0.26) |
| Guinea-Bissau | female | 20.52(14.56,28.30) | 13.71(10.43,17.75) | -1.19(-1.48,-0.90) | 20.52(14.56,28.30) | 13.71(10.43,17.75) | -1.19(-1.48,-0.90) |
| Guyana | female | 43.22(33.91,54.52) | 24.50(17.35,33.76) | -2.49(-2.84,-2.14) | 43.22(33.91,54.52) | 24.50(17.35,33.76) | -2.49(-2.84,-2.14) |
| Haiti | female | 69.81(65.32,74.53) | 52.37(49.73,55.11) | -0.80(-0.97,-0.63) | 69.81(65.32,74.53) | 52.37(49.73,55.11) | -0.80(-0.97,-0.63) |
| Honduras | female | 17.21(14.53,20.28) | 12.45(11.03,14.01) | -1.48(-1.86,-1.10) | 17.21(14.53,20.28) | 12.45(11.03,14.01) | -1.48(-1.86,-1.10) |
| Hungary | female | 53.41(50.10,56.90) | 30.85(27.92,34.02) | -2.71(-3.08,-2.34) | 53.41(50.10,56.90) | 30.85(27.92,34.02) | -2.71(-3.08,-2.34) |
| Iceland | female | 12.95(5.03,27.55) | 8.94(3.00,21.20) | -1.24(-1.36,-1.13) | 12.95(5.03,27.55) | 8.94(3.00,21.20) | -1.24(-1.36,-1.13) |
| India | female | 18.88(18.67,19.09) | 11.69(11.57,11.81) | -2.32(-2.65,-1.98) | 18.88(18.67,19.09) | 11.69(11.57,11.81) | -2.32(-2.65,-1.98) |
| Indonesia | female | 27.73(27.21,28.26) | 16.29(15.96,16.63) | -1.85(-2.08,-1.61) | 27.73(27.21,28.26) | 16.29(15.96,16.63) | -1.85(-2.08,-1.61) |
| Iran (Islamic Republic of) | female | 11.29(10.63,11.98) | 7.63(7.22,8.06) | -1.31(-1.36,-1.26) | 11.29(10.63,11.98) | 7.63(7.22,8.06) | -1.31(-1.36,-1.26) |
| Iraq | female | 8.36(7.37,9.46) | 5.04(4.56,5.56) | -1.68(-1.85,-1.51) | 8.36(7.37,9.46) | 5.04(4.56,5.56) | -1.68(-1.85,-1.51) |
| Ireland | female | 35.93(31.58,40.72) | 12.72(10.34,15.51) | -3.38(-3.59,-3.16) | 35.93(31.58,40.72) | 12.72(10.34,15.51) | -3.38(-3.59,-3.16) |
| Israel | female | 13.38(11.16,15.92) | 8.51(7.16,10.05) | -1.52(-1.63,-1.41) | 13.38(11.16,15.92) | 8.51(7.16,10.05) | -1.52(-1.63,-1.41) |
| Italy | female | 33.04(31.95,34.16) | 9.91(9.22,10.64) | -4.34(-4.61,-4.07) | 33.04(31.95,34.16) | 9.91(9.22,10.64) | -4.34(-4.61,-4.07) |
| Jamaica | female | 11.37(8.56,14.90) | 9.66(7.34,12.53) | -0.79(-1.09,-0.49) | 11.37(8.56,14.90) | 9.66(7.34,12.53) | -0.79(-1.09,-0.49) |
| Japan | female | 46.13(45.24,47.03) | 16.46(15.82,17.12) | -4.08(-4.42,-3.73) | 46.13(45.24,47.03) | 16.46(15.82,17.12) | -4.08(-4.42,-3.73) |
| Jordan | female | 7.22(5.23,9.81) | 4.46(3.65,5.41) | -1.89(-2.06,-1.73) | 7.22(5.23,9.81) | 4.46(3.65,5.41) | -1.89(-2.06,-1.73) |
| Kazakhstan | female | 15.05(13.77,16.42) | 9.52(8.53,10.60) | -1.37(-1.77,-0.97) | 15.05(13.77,16.42) | 9.52(8.53,10.60) | -1.37(-1.77,-0.97) |
| Kenya | female | 10.95(9.94,12.04) | 9.12(8.55,9.71) | -0.14(-0.39,0.12) | 10.95(9.94,12.04) | 9.12(8.55,9.71) | -0.14(-0.39,0.12) |
| Kiribati | female | 5.25(0.05,38.74) | 4.40(0.15,23.55) | -0.57(-0.82,-0.31) | 5.25(0.05,38.74) | 4.40(0.15,23.55) | -0.57(-0.82,-0.31) |
| Kuwait | female | 13.19(9.68,17.63) | 6.53(5.01,8.47) | -2.82(-3.48,-2.16) | 13.19(9.68,17.63) | 6.53(5.01,8.47) | -2.82(-3.48,-2.16) |
| Kyrgyzstan | female | 17.01(14.40,19.99) | 9.67(8.09,11.47) | -1.56(-2.15,-0.97) | 17.01(14.40,19.99) | 9.67(8.09,11.47) | -1.56(-2.15,-0.97) |
| Lao People's Democratic Republic | female | 29.91(26.16,34.08) | 26.06(23.60,28.70) | -0.62(-0.86,-0.37) | 29.91(26.16,34.08) | 26.06(23.60,28.70) | -0.62(-0.86,-0.37) |
| Latvia | female | 36.56(31.30,42.49) | 17.19(12.46,23.30) | -2.58(-3.08,-2.07) | 36.56(31.30,42.49) | 17.19(12.46,23.30) | -2.58(-3.08,-2.07) |
| Lebanon | female | 7.41(5.36,10.02) | 4.00(2.92,5.39) | -2.22(-2.34,-2.09) | 7.41(5.36,10.02) | 4.00(2.92,5.39) | -2.22(-2.34,-2.09) |
| Lesotho | female | 5.76(3.33,9.33) | 6.77(4.47,9.90) | 1.10(0.83,1.36) | 5.76(3.33,9.33) | 6.77(4.47,9.90) | 1.10(0.83,1.36) |
| Liberia | female | 8.18(5.78,11.31) | 7.02(5.54,8.78) | -0.84(-1.10,-0.59) | 8.18(5.78,11.31) | 7.02(5.54,8.78) | -0.84(-1.10,-0.59) |
| Libya | female | 10.25(7.95,13.07) | 5.34(4.23,6.66) | -1.96(-2.03,-1.89) | 10.25(7.95,13.07) | 5.34(4.23,6.66) | -1.96(-2.03,-1.89) |
| Lithuania | female | 35.77(31.44,40.55) | 15.72(12.03,20.31) | -2.09(-2.86,-1.31) | 35.77(31.44,40.55) | 15.72(12.03,20.31) | -2.09(-2.86,-1.31) |
| Luxembourg | female | 18.71(10.00,32.96) | 10.37(5.19,19.25) | -2.22(-2.33,-2.10) | 18.71(10.00,32.96) | 10.37(5.19,19.25) | -2.22(-2.33,-2.10) |
| Madagascar | female | 14.59(13.02,16.31) | 11.82(10.95,12.75) | -0.68(-0.76,-0.61) | 14.59(13.02,16.31) | 11.82(10.95,12.75) | -0.68(-0.76,-0.61) |
| Malawi | female | 13.69(12.04,15.54) | 12.41(11.35,13.56) | -0.29(-0.36,-0.22) | 13.69(12.04,15.54) | 12.41(11.35,13.56) | -0.29(-0.36,-0.22) |
| Malaysia | female | 24.42(22.84,26.08) | 17.49(16.50,18.53) | -1.13(-1.30,-0.95) | 24.42(22.84,26.08) | 17.49(16.50,18.53) | -1.13(-1.30,-0.95) |
| Maldives | female | 25.44(11.67,50.80) | 4.30(1.11,12.24) | -6.60(-7.12,-6.09) | 25.44(11.67,50.80) | 4.30(1.11,12.24) | -6.60(-7.12,-6.09) |
| Mali | female | 8.86(7.43,10.50) | 5.82(5.12,6.60) | -1.49(-1.67,-1.30) | 8.86(7.43,10.50) | 5.82(5.12,6.60) | -1.49(-1.67,-1.30) |
| Malta | female | 11.11(4.67,22.83) | 8.35(2.86,20.39) | -0.88(-0.99,-0.78) | 11.11(4.67,22.83) | 8.35(2.86,20.39) | -0.88(-0.99,-0.78) |
| Marshall Islands | female | 7.15(0.01,69.50) | 6.69(0.06,47.00) | -0.41(-0.67,-0.15) | 7.15(0.01,69.50) | 6.69(0.06,47.00) | -0.41(-0.67,-0.15) |
| Mauritania | female | 14.22(10.70,18.61) | 9.53(7.56,11.89) | -1.20(-1.47,-0.94) | 14.22(10.70,18.61) | 9.53(7.56,11.89) | -1.20(-1.47,-0.94) |
| Mauritius | female | 4.02(1.91,7.50) | 6.87(3.89,11.31) | 2.12(1.68,2.56) | 4.02(1.91,7.50) | 6.87(3.89,11.31) | 2.12(1.68,2.56) |
| Mexico | female | 15.00(14.43,15.59) | 11.24(10.84,11.65) | -0.33(-0.84,0.17) | 15.00(14.43,15.59) | 11.24(10.84,11.65) | -0.33(-0.84,0.17) |
| Micronesia (Federated States of) | female | 13.33(2.17,45.75) | 9.96(1.16,38.11) | -1.07(-1.17,-0.97) | 13.33(2.17,45.75) | 9.96(1.16,38.11) | -1.07(-1.17,-0.97) |
| Monaco | female | 16.51(0.11,151.48) | 11.74(0.02,114.05) | -1.07(-1.18,-0.97) | 16.51(0.11,151.48) | 11.74(0.02,114.05) | -1.07(-1.18,-0.97) |
| Mongolia | female | 8.00(5.46,11.47) | 6.86(4.99,9.27) | -0.26(-0.37,-0.14) | 8.00(5.46,11.47) | 6.86(4.99,9.27) | -0.26(-0.37,-0.14) |
| Montenegro | female | 30.09(21.13,41.59) | 20.71(12.78,32.00) | -1.30(-1.70,-0.90) | 30.09(21.13,41.59) | 20.71(12.78,32.00) | -1.30(-1.70,-0.90) |
| Morocco | female | 8.17(7.39,9.02) | 5.46(4.94,6.02) | -1.36(-1.51,-1.22) | 8.17(7.39,9.02) | 5.46(4.94,6.02) | -1.36(-1.51,-1.22) |
| Mozambique | female | 11.38(10.10,12.79) | 10.00(9.22,10.84) | -0.24(-0.31,-0.16) | 11.38(10.10,12.79) | 10.00(9.22,10.84) | -0.24(-0.31,-0.16) |
| Myanmar | female | 34.52(33.29,35.80) | 15.60(14.89,16.35) | -2.95(-3.39,-2.50) | 34.52(33.29,35.80) | 15.60(14.89,16.35) | -2.95(-3.39,-2.50) |
| Namibia | female | 6.88(4.06,11.08) | 6.43(4.44,9.04) | -0.03(-0.13,0.06) | 6.88(4.06,11.08) | 6.43(4.44,9.04) | -0.03(-0.13,0.06) |
| Nauru | female | 14.37(0.00,238.60) | 17.72(0.00,213.95) | 0.66(0.35,0.98) | 14.37(0.00,238.60) | 17.72(0.00,213.95) | 0.66(0.35,0.98) |
| Nepal | female | 10.44(9.43,11.55) | 5.47(4.94,6.04) | -2.27(-2.34,-2.21) | 10.44(9.43,11.55) | 5.47(4.94,6.04) | -2.27(-2.34,-2.21) |
| Netherlands | female | 100.23(96.57,104.01) | 28.39(26.36,30.54) | -4.37(-4.66,-4.07) | 100.23(96.57,104.01) | 28.39(26.36,30.54) | -4.37(-4.66,-4.07) |
| New Zealand | female | 32.49(28.38,37.03) | 9.98(7.99,12.36) | -4.63(-5.19,-4.07) | 32.49(28.38,37.03) | 9.98(7.99,12.36) | -4.63(-5.19,-4.07) |
| Nicaragua | female | 13.42(10.90,16.41) | 7.69(6.31,9.27) | -1.79(-1.88,-1.70) | 13.42(10.90,16.41) | 7.69(6.31,9.27) | -1.79(-1.88,-1.70) |
| Niger | female | 8.38(6.91,10.10) | 6.82(6.03,7.69) | -0.78(-0.89,-0.68) | 8.38(6.91,10.10) | 6.82(6.03,7.69) | -0.78(-0.89,-0.68) |
| Nigeria | female | 7.91(7.48,8.37) | 5.93(5.70,6.16) | -0.92(-1.09,-0.76) | 7.91(7.48,8.37) | 5.93(5.70,6.16) | -0.92(-1.09,-0.76) |
| Niue | female | 7.31(0.00,1056.97) | 9.38(0.00,1405.02) | -0.16(-0.46,0.15) | 7.31(0.00,1056.97) | 9.38(0.00,1405.02) | -0.16(-0.46,0.15) |
| North Macedonia | female | 24.20(19.56,29.61) | 13.46(9.97,17.92) | -2.12(-2.23,-2.01) | 24.20(19.56,29.61) | 13.46(9.97,17.92) | -2.12(-2.23,-2.01) |
| Northern Mariana Islands | female | 18.65(2.64,67.62) | 6.28(0.01,66.28) | -4.31(-4.74,-3.87) | 18.65(2.64,67.62) | 6.28(0.01,66.28) | -4.31(-4.74,-3.87) |
| Norway | female | 18.95(16.03,22.27) | 10.91(8.81,13.39) | -2.34(-2.83,-1.86) | 18.95(16.03,22.27) | 10.91(8.81,13.39) | -2.34(-2.83,-1.86) |
| Oman | female | 11.54(7.87,16.42) | 8.17(6.32,10.46) | -0.95(-1.07,-0.82) | 11.54(7.87,16.42) | 8.17(6.32,10.46) | -0.95(-1.07,-0.82) |
| Pakistan | female | 12.09(11.59,12.60) | 13.97(13.65,14.31) | 0.17(-0.13,0.46) | 12.09(11.59,12.60) | 13.97(13.65,14.31) | 0.17(-0.13,0.46) |
| Palau | female | 6.75(0.00,134.58) | 6.66(0.00,192.15) | 0.11(0.03,0.19) | 6.75(0.00,134.58) | 6.66(0.00,192.15) | 0.11(0.03,0.19) |
| Palestine | female | 5.38(3.15,8.75) | 3.34(2.30,4.70) | -1.67(-1.74,-1.59) | 5.38(3.15,8.75) | 3.34(2.30,4.70) | -1.67(-1.74,-1.59) |
| Panama | female | 12.32(9.42,15.89) | 8.64(6.73,10.92) | -1.29(-1.42,-1.17) | 12.32(9.42,15.89) | 8.64(6.73,10.92) | -1.29(-1.42,-1.17) |
| Papua New Guinea | female | 10.30(8.13,12.91) | 7.87(6.70,9.18) | -1.05(-1.16,-0.93) | 10.30(8.13,12.91) | 7.87(6.70,9.18) | -1.05(-1.16,-0.93) |
| Paraguay | female | 12.07(9.72,14.86) | 10.95(9.34,12.76) | -0.34(-0.47,-0.21) | 12.07(9.72,14.86) | 10.95(9.34,12.76) | -0.34(-0.47,-0.21) |
| Peru | female | 12.54(11.50,13.66) | 11.98(11.21,12.80) | 0.11(-0.15,0.37) | 12.54(11.50,13.66) | 11.98(11.21,12.80) | 0.11(-0.15,0.37) |
| Philippines | female | 16.89(16.16,17.63) | 10.91(10.49,11.35) | -1.45(-1.64,-1.27) | 16.89(16.16,17.63) | 10.91(10.49,11.35) | -1.45(-1.64,-1.27) |
| Poland | female | 37.86(36.43,39.33) | 30.24(28.78,31.75) | -1.42(-1.87,-0.97) | 37.86(36.43,39.33) | 30.24(28.78,31.75) | -1.42(-1.87,-0.97) |
| Portugal | female | 30.01(27.59,32.58) | 12.15(10.43,14.09) | -3.13(-3.39,-2.87) | 30.01(27.59,32.58) | 12.15(10.43,14.09) | -3.13(-3.39,-2.87) |
| Puerto Rico | female | 15.75(13.00,18.92) | 12.10(9.32,15.49) | -1.66(-2.18,-1.14) | 15.75(13.00,18.92) | 12.10(9.32,15.49) | -1.66(-2.18,-1.14) |
| Qatar | female | 10.99(4.53,23.09) | 6.15(3.97,9.39) | -2.06(-2.14,-1.98) | 10.99(4.53,23.09) | 6.15(3.97,9.39) | -2.06(-2.14,-1.98) |
| Republic of Korea | female | 49.99(48.62,51.39) | 13.26(12.44,14.13) | -5.39(-5.84,-4.93) | 49.99(48.62,51.39) | 13.26(12.44,14.13) | -5.39(-5.84,-4.93) |
| Republic of Moldova | female | 22.37(19.37,25.74) | 12.17(9.47,15.51) | -1.80(-2.21,-1.39) | 22.37(19.37,25.74) | 12.17(9.47,15.51) | -1.80(-2.21,-1.39) |
| Romania | female | 38.75(36.89,40.67) | 20.71(19.00,22.53) | -2.06(-2.24,-1.89) | 38.75(36.89,40.67) | 20.71(19.00,22.53) | -2.06(-2.24,-1.89) |
| Russian Federation | female | 19.37(18.87,19.89) | 14.16(13.67,14.66) | -0.85(-1.42,-0.29) | 19.37(18.87,19.89) | 14.16(13.67,14.66) | -0.85(-1.42,-0.29) |
| Rwanda | female | 90.58(85.64,95.76) | 35.50(33.37,37.73) | -3.31(-3.63,-2.99) | 90.58(85.64,95.76) | 35.50(33.37,37.73) | -3.31(-3.63,-2.99) |
| Saint Kitts and Nevis | female | 38.76(8.81,113.99) | 8.63(0.19,53.51) | -5.59(-6.04,-5.15) | 38.76(8.81,113.99) | 8.63(0.19,53.51) | -5.59(-6.04,-5.15) |
| Saint Lucia | female | 44.27(23.08,79.10) | 19.72(7.50,43.17) | -3.21(-3.52,-2.89) | 44.27(23.08,79.10) | 19.72(7.50,43.17) | -3.21(-3.52,-2.89) |
| Saint Vincent and the Grenadines | female | 19.31(5.45,52.72) | 12.75(2.22,40.63) | -2.45(-2.92,-1.97) | 19.31(5.45,52.72) | 12.75(2.22,40.63) | -2.45(-2.92,-1.97) |
| Samoa | female | 8.58(1.32,30.64) | 9.23(2.20,26.18) | 0.34(0.22,0.47) | 8.58(1.32,30.64) | 9.23(2.20,26.18) | 0.34(0.22,0.47) |
| San Marino | female | 13.27(0.05,110.32) | 9.12(0.00,107.66) | -1.41(-1.57,-1.24) | 13.27(0.05,110.32) | 9.12(0.00,107.66) | -1.41(-1.57,-1.24) |
| Sao Tome and Principe | female | 5.18(0.13,33.82) | 4.81(0.62,17.63) | -0.80(-1.36,-0.24) | 5.18(0.13,33.82) | 4.81(0.62,17.63) | -0.80(-1.36,-0.24) |
| Saudi Arabia | female | 11.10(9.84,12.50) | 6.33(5.79,6.91) | -1.66(-1.74,-1.59) | 11.10(9.84,12.50) | 6.33(5.79,6.91) | -1.66(-1.74,-1.59) |
| Senegal | female | 7.76(6.35,9.43) | 5.90(5.07,6.83) | -0.95(-1.16,-0.74) | 7.76(6.35,9.43) | 5.90(5.07,6.83) | -0.95(-1.16,-0.74) |
| Serbia | female | 49.93(46.67,53.36) | 18.62(16.43,21.03) | -3.58(-3.77,-3.39) | 49.93(46.67,53.36) | 18.62(16.43,21.03) | -3.58(-3.77,-3.39) |
| Seychelles | female | 15.62(2.22,59.88) | 19.27(4.50,54.50) | 0.72(0.30,1.13) | 15.62(2.22,59.88) | 19.27(4.50,54.50) | 0.72(0.30,1.13) |
| Sierra Leone | female | 9.69(7.68,12.11) | 8.39(7.11,9.85) | -0.34(-0.46,-0.22) | 9.69(7.68,12.11) | 8.39(7.11,9.85) | -0.34(-0.46,-0.22) |
| Singapore | female | 15.03(12.34,18.17) | 7.85(6.07,10.11) | -2.46(-2.57,-2.36) | 15.03(12.34,18.17) | 7.85(6.07,10.11) | -2.46(-2.57,-2.36) |
| Slovakia | female | 51.47(47.12,56.12) | 27.32(23.78,31.29) | -2.14(-2.38,-1.90) | 51.47(47.12,56.12) | 27.32(23.78,31.29) | -2.14(-2.38,-1.90) |
| Slovenia | female | 52.22(45.16,60.12) | 19.67(14.69,25.94) | -3.53(-4.02,-3.03) | 52.22(45.16,60.12) | 19.67(14.69,25.94) | -3.53(-4.02,-3.03) |
| Solomon Islands | female | 7.13(1.86,19.85) | 6.77(3.06,13.05) | -0.14(-0.30,0.02) | 7.13(1.86,19.85) | 6.77(3.06,13.05) | -0.14(-0.30,0.02) |
| Somalia | female | 11.65(9.91,13.62) | 8.74(7.82,9.75) | -0.47(-0.78,-0.15) | 11.65(9.91,13.62) | 8.74(7.82,9.75) | -0.47(-0.78,-0.15) |
| South Africa | female | 13.10(12.29,13.94) | 6.81(6.36,7.28) | -2.18(-2.60,-1.76) | 13.10(12.29,13.94) | 6.81(6.36,7.28) | -2.18(-2.60,-1.76) |
| South Sudan | female | 10.34(8.45,12.58) | 9.94(8.54,11.53) | -0.17(-0.47,0.13) | 10.34(8.45,12.58) | 9.94(8.54,11.53) | -0.17(-0.47,0.13) |
| Spain | female | 21.04(20.00,22.12) | 10.44(9.65,11.29) | -2.46(-2.69,-2.23) | 21.04(20.00,22.12) | 10.44(9.65,11.29) | -2.46(-2.69,-2.23) |
| Sri Lanka | female | 17.30(15.98,18.70) | 10.82(9.84,11.88) | -1.90(-2.12,-1.67) | 17.30(15.98,18.70) | 10.82(9.84,11.88) | -1.90(-2.12,-1.67) |
| Sudan | female | 8.21(7.31,9.21) | 5.68(5.19,6.20) | -1.11(-1.21,-1.01) | 8.21(7.31,9.21) | 5.68(5.19,6.20) | -1.11(-1.21,-1.01) |
| Suriname | female | 61.46(45.31,82.06) | 37.03(26.46,50.48) | -1.97(-2.52,-1.42) | 61.46(45.31,82.06) | 37.03(26.46,50.48) | -1.97(-2.52,-1.42) |
| Sweden | female | 45.85(42.39,49.54) | 9.56(8.09,11.24) | -4.94(-5.16,-4.72) | 45.85(42.39,49.54) | 9.56(8.09,11.24) | -4.94(-5.16,-4.72) |
| Switzerland | female | 53.76(49.67,58.13) | 15.47(13.39,17.82) | -4.08(-4.20,-3.96) | 53.76(49.67,58.13) | 15.47(13.39,17.82) | -4.08(-4.20,-3.96) |
| Syrian Arab Republic | female | 6.66(5.58,7.90) | 3.92(3.16,4.83) | -1.84(-1.96,-1.72) | 6.66(5.58,7.90) | 3.92(3.16,4.83) | -1.84(-1.96,-1.72) |
| Taiwan (Province of China) | female | 10.48(9.57,11.47) | 27.14(25.43,28.95) | 4.19(3.46,4.93) | 10.48(9.57,11.47) | 27.14(25.43,28.95) | 4.19(3.46,4.93) |
| Tajikistan | female | 9.97(8.09,12.20) | 5.78(4.78,6.92) | -1.96(-2.33,-1.59) | 9.97(8.09,12.20) | 5.78(4.78,6.92) | -1.96(-2.33,-1.59) |
| Thailand | female | 24.33(23.48,25.19) | 21.33(20.47,22.22) | -1.33(-2.09,-0.56) | 24.33(23.48,25.19) | 21.33(20.47,22.22) | -1.33(-2.09,-0.56) |
| Timor-Leste | female | 15.13(9.55,23.00) | 12.72(8.80,18.00) | -0.70(-1.17,-0.22) | 15.13(9.55,23.00) | 12.72(8.80,18.00) | -0.70(-1.17,-0.22) |
| Togo | female | 15.92(13.07,19.25) | 13.18(11.52,15.01) | -0.51(-0.58,-0.43) | 15.92(13.07,19.25) | 13.18(11.52,15.01) | -0.51(-0.58,-0.43) |
| Tokelau | female | 8.81(0.00,1533.13) | 9.29(0.00,1664.13) | -0.56(-0.85,-0.26) | 8.81(0.00,1533.13) | 9.29(0.00,1664.13) | -0.56(-0.85,-0.26) |
| Tonga | female | 6.62(0.20,39.62) | 4.85(0.09,29.94) | -1.33(-1.56,-1.11) | 6.62(0.20,39.62) | 4.85(0.09,29.94) | -1.33(-1.56,-1.11) |
| Trinidad and Tobago | female | 17.52(12.70,23.65) | 11.11(7.33,16.27) | -1.81(-1.99,-1.64) | 17.52(12.70,23.65) | 11.11(7.33,16.27) | -1.81(-1.99,-1.64) |
| Tunisia | female | 22.79(20.51,25.27) | 16.55(14.91,18.33) | -0.92(-1.03,-0.80) | 22.79(20.51,25.27) | 16.55(14.91,18.33) | -0.92(-1.03,-0.80) |
| Turkey | female | 8.22(7.70,8.78) | 5.11(4.77,5.48) | -1.51(-1.96,-1.06) | 8.22(7.70,8.78) | 5.11(4.77,5.48) | -1.51(-1.96,-1.06) |
| Turkmenistan | female | 9.63(7.52,12.19) | 6.08(4.63,7.85) | -1.38(-1.62,-1.15) | 9.63(7.52,12.19) | 6.08(4.63,7.85) | -1.38(-1.62,-1.15) |
| Tuvalu | female | 13.47(0.00,229.74) | 9.07(0.00,202.14) | -1.38(-1.49,-1.27) | 13.47(0.00,229.74) | 9.07(0.00,202.14) | -1.38(-1.49,-1.27) |
| Uganda | female | 14.54(13.22,15.96) | 32.15(30.97,33.37) | 1.81(1.28,2.34) | 14.54(13.22,15.96) | 32.15(30.97,33.37) | 1.81(1.28,2.34) |
| Ukraine | female | 17.91(17.07,18.78) | 12.95(12.10,13.86) | -1.07(-1.31,-0.83) | 17.91(17.07,18.78) | 12.95(12.10,13.86) | -1.07(-1.31,-0.83) |
| United Arab Emirates | female | 46.64(39.16,55.22) | 35.41(31.41,39.82) | -1.11(-1.75,-0.45) | 46.64(39.16,55.22) | 35.41(31.41,39.82) | -1.11(-1.75,-0.45) |
| United Kingdom | female | 18.26(17.44,19.11) | 9.59(9.02,10.19) | -2.73(-3.10,-2.36) | 18.26(17.44,19.11) | 9.59(9.02,10.19) | -2.73(-3.10,-2.36) |
| United Republic of Tanzania | female | 11.44(10.50,12.45) | 10.68(10.09,11.28) | -0.11(-0.18,-0.05) | 11.44(10.50,12.45) | 10.68(10.09,11.28) | -0.11(-0.18,-0.05) |
| United States of America | female | 21.45(21.04,21.86) | 13.24(12.94,13.54) | -1.66(-2.21,-1.11) | 21.45(21.04,21.86) | 13.24(12.94,13.54) | -1.66(-2.21,-1.11) |
| United States Virgin Islands | female | 22.70(7.05,55.20) | 13.27(1.02,59.36) | -1.46(-1.78,-1.15) | 22.70(7.05,55.20) | 13.27(1.02,59.36) | -1.46(-1.78,-1.15) |
| Uruguay | female | 33.95(29.34,39.08) | 25.63(21.75,30.02) | -1.15(-1.32,-0.99) | 33.95(29.34,39.08) | 25.63(21.75,30.02) | -1.15(-1.32,-0.99) |
| Uzbekistan | female | 10.47(9.51,11.51) | 9.97(9.24,10.75) | 0.21(-0.04,0.46) | 10.47(9.51,11.51) | 9.97(9.24,10.75) | 0.21(-0.04,0.46) |
| Vanuatu | female | 8.91(1.46,30.43) | 8.28(2.70,19.73) | -0.43(-0.53,-0.33) | 8.91(1.46,30.43) | 8.28(2.70,19.73) | -0.43(-0.53,-0.33) |
| Venezuela (Bolivarian Republic of) | female | 21.78(20.36,23.28) | 18.85(17.65,20.11) | -0.59(-0.82,-0.37) | 21.78(20.36,23.28) | 18.85(17.65,20.11) | -0.59(-0.82,-0.37) |
| Viet Nam | female | 23.94(23.13,24.76) | 26.17(25.44,26.91) | 0.79(0.43,1.16) | 23.94(23.13,24.76) | 26.17(25.44,26.91) | 0.79(0.43,1.16) |
| Yemen | female | 9.83(8.57,11.24) | 6.05(5.47,6.67) | -1.64(-1.76,-1.53) | 9.83(8.57,11.24) | 6.05(5.47,6.67) | -1.64(-1.76,-1.53) |
| Zambia | female | 40.41(37.20,43.87) | 37.41(35.55,39.35) | -0.25(-0.59,0.10) | 40.41(37.20,43.87) | 37.41(35.55,39.35) | -0.25(-0.59,0.10) |
| Zimbabwe | female | 5.33(4.32,6.51) | 17.23(15.83,18.73) | 6.11(4.75,7.49) | 5.33(4.32,6.51) | 17.23(15.83,18.73) | 6.11(4.75,7.49) |
| Afghanistan | male | 12.01(9.83,14.64) | 12.71(11.76,13.72) | 0.65(0.43,0.87) | 12.01(9.83,14.64) | 12.71(11.76,13.72) | 0.65(0.43,0.87) |
| Albania | male | 43.97(39.22,49.16) | 36.02(30.96,41.72) | -0.83(-1.31,-0.34) | 43.97(39.22,49.16) | 36.02(30.96,41.72) | -0.83(-1.31,-0.34) |
| Algeria | male | 19.06(17.82,20.36) | 12.66(11.92,13.43) | -1.22(-1.34,-1.10) | 19.06(17.82,20.36) | 12.66(11.92,13.43) | -1.22(-1.34,-1.10) |
| American Samoa | male | 7.59(0.06,58.85) | 13.14(0.43,72.97) | 3.04(2.11,3.97) | 7.59(0.06,58.85) | 13.14(0.43,72.97) | 3.04(2.11,3.97) |
| Andorra | male | 100.29(52.56,177.78) | 66.91(29.73,133.17) | -1.19(-1.58,-0.80) | 100.29(52.56,177.78) | 66.91(29.73,133.17) | -1.19(-1.58,-0.80) |
| Angola | male | 37.87(35.20,40.70) | 41.16(39.48,42.88) | 0.91(0.55,1.27) | 37.87(35.20,40.70) | 41.16(39.48,42.88) | 0.91(0.55,1.27) |
| Antigua and Barbuda | male | 95.12(48.75,169.58) | 31.80(10.83,73.62) | -3.93(-4.57,-3.30) | 95.12(48.75,169.58) | 31.80(10.83,73.62) | -3.93(-4.57,-3.30) |
| Argentina | male | 69.32(67.23,71.46) | 43.78(42.40,45.19) | -1.56(-2.09,-1.03) | 69.32(67.23,71.46) | 43.78(42.40,45.19) | -1.56(-2.09,-1.03) |
| Armenia | male | 18.94(15.88,22.45) | 37.13(32.11,42.77) | 3.22(2.01,4.45) | 18.94(15.88,22.45) | 37.13(32.11,42.77) | 3.22(2.01,4.45) |
| Australia | male | 73.41(70.54,76.37) | 26.19(24.68,27.77) | -3.16(-3.40,-2.91) | 73.41(70.54,76.37) | 26.19(24.68,27.77) | -3.16(-3.40,-2.91) |
| Austria | male | 88.63(83.89,93.59) | 38.75(35.56,42.18) | -2.99(-3.12,-2.86) | 88.63(83.89,93.59) | 38.75(35.56,42.18) | -2.99(-3.12,-2.86) |
| Azerbaijan | male | 20.70(18.45,23.18) | 10.92(9.59,12.41) | -2.85(-3.68,-2.01) | 20.70(18.45,23.18) | 10.92(9.59,12.41) | -2.85(-3.68,-2.01) |
| Bahamas | male | 137.13(108.48,171.60) | 68.92(51.47,90.40) | -2.28(-2.54,-2.02) | 137.13(108.48,171.60) | 68.92(51.47,90.40) | -2.28(-2.54,-2.02) |
| Bahrain | male | 19.30(12.82,28.69) | 13.86(10.63,17.95) | -1.13(-1.43,-0.84) | 19.30(12.82,28.69) | 13.86(10.63,17.95) | -1.13(-1.43,-0.84) |
| Bangladesh | male | 58.62(57.60,59.65) | 46.97(46.23,47.71) | -0.20(-0.47,0.09) | 58.62(57.60,59.65) | 46.97(46.23,47.71) | -0.20(-0.47,0.09) |
| Barbados | male | 77.66(55.92,105.17) | 46.56(29.40,70.32) | -2.36(-2.81,-1.91) | 77.66(55.92,105.17) | 46.56(29.40,70.32) | -2.36(-2.81,-1.91) |
| Belarus | male | 170.95(165.16,176.90) | 65.85(61.71,70.23) | -3.35(-4.07,-2.62) | 170.95(165.16,176.90) | 65.85(61.71,70.23) | -3.35(-4.07,-2.62) |
| Belgium | male | 102.58(97.99,107.34) | 46.78(43.63,50.10) | -2.80(-3.04,-2.55) | 102.58(97.99,107.34) | 46.78(43.63,50.10) | -2.80(-3.04,-2.55) |
| Belize | male | 329.88(273.30,396.08) | 198.34(170.55,229.55) | -2.90(-3.58,-2.22) | 329.88(273.30,396.08) | 198.34(170.55,229.55) | -2.90(-3.58,-2.22) |
| Benin | male | 49.33(44.38,54.73) | 35.62(33.24,38.13) | -1.30(-1.60,-1.00) | 49.33(44.38,54.73) | 35.62(33.24,38.13) | -1.30(-1.60,-1.00) |
| Bermuda | male | 160.65(97.69,253.15) | 25.72(3.52,94.48) | -6.43(-6.96,-5.89) | 160.65(97.69,253.15) | 25.72(3.52,94.48) | -6.43(-6.96,-5.89) |
| Bhutan | male | 45.72(35.03,59.09) | 22.64(16.23,30.86) | -2.87(-3.09,-2.65) | 45.72(35.03,59.09) | 22.64(16.23,30.86) | -2.87(-3.09,-2.65) |
| Bolivia (Plurinational State of) | male | 30.08(26.98,33.46) | 18.36(16.70,20.14) | -1.55(-1.73,-1.37) | 30.08(26.98,33.46) | 18.36(16.70,20.14) | -1.55(-1.73,-1.37) |
| Bosnia and Herzegovina | male | 47.92(43.70,52.44) | 44.37(38.79,50.60) | -0.10(-0.39,0.19) | 47.92(43.70,52.44) | 44.37(38.79,50.60) | -0.10(-0.39,0.19) |
| Botswana | male | 25.54(19.43,33.18) | 19.67(16.07,23.85) | -1.27(-1.64,-0.91) | 25.54(19.43,33.18) | 19.67(16.07,23.85) | -1.27(-1.64,-0.91) |
| Brazil | male | 75.62(74.64,76.61) | 78.95(78.10,79.80) | 0.42(-0.36,1.21) | 75.62(74.64,76.61) | 78.95(78.10,79.80) | 0.42(-0.36,1.21) |
| Brunei Darussalam | male | 169.06(138.75,204.25) | 65.31(51.22,82.47) | -3.38(-3.97,-2.79) | 169.06(138.75,204.25) | 65.31(51.22,82.47) | -3.38(-3.97,-2.79) |
| Bulgaria | male | 58.57(54.76,62.57) | 53.12(48.57,58.01) | -0.53(-0.80,-0.26) | 58.57(54.76,62.57) | 53.12(48.57,58.01) | -0.53(-0.80,-0.26) |
| Burkina Faso | male | 51.12(47.33,55.16) | 56.06(53.68,58.52) | 0.67(0.40,0.95) | 51.12(47.33,55.16) | 56.06(53.68,58.52) | 0.67(0.40,0.95) |
| Burundi | male | 161.42(153.66,169.50) | 74.66(71.36,78.08) | -3.08(-3.40,-2.76) | 161.42(153.66,169.50) | 74.66(71.36,78.08) | -3.08(-3.40,-2.76) |
| Cabo Verde | male | 31.05(17.18,53.63) | 35.61(26.10,47.60) | 0.18(-0.35,0.71) | 31.05(17.18,53.63) | 35.61(26.10,47.60) | 0.18(-0.35,0.71) |
| Cambodia | male | 73.36(69.27,77.64) | 97.34(94.16,100.60) | 0.53(0.30,0.77) | 73.36(69.27,77.64) | 97.34(94.16,100.60) | 0.53(0.30,0.77) |
| Cameroon | male | 57.82(54.24,61.60) | 59.76(57.81,61.76) | 0.12(-0.11,0.35) | 57.82(54.24,61.60) | 59.76(57.81,61.76) | 0.12(-0.11,0.35) |
| Canada | male | 71.71(69.44,74.03) | 36.15(34.64,37.72) | -1.95(-2.18,-1.72) | 71.71(69.44,74.03) | 36.15(34.64,37.72) | -1.95(-2.18,-1.72) |
| Central African Republic | male | 65.38(58.47,72.98) | 56.95(52.39,61.83) | -0.43(-0.50,-0.35) | 65.38(58.47,72.98) | 56.95(52.39,61.83) | -0.43(-0.50,-0.35) |
| Chad | male | 25.97(22.79,29.51) | 40.27(37.91,42.76) | 1.69(1.29,2.08) | 25.97(22.79,29.51) | 40.27(37.91,42.76) | 1.69(1.29,2.08) |
| Chile | male | 46.52(44.01,49.14) | 60.35(57.85,62.94) | 0.65(0.05,1.25) | 46.52(44.01,49.14) | 60.35(57.85,62.94) | 0.65(0.05,1.25) |
| China | male | 86.60(86.25,86.94) | 113.15(112.72,113.58) | 0.74(0.18,1.31) | 86.60(86.25,86.94) | 113.15(112.72,113.58) | 0.74(0.18,1.31) |
| Colombia | male | 95.99(93.66,98.37) | 87.43(85.62,89.28) | -1.15(-1.78,-0.51) | 95.99(93.66,98.37) | 87.43(85.62,89.28) | -1.15(-1.78,-0.51) |
| Comoros | male | 62.89(46.81,83.47) | 50.80(40.20,63.45) | -1.58(-2.37,-0.79) | 62.89(46.81,83.47) | 50.80(40.20,63.45) | -1.58(-2.37,-0.79) |
| Congo | male | 53.25(46.61,60.67) | 39.40(35.73,43.36) | -1.19(-1.58,-0.80) | 53.25(46.61,60.67) | 39.40(35.73,43.36) | -1.19(-1.58,-0.80) |
| Cook Islands | male | 18.91(0.13,150.60) | 43.86(1.76,226.44) | 3.34(2.99,3.70) | 18.91(0.13,150.60) | 43.86(1.76,226.44) | 3.34(2.99,3.70) |
| Costa Rica | male | 113.72(105.51,122.43) | 93.02(86.92,99.46) | -1.43(-1.83,-1.02) | 113.72(105.51,122.43) | 93.02(86.92,99.46) | -1.43(-1.83,-1.02) |
| Croatia | male | 78.52(72.89,84.48) | 52.93(47.40,58.97) | -1.85(-2.56,-1.15) | 78.52(72.89,84.48) | 52.93(47.40,58.97) | -1.85(-2.56,-1.15) |
| Cuba | male | 784.34(773.24,795.57) | 125.21(120.13,130.47) | -7.17(-7.80,-6.55) | 784.34(773.24,795.57) | 125.21(120.13,130.47) | -7.17(-7.80,-6.55) |
| Cyprus | male | 70.01(57.55,84.43) | 35.90(28.81,44.49) | -2.74(-3.21,-2.27) | 70.01(57.55,84.43) | 35.90(28.81,44.49) | -2.74(-3.21,-2.27) |
| Czechia | male | 81.22(77.18,85.42) | 49.56(46.03,53.30) | -2.95(-3.88,-2.01) | 81.22(77.18,85.42) | 49.56(46.03,53.30) | -2.95(-3.88,-2.01) |
| Côte d'Ivoire | male | 34.83(32.45,37.35) | 39.10(37.46,40.78) | 0.09(-0.13,0.31) | 34.83(32.45,37.35) | 39.10(37.46,40.78) | 0.09(-0.13,0.31) |
| Democratic People's Republic of Korea | male | 89.13(86.19,92.16) | 127.67(124.63,130.78) | 0.99(0.81,1.17) | 89.13(86.19,92.16) | 127.67(124.63,130.78) | 0.99(0.81,1.17) |
| Democratic Republic of the Congo | male | 35.21(33.82,36.64) | 34.15(33.29,35.02) | -0.03(-0.22,0.16) | 35.21(33.82,36.64) | 34.15(33.29,35.02) | -0.03(-0.22,0.16) |
| Denmark | male | 113.25(106.65,120.17) | 37.20(33.37,41.38) | -4.07(-4.36,-3.78) | 113.25(106.65,120.17) | 37.20(33.37,41.38) | -4.07(-4.36,-3.78) |
| Djibouti | male | 42.12(29.73,58.53) | 47.15(39.57,55.78) | 0.23(-0.06,0.52) | 42.12(29.73,58.53) | 47.15(39.57,55.78) | 0.23(-0.06,0.52) |
| Dominica | male | 99.44(55.74,167.10) | 78.12(38.08,142.27) | -1.11(-1.24,-0.98) | 99.44(55.74,167.10) | 78.12(38.08,142.27) | -1.11(-1.24,-0.98) |
| Dominican Republic | male | 67.97(63.71,72.46) | 66.42(63.13,69.84) | 0.34(-0.12,0.80) | 67.97(63.71,72.46) | 66.42(63.13,69.84) | 0.34(-0.12,0.80) |
| Ecuador | male | 64.23(60.69,67.94) | 86.05(83.07,89.12) | 2.14(1.31,2.97) | 64.23(60.69,67.94) | 86.05(83.07,89.12) | 2.14(1.31,2.97) |
| Egypt | male | 34.63(33.53,35.75) | 33.29(32.52,34.07) | 0.58(0.16,1.00) | 34.63(33.53,35.75) | 33.29(32.52,34.07) | 0.58(0.16,1.00) |
| El Salvador | male | 65.49(60.44,70.88) | 53.60(49.50,57.96) | -0.89(-1.30,-0.47) | 65.49(60.44,70.88) | 53.60(49.50,57.96) | -0.89(-1.30,-0.47) |
| Equatorial Guinea | male | 56.32(39.85,78.01) | 35.40(29.58,42.17) | -1.46(-1.91,-1.00) | 56.32(39.85,78.01) | 35.40(29.58,42.17) | -1.46(-1.91,-1.00) |
| Eritrea | male | 103.53(95.67,111.93) | 87.95(83.15,92.97) | -1.14(-1.43,-0.85) | 103.53(95.67,111.93) | 87.95(83.15,92.97) | -1.14(-1.43,-0.85) |
| Estonia | male | 145.53(131.83,160.30) | 30.84(23.60,39.80) | -5.18(-5.84,-4.51) | 145.53(131.83,160.30) | 30.84(23.60,39.80) | -5.18(-5.84,-4.51) |
| Eswatini | male | 36.10(26.12,49.00) | 71.73(61.35,83.44) | 2.64(1.72,3.57) | 36.10(26.12,49.00) | 71.73(61.35,83.44) | 2.64(1.72,3.57) |
| Ethiopia | male | 72.68(70.90,74.50) | 30.80(30.06,31.54) | -3.09(-3.46,-2.72) | 72.68(70.90,74.50) | 30.80(30.06,31.54) | -3.09(-3.46,-2.72) |
| Fiji | male | 16.23(10.58,23.98) | 16.27(10.95,23.28) | 0.37(0.15,0.58) | 16.23(10.58,23.98) | 16.27(10.95,23.28) | 0.37(0.15,0.58) |
| Finland | male | 137.56(129.93,145.56) | 47.09(42.51,52.05) | -3.07(-3.30,-2.83) | 137.56(129.93,145.56) | 47.09(42.51,52.05) | -3.07(-3.30,-2.83) |
| France | male | 59.80(58.36,61.26) | 33.96(32.82,35.12) | -1.92(-2.12,-1.72) | 59.80(58.36,61.26) | 33.96(32.82,35.12) | -1.92(-2.12,-1.72) |
| Gabon | male | 62.19(51.51,74.54) | 53.20(45.80,61.51) | -0.68(-0.82,-0.54) | 62.19(51.51,74.54) | 53.20(45.80,61.51) | -0.68(-0.82,-0.54) |
| Gambia | male | 29.56(21.96,39.23) | 38.14(32.67,44.34) | 0.60(0.28,0.93) | 29.56(21.96,39.23) | 38.14(32.67,44.34) | 0.60(0.28,0.93) |
| Georgia | male | 60.36(55.76,65.26) | 38.39(33.51,43.82) | -1.10(-2.09,-0.09) | 60.36(55.76,65.26) | 38.39(33.51,43.82) | -1.10(-2.09,-0.09) |
| Germany | male | 89.29(87.74,90.87) | 37.73(36.68,38.82) | -3.18(-3.34,-3.02) | 89.29(87.74,90.87) | 37.73(36.68,38.82) | -3.18(-3.34,-3.02) |
| Ghana | male | 67.09(64.03,70.27) | 81.68(79.54,83.87) | 0.96(0.84,1.08) | 67.09(64.03,70.27) | 81.68(79.54,83.87) | 0.96(0.84,1.08) |
| Greece | male | 33.52(30.96,36.23) | 26.84(24.20,29.70) | -0.64(-0.76,-0.53) | 33.52(30.96,36.23) | 26.84(24.20,29.70) | -0.64(-0.76,-0.53) |
| Greenland | male | 71.44(33.17,140.96) | 25.19(4.33,85.20) | -3.52(-3.84,-3.19) | 71.44(33.17,140.96) | 25.19(4.33,85.20) | -3.52(-3.84,-3.19) |
| Grenada | male | 175.04(116.97,254.08) | 86.71(51.40,138.15) | -2.64(-3.09,-2.19) | 175.04(116.97,254.08) | 86.71(51.40,138.15) | -2.64(-3.09,-2.19) |
| Guam | male | 11.18(2.98,29.91) | 18.39(6.26,42.20) | 2.73(1.71,3.76) | 11.18(2.98,29.91) | 18.39(6.26,42.20) | 2.73(1.71,3.76) |
| Guatemala | male | 53.56(49.70,57.65) | 46.34(43.98,48.80) | -0.46(-0.75,-0.18) | 53.56(49.70,57.65) | 46.34(43.98,48.80) | -0.46(-0.75,-0.18) |
| Guinea | male | 29.07(25.66,32.82) | 33.10(30.74,35.61) | 0.45(0.37,0.53) | 29.07(25.66,32.82) | 33.10(30.74,35.61) | 0.45(0.37,0.53) |
| Guinea-Bissau | male | 100.35(85.78,116.96) | 82.97(74.11,92.68) | -0.56(-0.60,-0.51) | 100.35(85.78,116.96) | 82.97(74.11,92.68) | -0.56(-0.60,-0.51) |
| Guyana | male | 160.76(141.90,181.61) | 190.54(169.10,214.11) | 0.14(-0.50,0.79) | 160.76(141.90,181.61) | 190.54(169.10,214.11) | 0.14(-0.50,0.79) |
| Haiti | male | 216.17(207.74,224.88) | 143.36(138.85,147.99) | -1.16(-1.47,-0.86) | 216.17(207.74,224.88) | 143.36(138.85,147.99) | -1.16(-1.47,-0.86) |
| Honduras | male | 70.24(64.47,76.43) | 35.22(32.67,37.93) | -2.88(-3.24,-2.52) | 70.24(64.47,76.43) | 35.22(32.67,37.93) | -2.88(-3.24,-2.52) |
| Hungary | male | 222.07(215.35,228.96) | 80.92(76.25,85.83) | -4.18(-4.66,-3.71) | 222.07(215.35,228.96) | 80.92(76.25,85.83) | -4.18(-4.66,-3.71) |
| Iceland | male | 42.44(26.72,64.21) | 22.55(12.31,38.52) | -2.20(-2.34,-2.06) | 42.44(26.72,64.21) | 22.55(12.31,38.52) | -2.20(-2.34,-2.06) |
| India | male | 114.61(114.11,115.12) | 104.95(104.59,105.31) | -0.30(-0.68,0.08) | 114.61(114.11,115.12) | 104.95(104.59,105.31) | -0.30(-0.68,0.08) |
| Indonesia | male | 67.44(66.62,68.28) | 61.79(61.15,62.43) | -0.27(-0.39,-0.14) | 67.44(66.62,68.28) | 61.79(61.15,62.43) | -0.27(-0.39,-0.14) |
| Iran (Islamic Republic of) | male | 24.50(23.54,25.48) | 26.63(25.86,27.42) | 0.31(-0.20,0.83) | 24.50(23.54,25.48) | 26.63(25.86,27.42) | 0.31(-0.20,0.83) |
| Iraq | male | 23.28(21.70,24.96) | 15.25(14.45,16.09) | -1.23(-1.33,-1.12) | 23.28(21.70,24.96) | 15.25(14.45,16.09) | -1.23(-1.33,-1.12) |
| Ireland | male | 105.51(98.02,113.44) | 30.61(26.84,34.78) | -4.32(-4.60,-4.05) | 105.51(98.02,113.44) | 30.61(26.84,34.78) | -4.32(-4.60,-4.05) |
| Israel | male | 45.21(41.04,49.71) | 32.16(29.50,34.99) | -1.38(-1.75,-1.02) | 45.21(41.04,49.71) | 32.16(29.50,34.99) | -1.38(-1.75,-1.02) |
| Italy | male | 94.81(92.98,96.67) | 42.76(41.35,44.21) | -2.65(-2.74,-2.56) | 94.81(92.98,96.67) | 42.76(41.35,44.21) | -2.65(-2.74,-2.56) |
| Jamaica | male | 42.72(36.95,49.22) | 40.58(35.62,46.06) | -1.43(-2.26,-0.60) | 42.72(36.95,49.22) | 40.58(35.62,46.06) | -1.43(-2.26,-0.60) |
| Japan | male | 85.21(84.02,86.42) | 26.44(25.66,27.25) | -3.90(-4.04,-3.77) | 85.21(84.02,86.42) | 26.44(25.66,27.25) | -3.90(-4.04,-3.77) |
| Jordan | male | 16.60(13.71,20.01) | 15.62(14.22,17.14) | 0.15(-0.10,0.41) | 16.60(13.71,20.01) | 15.62(14.22,17.14) | 0.15(-0.10,0.41) |
| Kazakhstan | male | 46.92(44.65,49.28) | 25.83(24.18,27.57) | -1.29(-1.82,-0.74) | 46.92(44.65,49.28) | 25.83(24.18,27.57) | -1.29(-1.82,-0.74) |
| Kenya | male | 32.38(30.64,34.20) | 45.80(44.51,47.13) | 1.67(1.30,2.05) | 32.38(30.64,34.20) | 45.80(44.51,47.13) | 1.67(1.30,2.05) |
| Kiribati | male | 18.07(3.12,61.01) | 16.67(4.51,43.62) | -0.54(-0.76,-0.32) | 18.07(3.12,61.01) | 16.67(4.51,43.62) | -0.54(-0.76,-0.32) |
| Kuwait | male | 57.75(50.88,65.42) | 59.74(54.81,65.07) | 0.27(-1.30,1.86) | 57.75(50.88,65.42) | 59.74(54.81,65.07) | 0.27(-1.30,1.86) |
| Kyrgyzstan | male | 58.31(53.36,63.62) | 35.39(32.32,38.69) | -1.19(-1.98,-0.39) | 58.31(53.36,63.62) | 35.39(32.32,38.69) | -1.19(-1.98,-0.39) |
| Lao People's Democratic Republic | male | 90.75(83.94,98.02) | 78.78(74.51,83.24) | -0.66(-0.75,-0.57) | 90.75(83.94,98.02) | 78.78(74.51,83.24) | -0.66(-0.75,-0.57) |
| Latvia | male | 193.82(181.52,206.76) | 57.70(48.98,67.69) | -4.09(-4.45,-3.73) | 193.82(181.52,206.76) | 57.70(48.98,67.69) | -4.09(-4.45,-3.73) |
| Lebanon | male | 34.91(30.02,40.43) | 17.94(15.60,20.56) | -1.93(-2.25,-1.62) | 34.91(30.02,40.43) | 17.94(15.60,20.56) | -1.93(-2.25,-1.62) |
| Lesotho | male | 21.51(15.82,28.69) | 53.28(46.33,61.01) | 3.81(3.21,4.42) | 21.51(15.82,28.69) | 53.28(46.33,61.01) | 3.81(3.21,4.42) |
| Liberia | male | 33.20(28.01,39.10) | 31.07(27.81,34.63) | 0.16(-0.17,0.50) | 33.20(28.01,39.10) | 31.07(27.81,34.63) | 0.16(-0.17,0.50) |
| Libya | male | 16.89(14.21,19.96) | 13.62(11.86,15.59) | -0.32(-0.50,-0.14) | 16.89(14.21,19.96) | 13.62(11.86,15.59) | -0.32(-0.50,-0.14) |
| Lithuania | male | 200.21(189.86,211.00) | 71.89(63.93,80.68) | -3.12(-3.90,-2.35) | 200.21(189.86,211.00) | 71.89(63.93,80.68) | -3.12(-3.90,-2.35) |
| Luxembourg | male | 64.38(46.89,86.89) | 26.46(17.75,38.46) | -2.93(-3.16,-2.70) | 64.38(46.89,86.89) | 26.46(17.75,38.46) | -2.93(-3.16,-2.70) |
| Madagascar | male | 55.08(52.03,58.29) | 42.21(40.52,43.95) | -0.71(-0.89,-0.53) | 55.08(52.03,58.29) | 42.21(40.52,43.95) | -0.71(-0.89,-0.53) |
| Malawi | male | 54.17(50.81,57.73) | 63.36(60.82,66.00) | 0.43(0.15,0.72) | 54.17(50.81,57.73) | 63.36(60.82,66.00) | 0.43(0.15,0.72) |
| Malaysia | male | 90.82(87.78,93.94) | 88.33(86.18,90.53) | -0.80(-1.17,-0.43) | 90.82(87.78,93.94) | 88.33(86.18,90.53) | -0.80(-1.17,-0.43) |
| Maldives | male | 51.48(30.45,83.23) | 12.08(7.12,20.37) | -5.27(-5.89,-4.64) | 51.48(30.45,83.23) | 12.08(7.12,20.37) | -5.27(-5.89,-4.64) |
| Mali | male | 30.07(27.19,33.19) | 25.09(23.51,26.76) | -0.34(-0.54,-0.14) | 30.07(27.19,33.19) | 25.09(23.51,26.76) | -0.34(-0.54,-0.14) |
| Malta | male | 32.56(20.59,49.32) | 22.29(12.51,37.82) | -1.38(-1.56,-1.20) | 32.56(20.59,49.32) | 22.29(12.51,37.82) | -1.38(-1.56,-1.20) |
| Marshall Islands | male | 13.23(0.44,75.23) | 11.82(0.76,53.13) | -0.03(-0.25,0.20) | 13.23(0.44,75.23) | 11.82(0.76,53.13) | -0.03(-0.25,0.20) |
| Mauritania | male | 74.20(65.52,83.80) | 47.96(43.18,53.15) | -1.54(-1.69,-1.39) | 74.20(65.52,83.80) | 47.96(43.18,53.15) | -1.54(-1.69,-1.39) |
| Mauritius | male | 7.84(4.77,12.18) | 43.61(35.52,53.06) | 7.07(5.94,8.22) | 7.84(4.77,12.18) | 43.61(35.52,53.06) | 7.07(5.94,8.22) |
| Mexico | male | 111.93(110.30,113.58) | 70.92(69.88,71.96) | -1.13(-1.57,-0.69) | 111.93(110.30,113.58) | 70.92(69.88,71.96) | -1.13(-1.57,-0.69) |
| Micronesia (Federated States of) | male | 15.78(3.21,47.68) | 16.77(4.04,46.93) | 0.24(0.14,0.34) | 15.78(3.21,47.68) | 16.77(4.04,46.93) | 0.24(0.14,0.34) |
| Monaco | male | 43.63(4.89,187.04) | 34.57(2.91,147.49) | -0.76(-0.82,-0.71) | 43.63(4.89,187.04) | 34.57(2.91,147.49) | -0.76(-0.82,-0.71) |
| Mongolia | male | 12.32(9.11,16.41) | 17.09(14.05,20.64) | 1.56(1.36,1.76) | 12.32(9.11,16.41) | 17.09(14.05,20.64) | 1.56(1.36,1.76) |
| Montenegro | male | 75.02(60.90,91.49) | 47.94(35.66,63.27) | -1.55(-1.97,-1.13) | 75.02(60.90,91.49) | 47.94(35.66,63.27) | -1.55(-1.97,-1.13) |
| Morocco | male | 16.14(15.03,17.32) | 11.89(11.12,12.70) | -1.02(-1.13,-0.90) | 16.14(15.03,17.32) | 11.89(11.12,12.70) | -1.02(-1.13,-0.90) |
| Mozambique | male | 50.95(47.98,54.06) | 89.17(86.66,91.74) | 2.42(2.05,2.80) | 50.95(47.98,54.06) | 89.17(86.66,91.74) | 2.42(2.05,2.80) |
| Myanmar | male | 74.52(72.68,76.40) | 59.14(57.71,60.59) | -0.82(-0.93,-0.70) | 74.52(72.68,76.40) | 59.14(57.71,60.59) | -0.82(-0.93,-0.70) |
| Namibia | male | 26.15(20.14,33.56) | 42.44(36.89,48.63) | 1.30(0.70,1.90) | 26.15(20.14,33.56) | 42.44(36.89,48.63) | 1.30(0.70,1.90) |
| Nauru | male | 30.18(0.08,263.48) | 42.46(0.94,258.30) | 1.09(0.73,1.45) | 30.18(0.08,263.48) | 42.46(0.94,258.30) | 1.09(0.73,1.45) |
| Nepal | male | 59.29(56.73,61.94) | 45.51(43.80,47.27) | -0.72(-0.95,-0.49) | 59.29(56.73,61.94) | 45.51(43.80,47.27) | -0.72(-0.95,-0.49) |
| Netherlands | male | 155.75(151.30,160.31) | 56.67(53.84,59.61) | -3.65(-3.89,-3.41) | 155.75(151.30,160.31) | 56.67(53.84,59.61) | -3.65(-3.89,-3.41) |
| New Zealand | male | 107.35(99.75,115.38) | 38.14(34.24,42.38) | -4.14(-4.59,-3.69) | 107.35(99.75,115.38) | 38.14(34.24,42.38) | -4.14(-4.59,-3.69) |
| Nicaragua | male | 68.22(62.00,74.95) | 54.86(51.06,58.87) | -0.58(-0.79,-0.37) | 68.22(62.00,74.95) | 54.86(51.06,58.87) | -0.58(-0.79,-0.37) |
| Niger | male | 30.89(27.91,34.14) | 29.98(28.26,31.78) | 0.15(0.04,0.25) | 30.89(27.91,34.14) | 29.98(28.26,31.78) | 0.15(0.04,0.25) |
| Nigeria | male | 29.38(28.55,30.23) | 29.06(28.52,29.62) | -0.01(-0.16,0.15) | 29.38(28.55,30.23) | 29.06(28.52,29.62) | -0.01(-0.16,0.15) |
| Niue | male | 14.26(0.00,1058.33) | 17.25(0.00,1461.47) | 0.25(0.03,0.47) | 14.26(0.00,1058.33) | 17.25(0.00,1461.47) | 0.25(0.03,0.47) |
| North Macedonia | male | 42.15(36.07,48.97) | 27.32(22.40,33.13) | -1.67(-1.88,-1.46) | 42.15(36.07,48.97) | 27.32(22.40,33.13) | -1.67(-1.88,-1.46) |
| Northern Mariana Islands | male | 7.46(0.14,54.96) | 9.99(0.15,67.06) | 1.40(0.52,2.29) | 7.46(0.14,54.96) | 9.99(0.15,67.06) | 1.40(0.52,2.29) |
| Norway | male | 58.24(53.14,63.71) | 24.69(21.58,28.15) | -2.41(-2.77,-2.05) | 58.24(53.14,63.71) | 24.69(21.58,28.15) | -2.41(-2.77,-2.05) |
| Oman | male | 30.50(25.97,35.67) | 42.69(39.06,46.65) | 2.37(1.61,3.13) | 30.50(25.97,35.67) | 42.69(39.06,46.65) | 2.37(1.61,3.13) |
| Pakistan | male | 57.81(56.77,58.87) | 93.41(92.56,94.27) | 1.45(1.12,1.79) | 57.81(56.77,58.87) | 93.41(92.56,94.27) | 1.45(1.12,1.79) |
| Palau | male | 7.80(0.00,129.61) | 7.21(0.00,145.17) | -0.16(-0.22,-0.09) | 7.80(0.00,129.61) | 7.21(0.00,145.17) | -0.16(-0.22,-0.09) |
| Palestine | male | 11.97(8.55,16.51) | 8.46(6.79,10.44) | -1.20(-1.32,-1.08) | 11.97(8.55,16.51) | 8.46(6.79,10.44) | -1.20(-1.32,-1.08) |
| Panama | male | 139.42(129.33,150.14) | 85.25(79.11,91.75) | -1.87(-2.15,-1.60) | 139.42(129.33,150.14) | 85.25(79.11,91.75) | -1.87(-2.15,-1.60) |
| Papua New Guinea | male | 14.11(11.63,17.00) | 11.39(10.00,12.92) | -0.87(-1.00,-0.75) | 14.11(11.63,17.00) | 11.39(10.00,12.92) | -0.87(-1.00,-0.75) |
| Paraguay | male | 25.36(21.90,29.23) | 35.28(32.39,38.37) | 1.29(0.93,1.65) | 25.36(21.90,29.23) | 35.28(32.39,38.37) | 1.29(0.93,1.65) |
| Peru | male | 52.26(50.08,54.52) | 67.30(65.46,69.18) | 1.79(1.24,2.34) | 52.26(50.08,54.52) | 67.30(65.46,69.18) | 1.79(1.24,2.34) |
| Philippines | male | 45.01(43.83,46.22) | 47.94(47.06,48.82) | 0.54(0.29,0.80) | 45.01(43.83,46.22) | 47.94(47.06,48.82) | 0.54(0.29,0.80) |
| Poland | male | 150.89(148.03,153.80) | 91.13(88.70,93.62) | -2.69(-3.22,-2.16) | 150.89(148.03,153.80) | 91.13(88.70,93.62) | -2.69(-3.22,-2.16) |
| Portugal | male | 106.96(102.35,111.73) | 36.82(33.79,40.07) | -4.05(-4.26,-3.84) | 106.96(102.35,111.73) | 36.82(33.79,40.07) | -4.05(-4.26,-3.84) |
| Puerto Rico | male | 126.44(118.13,135.19) | 86.37(78.50,94.82) | -2.36(-3.02,-1.70) | 126.44(118.13,135.19) | 86.37(78.50,94.82) | -2.36(-3.02,-1.70) |
| Qatar | male | 51.62(39.93,66.51) | 36.71(31.73,42.54) | -0.53(-0.86,-0.20) | 51.62(39.93,66.51) | 36.71(31.73,42.54) | -0.53(-0.86,-0.20) |
| Republic of Korea | male | 194.82(192.18,197.50) | 30.81(29.61,32.05) | -7.56(-8.27,-6.84) | 194.82(192.18,197.50) | 30.81(29.61,32.05) | -7.56(-8.27,-6.84) |
| Republic of Moldova | male | 114.29(107.20,121.75) | 38.70(33.86,44.12) | -2.70(-3.13,-2.27) | 114.29(107.20,121.75) | 38.70(33.86,44.12) | -2.70(-3.13,-2.27) |
| Romania | male | 121.61(118.34,124.94) | 75.87(72.63,79.22) | -1.30(-1.46,-1.15) | 121.61(118.34,124.94) | 75.87(72.63,79.22) | -1.30(-1.46,-1.15) |
| Russian Federation | male | 69.02(68.07,69.99) | 36.76(35.97,37.55) | -1.78(-2.44,-1.11) | 69.02(68.07,69.99) | 36.76(35.97,37.55) | -1.78(-2.44,-1.11) |
| Rwanda | male | 298.05(289.00,307.33) | 115.39(111.44,119.45) | -4.52(-5.11,-3.92) | 298.05(289.00,307.33) | 115.39(111.44,119.45) | -4.52(-5.11,-3.92) |
| Saint Kitts and Nevis | male | 153.95(81.17,269.38) | 53.29(19.42,119.21) | -4.47(-5.09,-3.86) | 153.95(81.17,269.38) | 53.29(19.42,119.21) | -4.47(-5.09,-3.86) |
| Saint Lucia | male | 203.97(152.50,269.05) | 87.76(58.99,126.36) | -3.47(-3.86,-3.07) | 203.97(152.50,269.05) | 87.76(58.99,126.36) | -3.47(-3.86,-3.07) |
| Saint Vincent and the Grenadines | male | 131.90(88.44,192.00) | 71.65(40.14,118.55) | -2.76(-3.15,-2.37) | 131.90(88.44,192.00) | 71.65(40.14,118.55) | -2.76(-3.15,-2.37) |
| Samoa | male | 11.60(2.84,34.08) | 14.44(5.07,33.10) | 0.81(0.71,0.91) | 11.60(2.84,34.08) | 14.44(5.07,33.10) | 0.81(0.71,0.91) |
| San Marino | male | 121.25(42.59,273.85) | 72.15(15.92,206.49) | -1.39(-1.54,-1.23) | 121.25(42.59,273.85) | 72.15(15.92,206.49) | -1.39(-1.54,-1.23) |
| Sao Tome and Principe | male | 24.22(6.41,66.98) | 35.11(19.91,57.76) | 1.20(0.81,1.60) | 24.22(6.41,66.98) | 35.11(19.91,57.76) | 1.20(0.81,1.60) |
| Saudi Arabia | male | 14.69(13.49,15.97) | 11.30(10.68,11.95) | -0.61(-0.73,-0.48) | 14.69(13.49,15.97) | 11.30(10.68,11.95) | -0.61(-0.73,-0.48) |
| Senegal | male | 30.37(27.35,33.66) | 26.42(24.61,28.33) | -0.15(-0.34,0.04) | 30.37(27.35,33.66) | 26.42(24.61,28.33) | -0.15(-0.34,0.04) |
| Serbia | male | 149.28(143.74,154.99) | 57.21(53.55,61.07) | -3.08(-3.23,-2.93) | 149.28(143.74,154.99) | 57.21(53.55,61.07) | -3.08(-3.23,-2.93) |
| Seychelles | male | 37.24(13.16,85.84) | 34.92(14.84,72.38) | 0.43(-0.27,1.13) | 37.24(13.16,85.84) | 34.92(14.84,72.38) | 0.43(-0.27,1.13) |
| Sierra Leone | male | 42.08(37.47,47.13) | 37.66(34.80,40.71) | -0.19(-0.32,-0.06) | 42.08(37.47,47.13) | 37.66(34.80,40.71) | -0.19(-0.32,-0.06) |
| Singapore | male | 68.28(62.54,74.42) | 18.73(15.98,21.90) | -4.52(-4.71,-4.33) | 68.28(62.54,74.42) | 18.73(15.98,21.90) | -4.52(-4.71,-4.33) |
| Slovakia | male | 190.60(182.29,199.20) | 76.58(70.78,82.79) | -3.28(-3.59,-2.96) | 190.60(182.29,199.20) | 76.58(70.78,82.79) | -3.28(-3.59,-2.96) |
| Slovenia | male | 122.65(111.86,134.25) | 46.13(38.51,54.94) | -4.02(-4.55,-3.49) | 122.65(111.86,134.25) | 46.13(38.51,54.94) | -4.02(-4.55,-3.49) |
| Solomon Islands | male | 12.71(5.16,26.91) | 16.11(9.94,24.85) | 0.89(0.74,1.04) | 12.71(5.16,26.91) | 16.11(9.94,24.85) | 0.89(0.74,1.04) |
| Somalia | male | 64.70(60.69,68.91) | 84.45(81.66,87.33) | 1.17(0.97,1.36) | 64.70(60.69,68.91) | 84.45(81.66,87.33) | 1.17(0.97,1.36) |
| South Africa | male | 70.85(68.88,72.85) | 52.76(51.50,54.05) | -1.64(-2.20,-1.07) | 70.85(68.88,72.85) | 52.76(51.50,54.05) | -1.64(-2.20,-1.07) |
| South Sudan | male | 41.64(37.98,45.59) | 57.51(53.87,61.36) | 0.96(0.62,1.30) | 41.64(37.98,45.59) | 57.51(53.87,61.36) | 0.96(0.62,1.30) |
| Spain | male | 61.29(59.53,63.09) | 29.40(28.07,30.78) | -2.86(-3.13,-2.58) | 61.29(59.53,63.09) | 29.40(28.07,30.78) | -2.86(-3.13,-2.58) |
| Sri Lanka | male | 77.35(74.54,80.24) | 65.18(62.68,67.76) | -1.31(-1.87,-0.74) | 77.35(74.54,80.24) | 65.18(62.68,67.76) | -1.31(-1.87,-0.74) |
| Sudan | male | 15.33(14.03,16.72) | 13.55(12.78,14.35) | -0.21(-0.33,-0.10) | 15.33(14.03,16.72) | 13.55(12.78,14.35) | -0.21(-0.33,-0.10) |
| Suriname | male | 289.27(252.88,330.02) | 142.35(120.57,167.00) | -2.66(-3.01,-2.31) | 289.27(252.88,330.02) | 142.35(120.57,167.00) | -2.66(-3.01,-2.31) |
| Sweden | male | 74.13(69.81,78.66) | 22.26(20.06,24.66) | -3.59(-3.91,-3.27) | 74.13(69.81,78.66) | 22.26(20.06,24.66) | -3.59(-3.91,-3.27) |
| Switzerland | male | 116.29(110.43,122.40) | 32.58(29.63,35.78) | -3.90(-4.04,-3.76) | 116.29(110.43,122.40) | 32.58(29.63,35.78) | -3.90(-4.04,-3.76) |
| Syrian Arab Republic | male | 24.49(22.48,26.66) | 16.35(14.42,18.53) | -1.09(-1.36,-0.82) | 24.49(22.48,26.66) | 16.35(14.42,18.53) | -1.09(-1.36,-0.82) |
| Taiwan (Province of China) | male | 14.64(13.57,15.77) | 48.85(46.62,51.17) | 5.66(4.52,6.81) | 14.64(13.57,15.77) | 48.85(46.62,51.17) | 5.66(4.52,6.81) |
| Tajikistan | male | 14.31(12.01,16.95) | 8.78(7.56,10.14) | -1.78(-2.14,-1.42) | 14.31(12.01,16.95) | 8.78(7.56,10.14) | -1.78(-2.14,-1.42) |
| Thailand | male | 72.45(70.98,73.94) | 89.12(87.31,90.96) | -1.12(-1.90,-0.33) | 72.45(70.98,73.94) | 89.12(87.31,90.96) | -1.12(-1.90,-0.33) |
| Timor-Leste | male | 35.59(26.94,46.33) | 39.43(32.16,48.06) | 0.33(-0.14,0.80) | 35.59(26.94,46.33) | 39.43(32.16,48.06) | 0.33(-0.14,0.80) |
| Togo | male | 54.50(48.74,60.82) | 59.22(55.44,63.21) | 0.33(0.09,0.57) | 54.50(48.74,60.82) | 59.22(55.44,63.21) | 0.33(0.09,0.57) |
| Tokelau | male | 8.76(0.00,1595.69) | 10.07(0.00,1660.15) | 0.13(-0.03,0.29) | 8.76(0.00,1595.69) | 10.07(0.00,1660.15) | 0.13(-0.03,0.29) |
| Tonga | male | 7.29(0.24,45.52) | 9.47(0.83,40.67) | 0.92(0.66,1.18) | 7.29(0.24,45.52) | 9.47(0.83,40.67) | 0.92(0.66,1.18) |
| Trinidad and Tobago | male | 122.13(108.84,136.66) | 90.93(79.34,103.82) | -1.01(-1.29,-0.72) | 122.13(108.84,136.66) | 90.93(79.34,103.82) | -1.01(-1.29,-0.72) |
| Tunisia | male | 44.10(40.96,47.44) | 44.22(41.46,47.13) | -0.19(-0.30,-0.09) | 44.10(40.96,47.44) | 44.22(41.46,47.13) | -0.19(-0.30,-0.09) |
| Turkey | male | 35.20(34.15,36.29) | 34.82(33.91,35.74) | 0.69(-0.26,1.65) | 35.20(34.15,36.29) | 34.82(33.91,35.74) | 0.69(-0.26,1.65) |
| Turkmenistan | male | 35.00(30.81,39.64) | 18.61(16.16,21.33) | -1.75(-2.20,-1.29) | 35.00(30.81,39.64) | 18.61(16.16,21.33) | -1.75(-2.20,-1.29) |
| Tuvalu | male | 15.89(0.00,275.07) | 18.05(0.01,188.95) | 0.57(0.41,0.74) | 15.89(0.00,275.07) | 18.05(0.01,188.95) | 0.57(0.41,0.74) |
| Uganda | male | 78.84(75.69,82.10) | 134.67(132.11,137.27) | 0.98(0.39,1.57) | 78.84(75.69,82.10) | 134.67(132.11,137.27) | 0.98(0.39,1.57) |
| Ukraine | male | 64.66(63.05,66.30) | 38.57(37.12,40.08) | -1.44(-1.91,-0.97) | 64.66(63.05,66.30) | 38.57(37.12,40.08) | -1.44(-1.91,-0.97) |
| United Arab Emirates | male | 86.37(78.09,95.44) | 97.35(91.56,103.44) | 1.03(0.12,1.95) | 86.37(78.09,95.44) | 97.35(91.56,103.44) | 1.03(0.12,1.95) |
| United Kingdom | male | 67.44(65.86,69.04) | 30.89(29.84,31.97) | -3.05(-3.37,-2.72) | 67.44(65.86,69.04) | 30.89(29.84,31.97) | -3.05(-3.37,-2.72) |
| United Republic of Tanzania | male | 66.52(64.12,68.99) | 58.36(56.92,59.84) | -0.62(-0.78,-0.47) | 66.52(64.12,68.99) | 58.36(56.92,59.84) | -0.62(-0.78,-0.47) |
| United States of America | male | 89.57(88.74,90.40) | 44.67(44.12,45.23) | -2.31(-2.60,-2.02) | 89.57(88.74,90.40) | 44.67(44.12,45.23) | -2.31(-2.60,-2.02) |
| United States Virgin Islands | male | 144.65(95.28,211.28) | 151.96(87.85,246.21) | -0.05(-0.53,0.43) | 144.65(95.28,211.28) | 151.96(87.85,246.21) | -0.05(-0.53,0.43) |
| Uruguay | male | 39.60(34.56,45.17) | 40.34(35.40,45.79) | 0.08(-0.24,0.40) | 39.60(34.56,45.17) | 40.34(35.40,45.79) | 0.08(-0.24,0.40) |
| Uzbekistan | male | 32.32(30.60,34.12) | 55.44(53.71,57.21) | 2.74(1.76,3.72) | 32.32(30.60,34.12) | 55.44(53.71,57.21) | 2.74(1.76,3.72) |
| Vanuatu | male | 16.00(4.65,40.75) | 20.86(10.85,36.67) | 0.71(0.64,0.78) | 16.00(4.65,40.75) | 20.86(10.85,36.67) | 0.71(0.64,0.78) |
| Venezuela (Bolivarian Republic of) | male | 86.84(83.96,89.80) | 56.80(54.56,59.12) | -1.53(-1.80,-1.26) | 86.84(83.96,89.80) | 56.80(54.56,59.12) | -1.53(-1.80,-1.26) |
| Viet Nam | male | 60.98(59.65,62.34) | 88.16(86.83,89.50) | 1.74(1.46,2.02) | 60.98(59.65,62.34) | 88.16(86.83,89.50) | 1.74(1.46,2.02) |
| Yemen | male | 17.04(15.30,18.93) | 13.16(12.29,14.07) | -0.74(-0.83,-0.65) | 17.04(15.30,18.93) | 13.16(12.29,14.07) | -0.74(-0.83,-0.65) |
| Zambia | male | 111.96(106.51,117.65) | 134.96(131.31,138.69) | 0.08(-0.28,0.44) | 111.96(106.51,117.65) | 134.96(131.31,138.69) | 0.08(-0.28,0.44) |
| Zimbabwe | male | 11.29(9.72,13.05) | 57.34(54.60,60.19) | 6.70(4.86,8.57) | 11.29(9.72,13.05) | 57.34(54.60,60.19) | 6.70(4.86,8.57) |
